# Ozonation of Pharmaceuticals and Their Human Metabolites in Wastewater—Insights from Laboratory Experiments and Field Data

**DOI:** 10.1021/acs.est.5c08128

**Published:** 2025-09-22

**Authors:** Corina Meyer, Pia M. Kronsbein, Valentin Rougé, Urs von Gunten, Christa S. McArdell, Juliane Hollender

**Affiliations:** † 28499 Eawag: Swiss Federal Institute of Aquatic Science and Technology, Ueberlandstrasse 133, 8600 Duebendorf, Switzerland; ‡ Institute of Biogeochemistry and Pollutant Dynamics, 5205 ETH Zurich, Universitaetstrasse 16, 8092 Zurich, Switzerland; ¶ School of Architecture, Civil and Environmental Engineering (ENAC), Ecole Polytechnique Fédérale Lausanne (EPFL), 1015 Lausanne, Switzerland

**Keywords:** Pharmaceuticals, Human metabolites, Wastewater treatment, Ozonation, Multicompound competition kinetics

## Abstract

Pharmaceuticals and their human metabolites are contaminants of emerging concern in aquatic environments. While monitoring usually targets parent compounds, metabolites, often excreted at higher loads, are largely overlooked. This study investigates the behavior of both during wastewater treatment, focusing on ozonation as an advanced treatment step. Second-order rate constants for reactions of 87 parent compounds and 130 metabolites with ozone were determined using a multicompound competition kinetics method, enabling exploration of functional group-specific reactivity trends. Aromatic hydroxylation generally increased ozone reactivity (up to 5 orders of magnitude), whereas *N*-oxides (up to 5 orders of magnitude) and *N*-dealkylated metabolites (up to 2 orders of magnitude) showed reduced reactivity compared to their parents. These constants, combined with predicted second-order rate constants for the reaction with hydroxyl radicals, as well as estimated ozone exposures and experimentally determined hydroxyl radical exposures, were used to model the abatement in three Swiss wastewater treatment plants. Modeled and observed abatement agreed well. A derivative-based sensitivity analysis highlighted ozone exposure as most crucial for moderately to highly ozone-reactive compounds, whereas hydroxyl radical exposure dominated for ozone-resistant compounds. This study emphasizes the importance of considering both parent compounds and metabolites in wastewater treatment processes to address contaminants holistically.

## Graphical abstract

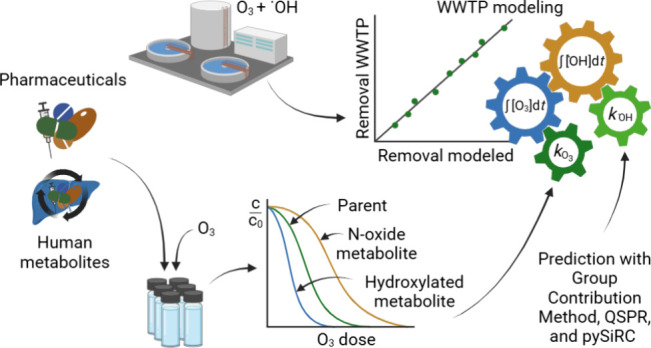

## 1 Introduction

Conventional municipal wastewater treatment plants (WWTPs) were primarily constructed for the removal of particles, nutrients, and organic matter but are not specifically designed to target micropollutants such as pharmaceuticals and their human metabolites.^1,2^ Since such compounds are often not well sorbing nor biodegradable, WWTP discharges are a significant source of pharmaceutical residues contaminating the receiving water bodies.^3,4^ In response, Switzerland introduced a new Water Protection Act that has been effective since 2016. Ozonation and adsorption to powdered or granular activated carbon (GAC) are the primary technologies selected for this purpose.^5–7^ In line with Switzerland, the revised European Union Urban Wastewater Treatment Directive, which came into force in 2025,^8^ mandates the upgrade of selected WWTPs with advanced treatment processes. The performance of these upgraded WWTPs is assessed according to both directives by the removal of 12 indicator substances, with an average elimination efficiency of more than 80% required.^8,9^

The strong oxidative property of ozone (O_3_) makes it effective for the abatement of micropollutants,^2,10–12^ including pharmaceuticals and estrogenic compounds.^6,13,14^ Since the early 2000s, ozonation has been tested in pilot WWTPs as an advanced treatment.^15^ Over the years, ozonation has become a key technology that complements conventional wastewater treatment to improve effluent quality and minimize environmental impact of micropollutants.^5,16^ Ozone is a selective oxidant, exhibiting high reactivity toward electron-rich functional groups such as activated aromatic compounds, olefins, neutral amines, and reduced sulfur compounds, while showing limited reactivity with electron-deficient functional groups.^17,18^ Moreover, O_3_ generates hydroxyl radicals (^•^OH) during its decomposition in water, enabling a powerful but less selective secondary oxidation.^5,17,19,20^ This mechanism with *in situ* formation of ^•^OH, referred to as advanced oxidation processes, enables the abatement of micropollutants that are recalcitrant to O_3_.^21^ It is particularly relevant in wastewater treatment systems with high levels of dissolved organic matter (DOM), which enhances ^•^OH formation.^22^

To assess the reactivity of micropollutants with ozone in aqueous solution, about 500 second-order rate constants for the reactions of inorganic and organic compounds with O_3_ and about 2000 for the reactions with ^•^OH have been determined to date.^23^ Moreover, the formation of ozonation byproducts (reaction products of O_3_ and ^•^OH with the wastewater matrix)^24–27^ and ozonation transformation products (reactions products of O_3_ and ^•^OH with the micropollutants)^28–32^ have been studied. The mitigation of their possible adverse effects is often addressed by post-treatment by either biological systems (sand filtration) or adsorptive/biological systems based on GAC filtration.^5,6,31,33,34^ However, most studies on micropollutants have focused primarily on active ingredients (parent compounds),^17^ overlooking the fact that human metabolites of pharmaceuticals often enter WWTPs in loads exceeding those of the parent compounds^35,36^ and can contribute significantly to toxicity.^37^ These metabolites arise from two main types of metabolization reactions: phase I reactions, which typically involve functionalization processes such as oxidation, reduction, or hydrolysis, and phase II reactions, which involve conjugation with polar moieties (e.g., glucuronidation, sulfation, acetylation) to increase solubility and facilitate excretion.^38^ Previously, the ozone reactivities of only few metabolites of sulfamethoxazole,^39,40^ carbamazepine,^41–43^ venlafaxine,^43–45^ and citalopram^43,45,46^ were studied in detail. Understanding the behavior of human pharmaceutical metabolites during ozonation is crucial, particularly since a previous study on a selection of three parent compounds and eight metabolites showed that the metabolites were less efficiently abated than the respective parent compounds.^47^ This highlights the importance of considering both the parent compounds and their metabolites to assess the ozonation efficacy.

To evaluate and compare the ozone efficiency for the abatement of parent compounds and their metabolites, experimental second-order rate constants for the reaction with ozone are preferred. If such experimental values are not available, values predicted by quantitative structure–activity relationships (QSARs) based on Hammett/Taft constants or other physicochemical properties can be applied.^48,49^ However, the available data limit QSAR predictions to compounds with common structural features, excluding less studied moieties, such as heterocycles or reduced sulfur compounds. Additionally, QSARs rely on Hammett/Taft constants, which are not always available in the literature. The pH sensitivity of the ozone reactivity of certain micropollutants further complicates predictions, because p*K*_a_ values remain challenging to estimate accurately.^50^ An alternative approach involves quantum-chemical calculations combined with QSARs,^29,51,52^ which can provide valuable insights and typically show results of similar quality as conventional QSAR approaches.

The aim of this study was to enhance the understanding of the ozone reactivities of both parent pharmaceutical compounds and their human metabolites for a broad range of substance classes with different functional groups and subsequently during wastewater ozonation. Second-order rate constants for the reactions of more than 200 compounds with ozone were determined in laboratory experiments. To cope with the large number of compounds, multicompound competition kinetics were applied, by mixing several compounds and including reference compounds with known second-order rate constants (*k*_O_3__) for their reactions with ozone.^53^ The mixtures of compounds were created by ensuring that structurally similar compounds and compounds with identical exact masses were not present in the same mixture to disentangle abatement and potential formation of ozonation transformation products identical to human metabolites and to avoid analytical problems. Moreover, the mixtures covered different, narrow reactivity ranges and were generated based on *k*_O_3__ predictions from QSARs. The second-order rate constants for the reactions with ^•^OH were obtained by predictions. Subsequently, these second-order rate constants for the reactions with O_3_ and ^•^OH were used together with O_3_ exposure estimations and experimentally determined ^•^OH exposures to model the abatement of selected compounds during ozonation steps of three previously sampled WWTPs.^35,36^

## 2 Materials and Methods

### 2.1 Compound Selection

Parent pharmaceutical compounds and their human metabolites analyzed in this study were selected using pharmaceutical consumption data from Switzerland (2014–2016), provided by the Swiss Federal Office for the Environment, as well as metabolites identified through a literature review.^35,54,55^ These compounds were analyzed by target and suspect screening in wastewater samples from three Swiss WWTPs in previous studies.^35,36^ Target screening covered 284 parent compounds and 88 metabolites, and 6 parent compounds and 66 metabolites without reference standards identified from a suspect screening were included. For 89 compound pairs linked by human metabolism, second-order rate constants for the reactions with O_3_ were measured. To enhance the knowledge on ozone reactivity, the study was expanded to include additional compounds and their metabolites or structurally similar substances (55 compounds), not previously analyzed in the WWTPs. Detailed information on all studied compounds and their reference materials is provided in SI-A1.

### 2.2 Prediction of Second-Order Rate Constants for Reactions with Ozone

Prior to experimental determination, available *k*_O_3__ values at pH 7 and 8 of the 329 compounds detected previously in wastewater and with the available in-house reference standard were compiled from literature (13%) or predicted via QSARs (57%).^48^ For compounds with no literature *k*_O_3__ or applicable QSAR, second-order rate constants were estimated from structurally similar compounds (30%). In such cases, the most ozone-reactive structural moiety relevant to the compound was identified, and its reported second-order rate constant for the reaction with ozone was directly adopted as a conservative estimate. The applied QSARs were developed by Lee et al.^48^ and can be used to predict *k*_O_3__ values for olefins, benzenes, phenols, phenolates, anilines, and amines by considering the published correlations. The correlations of second-order rate constants for reactions with ozone and Hammett or Taft constants consider the electronic effects of substituents on the reactivity. The olefin and amine QSARs use Taft (σ*) constants,^56^ while the benzene, phenol, phenolate, and aniline QSARs are based on Hammett (σ^+^ and σ^–^) constants.^57,58^ Due to the structural complexity of some compounds, exact substituent constants were not always available. Thus, Hammett/Taft constants of substituents with similar expected electron-withdrawing or electron-donating capacities were selected. Cyclic systems containing amines or olefins were reduced to their linear analogs, while polycyclic phenol­(ate)­s were approximated as monocyclic structures, although polycycles tend to have higher reactivities.^59^ For compounds with acid–base speciation, including amines and phenols, the apparent second-order rate constants were calculated based on species-specific second-order rate constants and their speciation at pH 7. p*K*_a_ values predicted by JChem of ChemAxon^60^ were used. In the case of heterocyclic or sulfur-containing compounds, *k*_O_3__ were approximated from structurally related compounds with available literature values.^61–64^ Overall, QSARs were used to predict *k*_O_3__ values for 189 compounds without experimental data, whereas the remaining 100 compounds were approximated from structurally similar compounds. Details of the prediction for each compound, including the specific Hammett/Taft substituent constants used, the p*K*_*a*_ values applied for speciation, and the calculation of second-order rate constants at pH 7 and 8, are provided in SI-A2.

### 2.3 Experimental Determination of Second-Order Rate Constants for Reactions with Ozone (*k*_app,O_3__)

#### 2.3.1 Ozonation Experiments

The applied multicompound approach is based on competition kinetics.^53^ Fifteen competitor compounds, mostly micropollutants with various functional groups (Table SI-B2), were selected that cover a wide range of partially overlapping second-order rate constants (1 to 10^8^ M^–1^ s^–1^). The observed abatement of two specific compounds, including a target compound (pharmaceutical or metabolite) and its competitor, is linked, as both experience the same ozone exposure, regardless of matrix complexity.^17,53^ To apply this method for the experimental determination of apparent *k*_O_3__ values at pH 7 (*k*_app,O_3__), target compounds were grouped based on the predicted *k*_app,O_3__ into five mixtures covering different reactivity ranges (10^–2^ to 5 × 10^3^ M^–1^ s^–1^, 5 to 10^6^ M^–1^ s^–1^, 10^3^ to 5 × 10^6^ M^–1^ s^–1^, 10^5^ to 5 × 10^9^ M^–1^ s^–1^, and 10^7^ to 10^9^ M^–1^ s^–1^). To prevent interferences, compounds with identical exact masses and those that could form through the oxidation of other target compounds (parents and metabolites) were placed in different mixtures. This means that a maximum of four metabolites of a specific parent compound could be covered within the experimental setup provided that their predicted reactivities match one of the five different reactivity groups. These prerequisites, together with the exclusion of some compounds for which experimental *k*_app,O_3__ are already available, reduced the number of compounds studied experimentally from 329 to 228.

Stock solutions of the five mixtures and the competitors were prepared in ethanol (10 mg L^–1^). To ensure the absence of ethanol during ozonation, which can promote ^•^OH formation during ozonation,^19,32,65^ an aliquot of the respective target compound mixture and the competitor mixture were combined with an equal volume of ultrapure water, generated with an ultrapure laboratory water system (Sartorius Arium Pro, Germany; resistivity of 18.2 MΩ·cm). The mixtures were evaporated to dryness under a vacuum (Syncore Analyst R-12, BÜCHI Labortechnik AG, Switzerland, 110 rpm, 45 °C, two cycles of 10 min at 150 mbar, 30 min at 20 mbar). After the first cycle, 1 mL of ultrapure water was added to the vials to facilitate the removal of the remaining traces of ethanol. After redissolution in ultrapure water, phosphate buffer (4 mM, pH 7) and *tert*-butanol (*t*BuOH) (80 mM) for ^•^OH scavenging were added. The pH values of the mixtures ranged from 6.97 to 7.02. Aliquots of 2 mL with a competitor and target compound concentration of 10 μg L^–1^ each (11–85 nmol L^–1^) were distributed into 5 mL sample vials. Then, 2 mL of a prediluted ozone stock solution was added under vigorous stirring on a magnetic stirring plate. After 1 min, the stirring bar was removed and the vial closed. The ozone dosing solution was prepared from an ozone stock solution in ultrapure water. Ozone stock solutions were prepared by an ozone generator (BMT 803 BT, BMT Messtechnik, Germany), sparging O_3_-containing gas into ultrapure water, which was cooled in an ice bath.^53^ Stock solution concentrations of 1.5 mM to 2.0 mM were measured spectrophotometrically (Cary 100 Scan, Varian, U.S) at 260 nm in a 1 cm quartz cuvette (ϵ_260_ = 3200 M^–1^cm^–1^).^17^ Based on preliminary tests, used to fine-tune ozone doses and achieve a sufficient number of data points, the stoichiometric range of ozone doses (molar ratio between the sum of all compounds in a mixture and O_3_) was set from 0.0025 to 100. Experiments were performed at room temperature (22 ± 1 °C) and in triplicate on three separate days within 1 week maximum.

#### 2.3.2 HPLC-HRMS/MS Analysis

From each sample vial, 1 mL was transferred to short-thread vials for HPLC-HRMS/MS analysis and subsequently stored at 4 °C until measurement. After injection of 100 μL, chromatographic separation was achieved with a reversed-phase C18 column at 30 °C (Atlantis T3, 3 μm, 3.0 × 150 mm, Waters, U.S.) using ultrapure water and methanol, both with 0.1% formic acid, as mobile phases. Full scan data in both ionization modes were acquired simultaneously in switch mode (ESI+ and ESI−) on a high-resolution mass spectrometer (Q Exactive Plus, Thermo Fisher Scientific, U.S.). Full scan MS1 acquisition (*m*/*z*: 100 to 1000) was performed with a mass resolution of 140 000 at 200 *m*/*z*, followed by data-dependent MS/MS scans with a resolution of 17 500 at 200 *m*/*z* using higher energy collision-induced dissociation (HCD) and an isolation window of 1 Da. Peak integration was carried out using Skyline.^66–68^ For more details on sample analysis, see SI-B1.2.2.

#### 2.3.3 Determination of Second-Order Rate Constants

To experimentally determine apparent second-order rate constants at pH 7 (*k*_app,O_3__), competition kinetics were applied.^17^ The abatement of two compounds, i.e., of a target compound and a competitor, can be formulated as follows (eq 1):

1 $$\ln\left(\frac{A_{\mathrm{target}}}{A_{\mathrm{target},0}}\right)=\ln\left(\frac{A_{\mathrm{competitor}}}{A_{\mathrm{competitor},0}}\right)\cdot \frac{k_{\mathrm{app},O_{3},\mathrm{target}}}{k_{\mathrm{app},O_{3},\mathrm{competitor}}}$$

Instead of concentrations, as typically done, peak areas (*A*) from HPLC-HRMS/MS analyses were directly used since the peak intensity response was linear and only the relative abatement is relevant. From the slope of the linear regression of eq 1 and the known *k*_app,O_3_,competitor_, *k*_app,O_3__ of the target compound can be calculated.

The used competitors with their *k*_app,O_3__ values are provided in Table SI-B2, whereby the acid–base speciation of pH-dependent competitors was considered. The selected competitors have been previously tested and are robust for *k*_O_3__ determination.^53^ For the correlations between a target compound/competitor pair according to eq 1, only data points between 10 and 90% abatement were considered. Each target compound was initially correlated with every competitor to evaluate possible pairs, but *k*_app,O_3__ was only calculated if (i) there were at least 10 data points, (ii) the coefficient of determination (R^2^) was >0.9, (iii) the intercept was negligible (intercept <10 × slope), and (iv) *k*_app,O_3__ of target and competitor were within 1 order of magnitude, corresponding to a slope between 0.1 and 10. If multiple competitors fulfilled all of the aforementioned criteria for a given target compound, an average *k*_app,O_3__ and the corresponding standard deviation were calculated. More details on the applied method are provided elsewhere.^53^

### 2.4 Wastewater Sample Collection, Preparation, and Analysis

Wastewater samples for the previous studies^35,36^ were collected from three Swiss WWTPs, including Altenrhein, Neugut (Duebendorf), and Werdhoelzli (Zurich). These WWTPs were chosen since they are already equipped with an advanced treatment, including ozonation (Altenrhein additionally with a GAC filter) and apply different average specific ozone doses of 0.1 g_O_3__ g_DOC_^–1^, 0.4 g_O_3__ g_DOC_^–1^, and 0.6 g_O_3__ g_DOC_^–1^, respectively. A scheme of these WWTPs is displayed in Figure SI-B6. The samples were collected as 24 h flow-proportional composite samples before and after the ozonation process over five workdays and during dry weather conditions from Monday, February 28, to Friday, March 4, 2022. Additional details regarding the sampling methodology, the sample preparation, and the analysis are provided elsewhere.^36^ An additional 24 h flow-proportional secondary effluent composite sample of each WWTP prior to ozonation was collected on August 6, 2024 to determine ^•^OH exposures (see Section 2.5.2).

### 2.5 Abatement during Ozonation

Abatement of pharmaceuticals and their metabolites during ozonation of wastewater occurs by reaction with O_3_ and with ^•^OH formed from the reaction of O_3_ with DOM and its autocatalytic decomposition. Therefore, the abatement of pharmaceuticals during ozonation can be expressed as follows (eq 2):^69^

2 −ln([c]out[c]in)=kO3∫[O3]dt+kOH•∫[OH•]dt

where *k*_O_3__ and kOH• are the apparent second-order rate constants for the reactions of the pharmaceutical with O_3_ and ^•^OH, while ∫[O_3_]­d*t* and ∫[^•^OH]­d*t* are the O_3_ and ^•^OH exposures, respectively. Hence, the abatement of pharmaceuticals can be predicted and compared with field data if these four parameters are known. The procedure used to determine *k*_O_3__ values for the target compounds is described above, while the determination of the remaining three parameters is described in Sections 2.5.1 and 2.5.2.

#### Prediction of Second-Order Rate Constants for Reactions with Hydroxyl Radicals ( kOH• )

2.5.1

For ^•^OH, which react fast and close to the diffusion limit (∼1 × 10^10^ M^–1^ s^–1^) for most studied compounds,^17^ three different prediction approaches were tested. These include the group contribution method (GCM),^70^ a quantitative structure–property relationship (QSPR) approach based on molecular DRAGON descriptors,^71,72^ and a machine learning (ML) approach based on molecular fingerprints, called pySiRC (python Simulator of Rate Constant).^73,74^

The GCM models the second-order rate constant of an organic compound as the sum of four ^•^OH-initiated reactions, estimated using the activation energy (*E*_*a*_).^70^ Each reaction mechanism has a base activation energy (*E*_*a*_^0^) and contributions (*E*_*a*_^*R*_*i*_^) from functional groups at neighboring (α-position) or next-nearest (β-position) sites. These contributions were empirically determined with a large data set.^70^ The GCM accounts for four ^•^OH-initiated mechanisms in the aqueous phase: (1) H atom abstraction, (2) addition to alkenes, (3) addition to aromatic compounds, and (4) interaction with S-, N-, or P-containing compounds.^70^ For more details on GCM, see SI-B1.3.1.

In the QSPR model, multiple linear regression was used to identify a linear relationship between kOH• and molecular descriptors (DRAGON descriptors). These descriptors include electronegativity (Me), number of double bonds (nDB), number of primary alkyl halide substructures (CH2RX), number of acceptor atoms for hydrogen bonds (nHAcc), and three descriptors related to the topological structure of a compound (Mor27p, MATS2m, and Vindex).^72^ However, the DRAGON software used for the prediction of the molecular descriptors has been discontinued, requiring a combination of multiple other software applications for the prediction of the seven included descriptors. The descriptor MATS2m was calculated with the ChemoPy python package^75^ which is part of ChemDes.^76^ The other six descriptors were calculated with alvaDesc,^77^ whereby Mor27p required previous 3D structure generation, which was conducted with CORINA classic from the SMILES representation.^78–80^

pySiRC incorporates three ML algorithms (Neural Network (NN),^81^ Random Forest (RF),^82^ and XGBoost^83^) applied to two types of molecular fingerprints (MORGAN and MACCS), resulting in six ML models. This method differs from the GCM and QSPR models in that it does not rely on predefined mechanistic pathways or a small fixed set of descriptors but learns directly from the data. The molecular fingerprints convert a molecular structure into a binary sequence with each bit indicating the presence or absence of specific molecular fragments. These fingerprints are weighted differently based on their contribution to the prediction, allowing the model to emphasize the most relevant features for predicting rate constants.^73,74^

#### 2.5.2 ^•^OH and O_3_ Exposures

The remaining two parameters, ^•^OH and O_3_ exposures, depend on the water matrix and the specific ozone dose. To determine the ^•^OH exposure, fresh 24 h-composite samples were collected before ozonation at the three WWTPs, stored at 4 °C in the dark, and used for ozone exposure determination on the following day. Freezing and thawing processes of stored samples may alter the DOM and hence have an influence on the O_3_ and ^•^OH exposures.^84^ The ^•^OH exposures were calculated from the abatement of the ozone-recalcitrant compound *para*-chlorobenzoic acid (*p*CBA) ( kOH• = 5.2 × 10^9^ M^–1^ s^–1^)^69^ by applying the same specific ozone doses as in the WWTPs to 50 mL aliquots of secondary effluents at room temperature (22 ± 1 °C). The samples were analyzed by HPLC (Ultimate 3000, Thermo Fisher Scientific, U.S.) with a reversed-phase C18 column at 30 °C (XBridge, 3.5 μm, 2.1× 50 mm, Waters, U.S.) and a diode array detector (DAD) to detect *p*CBA by absorption at 240 nm.

The O_3_ exposures for the three WWTPs were derived from literature values of WWTPs with identical specific ozone doses and similar alkalinities, dissolved organic carbon (DOC), nitrite, and bromide concentrations.^85,86^

### 2.6 Calculation of Abatement Efficiency

To enable a comparison of laboratory and field data, the abatement of compounds during ozonation can be described as a relative abatement by eq 3, where *c*_in_ and *c*_out_ represent the concentrations before and after ozonation.

3 $$\mathrm{Abatement}\left[\%\right]=\left(1-\frac{{\left[c\right]}_{\mathrm{out}}}{{\left[c\right]}_{\mathrm{in}}}\right)\cdot 100$$

Combination of eq 2 and eq 3 yields eq 4, which enables the calculation of the relative abatement in WWTPs based on data obtained from laboratory experiments without knowing explicit concentrations.

4 Abatement⁡[%]=(1−e−kO3∫[O3]dt−kOH•∫[OH•]dt)·100

For field data with [*c*]_out_ below the limit of quantification (LOQ), a value of 0.5 × LOQ was applied. Compounds with effluent concentrations below the LOQ were in 94% of cases abated by >95%, thus the *ad hoc* assumption of 0.5 × LOQ in the effluent had only a small bias on the overall calculations. Unless otherwise specified, relative abatements represent averages over all five consecutive sampling days.

### 2.7 Sensitivity Analysis and Monte Carlo Sampling

A sensitivity analysis was performed to understand which of the four input variables (*k*_O_3__, kOH• , O_3_ exposure, and ^•^OH exposure) have the most significant effect on the abatement calculations in the WWTPs. For this purpose, a derivative-based sensitivity analysis was chosen, which quantifies the influence of a parameter on the relative abatement by evaluating the derivative of the abatement with respect to this parameter. Equation 4 was partially derived with respect to all four parameters for each individual compound. The resulting derivatives were normalized to create a dimensionless sensitivity index, simplifying the comparison of the inputs with different units and scales. For more details, see SI-B1.6.

To address variability among input parameters in the ozonation model and the resulting propagation of uncertainty in model predictions, a Monte Carlo approach was employed. Second-order rate constants for reactions with O_3_ and ^•^OH exposures were sampled as normal distributions with mean and standard deviations determined by laboratory experiments. For the kOH• values, the mean and standard deviation over all six *pySiRC* models were taken for normal distribution sampling. In contrast, a uniform distribution with ± 80% deviation was assumed for the ozone exposures.

## 3 Results and Discussion

### 3.1 Apparent Second-Order Rate Constants for Reactions with Ozone at pH 7 (*k*_app,O_3_,pH7_)

#### 3.1.1 Determination of *k*_app,O_3__ and Comparison with Literature and Predicted Values

The *k*_app,O_3__ determined experimentally for 218 of the 228 target compounds at pH 7 covered a wide reactivity range over 9 orders of magnitude from 10^–1^ M^–1^ s^–1^ to 10^8^ M^–1^ s^–1^ (SI-A3). Six compounds exhibited a *k*_app,O_3__ below the minimal determinable value given by the used competitors, and for four compounds analytical problems prevented *k*_app,O_3__ determination. To check the validity of the experimental values determined by the multicompound competition kinetics approach,^53^ 24 compounds with available literature values were included. Of these compounds, 15 show less than a factor of 2 deviation and 8 less than a factor of 5 (Figure 1 and SI-B3). Only the reactivity of telmisartan toward ozone (*k*_app,O_3__ = 120 ± 6 M^–1^ s^–1^) was underestimated by 3 orders of magnitude compared to *k*_app,O_3__ determined via competition kinetics using *p*-cresol as a reference (1.2 × 10^5^ M^–1^ s^–1^).^5^ Since the second-order rate constant of *p*-cresol is strongly pH-dependent,^59^ this may affect the accuracy of this determination with *p*-cresol. The *k*_app,O_3__ value for telmisartan determined with the competitors bezafibrate, carbofuran, and *N* ^4^-acetylsulfamethoxazole in this study appears plausible, given the expected reactivity of its benzimidazole moieties (2.2 M^–1^ s^–1^ ^87^) and the alkyl substitution. This effect of increasing *k*_O_3__ with increasing degree of alkyl substitution is also observed when moving from benzene (*k*_O_3__ = 2 M^–1^ s^–1^)^59^ to toluene (*k*_O_3__ = 14 M^–1^ s^–1^)^59^ and further to xylenes (*k*_O_3__ = 90 M^–1^ s^–1^ to 140 M^–1^ s^–1^).^88^ A similarly low second-order rate constant (*k*_app,O_3__ = 111 ± 5 M^–1^ s^–1^) was determined in another mixture in this study for telmisartan-O-acyl-glucuronide, where the benzimidazole moieties are also likely responsible for ozone reactivity. Overall, the good agreement with literature values (SI-A3) underlines the robustness of the employed method and the applicability to a broad selection of compounds, including synthetic and natural products.^53^

**Figure 1:**
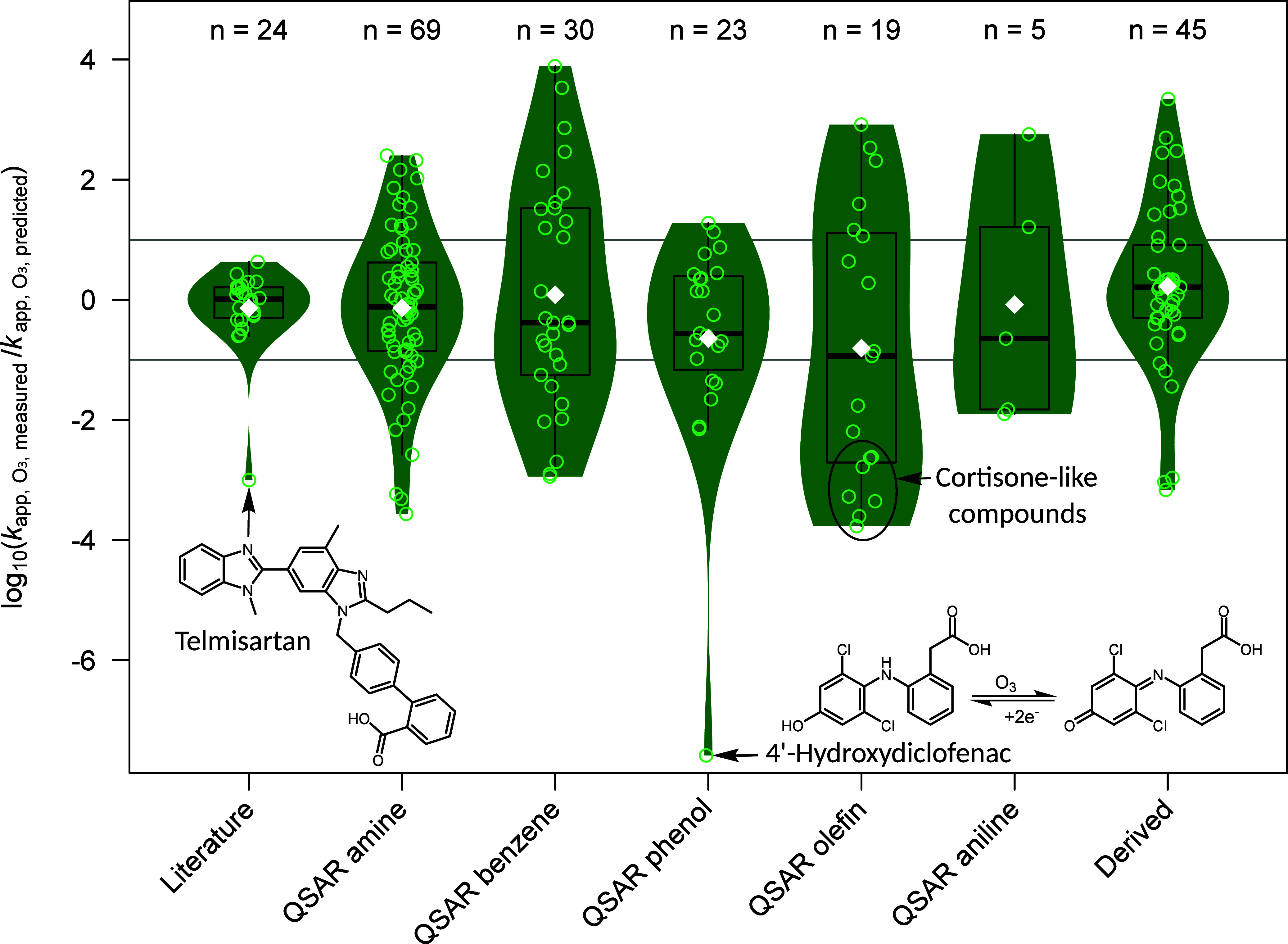
Comparison of the measured and predicted apparent second-order ozone rate constants. Logarithmic ratios of measured (*k*_app,O_3_,measured_) and predicted (*k*_app,O_3_,predicted_) apparent second-order rate constants for reactions with ozone at pH 7 were calculated for all studied compounds. The compounds are categorized in different groups. *Literature* shows the comparison to *k*_app,O_3__ from literature for 24 compounds,^5,10,15,88–103^ *QSARs* show the comparison to *k*_app,O_3__ derived from different QSARs for 146 compounds, and *Derived* depicts 45 compounds for which *k*_app,O_3__ were estimated from structurally similar compounds. The white diamonds indicate the mean values. Compounds with large discrepancies are labeled in the figure and are discussed in detail in the text. Gray lines indicate a deviation of 1 order of magnitude between measured and predicted *k*_app,O_3__. A list of all compounds included in this graph, together with their predicted and experimentally determined *k*_app,O_3__, is provided in SI-A3.

For compounds without literature values, *k*_O_3__ values were predicted by QSARs for amines, benzenes, phenols/phenolates, olefins, and anilines^48^ or estimated based on structurally similar compounds with known *k*_app,O_3__ values. The deviations between the predicted and measured values are larger than between values from the literature and our measurements due to the inherent uncertainties and limitations associated with the applied prediction models. As shown in Figure 1, only 49% of QSAR-predicted *k*_app,O_3__ were within 1 order of magnitude, while for 24% of the compounds, *k*_app,O_3__ differed by more than 2 orders of magnitude. A detailed discussion of all compounds showing a deviation of >|2.5| orders of magnitude is provided in SI-B4. One of the compounds exhibiting a much lower measured *k*_app,O_3__ than predicted by the phenol and phenolate QSARs is 4′-hydroxydiclofenac (*k*_app,O_3__ = 7.0 ± 0.4 M^–1^ s^–1^ vs *k*_pred,O_3__ = 2.7 × 10^8^ M^–1^ s^–1^). Although the electron-withdrawing effect of the two chlorine atoms at the phenol reduces the reactivity compared to phenol, this effect is compensated by the lower p*K*_a_ value and the hence higher fraction of the more reactive phenolate at pH 7.^88^ Thus, the chlorine substitution cannot explain the observed more than 7 orders of magnitude difference. The low *k*_app,O_3__ of 4’-hydroxydiclofenac may be explained by a secondary reaction of its oxidation product. Reaction with ozone is expected to proceed via a two-electron oxidation to form the dichloro-benzoquinoneimine. Similar 2,6-dihaloquinones are known to be unstable and can undergo reductive back-reactions,^104^ either spontaneously or via interaction with other components in the solution, regenerating 4′-hydroxydiclofenac. This hypothesis is supported when comparing with the other hydroxylated metabolite of diclofenac. 5-Hydroxydiclofenac, which has a much higher *k*_app,O_3__ value of (6.4 ± 1.4) × 10^7^ M^–1^ s^–1^, lacks electron-withdrawing substituents, and its corresponding benzoquinoneimine is expected to be more stable and less prone to such back-reactions. This difference in stability may explain the selectively lower apparent reactivity of 4′-hydroxydiclofenac in the competition kinetics experiments.

For compounds with cortisone-like structures (betamethasone, betamethasone-21-acetate, cortisone, hydrocortisone, methylprednisolone, prednisolone, and prednisone), *k*_O_3__ values were predicted with the olefin QSAR. However, the predicted values (*k*_app,O_3__ = 1.4 × 10^5^ M^–1^ s^–1^ to 2.0 × 10^5^ M^–1^ s^–1^) are up to 4 orders of magnitude higher than the measured rate constants (*k*_app,O_3__ = 24 M^–1^ s^–1^ to 436 M^–1^ s^–1^). A structurally similar compound, the hormone progesterone, has a *k*_app,O_3__ of 480 M^–1^ s^–1^,^105^ which is closer to the measured second-order rate constants. Hence, the olefin QSAR seems not to be applicable for predictions of *k*_O_3__ of compounds with cortisone-like structures, likely due to the nonplanar geometry of the olefin in the fused ring structure. Additionally, the deactivating effect of the carbonyl group seems underestimated by the olefin QSAR.^48^ This is supported by the *k*_O_3__ determined for cyclic olefins with *k*_O_3__ of about 1 × 10^3^ M^–1^ s^–1^.^106^

For 70 of the 143 compounds, second-order rate constants for reactions with ozone were within 1 order of magnitude compared to the measured *k*_app,O_3__. Current QSAR predictions rely on knowledge of the reactivity of certain functional groups; however, a sufficient database is not yet available for all of them, e.g., reduced sulfur compounds and heterocycles. Further complications arise for compounds undergoing acid–base speciation, like amines and phenols, for which accurate speciation depends on reliable p*K*_a_ predictions, which often overlook steric effects and hydrogen bonding. For most of the speciation-dependent reactive sites, the reactivity of a compound is driven by the deprotonated species, while the contribution of the protonated species is often negligible. For instance, benzoylecgonine has a predicted p*K*_a_ value of 9.14 ^60^ and a determined p*K*_a_ value of 11.8 ^107^ for the tertiary amine. As the neutral amine is the main reactive species, a difference of >2 pH-units would lead to a >2 orders of magnitude deviation from the predicted *k*_app,O_3__ at pH 7.

For 194 of the 218 target compounds with determined *k*_app,O_3__ in this study, no previous literature values exist. Newly measured apparent second-order rate constants encompass 67 compounds for which the amine group is the ozone-reactive site, 30 benzenes, 24 olefins, 22 phenolic compounds, 12 anilines, 3 reduced sulfur compounds, and 21 heterocycles. Furthermore, 12 *k*_app,O_3__ values were determined for compounds containing multiple possible reactive sites, where the identification of the most reactive site would need additional investigation. Especially for heterocycles, for which, if at all, second-order rate constants are only available for unsubstituted aromatic, e.g., imidazole, pyrimidine,^61,62^ and aliphatic cycles, e.g., piperazine, morpholine,^108^ our results can be valuable. The experimentally determined *k*_app,O_3__ from this study can contribute to improving predictions of second-order rate constants, including the development of QSARs for reactive moieties that currently lack sufficient experimental data.

#### 3.1.2 Reactivities of Parent Compounds and Their Metabolites

To evaluate whether specific human metabolism reactions consistently influence abatement rates during ozonation, we systematically compared second-order rate constants for the reactions with ozone for all parent-metabolite pairs categorized by their functional group changes at the site of metabolism. This approach allows for the identification of potential trends in the ozone reactivities of metabolites relative to their parent compounds. Figure 2 shows *k*_app,O_3__ ratios of metabolite–parent pairs for metabolizations by phase I reactions (hydroxylations, *N*-oxidation reactions, formation of carboxylic acids, and dealkylations) as well as phase II reactions (conjugations). Figures SI-B85 and SI-B89 additionally provide the structures of the discussed metabolite–parent pairs.

**Figure 2:**
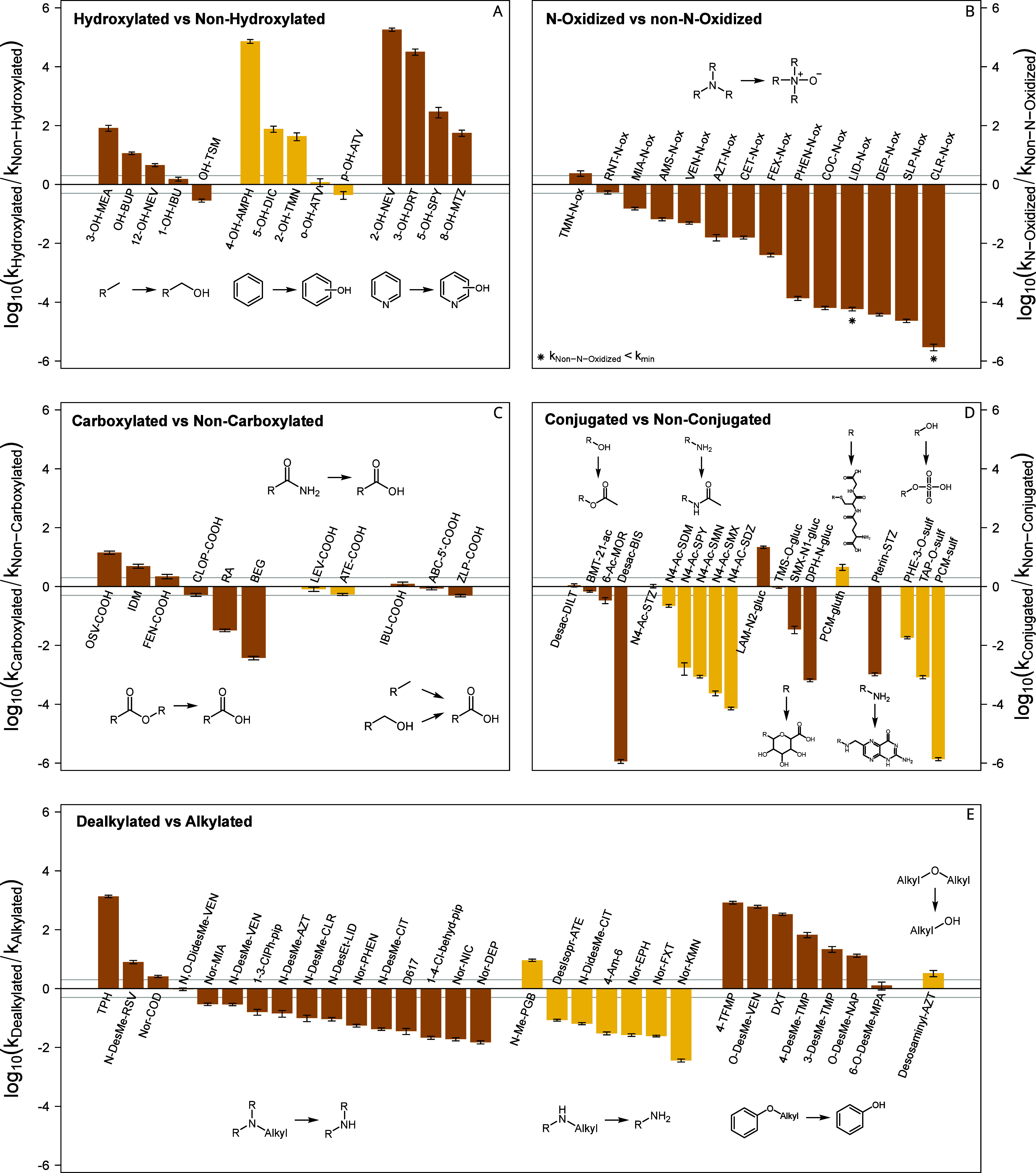
Ratios of measured apparent second-order rate constants at pH 7 for the reactions with ozone of the metabolite–parent pairs. The pairs are grouped according to the changes of functional groups by hydroxylations (A), *N*-oxidation reactions (B), formation of carboxylic acids (C), conjugation reactions (D), and dealkylations (E). Within the groups, different metabolic reactions (differentiated visually by two colors) leading to the respective metabolites are distinguished additionally. Apparent second-order rate constants lower than experimentally determinable are marked by ∗. For them, a *k*_app,O_3__ of 1 × 10^–1^ M^–1^ s^–1^ was assumed, corresponding to half of the lowest determined second-order rate constant, to enable ratio calculations. The gray lines indicate reactivity differences of a factor of 2. Figures SI-B85 to SI-B89 provide more detailed information and all abbreviations are listed in SI-A1.

##### 3.1.2.1 Effect of Hydroxylation on Ozone Reactivity

Hydroxylated aromatic metabolites generally react faster with ozone than their respective parent compounds with between 40 to 70 000 times higher *k*_app,O_3__ (Figures 2A and SI-B85). This can be attributed to the formation of phenolic-type structures, when the hydroxylation occurs at a benzene (e.g., 4-hydroxyamphetamine (4-OH-AMPH), 5-hydroxydiclofenac (5-OH-DIC)), or a pyridine ring (e.g., 2-hydroxynevirapine (2-OH-Nev), 3-hydroxydesloratadine (3-OH-DRT)). For *o*/*p*-hydroxyatorvastatin this increased reactivity due to the generation of phenolic structures was not observed, since the pyrrole ring, another highly reactive moiety (k_O_3__ = 1.4 × 10^6^ M^–1^ s^–1^ ^62^), is present in these two compounds. Thus, the additional phenol with a reactivity around 1 × 10^6^ M^–1^ s^–1^ at pH 7 ^88^ does not lead to a significant change in reactivity compared to the parent compound. However, if a hydroxylation occurs at an alkyl chain (e.g., 1-hydroxyibuprofen (1-OH-IBU), hydroxytorasemide (OH-TSM), and 12-hydroxyevirapine (12-OH-NEV)), no significant differences in ozone reactivities are observed. Two exceptions to this trend are hydroxybupropion (OH-BUP) and 3-hydroxymethylmefenamic acid (3-OH-MEA), where the metabolites react considerably faster than their parents. While bupropion (BUP) has a p*K*_a_ of 8.35 for the secondary amine,^109^ the predicted p*K*_a_ value of OH-BUP is 7.65,^60^ leading to a higher fraction of neutral amine for OH-BUP compared to BUP, explaining the increased reactivity at pH 7. However, for 3-OH-MEA the increased reactivity compared to the parent mefenamic acid remains unclear as the reactivity of both compounds is expected to be driven by the aniline-type moiety, with an apparent second-order rate constant around 1 × 10^6^ M^–1^ s^–1^.

##### 3.1.2.2 Effect of *N*-Oxide Formation on Ozone Reactivity

Contrary to hydroxylated metabolites, compounds with a tertiary amine group oxidized to an *N*-oxide generally have lower apparent second-order rate constants than their nonoxidized amine analogues (Figures 2B and SI-B86). A lower reactivity of the *N*-oxide compared to the amine is expected, since the lone pair is bound to oxygen, reducing the electron density of the nitrogen atom.^110^ The extent of reactivity changes between the parent and the metabolite depends on the importance of the amine relative to the compound’s second-most reactive functional group. For compounds with additional reactive groups, such as trimipramine (TMN) and mianserin (MIA), with an aniline-type moiety exhibiting reactivities around 5 × 10^4^ M^–1^ s^–1^ to 1 × 10^5^ M^–1^ s^–1^,^17^ similar reactivities of the parent and the *N*-oxidized metabolite are observed. Likewise, ranitidine (RNT) reacts mainly at other functional groups, like the thioether and the furan.^98^ Azithromycin-*N*-oxide (AZT-*N*-ox) possesses an additional tertiary amine, and its reactivity compared to the parent azithromycin (AZT) is around 2 orders of magnitude lower. This is caused by the higher predicted p*K*_a_ value of 11.24 ^60^ of this amine compared to the one that reacts first with ozone (p*K*_a_ = 8.74 ^111^), explaining the observed reactivity differences. For compounds where the amine is the sole reactive site (e.g., clarithromycin (CLR)), the reactivity difference between the parent and the *N*-oxide metabolite can exceed 4 orders of magnitude, while differences larger than 2 orders of magnitude were observed for compounds with benzene groups as second-most reactive sites (e.g., pheniramine (PHEN), lidocaine (LID)). Deprenyl (DEP) exhibits an alkyne group in addition to the tertiary amine. Although second-order rate constants for the reaction of ozone with alkynes are reported to be around 2 × 10^2^ M^–1^ s^–1^,^106^ the *k*_app,O_3__ for deprenyl-*N*-oxide (DEP-*N*-ox) was determined to be (3.5 ± 0.2) × 10^1^ M^–1^ s^–1^ in this study. It is hypothesized that the *N*-oxide in the β-position has a deactivating effect on the alkyne group.

##### 3.1.2.3 Effect of Carboxylic Acid Formation on Ozone Reactivity

The formation of carboxylic acids had the smallest effect on reactivity toward ozone among all analyzed changes in functional groups (Figures 2C and SI-B87). This is reasonable, since neither the resulting carboxylic acid, nor the precursors (esters, amides, aliphatic alcohols) have a high ozone reactivity. Nonetheless, if the formation of the carboxylic acid occurs nearby the reactive site, they may have an influence, especially since they are usually present as carboxylates at neutral pH. For example for oseltamivir and its human metabolite oseltamivir acid featuring a carboxylate, the olefin is responsible for the reaction with ozone. Since the carboxylate is conjugated to this olefin and has a slight electron-donating effect in contrast to the electron-withdrawing ester,^57^ a *k*_app,O_3__ increase by 1 order of magnitude was observed, in agreement with a previous study conducted on model olefinic compounds.^101,106^

##### 3.1.2.4 Effect of Conjugation on Ozone Reactivity

Acetylated phase II metabolites (Figures 2D and SI-B88) were found to generally react more slowly than their deacetylated forms. For sulfonamide antibiotics, the acetylation leads to an amide, deactivating the reactive aniline.^101^ Similarly, bisacodyl (BIS) is less reactive than desacetylbisacodyl (Desac-BIS), since the phenol is converted to phenyl acetate.^112^ For diltiazem (DILT) and sulfathiazole (STZ), the acetyl groups have no effect since the main reactivity is governed by the thioether or the thiazole moiety, respectively. Although a second-order rate constant for 1,3-thiazole of 1.5 × 10^2^ M^–1^ s^–1^ is reported,^113^ the attached sulfonamide with a predicted p*K*_a_ of 5.57 ^60^ for the metabolite *N* ^4^-acetylsulfathiazole and 5.73 ^60^ for the parent sulfathiazole is largely deprotonated at pH 7. This deprotonation introduces a strong electron-donating effect, which activates the thiazole ring toward electrophilic attack by ozone, resulting in a higher reactivity compared to that of the unsubstituted thiazole ring. Due to the similar p*K*_*a*_ values of thiazole and *N* ^4^-acetylsulfathiazole, a comparable reactivity of parent and metabolite of around 1 × 10^7^ M^–1^ s^–1^ is observed. SI-B4.6 provides a more detailed discussion. Similar to the acetylated metabolites, the studied sulfate metabolites are less reactive than their nonconjugated analogues, since the phenolic moieties are deactivated. The only studied glutathione metabolite, paracetamol-glutathione (PCM-gluth), reacts faster than paracetamol, since the attached glutathione moiety lowers the p*K*_a_ of the phenol from 9.4 ^114^ to 8.9,^60^ leading to a higher fraction of the more reactive phenolate species at pH 7. For glucuronide (gluc) metabolites, the reactivity also depends on the various functional groups of the compound. For diphenhydramine-*N*-glucuronide (DPH-*N*-gluc), the glucuronide covers the most reactive site in the molecule (tertiary amine) and the reactivity is reduced, while the glucuronide is far away from the most reactive sites in telmisartan (TMS, benzimidazole/benzene) resulting in an unchanged reactivity. For sulfamethoxazole-*N*1-glucuronide (SMX-*N*1-gluc), the ozone reactivity compared to the parent sulfamethoxazole (SMX) is reduced by more than 1 order of magnitude. With a p*K*_a_ of 5.7 ^115^ for SMX, the secondary sulfonamide in the *para*-position to the aniline is largely deprotonated. Since the glucuronidation converts the secondary sulfonamide into a tertiary sulfonamide, deprotonation is no longer possible, leading to a lower *k*_app,O_3__ for SMX-*N*1-gluc compared to SMX. In contrast, for lamotrigine (LAM), the glucuronide conjugation increases the reactivity from 17 M^–1^ s^–1^ to 3.7 × 10^2^ M^–1^ s^–1^. This conjugation alters the electronic structure of the *N* ^2^-atom and renders the *N*-heterocycle in lamotrigine-*N* ^2^-glucuronide (LAM-*N* ^2^-gluc) nonaromatic. It is hypothesized that the loss of aromaticity results in an olefin that is more reactive than the corresponding aromatic system. However, the olefin remains strongly deactivated by the adjacent amine, imine, and diazene groups, which explains why its *k*_app,O_3__ is several orders of magnitude lower than that of simple olefins.^106^

##### 3.1.2.5 Effect of Dealkylation on Ozone Reactivity

The effect of dealkylation on the ozone reactivity depends on the site of dealkylation (Figures 2E and SI-B89). Dealkylation of aromatic ethers may lead to the formation of phenols, increasing the reactivity toward ozone by 10- to 830-fold (e.g., O-desmethylvenlafaxine (O-DesMe-VEN), dextrorphan (DXT)), except for 6-O-desmethylmycophenolic acid (6-O–DesMe-MPA), where the parent mycophenolic acid already possesses a phenolic moiety. Although a larger difference between anisole (*k*_O_3__ = 290 M^–1^ s^–1^)^59^ and phenol (*k*_app,O_3__ around 1 × 10^6^ M^–1^ s^–1^ at pH 7)^88^ is expected, most of the observed reactivity differences between parent and *O*-dealkylated metabolite are smaller, since the parent compounds exhibit other, more reactive functional groups in addition to the anisole group, such as amines (venlafaxine (VEN), dextromethorphan (DXM)), 2,4-diaminopyrimidine moieties (3/4-desmethyltrimethoprim (3/4-DesMe-TMP)), or a naphthalene moiety (naproxen (NAP)). *N*-Dealkylations of tertiary or secondary amines decrease the reactivity of up to 2 orders of magnitude, since this leads to a loss of the inductive effect and the electron density at the nitrogen atom. The observed reactivity difference between tertiary and secondary amines is smaller than between secondary and primary amines.^116^

### kOH• Prediction

3.2

As kOH• values were not determined in this study, predicted kOH• values using the three different methods (GCM, QSPR, pySiRC) were compared to 44 kOH• values from literature (Figure 3). The GCM systematically overestimates second-order rate constants, on average by more than a factor of 2, with outliers reaching up to 1 order of magnitude. Since the GCM is based on Benson’s thermochemical group additivity^117^ and thus sums together all reactive sites which can react according to one of the four reaction mechanisms, the GCM is limited to calculating kOH• for low molecular weight compounds. In the current study many compounds have higher molecular weights, and therefore, the kOH• values easily exceed the diffusion limitation in the aqueous phase (∼1 × 10^10^ M^–1^ s^–1^). Further, the contributions from functional groups are limited regarding data availability and require approximations for others.

**Figure 3:**
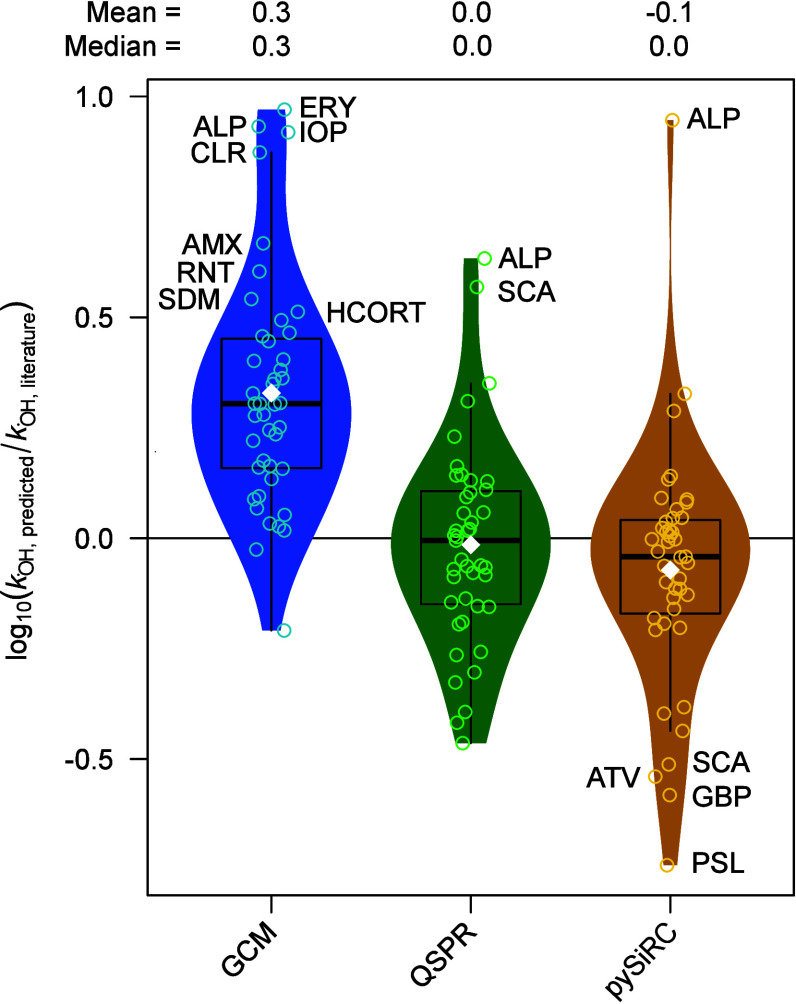
Prediction of kOH• . Violin plots of the logarithmic ratios of kOH• values from prediction and literature for 44 compounds (SI-A5) for the three different methods, group contribution method (GCM), quantitative structure property relationships (QSPR), and mean of 6 ML algorithms (pySiRC). The white diamonds indicate the mean values. Compounds with more than a factor of 2 deviation from literature are labeled: allopurinol (ALP), amoxicillin (AMX), atorvastatin (ATV), clarithromycin (CLR), erythromycin (ERY), gabapentin (GBP), hydrocortisone (HCORT), iopromide (IOP), prednisolone (PSL), succinic acid (SCA), ranitidine (RNT), and sulfadimethoxine (SDM).

In contrast, the second-order rate constants estimated by the QSPR model deviate on average only by about 10% from the literature values, as shown in Figure 3. Moreover, it has the advantage of being applicable to all organic compounds in an automated way. Despite the good agreement with literature values, the QSPR lacks mechanistic understanding because it relies on statistical correlations between molecular descriptors and reactivity rather than explicitly accounting for the underlying chemical reaction mechanisms and electronic effects.

To combine the advantages of both tools, the mechanistic interpretability of the GCM, and the automated calculation and the broader applicability domain of the QSPR model, pySiRC was used and the kOH• values of all six models, originating from the combination of three ML algorithms with two types of molecular fingerprints, were averaged and compared to literature values. Figure SI-B90 shows that all ML models perform similarly well with deviations of around 10%. An additional advantage is the availability of pySiRC as a web graphical platform, allowing a fast and user-friendly application. Due to the good model performance, the good comparability of the six models and especially of the mechanistic interpretability, it was decided to calculate a mean and standard deviation over all applicable pySiRC models per compound and to use them to describe a normal distribution for the Monte Carlo approach when modeling the abatement of pharmaceuticals during ozonation by eq 2.

### 3.3 ^•^OH and O_3_ Exposure

The secondary effluent samples obtained during the 2024 sampling campaign were used to determine the ^•^OH exposure. Additionally, they were characterized in terms of physicochemical properties and water constituents. Table 1 summarizes the pH, alkalinity, DOC, bromide, nitrite, nitrate, and ammonium concentrations of the three secondary effluents. DOM, (bi)­carbonate, nitrite, and bromide are ^•^OH scavengers,^17,69,118^ reducing the efficiency of ^•^OH-induced micropollutant oxidation. Hence, these parameters are relevant for micropollutant abatement during the ozonation of wastewater. The measured ^•^OH exposures (Section 2.5.2) at WWTPs Altenrhein, Neugut, and Werdhoelzli increase with increasing applied specific ozone doses. These ^•^OH exposures are consistent with literature, with values from 1.03 × 10^–11^ Ms to 1.85 × 10^–11^ Ms at a specific ozone dose of 0.1 g_O_3__ g_DOC_^–1^, of 6.80 × 10^–11^ Ms to 1.52 × 10^–10^ Ms at 0.5 g_O_3__ g_DOC_^–1^, and of 1.40 × 10^–10^ Ms to 2 × 10^–10^ Ms at 0.6 g_O_3__ g_DOC_^–1^ for WWTPs with similar pH, DOC, (bi)­carbonate, nitrite, and bromide concentrations.^69^ The relative contributions to ^•^OH scavenging by the different scavengers are listed in Table 1 and more details are provided in SI-B7.

**Table 1:** Water Quality Parameters Characterizing the Secondary Effluents of WWTPs Altenrhein, Neugut, and Werdhoelzli, as well as the Applied Specific Ozone Doses and the Resulting Measured ^•^OH Exposures^a^

	Altenrhein	Neugut	Werdhoelzli
pH	8.0	8.2	8.2
DOC [mg_C_ L^–1^]	6.6 {80.5}	4.4 {74.4}	6.4 {72.5}
Alkalinity [mmol L^–1^]	4.2 {13.3}	5.3 {23.2}	3.4 {10.0}
Bromide [mg L^–1^]	0.06 {0.3}	<0.05 {0.3}	<0.05 {0.2}
Nitrite [μg_N_ L^–1^] ([μg L^–1^])	23.9 (78.4) {5.9}	6.1 (20.1) {2.1}	75.0 (246.3) {17.3}
Nitrate [mg_N_ L^–1^] ([mg L^–1^])	41.7 (184.5)	7.3 (32.2)	5.0 (22.2)
Ammonium [μg_N_ L^–1^] ([μg L^–1^])	40.3 (51.8)	41.7 (53.7)	202.9 (261.2)
Specific ozone dose [g_O_3__ g_DOC_^–1^]	0.1	0.4	0.6
^•^OH exposure [Ms]	(2.26 ± 0.05) × 10^–11^	(7.76 ± 0.09) × 10^–11^	(2.24 ± 0.02) × 10^–10^
O_3_ exposure [Ms] (estimated)	1.3 × 10^–6^	2.2 × 10^–4^	2.8 × 10^–4^

a The relative contributions to ^•^OH scavenging by the different scavengers are provided in braces {%}.

O_3_ exposures were derived from literature values,^85,86^ because for all WWTPs investigated, the low specific ozone doses and the associated rapid ozone decrease, the classical indigo method is not applicable^119^ and quench–flow experiments would be necessary.^22^ For optimal estimation, ozone exposures from Swiss WWTPs with similar specific ozone doses, pH values, alkalinities, nitrite, and DOC concentrations were chosen. For WWTP Neugut, an ozone exposure of 2.2 × 10^–4^ Ms was chosen, which was determined for the secondary effluent of a Swiss WWTP at a specific ozone dose of 0.44 g_O_3__ g_DOC_^–1^, a DOC of 4.5 mg_C_ L^–1^, and an alkalinity of 3.6 mmol L^–1^.^85^ The ozone exposure for WWTP Werdhoelzli was approximated from a study conducted with secondary effluent of WWTP Neugut, which exhibited a similar specific ozone dose (0.58 g_O_3__ g_DOC_^–1^), DOC (6.2 mg_C_ L^–1^), and an alkalinity of 6.3 mmol L^–1^, leading to 2.8 × 10^–4^ Ms.^86^ For the low specific ozone dose of WWTP Altenrhein, no literature value was available. Therefore, its ozone exposure was estimated based on a linear correlation between the natural logarithm of the specific ozone dose and the natural logarithm of the ozone exposure.^86^ This results in a value of 1.3 × 10^–6^ Ms.

To account for the substantial uncertainties associated with the determination of O_3_ and ^•^OH exposures, the Monte Carlo simulation parameters were adjusted accordingly. For O_3_ exposures, a uniform distribution of ±80% around the values derived from literature were applied. ^•^OH exposures were measured in triplicate, allowing calculation of mean values and standard deviations; a normal distribution was assumed for these values. To reflect potential variations in the water matrix between the 2022 and 2024 samples, the standard deviations were multiplied by a factor of 10. These adjustments explain the wider error bars in the modeled abatements (Figure 4) compared with the measured abatements.

**Figure 4:**
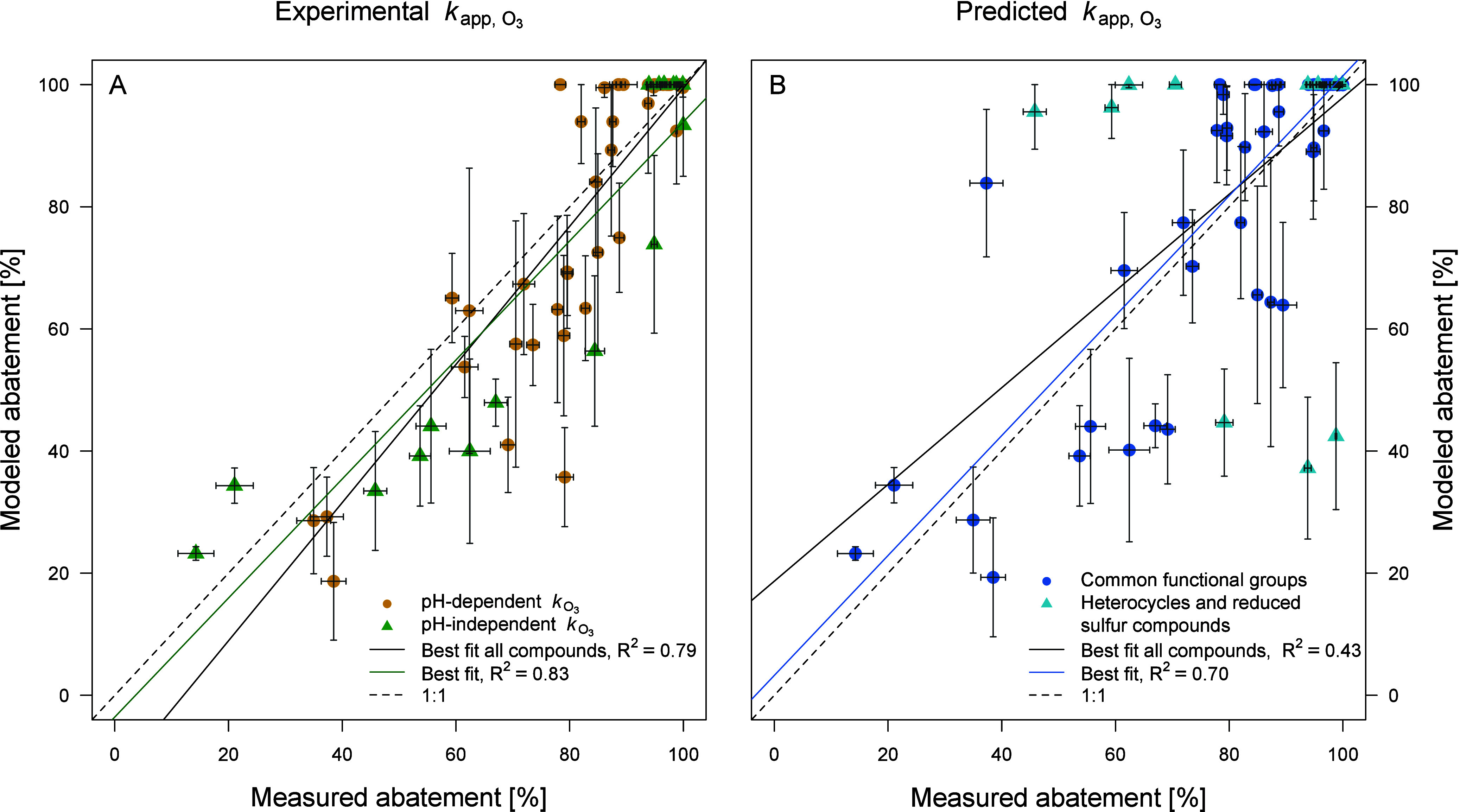
Comparison of measured and modeled relative abatements of selected micropollutants during ozonation of the secondary effluent from WWTP Neugut. Modeled abatements of target compounds during ozonation are presented based on (A) experimentally determined *k*_O_3__ and (B) predicted *k*_O_3__. The specific ozone dose was 0.4 g_O_3__ g_DOC_^–1^ and the ozone exposure was set to 2.2 × 10^–4^ Ms. The error bars of the model were determined with Monte Carlo sampling, while the error bars from the WWTP (measured abatement) correspond to standard deviations from triplicate analyses and the five consecutive sampling days. Compounds with common functional groups refer to anilines, amines, benzenes, olefins, and phenol­(ate)­s.

### 3.4 Measured versus Modeled Abatement in Full-Scale Ozonation

The abatement of pharmaceuticals and their human metabolites over different treatment stages were determined in previous sampling campaigns of the three WWTPs.^36^ The mean abatements for parent compounds, phase I metabolites, and phase II metabolites over the ozonation step were 33%, 28%, and 29% at WWTP Altenrhein; 69%, 49%, and 46% at WWTP Neugut; and 75%, 55%, and 64% at WWTP Werdhoelzli, respectively. This increase in removal efficiency corresponds to increasing specific ozone doses of 0.1 g_O_3__ g_DOC_^–1^, 0.4 g_O_3__ g_DOC_^–1^, and 0.6 g_O_3__ g_DOC_^–1^, respectively.

The abatement of the studied parent compounds during ozonation of wastewater was modeled based on eq 4 combined with a Monte Carlo simulation. Since transformation products identical to human phase I metabolites can form during ozonation of wastewater and since their formation rates are not known, phase I metabolites were not considered. While phase II metabolites do not face this issue, they are largely cleaved during biological wastewater treatment, resulting only in low concentrations reaching the ozonation step. Consequently, the number of phase II metabolites detected in the three WWTPs prior to ozonation is small. Given this low prevalence, their inclusion would be neither of high practical relevance nor representative, which is why only parent compounds were considered in the modeling. Nonetheless, *k*_app,O_3__ values of phase I and phase II metabolites were determined in this study and discussed in detail in Section 3.1.2. Figure 4 shows the measured and modeled abatements of target compounds in wastewater samples during ozonation for WWTP Neugut using experimental *k*_app,O_3__ values (A) and predicted *k*_app,O_3__ values (B). The comparison of the two graphs reveals a significant difference in model variance, as evidenced by the *R* ^2^ values of 0.79 and 0.43, respectively. However, when categorizing the compounds in Figure 4B based on their reactive functional groups, a distinction can be made between common functional groups for which QSAR models are available (such as anilines, amines, benzenes, olefins, and phenol­(ate)­s) and those for which only a few literature values exist, and hence, no QSAR has been established yet (such as heterocycles and reduced sulfur compounds). Considering only compounds with common functional groups (anilines, amines, benzenes, olefins, and phenol­(ate)­s), an increase in *R* ^2^ from 0.43 to 0.70 is observed. In this study, we also determined *k*_app,O_3__ values for 21 compounds containing heterocycles and 3 containing reduced sulfur moieties. This work, together with other^87,113,120^ studies contributes to a more comprehensive understanding of the abatement of such compound classes during wastewater ozonation.

The use of experimental *k*_app,O_3__ values results in an improved model performance, with a slope of 1.12 and an intercept of −11.6%. It is important to note that the currently used *k*_app,O_3__ values were determined at pH 7, whereas the wastewaters have a pH ∼ 8. Exclusion of compounds with pH-dependent second-order rate constants leads to an even better model performance, with a slope of 0.99, an intercept of −3.9%, and an *R* ^2^ of 0.83. Compounds with pH-sensitive reactive moieties such as amines and phenols are expected to react about a factor of 10 faster at the higher pH due to the deprotonation and the associated increased electron density.^17^ The compounds with pH-sensitive reactive moieties are highlighted in Figure 4A (brown symbols). Moreover, the targeted specific ozone dose in WWTP depends on the precision and frequency of the DOC measurements. Hence, short-term fluctuations of the DOC concentration may have an additional influence on the specific ozone dose and, subsequently, compound abatement. Another important factor to consider is temperature, which can significantly influence micropollutant abatement during ozonation. The *k*_app,O_3__ are temperature-dependent and typically increase with increasing temperature, whereas the kOH• values are much less affected, as they are largely diffusion-controlled. The temperature dependence of *k*_app,O_3__ can be implemented by using an Arrhenius-type relationship. However, the activation energies vary substantially between compounds and are often not known, making general application challenging.^106^ Figures SI-B91 and SI-B92 visualize the analogous model results for WWTPs Altenrhein and Werdhoelzli, respectively. Similarly, better model results were obtained with experimental compared with predicted *k*_app,O_3__ values.

The modeled abatements at pH 7 show considerable uncertainties, as visualized by the error bars in Figure 4. Nevertheless, modeling allows for the identification of the most critical parameters relevant for micropollutant abatement during wastewater ozonation using a sensitivity analysis. Figure 5A shows the abatement based on eq 4 as a function of *k*_O_3__ for fixed ^•^OH and O_3_ exposures provided in Table 1. For kOH• , three different values covering the range of predicted kOH• (SI-A5) were considered. For compounds with *k*_O_3__ values >1 × 10^4^ M^–1^ s^–1^ (WWTPs Neugut and Werdhoelzli) and >1 × 10^7^ M^–1^ s^–1^ (WWTP Altenrhein), the abatement reaches 100% and is independent of the *k*_O_3__. In these reactivity ranges, compounds are efficiently degraded by O_3_ and their abatement is accurately predicted. Next to the effect of *k*_O_3__, Figure 5A further clarifies the effect of ^•^OH on the removal of pharmaceuticals during the ozonation of wastewater. An increase in the kOH• value from 1 × 10^9^ M^–1^ s^–1^ to 5 × 10^9^ M^–1^ s^–1^ leads to an increase in removal from 10% to 45% for compounds with low reactivity toward ozone (*k*_O_3__ = 1 × 10^–1^ M^–1^ s^–1^ to 1 × 10^2^ M^–1^ s^–1^), depending on the ^•^OH exposure and hence the applied specific ozone dose of the WWTP. Moving toward compounds with higher reactivity with O_3_, the effect of increasing kOH• values vanishes, since the abatement is driven by the fast reaction with ozone and the higher O_3_ exposure compared to the significantly lower ^•^OH exposure.

**Figure 5:**
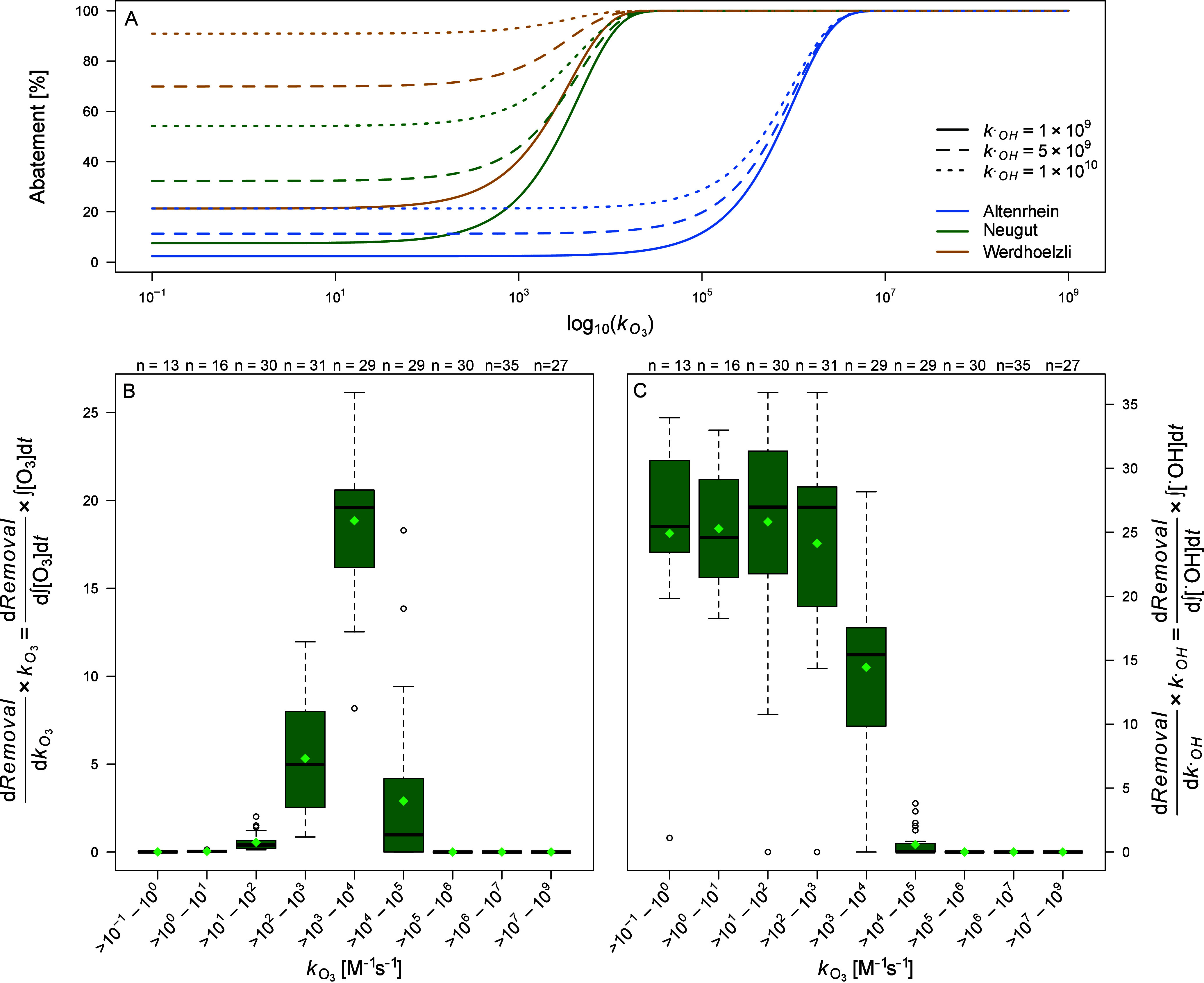
Influence of *k*_O_3__, kOH• , O_3_ exposure, and ^•^OH exposure on abatement of micropollutants during wastewater ozonation. (A) Abatement during ozonation as a function of *k*_O_3__ for the three WWTPs Altenrhein, Neugut, and Werdhoelzli, assuming their ^•^OH and O_3_ exposures as constant based on the laboratory experiments and literature values, respectively. Three different kOH• values in the predicted range were considered. (B) Derivative-based local sensitivity analysis with respect to *k*_O_3__ and O_3_ exposure and (C) with respect to kOH• and ^•^OH exposure for WWTP Neugut. Light green diamonds indicate the mean values.

For the derivative-based sensitivity analysis, the removal function was derived with respect to the four parameters *k*_O_3__, kOH• , O_3_ exposure, and ^•^OH exposure, and further normalized to obtain the sensitivity indices for all selected compounds. Due to this normalization, the relative effects of the four parameters on the removal can be directly compared, and the compounds are grouped according to their reactivity toward ozone (*k*_app,O_3__). Since the derivative corresponds to the slope of the removal function (Figure 5A), the highest sensitivity indices for *k*_O_3__ and O_3_ exposure (Figure 5B) are observed in the range where the removal curve is steepest (1 × 10^2^ M^–1^ s^–1^ to 1 × 10^4^ M^–1^ s^–1^). The sensitivity indices represent how changes in the four key parameters influence abatement and the corresponding impact can be directly observed on the *y*-axis of Figures 5B/C. For example, for a compound with a second-order rate constant *k*_O_3__ between 1 × 10^2^ and 1 × 10^3^ M^–1^ s^–1^, a 1% increase in either *k*_O_3__ or O_3_ exposure leads to an approximately 5% change in abatement, as indicated by the mean sensitivity values in Figure 5B. In contrast, a 1% increase in kOH• or ^•^OH exposure results in a much larger change in removal of around 25%, which can be derived from the *y*-axis of Figure 5C. Equivalent analyses for WWTPs Altenrhein and Werdhoelzli are shown in Figures SI-B94/95.

The derivative-based sensitivity analysis in Figure 5B/C confirms the conclusion derived from Figure 5A, namely, the low impact of *k*_O_3__ and kOH• variability on the predicted removal for compounds with high *k*_O_3__, as the abatement reaches 100% and is independent of *k*_O_3__. Conversely, for *k*_O_3__ values <1 × 10^3^ M^–1^ s^–1^ (WWTPs Neugut and Werdhoelzli) and <1 × 10^4^ M^–1^ s^–1^ (WWTP Altenrhein), ozone barely reacts with the selected compounds and thus the abatement does not strongly depend on *k*_O_3__ (Figure 5B), but becomes highly contingent on kOH• (Figure 5C). This leads to a high dependency of the predicted abatements on the precision of kOH• . For intermediate *k*_O_3__ values, i.e., 1 × 10^3^ M^–1^ s^–1^ to 1 × 10^4^ M^–1^ s^–1^ for WWTPs Neugut and Werdhoelzli, and from 1 × 10^4^ M^–1^ s^–1^ to 1 × 10^7^ M^–1^ s^–1^ for WWTP Altenrhein, abatement is contingent on both, the reaction with O_3_ and ^•^OH. Compounds with *k*_O_3__ in the range from 1 × 10^–1^ M^–1^ s^–1^to 1 × 10^3^ M^–1^ s^–1^ exhibit mean sensitivity indices with respect to kOH• and ^•^OH exposure around 25%. These values exceed the average sensitivity indices observed for intermediately reactive compounds with respect to *k*_O_3__ and O_3_ exposure (<20%). Therefore, an increasing ^•^OH exposure has a stronger impact on the abatement of barely ozone-reactive compounds compared to the effect of an increasing O_3_ exposure on the removal of intermediately ozone-reactive compounds. This means that abatement relies heavily on ^•^OH, making the extent of their degradation directly proportional to ^•^OH exposure.^121^ In general, ^•^OH exposure is reduced by ^•^OH scavengers such as DOM, (bi)­carbonate, bromide, and nitrite, with their concentrations playing a critical role in the extent of scavenging. In the investigated wastewaters, ^•^OH scavenging is dominated by DOM, followed by (bi)­carbonate and nitrite, each contributing to a similar extent, but to a lower relative extent than DOM; bromide is negligible in the concentration range of this study (Table 1). This effect is even more important for ozone, since both DOM and nitrite efficiently consume ozone. Therefore, a well-functioning biological wastewater treatment, including nitrification-denitrification, is crucial to minimize DOM and nitrite levels and maintain high ^•^OH and O_3_ exposures for effective micropollutant abatement.

## 4 Practical Implications

This study demonstrates the effective applicability of multicompound competition kinetics for the determination of apparent second-order rate constants *k*_app,O_3__ of reactions of ozone with pharmaceuticals and human metabolites. While the applied approach offers significant advantages for a good estimation of the reaction kinetics, conventional methods targeting individual compounds are preferable when high precision in determining *k*_app,O_3__ is required. However, toward the aim of deepening the understanding of the reactivity of pharmaceuticals compared to their human metabolites during ozonation, multicompound competition kinetics is a valuable tool.

Besides the determination of *k*_app,O_3__ values, kOH• values are crucial for understanding the abatement behavior of pharmaceuticals and their human metabolites during ozonation of wastewater. A multicompound competition kinetics approach could also help to understand the reactivity of target compounds toward hydroxyl radicals, allowing for a quick determination of kOH• values. As the sensitivity analysis has shown, the kOH• values, as well as the ^•^OH exposure, are critical factors for the abatement of compounds exhibiting low *k*_app,O_3__ (<1 × 10^2^ M^–1^ s^–1^). It is thus important not only to reliably determine second-order rate constants for the reactions with O_3_ and ^•^OH, but also to measure their exposures, ideally in the same samples used for micropollutant analysis. For compounds with intermediate reactivities toward ozone (1 × 10^2^ M^–1^ s^–1^ < *k*_app,O_3__ < 1 × 10^4^ M^–1^ s^–1^), with the exact range depending on the applied specific ozone dose, the sensitivity analysis identified *k*_app,O_3__ and O_3_ exposure to be the only parameters needed to accurately evaluate their abatement.

Based on analysis of parent pharmaceutical compounds and their human metabolites, a flowchart was developed to estimate the relative reactivities of two structurally similar compounds (Figure 6). This chart can be used for an initial assessment of newly identified metabolites and transformation products during ozonation. While precise absolute predictions of second-order rate constants with ozone may be challenging for complex molecules, a relative assessment of two structurally related compounds, as for parent and human metabolites, is feasible and relatively straightforward. One example is efavirenz, an HIV medication not included in this study. The parent compound has two ozone-reactive sites: an alkyne (∼10^2^ M^–1^ s^–1^) and a deactivated benzene ring (∼1 M^–1^ s^–1^), with the alkyne expected to be more reactive. From human metabolism studies it is known that the benzene ring can be hydroxylated at two positions, leading to the formation of phenol-type structures.^122^ According to the flowchart, these aromatic hydroxylations yield metabolites that react faster with ozone than the parent compound. Another example is sertraline, an antidepressant containing two benzene rings (∼10^2^ M^–1^ s^–1^) and a secondary amine (∼10^3^ M^–1^ s^–1^), with the amine expected to be the most reactive site at pH 7. Its known human metabolite, *N*-desmethylsertraline,^123^ features a primary amine. Since secondary amines are generally more reactive than primary amines according to the flowchart, this metabolite is expected to be less reactive than sertraline during ozonation.

**Figure 6:**
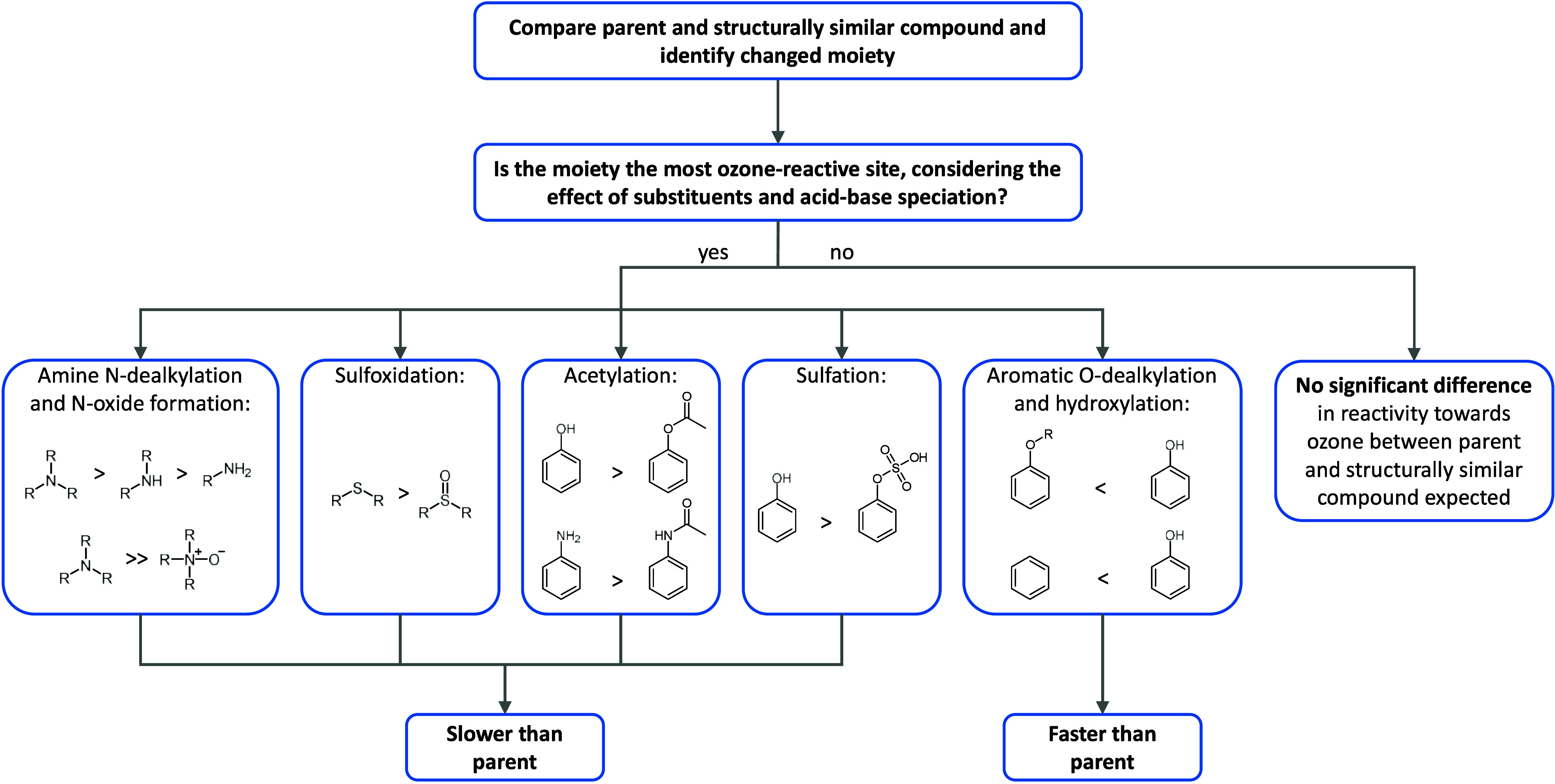
Flowchart for relative ozone reactivity determination of structurally similar compounds. The graph summarizes the obtained results from the functional group change analysis between the parents and metabolites.

Human pharmaceutical metabolites represent a significant portion of the total pharmaceutical load entering WWTPs (up to 50%).^36^ However, despite notable differences in the abatement efficiencies of directly related parent pharmaceutical compounds and their human metabolites, average abatements during wastewater ozonation over all studied compounds are generally comparable, as demonstrated in our previous publication.^36^ Based on these findings, optimization of ozonation conditions in full-scale wastewater treatment plants is not necessary, as current treatment practices are sufficient to effectively abate both parent compounds and their metabolites. Overall, these results indicate that current treatment strategies and regulatory indicators provide a suitable basis for addressing pharmaceuticals as well as their metabolites in wastewater, although continued evaluation remains important.

## Supplementary Material







## Data Availability

In this study, RStudio was used for data visualization and figure creation. The code for the ozone model, including Monte Carlo sampling is provided on GitLab (https://gitlab.com/Corina_Meyer/ozone-model/-/tree/main).

## Notes

Compound information; *k*_O_3__ prediction; experimentally determined *k*_O_3__ values; available literature *k*_O_3__ values for parent pharmaceuticals compounds; kOH• prediction (QSPR, pySiRC); concentrations of pharmaceuticals and their metabolites in wastewater; and data on WWTPs (XLSX)
Chemicals and solvents; experimental details to ozonation experiments; details about kOH• prediction tools; information about sampled WWTPs; linear correlations for *k*_O_3__ derivations with multicompound competition kinetics; comparison of literature and experimental *k*_O_3__ values and discussion of outliers; comparison of abatement behavior between parents and metabolites; model results; and sensitivity analysis (PDF)
kOH• prediction (GCM) (XLSX)
