# Supplementary material for: Ozonation of Pharmaceuticals and Their Human Metabolites in Wastewater—Insights from Laboratory Experiments and Field Data
Source: Environ Sci Technol. 2025 Sep 22;59(38):20842–59. doi: 10.1021/acs.est.5c08128 (PMC12490022; doi:10.1021/acs.est.5c08128)
Supplement: Supplementary file 2 [file es5c08128_si_002.pdf]

Supporting Information B to:

Ozonation of Pharmaceuticals and Their Human Metabolites in Wastewater - Insights from Laboratory Experiments and Field Data

Corina Meyer[†,‡], Pia M. Kronsbein[†], Valentin Rougé[†], Urs von Gunten[†, §], Christa S. McArdell[†], Juliane Hollender[*,†,‡]

[†] Eawag: Swiss Federal Institute of Aquatic Science and Technology, Ueberlandstrasse 133, 8600, Duebendorf, Switzerland
[‡] Institute of Biogeochemistry and Pollutant Dynamics, Universitaetstrasse 16, ETH Zurich, 8092, Zurich, Switzerland
[§] School of Architecture, Civil and Environmental Engineering (ENAC), Ecole Polytechnique Federale de Lausanne (EPFL), 1015, Lausanne, Switzerland

Environmental Science & Technology, 2025

Supporting Pages: S2 - S159
Supporting Figures: SI-B1 - SI-B95
Supporting Tables: SI-B1 - SI-B9

Contents

SI-B1 Materials & Methods S20
SI-B1.1 Chemicals and Solvents S20
SI-B1.2 Ozonation Experiments S21
SI-B1.2.1 Competitor Compounds S21
SI-B1.2.2 HPLC-HRMS/MS S23
SI-B1.2.3 Secondary effluent characterization S23
SI-B1.2.4 $^{\bullet}$OH exposure S24
SI-B1.3 $k_{^{\bullet}\mathrm{OH}}$ prediction S25
SI-B1.3.1 Group contribution method S25
SI-B1.3.2 Quantitative structure property relationship S30
SI-B1.4 Machine learning model - pySiRC S30
SI-B1.5 Wastewater Treatment Plants S31
SI-B1.6 Sensitivity analysis S32
SI-B2 $k_{\mathrm{O_3}}$ value derivation using multi-compound competition kinetics S33
SI-B3 Comparing Literature and Experimental $k_{\mathrm{app,O_3}}$ S129
SI-B4 Measured vs Predicted $k_{\mathrm{app,O_3}}$: Outliers S134
SI-B4.1 4'-Hydroxydiclofenac S135
SI-B4.2 Cortisone-like structures S136
SI-B4.3 Nevirapine and 12-hydroxynevirapine S136
SI-B4.4 Benzoylecgonine and norketamine S137
SI-B4.5 D617 and lidocaine-*N*-oxide S137
SI-B4.6 N^4-Acetylsulfadiazine, N^4-acetylsulfapyridine and N^4-acetylsulfathiazole S138
SI-B4.7 Telmisartan, telmisartan-*O*-acyl-glucuronide and pantoprazole S139
SI-B4.8 3,5-Diamino-2,4,6-triiodobenzoic acid S140
SI-B4.9 Iminostilbene S140
SI-B4.10 Sulpiride-*N*-oxide S141
SI-B4.11 Chlorothiazide S141
SI-B4.12 Amlodipine S142
SI-B4.13 1,7-Dimethyluric acid S142
SI-B5 Parent vs metabolites S143
SI-B5.1 Hydroxylated vs non-hydroxylated S143
SI-B5.2 *N*-Oxidized vs non-*N*-oxidized S144
SI-B5.3 Carboxylated vs non-carboxylated S145
SI-B5.4 Conjugated vs non-conjugated S146
SI-B5.5 Dealkylated vs non-dealkylated S148
SI-B6 $k_{^{\bullet}\mathrm{OH}}$ Prediction S150
SI-B7 Relative Contribution of $^{\bullet}$OH Scavengers S151
SI-B8 Measured vs Predicted Abatement S152
SI-B9 Sensitivity analysis S154

List of Figures

SI-B1 Three of the four key reaction mechanisms included in group contribution method (GCM) are possible for atenolol: H-atom abstraction (yellow), hydroxyl radical addition to aromatic rings (blue) and reaction with nitrogen-containing moieties (green). S25

SI-B2 Excerpt of the Excel sheet to predict $k_{^\bullet OH}$ values by group contribution method (GCM), considering the contribution of H-atom abstraction. S26

SI-B3 Excerpt of the Excel sheet to predict $k_{^\bullet OH}$ values by group contribution method (GCM), considering the contribution of hydroxyl radical addition to alkenes. S27

SI-B4 Excerpt of the Excel sheet to predict $k_{^\bullet OH}$ values by group contribution method (GCM), considering the contribution of hydroxyl radical addition to aromatic rings. S28

SI-B5 Excerpt of the Excel sheet to predict $k_{^\bullet OH}$ values by group contribution method (GCM), considering the contribution of reactions with sulfur, nitrogen or phosphorus-containing moieties. S29

SI-B6 Flow scheme of the wastewater treatment plant (WWTP) Altenrhein (above), Neugut and Werdhoelzli (below), including sampling locations. At wastewater treatment plant (WWTP) Altenrhein, conventional activated sludge treatment (2/3 of wastewater volume) and the fixed-bed reactor (1/3 of wastewater volume) are operated in parallel. The granular activated carbon (GAC) filter was sampled after a run time of 22 700 bed volumes. The circles refer to 24 h flow proportional composite samples, while the diamonds indicate 24 h time-proportional sampling. S31

SI-B7 Correlations between the natural logarithm of the relative residual peak areas of acemethacin (a - c) and indomethacin (d - e) with competitors upon ozonation at pH 7 (2 mM phosphate), 22 °C, and in presence of *t*BuOH (40 mM). The intercept was considered negligible (<10×slope) and the standard deviations of competitor k_{app,O_3} values were considered in the calculation of the standard deviations of the target k_{app,O_3} values. Note that the axes do not necessarily originate at zero. S33

SI-B8 Correlations between the natural logarithm of the relative residual peak areas of allopurinol (a) and oxypurinol (b - c) with competitors upon ozonation at pH 7 (2 mM phosphate), 22 °C, and in presence of *t*BuOH (40 mM). The intercept was considered negligible (<10×slope) and the standard deviations of competitor k_{app,O_3} values were considered in the calculation of the standard deviations of the target k_{app,O_3} values. Note that the axes do not necessarily originate at zero. S34

SI-B9 Correlations between the natural logarithm of the relative residual peak areas of amisulpride (a - b) and amisulpride-N-oxide (c) with competitors upon ozonation at pH 7 (2 mM phosphate), 22 °C, and in presence of tBuOH (40 mM). The intercept was considered negligible (<10×slope) and the standard deviations of competitor $k_{\mathrm{app,O_3}}$ values were considered in the calculation of the standard deviations of the target $k_{\mathrm{app,O_3}}$ values. Note that the axes do not necessarily originate at zero. S35
SI-B10 Correlations between the natural logarithm of the relative residual peak areas of amlodipine (a), O-des(2-aminoethyl)-O-carboxymethyl-dehydroamlodipine (b) and dehydroamlodipine (c - d) with competitors upon ozonation at pH 7 (2 mM phosphate), 22 °C, and in presence of tBuOH (40 mM). The intercept was considered negligible (<10×slope) and the standard deviations of competitor $k_{\mathrm{app,O_3}}$ values were considered in the calculation of the standard deviations of the target $k_{\mathrm{app,O_3}}$ values. Note that the axes do not necessarily originate at zero. S36
SI-B11 Correlations between the natural logarithm of the relative residual peak areas of amoxicillin (a - c), amoxicillin impurity E (d) and amoxicillin impurity F (e - f) with competitors upon ozonation at pH 7 (2 mM phosphate), 22 °C, and in presence of tBuOH (40 mM). The intercept was considered negligible (<10×slope) and the standard deviations of competitor $k_{\mathrm{app,O_3}}$ values were considered in the calculation of the standard deviations of the target $k_{\mathrm{app,O_3}}$ values. Note that the axes do not necessarily originate at zero. S37
SI-B12 Correlations between the natural logarithm of the relative residual peak areas of atenolol (a - b), atenolol acid (c - d) and atenolol-desisopropyl (e - f) with competitors upon ozonation at pH 7 (2 mM phosphate), 22 °C, and in presence of tBuOH (40 mM). The intercept was considered negligible (<10×slope) and the standard deviations of competitor $k_{\mathrm{app,O_3}}$ values were considered in the calculation of the standard deviations of the target $k_{\mathrm{app,O_3}}$ values. Note that the axes do not necessarily originate at zero. S38
SI-B13 Correlations between the natural logarithm of the relative residual peak areas of azithromycin (a), desosaminylazithromycin (b), N-desmethylazithromycin (c - d) and azithromycin-N-oxide (e - f) with competitors upon ozonation at pH 7 (2 mM phosphate), 22 °C, and in presence of tBuOH (40 mM). The intercept was considered negligible (<10×slope) and the standard deviations of competitor $k_{\mathrm{app,O_3}}$ values were considered in the calculation of the standard deviations of the target $k_{\mathrm{app,O_3}}$ values. Note that the axes do not necessarily originate at zero. S39
SI-B14 Correlations between the natural logarithm of the relative residual peak areas of betamethasone (a - b) and betamethasone-21-acetate (c) with competitors upon ozonation at pH 7 (2 mM phosphate), 22 °C, and in presence of tBuOH (40 mM). The intercept was considered negligible (<10×slope) and the standard deviations of competitor $k_{\mathrm{app,O_3}}$ values were considered in the calculation of the standard deviations of the target $k_{\mathrm{app,O_3}}$ values. Note that the axes do not necessarily originate at zero. S40
SI-B14 Correlations between the natural logarithm of the relative residual peak areas of bupropion (a - c), dihydrobupropion (d - e) and hydroxybupropion (f - g) with competitors upon ozonation at pH 7 (2 mM phosphate), 22 °C, and in presence of tBuOH (40 mM). The intercept was considered negligible (<10×slope) and the standard deviations of competitor $k_{\mathrm{app,O_3}}$ values were considered in the calculation of the standard deviations of the target $k_{\mathrm{app,O_3}}$ values. Note that the axes do not necessarily originate at zero. S42

SI-B15 Correlations between the natural logarithm of the relative residual peak areas of emtricitabine (a - b) and 5-fluorocytosine (c - e) with competitors upon ozonation at pH 7 (2 mM phosphate), 22 °C, and in presence of *t*BuOH (40 mM). The intercept was considered negligible (<10×slope) and the standard deviations of competitor k_{app,O_3} values were considered in the calculation of the standard deviations of the target k_{app,O_3} values. Note that the axes do not necessarily originate at zero. S43
SI-B16 Correlations between the natural logarithm of the relative residual peak areas of iminostilbene (a) and licarbazepine (b) with competitors upon ozonation at pH 7 (2 mM phosphate), 22 °C, and in presence of *t*BuOH (40 mM). The intercept was considered negligible (<10×slope) and the standard deviations of competitor k_{app,O_3} values were considered in the calculation of the standard deviations of the target k_{app,O_3} values. Note that the axes do not necessarily originate at zero. S44
SI-B16 Correlations between the natural logarithm of the relative residual peak areas of cetirizine (a - b), 4-chlorobenzophenone (c), 1-(4-chlorobenzhydryl)piperazine (d - e) and cetirizine-*N*-oxide (f - g) with competitors upon ozonation at pH 7 (2 mM phosphate), 22 °C, and in presence of *t*BuOH (40 mM). The intercept was considered negligible (<10×slope) and the standard deviations of competitor k_{app,O_3} values were considered in the calculation of the standard deviations of the target k_{app,O_3} values. Note that the axes do not necessarily originate at zero. S46
SI-B17 Correlations between the natural logarithm of the relative residual peak areas of citalopram (a - b), *N*-desmethylcitalopram (c - d) and *N*-didesmethylcitalopram (e) with competitors upon ozonation at pH 7 (2 mM phosphate), 22 °C, and in presence of *t*BuOH (40 mM). The intercept was considered negligible (<10×slope) and the standard deviations of competitor k_{app,O_3} values were considered in the calculation of the standard deviations of the target k_{app,O_3} values. Note that the axes do not necessarily originate at zero. S47
SI-B18 Correlations between the natural logarithm of the relative residual peak areas of clarithromycin (a) and *N*-desmethylclarithromycin (b - c) with competitors upon ozonation at pH 7 (2 mM phosphate), 22 °C, and in presence of *t*BuOH (40 mM). The intercept was considered negligible (<10×slope) and the standard deviations of competitor k_{app,O_3} values were considered in the calculation of the standard deviations of the target k_{app,O_3} values. Note that the axes do not necessarily originate at zero. S48
SI-B19 Correlations between the natural logarithm of the relative residual peak areas of clopidogrel (a - c) and clopidogrel carboxylic acid (d - f) with competitors upon ozonation at pH 7 (2 mM phosphate), 22 °C, and in presence of *t*BuOH (40 mM). The intercept was considered negligible (<10×slope) and the standard deviations of competitor k_{app,O_3} values were considered in the calculation of the standard deviations of the target k_{app,O_3} values. Note that the axes do not necessarily originate at zero. S49
SI-B19 Correlations between the natural logarithm of the relative residual peak areas of morphine (a), 6-acetylmorphine (b), codeine (c - e) and norcodeine (f - g) with competitors upon ozonation at pH 7 (2 mM phosphate), 22 °C, and in presence of *t*BuOH (40 mM). The intercept was considered negligible (<10×slope) and the standard deviations of competitor k_{app,O_3} values were considered in the calculation of the standard deviations of the target k_{app,O_3} values. Note that the axes do not necessarily originate at zero. S51

SI-B20 Correlations between the natural logarithm of the relative residual peak areas of deprenyl (a - c), deprenyl-N-oxide (d) and nordeprenyl (e - f) with competitors upon ozonation at pH 7 (2 mM phosphate), 22 °C, and in presence of tBuOH (40 mM). The intercept was considered negligible (<10×slope) and the standard deviations of competitor k_{app,O_3} values were considered in the calculation of the standard deviations of the target k_{app,O_3} values. Note that the axes do not necessarily originate at zero. S52
SI-B21 Correlations between the natural logarithm of the relative residual peak areas of dextromethorphan (a - b) and dextrorphan (c - e) with competitors upon ozonation at pH 7 (2 mM phosphate), 22 °C, and in presence of tBuOH (40 mM). The intercept was considered negligible (<10×slope) and the standard deviations of competitor k_{app,O_3} values were considered in the calculation of the standard deviations of the target k_{app,O_3} values. Note that the axes do not necessarily originate at zero. S53
SI-B22 Correlations between the natural logarithm of the relative residual peak areas of diatrizoate (a), 3,5-siamino-2,4,6-triiodobenzoic acid (b) and 3,5-diiodo-L-tyrosine (c) with competitors upon ozonation at pH 7 (2 mM phosphate), 22 °C, and in presence of tBuOH (40 mM). The intercept was considered negligible (<10×slope) and the standard deviations of competitor k_{app,O_3} values were considered in the calculation of the standard deviations of the target k_{app,O_3} values. Note that the axes do not necessarily originate at zero. S54
SI-B22 Correlations between the natural logarithm of the relative residual peak areas of diclofenac (a - c), 5-hydroxydiclofenac (d), 4'-hydroxydiclofenac (e - f) and diclofenac carboxylic acid (g -h) with competitors upon ozonation at pH 7 (2 mM phosphate), 22 °C, and in presence of tBuOH (40 mM). The intercept was considered negligible (<10×slope) and the standard deviations of competitor k_{app,O_3} values were considered in the calculation of the standard deviations of the target k_{app,O_3} values. Note that the axes do not necessarily originate at zero. S56
SI-B23 Correlations between the natural logarithm of the relative residual peak areas of diltiazem (a - b) and desacetyldiltiazem (c - d) with competitors upon ozonation at pH 7 (2 mM phosphate), 22 °C, and in presence of tBuOH (40 mM). The intercept was considered negligible (<10×slope) and the standard deviations of competitor k_{app,O_3} values were considered in the calculation of the standard deviations of the target k_{app,O_3} values. Note that the axes do not necessarily originate at zero. S57
SI-B24 Correlations between the natural logarithm of the relative residual peak areas of fenofibrate (a) and fenofibric acid (b) with competitors upon ozonation at pH 7 (2 mM phosphate), 22 °C, and in presence of tBuOH (40 mM). The intercept was considered negligible (<10×slope) and the standard deviations of competitor k_{app,O_3} values were considered in the calculation of the standard deviations of the target k_{app,O_3} values. Note that the axes do not necessarily originate at zero. S58
SI-B25 Correlations between the natural logarithm of the relative residual peak areas of fexofenadine (a - b) and fexofenadine-N-oxide (c) with competitors upon ozonation at pH 7 (2 mM phosphate), 22 °C, and in presence of tBuOH (40 mM). The intercept was considered negligible (<10×slope) and the standard deviations of competitor k_{app,O_3} values were considered in the calculation of the standard deviations of the target k_{app,O_3} values. Note that the axes do not necessarily originate at zero. S59

SI-B25 Correlations between the natural logarithm of the relative residual peak areas of fluoxetine (a - c), 4-trifluoromethylphenol (d), norfluoxetine (e - f) and fluoxetine formamide (g) with competitors upon ozonation at pH 7 (2 mM phosphate), 22 °C, and in presence of *t*BuOH (40 mM). The intercept was considered negligible (<10×slope) and the standard deviations of competitor k_{app,O_3} values were considered in the calculation of the standard deviations of the target k_{app,O_3} values. Note that the axes do not necessarily originate at zero. S61
SI-B26 Correlations between the natural logarithm of the relative residual peak areas of gemcitabine (a) and 2-deoxy-2,2-difluorouridine (b - c) with competitors upon ozonation at pH 7 (2 mM phosphate), 22 °C, and in presence of *t*BuOH (40 mM). The intercept was considered negligible (<10×slope) and the standard deviations of competitor k_{app,O_3} values were considered in the calculation of the standard deviations of the target k_{app,O_3} values. Note that the axes do not necessarily originate at zero. S62
SI-B27 Correlations between the natural logarithm of the relative residual peak areas of chlorothiazide (a), hydrochlorothiazide (b) and 4-amino-6-chlorobenzene-1,3-disulfonamide (c - d) with competitors upon ozonation at pH 7 (2 mM phosphate), 22 °C, and in presence of *t*BuOH (40 mM). The intercept was considered negligible (<10×slope) and the standard deviations of competitor k_{app,O_3} values were considered in the calculation of the standard deviations of the target k_{app,O_3} values. Note that the axes do not necessarily originate at zero. S63
SI-B28 Correlations between the natural logarithm of the relative residual peak areas of cortisone (a - c) and hydrocortisone (d - e) with competitors upon ozonation at pH 7 (2 mM phosphate), 22 °C, and in presence of *t*BuOH (40 mM). The intercept was considered negligible (<10×slope) and the standard deviations of competitor k_{app,O_3} values were considered in the calculation of the standard deviations of the target k_{app,O_3} values. Note that the axes do not necessarily originate at zero. S64
SI-B28 Correlations between the natural logarithm of the relative residual peak areas of lidocaine (a - c), lidocaine-*N*-oxide (d) and lidocaine-*N*-desethyl (e - g) with competitors upon ozonation at pH 7 (2 mM phosphate), 22 °C, and in presence of *t*BuOH (40 mM). The intercept was considered negligible (<10×slope) and the standard deviations of competitor k_{app,O_3} values were considered in the calculation of the standard deviations of the target k_{app,O_3} values. Note that the axes do not necessarily originate at zero. S66
SI-B29 Correlations between the natural logarithm of the relative residual peak areas of 4-aminoantipyrine (a) and 4-formylaminantipyrine (b) with competitors upon ozonation at pH 7 (2 mM phosphate), 22 °C, and in presence of *t*BuOH (40 mM). The intercept was considered negligible (<10×slope) and the standard deviations of competitor k_{app,O_3} values were considered in the calculation of the standard deviations of the target k_{app,O_3} values. Note that the axes do not necessarily originate at zero. S67
SI-B30 Correlations between the natural logarithm of the relative residual peak areas of methadone (a - b) and EDDP (c) with competitors upon ozonation at pH 7 (2 mM phosphate), 22 °C, and in presence of *t*BuOH (40 mM). The intercept was considered negligible (<10×slope) and the standard deviations of competitor k_{app,O_3} values were considered in the calculation of the standard deviations of the target k_{app,O_3} values. Note that the axes do not necessarily originate at zero. S68

SI-B30 Correlations between the natural logarithm of the relative residual peak areas of amphetamine (a), 4-hydroxyamphetamine (b), ephedrine (c) and norephedrine (d - e) with competitors upon ozonation at pH 7 (2 mM phosphate), 22 °C, and in presence of *t*BuOH (40 mM). The intercept was considered negligible (<10×slope) and the standard deviations of competitor $k_{\mathrm{app,O_3}}$ values were considered in the calculation of the standard deviations of the target $k_{\mathrm{app,O_3}}$ values. Note that the axes do not necessarily originate at zero. S70
SI-B31 Correlations between the natural logarithm of the relative residual peak areas of methylphenidate (a - b) and ritalinic acid (c - d) with competitors upon ozonation at pH 7 (2 mM phosphate), 22 °C, and in presence of *t*BuOH (40 mM). The intercept was considered negligible (<10×slope) and the standard deviations of competitor $k_{\mathrm{app,O_3}}$ values were considered in the calculation of the standard deviations of the target $k_{\mathrm{app,O_3}}$ values. Note that the axes do not necessarily originate at zero. S71
SI-B31 Correlations between the natural logarithm of the relative residual peak areas of mianserin (a - c), normianserin (d - f) and mianserin-*N*-oxide (g - h) with competitors upon ozonation at pH 7 (2 mM phosphate), 22 °C, and in presence of *t*BuOH (40 mM). The intercept was considered negligible (<10×slope) and the standard deviations of competitor $k_{\mathrm{app,O_3}}$ values were considered in the calculation of the standard deviations of the target $k_{\mathrm{app,O_3}}$ values. Note that the axes do not necessarily originate at zero. S73
SI-B32 Correlations between the natural logarithm of the relative residual peak areas of mycophenolic acid (a), and 6-*O*-desmethylmycophenolic acid (b) with competitors upon ozonation at pH 7 (2 mM phosphate), 22 °C, and in presence of *t*BuOH (40 mM). The intercept was considered negligible (<10×slope) and the standard deviations of competitor $k_{\mathrm{app,O_3}}$ values were considered in the calculation of the standard deviations of the target $k_{\mathrm{app,O_3}}$ values. Note that the axes do not necessarily originate at zero. S73
SI-B33 Correlations between the natural logarithm of the relative residual peak areas of nicotine (a - b), nornicotine (c - d) and cotinine (e) with competitors upon ozonation at pH 7 (2 mM phosphate), 22 °C, and in presence of *t*BuOH (40 mM). The intercept was considered negligible (<10×slope) and the standard deviations of competitor $k_{\mathrm{app,O_3}}$ values were considered in the calculation of the standard deviations of the target $k_{\mathrm{app,O_3}}$ values. Note that the axes do not necessarily originate at zero. S74
SI-B34 Correlations between the natural logarithm of the relative residual peak areas of oseltamivir (a - b) and oseltamivir acid (c) with competitors upon ozonation at pH 7 (2 mM phosphate), 22 °C, and in presence of *t*BuOH (40 mM). The intercept was considered negligible (<10×slope) and the standard deviations of competitor $k_{\mathrm{app,O_3}}$ values were considered in the calculation of the standard deviations of the target $k_{\mathrm{app,O_3}}$ values. Note that the axes do not necessarily originate at zero. S75
SI-B35 Correlations between the natural logarithm of the relative residual peak areas of pheniramine (a - c), pheniramine-*N*-oxide (d), *N*-desmethylpheniramine (e - f) and with competitors upon ozonation at pH 7 (2 mM phosphate), 22 °C, and in presence of *t*BuOH (40 mM). The intercept was considered negligible (<10×slope) and the standard deviations of competitor $k_{\mathrm{app,O_3}}$ values were considered in the calculation of the standard deviations of the target $k_{\mathrm{app,O_3}}$ values. Note that the axes do not necessarily originate at zero. S76

SI-B35 Correlations between the natural logarithm of the relative residual peak areas of prednisone (a - c), prednisolone (d) and methylprednisolone (e - f) with competitors upon ozonation at pH 7 (2 mM phosphate), 22 °C, and in presence of *t*BuOH (40 mM). The intercept was considered negligible (<10×slope) and the standard deviations of competitor k_{app,O_3} values were considered in the calculation of the standard deviations of the target k_{app,O_3} values. Note that the axes do not necessarily originate at zero. S78
SI-B36 Correlations between the natural logarithm of the relative residual peak areas of ranitidine (a), ranitidine-*S*-oxide (b) and ranitidine-*N*-oxide (c - d) and with competitors upon ozonation at pH 7 (2 mM phosphate), 22 °C, and in presence of *t*BuOH (40 mM). The intercept was considered negligible (<10×slope) and the standard deviations of competitor k_{app,O_3} values were considered in the calculation of the standard deviations of the target k_{app,O_3} values. Note that the axes do not necessarily originate at zero. S79
SI-B36 Correlations between the natural logarithm of the relative residual peak areas of sulfapyridine (a - c), 5-hydroxysulfapyridine (d) and N^4-acetylsulfapyridine (e - g) with competitors upon ozonation at pH 7 (2 mM phosphate), 22 °C, and in presence of *t*BuOH (40 mM). The intercept was considered negligible (<10×slope) and the standard deviations of competitor k_{app,O_3} values were considered in the calculation of the standard deviations of the target k_{app,O_3} values. Note that the axes do not necessarily originate at zero. S81
SI-B37 Correlations between the natural logarithm of the relative residual peak areas of theophylline (a - b), theobromine (c - d) and 1,7-dimethyluric acid (e) with competitors upon ozonation at pH 7 (2 mM phosphate), 22 °C, and in presence of *t*BuOH (40 mM). The intercept was considered negligible (<10×slope) and the standard deviations of competitor k_{app,O_3} values were considered in the calculation of the standard deviations of the target k_{app,O_3} values. Note that the axes do not necessarily originate at zero. S82
SI-B38 Correlations between the natural logarithm of the relative residual peak areas of trazodone (a) and 1-(3-chlorophenyl)piperazine (b) with competitors upon ozonation at pH 7 (2 mM phosphate), 22 °C, and in presence of *t*BuOH (40 mM). The intercept was considered negligible (<10×slope) and the standard deviations of competitor k_{app,O_3} values were considered in the calculation of the standard deviations of the target k_{app,O_3} values. Note that the axes do not necessarily originate at zero. S83
SI-B39 Correlations between the natural logarithm of the relative residual peak areas of trimipramine (a), 2-hydroxytrimipramine (b) and trimipramine-*N*-oxide (c) with competitors upon ozonation at pH 7 (2 mM phosphate), 22 °C, and in presence of *t*BuOH (40 mM). The intercept was considered negligible (<10×slope) and the standard deviations of competitor k_{app,O_3} values were considered in the calculation of the standard deviations of the target k_{app,O_3} values. Note that the axes do not necessarily originate at zero. S84
SI-B39 Correlations between the natural logarithm of the relative residual peak areas of telmisartan (a - c), telmisartan-*O*-acyl-glucuronide (d), losartan (e - f) and eprosartan (g - i) with competitors upon ozonation at pH 7 (2 mM phosphate), 22 °C, and in presence of *t*BuOH (40 mM). The intercept was considered negligible (<10×slope) and the standard deviations of competitor k_{app,O_3} values were considered in the calculation of the standard deviations of the target k_{app,O_3} values. Note that the axes do not necessarily originate at zero. S86

SI-B39 Correlations between the natural logarithm of the relative residual peak areas of venlafaxine (a - b), *N*-desvenlafaxine (c - d), *O*-desvenlafaxine (e), *N,O*-didesvenlafaxine (f - g) and venlafaxine-*N*-oxide (h - i) with competitors upon ozonation at pH 7 (2 mM phosphate), 22 °C, and in presence of *t*BuOH (40 mM). The intercept was considered negligible (<10×slope) and the standard deviations of competitor k_{app,O_3} values were considered in the calculation of the standard deviations of the target k_{app,O_3} values. Note that the axes do not necessarily originate at zero. S88
SI-B40 Correlations between the natural logarithm of the relative residual peak areas of verapamil (a) and D617 (b - c) with competitors upon ozonation at pH 7 (2 mM phosphate), 22 °C, and in presence of *t*BuOH (40 mM). The intercept was considered negligible (<10×slope) and the standard deviations of competitor k_{app,O_3} values were considered in the calculation of the standard deviations of the target k_{app,O_3} values. Note that the axes do not necessarily originate at zero. S89
SI-B41 Correlations between the natural logarithm of the relative residual peak areas of nevirapine (a), 2-hydroxynevirapine (b) and 12-hydroxynevirapine (c - d) with competitors upon ozonation at pH 7 (2 mM phosphate), 22 °C, and in presence of *t*BuOH (40 mM). The intercept was considered negligible (<10×slope) and the standard deviations of competitor k_{app,O_3} values were considered in the calculation of the standard deviations of the target k_{app,O_3} values. Note that the axes do not necessarily originate at zero. S90
SI-B42 Correlations between the natural logarithm of the relative residual peak areas of 3-desmethyltrimethoprim (a) and 4-desmethyltrimethoprim (b) with competitors upon ozonation at pH 7 (2 mM phosphate), 22 °C, and in presence of *t*BuOH (40 mM). The intercept was considered negligible (<10×slope) and the standard deviations of competitor k_{app,O_3} values were considered in the calculation of the standard deviations of the target k_{app,O_3} values. Note that the axes do not necessarily originate at zero. S91
SI-B43 Correlations between the natural logarithm of the relative residual peak areas of mefenamic acid (a) and 3-hydroxymethylmefenamic acid (b) with competitors upon ozonation at pH 7 (2 mM phosphate), 22 °C, and in presence of *t*BuOH (40 mM). The intercept was considered negligible (<10×slope) and the standard deviations of competitor k_{app,O_3} values were considered in the calculation of the standard deviations of the target k_{app,O_3} values. Note that the axes do not necessarily originate at zero. S91
SI-B44 Correlations between the natural logarithm of the relative residual peak areas of propanolol (a) and 4-hydroxypropanolol sulfate (b - c) with competitors upon ozonation at pH 7 (2 mM phosphate), 22 °C, and in presence of *t*BuOH (40 mM). The intercept was considered negligible (<10×slope) and the standard deviations of competitor k_{app,O_3} values were considered in the calculation of the standard deviations of the target k_{app,O_3} values. Note that the axes do not necessarily originate at zero. S92
SI-B45 Correlations between the natural logarithm of the relative residual peak areas of mirtazapine (a - c), 8-hydroxymirtazapine (d) and 1-oxo-mirtazapine (e) with competitors upon ozonation at pH 7 (2 mM phosphate), 22 °C, and in presence of *t*BuOH (40 mM). The intercept was considered negligible (<10×slope) and the standard deviations of competitor k_{app,O_3} values were considered in the calculation of the standard deviations of the target k_{app,O_3} values. Note that the axes do not necessarily originate at zero. S93

SI-B46 Correlations between the natural logarithm of the relative residual peak areas of abacavir (a) and abacavir-5'-carboxylate (b) with competitors upon ozonation at pH 7 (2 mM phosphate), 22 °C, and in presence of *t*BuOH (40 mM). The intercept was considered negligible (<10×slope) and the standard deviations of competitor k_{app,O_3} values were considered in the calculation of the standard deviations of the target k_{app,O_3} values. Note that the axes do not necessarily originate at zero. S94
SI-B47 Correlations between the natural logarithm of the relative residual peak areas of clindamycin (a - b) and clindamycin-sulfoxide (c - d) with competitors upon ozonation at pH 7 (2 mM phosphate), 22 °C, and in presence of *t*BuOH (40 mM). The intercept was considered negligible (<10×slope) and the standard deviations of competitor k_{app,O_3} values were considered in the calculation of the standard deviations of the target k_{app,O_3} values. Note that the axes do not necessarily originate at zero. S95
SI-B48 Correlations between the natural logarithm of the relative residual peak areas of bisacodyl (a) and desacetylbisacodyl (b) with competitors upon ozonation at pH 7 (2 mM phosphate), 22 °C, and in presence of *t*BuOH (40 mM). The intercept was considered negligible (<10×slope) and the standard deviations of competitor k_{app,O_3} values were considered in the calculation of the standard deviations of the target k_{app,O_3} values. Note that the axes do not necessarily originate at zero. S96
SI-B49 Correlations between the natural logarithm of the relative residual peak areas of diphenhydramine (a) and diphenhydramine-*N*-glucuronide (b) with competitors upon ozonation at pH 7 (2 mM phosphate), 22 °C, and in presence of *t*BuOH (40 mM). The intercept was considered negligible (<10×slope) and the standard deviations of competitor k_{app,O_3} values were considered in the calculation of the standard deviations of the target k_{app,O_3} values. Note that the axes do not necessarily originate at zero. S97
SI-B50 Correlations between the natural logarithm of the relative residual peak areas of hesperitin (a) and eriodictyol (b) with competitors upon ozonation at pH 7 (2 mM phosphate), 22 °C, and in presence of *t*BuOH (40 mM). The intercept was considered negligible (<10×slope) and the standard deviations of competitor k_{app,O_3} values were considered in the calculation of the standard deviations of the target k_{app,O_3} values. Note that the axes do not necessarily originate at zero. S97
SI-B51 Correlations between the natural logarithm of the relative residual peak areas of lamotrigine (a) and lamotirgine-N2-glucuronide (b - d) with competitors upon ozonation at pH 7 (2 mM phosphate), 22 °C, and in presence of *t*BuOH (40 mM). The intercept was considered negligible (<10×slope) and the standard deviations of competitor k_{app,O_3} values were considered in the calculation of the standard deviations of the target k_{app,O_3} values. Note that the axes do not necessarily originate at zero. S98
SI-B52 Correlations between the natural logarithm of the relative residual peak areas of cocaine (a - b), benzoylecgonine (c) and anhydroecgonine methyl ester (d) with competitors upon ozonation at pH 7 (2 mM phosphate), 22 °C, and in presence of *t*BuOH (40 mM). The intercept was considered negligible (<10×slope) and the standard deviations of competitor k_{app,O_3} values were considered in the calculation of the standard deviations of the target k_{app,O_3} values. Note that the axes do not necessarily originate at zero. S99

SI-B53 Correlations between the natural logarithm of the relative residual peak areas of rosuvastatin (a - b) and *N*-desmethylrosuvastatin (c - e) with competitors upon ozonation at pH 7 (2 mM phosphate), 22 °C, and in presence of *t*BuOH (40 mM). The intercept was considered negligible (<10×slope) and the standard deviations of competitor $k_{\mathrm{app,O_3}}$ values were considered in the calculation of the standard deviations of the target $k_{\mathrm{app,O_3}}$ values. Note that the axes do not necessarily originate at zero. S100
SI-B54 Correlations between the natural logarithm of the relative residual peak areas of pregabalin (a - b) and *N*-methylpregabalin (c - d) with competitors upon ozonation at pH 7 (2 mM phosphate), 22 °C, and in presence of *t*BuOH (40 mM). The intercept was considered negligible (<10×slope) and the standard deviations of competitor $k_{\mathrm{app,O_3}}$ values were considered in the calculation of the standard deviations of the target $k_{\mathrm{app,O_3}}$ values. Note that the axes do not necessarily originate at zero. S101
SI-B55 Correlations between the natural logarithm of the relative residual peak areas of atorvastatin (a), ortho-hydroxyatorvastatin (b) and para-hydroxyatrovastatin (c) with competitors upon ozonation at pH 7 (2 mM phosphate), 22 °C, and in presence of *t*BuOH (40 mM). The intercept was considered negligible (<10×slope) and the standard deviations of competitor $k_{\mathrm{app,O_3}}$ values were considered in the calculation of the standard deviations of the target $k_{\mathrm{app,O_3}}$ values. Note that the axes do not necessarily originate at zero. S102
SI-B56 Correlations between the natural logarithm of the relative residual peak areas of paracetamol (a), paracetamol-glutathione (b) and paracetamol-sulfate (c) with competitors upon ozonation at pH 7 (2 mM phosphate), 22 °C, and in presence of *t*BuOH (40 mM). The intercept was considered negligible (<10×slope) and the standard deviations of competitor $k_{\mathrm{app,O_3}}$ values were considered in the calculation of the standard deviations of the target $k_{\mathrm{app,O_3}}$ values. Note that the axes do not necessarily originate at zero. S103
SI-B57 Correlations between the natural logarithm of the relative residual peak areas of phenylephrine (a) and phenylephrine-3-*O*-sulfate (b - c) with competitors upon ozonation at pH 7 (2 mM phosphate), 22 °C, and in presence of *t*BuOH (40 mM). The intercept was considered negligible (<10×slope) and the standard deviations of competitor $k_{\mathrm{app,O_3}}$ values were considered in the calculation of the standard deviations of the target $k_{\mathrm{app,O_3}}$ values. Note that the axes do not necessarily originate at zero. S104
SI-B58 Correlations between the natural logarithm of the relative residual peak areas of primidone (a) and PEMA (b) with competitors upon ozonation at pH 7 (2 mM phosphate), 22 °C, and in presence of *t*BuOH (40 mM). The intercept was considered negligible (<10×slope) and the standard deviations of competitor $k_{\mathrm{app,O_3}}$ values were considered in the calculation of the standard deviations of the target $k_{\mathrm{app,O_3}}$ values. Note that the axes do not necessarily originate at zero. S105
SI-B59 Correlations between the natural logarithm of the relative residual peak areas of tapentadol (a - c) and tapentadol-*O*-sulfate (d - e) with competitors upon ozonation at pH 7 (2 mM phosphate), 22 °C, and in presence of *t*BuOH (40 mM). The intercept was considered negligible (<10×slope) and the standard deviations of competitor $k_{\mathrm{app,O_3}}$ values were considered in the calculation of the standard deviations of the target $k_{\mathrm{app,O_3}}$ values. Note that the axes do not necessarily originate at zero. S106

SI-B60 Correlations between the natural logarithm of the relative residual peak areas of torasemide (a - b), hydroxytorasemide (c) and torasemide carboxylic acid (d - e) with competitors upon ozonation at pH 7 (2 mM phosphate), 22 °C, and in presence of *t*BuOH (40 mM). The intercept was considered negligible (<10×slope) and the standard deviations of competitor k_{app,O_3} values were considered in the calculation of the standard deviations of the target k_{app,O_3} values. Note that the axes do not necessarily originate at zero. S107
SI-B61 Correlations between the natural logarithm of the relative residual peak areas of zolpidem (a) and zolpidem carboxylic acid (b) with competitors upon ozonation at pH 7 (2 mM phosphate), 22 °C, and in presence of *t*BuOH (40 mM). The intercept was considered negligible (<10×slope) and the standard deviations of competitor k_{app,O_3} values were considered in the calculation of the standard deviations of the target k_{app,O_3} values. Note that the axes do not necessarily originate at zero. S108
SI-B62 Correlations between the natural logarithm of the relative residual peak areas of naproxen (a - b), *O*-desmethylnaproxen (c) and naproxen methyl ester (d - e) with competitors upon ozonation at pH 7 (2 mM phosphate), 22 °C, and in presence of *t*BuOH (40 mM). The intercept was considered negligible (<10×slope) and the standard deviations of competitor k_{app,O_3} values were considered in the calculation of the standard deviations of the target k_{app,O_3} values. Note that the axes do not necessarily originate at zero. S109
SI-B63 Correlations between the natural logarithm of the relative residual peak areas of levetiracetam (a) and levetiracetam acid (b) with competitors upon ozonation at pH 7 (2 mM phosphate), 22 °C, and in presence of *t*BuOH (40 mM). The intercept was considered negligible (<10×slope) and the standard deviations of competitor k_{app,O_3} values were considered in the calculation of the standard deviations of the target k_{app,O_3} values. Note that the axes do not necessarily originate at zero. S110
SI-B64 Correlations between the natural logarithm of the relative residual peak areas of gabapentin (a) and gabapentin-lactam (b) with competitors upon ozonation at pH 7 (2 mM phosphate), 22 °C, and in presence of *t*BuOH (40 mM). The intercept was considered negligible (<10×slope) and the standard deviations of competitor k_{app,O_3} values were considered in the calculation of the standard deviations of the target k_{app,O_3} values. Note that the axes do not necessarily originate at zero. S110
SI-B65 Correlations between the natural logarithm of the relative residual peak areas of sulfathiazole (a), N^4-acetylsulfathiazole (b - c) and pterin-sulfathiazole (d - e) with competitors upon ozonation at pH 7 (2 mM phosphate), 22 °C, and in presence of *t*BuOH (40 mM). The intercept was considered negligible (<10×slope) and the standard deviations of competitor k_{app,O_3} values were considered in the calculation of the standard deviations of the target k_{app,O_3} values. Note that the axes do not necessarily originate at zero. S111
SI-B65 Correlations between the natural logarithm of the relative residual peak areas of benzotriazole (a - b), 4-methylbenzotriazole (c - d) and 5-methylbenzotriazole (e - f) with competitors upon ozonation at pH 7 (2 mM phosphate), 22 °C, and in presence of *t*BuOH (40 mM). The intercept was considered negligible (<10×slope) and the standard deviations of competitor k_{app,O_3} values were considered in the calculation of the standard deviations of the target k_{app,O_3} values. Note that the axes do not necessarily originate at zero. S113

SI-B66 Correlations between the natural logarithm of the relative residual peak areas of azoxystrobin (a) and azoxystrobin acid (b - c) with competitors upon ozonation at pH 7 (2 mM phosphate), 22 °C, and in presence of *t*BuOH (40 mM). The intercept was considered negligible (<10×slope) and the standard deviations of competitor $k_{\mathrm{app,O_3}}$ values were considered in the calculation of the standard deviations of the target $k_{\mathrm{app,O_3}}$ values. Note that the axes do not necessarily originate at zero. S114
SI-B67 Correlations between the natural logarithm of the relative residual peak areas of 1-hydroxyibuprofen (a) and caroxyibuprofen (b) with competitors upon ozonation at pH 7 (2 mM phosphate), 22 °C, and in presence of *t*BuOH (40 mM). The intercept was considered negligible (<10×slope) and the standard deviations of competitor $k_{\mathrm{app,O_3}}$ values were considered in the calculation of the standard deviations of the target $k_{\mathrm{app,O_3}}$ values. Note that the axes do not necessarily originate at zero. S115
SI-B68 Correlations between the natural logarithm of the relative residual peak areas of iopromide (a) and desmethoxyiopromide (b) with competitors upon ozonation at pH 7 (2 mM phosphate), 22 °C, and in presence of *t*BuOH (40 mM). The intercept was considered negligible (<10×slope) and the standard deviations of competitor $k_{\mathrm{app,O_3}}$ values were considered in the calculation of the standard deviations of the target $k_{\mathrm{app,O_3}}$ values. Note that the axes do not necessarily originate at zero. S115
SI-B69 Correlations between the natural logarithm of the relative residual peak areas of ketamine (a - b) and norketamine (c - d) with competitors upon ozonation at pH 7 (2 mM phosphate), 22 °C, and in presence of *t*BuOH (40 mM). The intercept was considered negligible (<10×slope) and the standard deviations of competitor $k_{\mathrm{app,O_3}}$ values were considered in the calculation of the standard deviations of the target $k_{\mathrm{app,O_3}}$ values. Note that the axes do not necessarily originate at zero. S116
SI-B70 Correlations between the natural logarithm of the relative residual peak areas of pantoprazole (a - b) and 4-*O*-desmethylpantoprazole-sulfide (c - e) with competitors upon ozonation at pH 7 (2 mM phosphate), 22 °C, and in presence of *t*BuOH (40 mM). The intercept was considered negligible (<10×slope) and the standard deviations of competitor $k_{\mathrm{app,O_3}}$ values were considered in the calculation of the standard deviations of the target $k_{\mathrm{app,O_3}}$ values. Note that the axes do not necessarily originate at zero. S117
SI-B71 Correlations between the natural logarithm of the relative residual peak areas of sulfadiazine (a - c) and N^4-acetylsulfamethoxazole (d - e) with competitors upon ozonation at pH 7 (2 mM phosphate), 22 °C, and in presence of *t*BuOH (40 mM). The intercept was considered negligible (<10×slope) and the standard deviations of competitor $k_{\mathrm{app,O_3}}$ values were considered in the calculation of the standard deviations of the target $k_{\mathrm{app,O_3}}$ values. Note that the axes do not necessarily originate at zero. S118
SI-B72 Correlations between the natural logarithm of the relative residual peak areas of sulfadimethoxine (a - c) and N^4-acetylsulfadimethoxine (d - f) with competitors upon ozonation at pH 7 (2 mM phosphate), 22 °C, and in presence of *t*BuOH (40 mM). The intercept was considered negligible (<10×slope) and the standard deviations of competitor $k_{\mathrm{app,O_3}}$ values were considered in the calculation of the standard deviations of the target $k_{\mathrm{app,O_3}}$ values. Note that the axes do not necessarily originate at zero. S119

SI-B73 Correlations between the natural logarithm of the relative residual peak areas of sulfamethazine (a - c) and N^4-acetylsulfamethazine (d - e) with competitors upon ozonation at pH 7 (2 mM phosphate), 22 °C, and in presence of *t*BuOH (40 mM). The intercept was considered negligible (<10×slope) and the standard deviations of competitor k_{app,O_3} values were considered in the calculation of the standard deviations of the target k_{app,O_3} values. Note that the axes do not necessarily originate at zero. S120
SI-B74 Correlations between the natural logarithm of the relative residual peak areas of sulpiride (a) and sulpiride-*N*-oxide (b) with competitors upon ozonation at pH 7 (2 mM phosphate), 22 °C, and in presence of *t*BuOH (40 mM). The intercept was considered negligible (<10×slope) and the standard deviations of competitor k_{app,O_3} values were considered in the calculation of the standard deviations of the target k_{app,O_3} values. Note that the axes do not necessarily originate at zero. S121
SI-B75 Correlations between the natural logarithm of the relative residual peak areas of desloratadine (a - b) and 3-hydroxydesloratadine (c) with competitors upon ozonation at pH 7 (2 mM phosphate), 22 °C, and in presence of *t*BuOH (40 mM). The intercept was considered negligible (<10×slope) and the standard deviations of competitor k_{app,O_3} values were considered in the calculation of the standard deviations of the target k_{app,O_3} values. Note that the axes do not necessarily originate at zero. S122
SI-B76 Correlations between the natural logarithm of the relative residual peak areas of aliskiren with competitor roxithromycin upon ozonation at pH 7 (2 mM phosphate), 22 °C, and in presence of *t*BuOH (40 mM). The intercept was considered negligible (<10×slope) and the standard deviations of competitor k_{app,O_3} values were considered in the calculation of the standard deviations of the target k_{app,O_3} values. Note that the axes do not necessarily originate at zero. S123
SI-B77 Correlations between the natural logarithm of the relative residual peak areas of didanosine with competitor alachlor upon ozonation at pH 7 (2 mM phosphate), 22 °C, and in presence of *t*BuOH (40 mM). The intercept was considered negligible (<10×slope) and the standard deviations of competitor k_{app,O_3} values were considered in the calculation of the standard deviations of the target k_{app,O_3} values. Note that the axes do not necessarily originate at zero. S123
SI-B78 Correlations between the natural logarithm of the relative residual peak areas of lamivudine with competitor roxithromycin upon ozonation at pH 7 (2 mM phosphate), 22 °C, and in presence of *t*BuOH (40 mM). The intercept was considered negligible (<10×slope) and the standard deviations of competitor k_{app,O_3} values were considered in the calculation of the standard deviations of the target k_{app,O_3} values. Note that the axes do not necessarily originate at zero. S124
SI-B79 Correlations between the natural logarithm of the relative residual peak areas of *O*-desarylranolazine with competitors upon ozonation at pH 7 (2 mM phosphate), 22 °C, and in presence of *t*BuOH (40 mM). The intercept was considered negligible (<10×slope) and the standard deviations of competitor k_{app,O_3} values were considered in the calculation of the standard deviations of the target k_{app,O_3} values. Note that the axes do not necessarily originate at zero. S125

SI-B80 Correlations between the natural logarithm of the relative residual peak areas of omeprazole with competitor roxithromycin upon ozonation at pH 7 (2 mM phosphate), 22 °C, and in presence of *t*BuOH (40 mM). The intercept was considered negligible (<10×slope) and the standard deviations of competitor k_{app,O_3} values were considered in the calculation of the standard deviations of the target k_{app,O_3} values. Note that the axes do not necessarily originate at zero. S126
SI-B81 Correlations between the natural logarithm of the relative residual peak areas of phenolic glucuronide with competitor alachlor upon ozonation at pH 7 (2 mM phosphate), 22 °C, and in presence of *t*BuOH (40 mM). The intercept was considered negligible (<10×slope) and the standard deviations of competitor k_{app,O_3} values were considered in the calculation of the standard deviations of the target k_{app,O_3} values. Note that the axes do not necessarily originate at zero. S126
SI-B82 Correlations between the natural logarithm of the relative residual peak areas of sacubitrilat (LBQ657) with competitor alachlor upon ozonation at pH 7 (2 mM phosphate), 22 °C, and in presence of *t*BuOH (40 mM). The intercept was considered negligible (<10×slope) and the standard deviations of competitor k_{app,O_3} values were considered in the calculation of the standard deviations of the target k_{app,O_3} values. Note that the axes do not necessarily originate at zero. S127
SI-B83 Correlations between the natural logarithm of the relative residual peak areas of tolperisone with competitors penicillin G, roxithromycin and tramadol upon ozonation at pH 7 (2 mM phosphate), 22 °C, and in presence of *t*BuOH (40 mM). The intercept was considered negligible (<10×slope) and the standard deviations of competitor k_{app,O_3} values were considered in the calculation of the standard deviations of the target k_{app,O_3} values. Note that the axes do not necessarily originate at zero. S128
SI-B84 Evolution of peak areas of 5-hydroxydiclofenac, the benzoquinoneimine counterpart, and the competitor bezafibrate (left), and of 4-hydroxydiclofenac and its benzoquinoneimine counterpart (right) with increasing applied specific ozone doses in ultrapure water at pH 7, and with excess of tBuOH to scavenge hydroxyl radicals. Specific ozone doses ranged from 0.02 to 690 mol ozone per mol of 5-hydroxydiclofenac (left), and from 2 to 1560 mol ozone per mol of 4-hydroxydiclofenac (right). Note that these specific ozone doses cannot be taken as absolute values, since other compounds and competitors are present in solution which contribute to ozone consumption. S136
SI-B85 Ratios of measured second-order rate constants for the reactions with ozone of hydroxylated metabolite-parent pairs. The gray lines indicate a reactivity difference of a factor two. The structures of the hydroxylated metabolites are shown below, with the hydroxyl moiety added compared to the parent highlighted in green. S143
SI-B86 Ratios of measured second-order rate constants for the reactions with ozone of *N*-oxide metabolite-parent pairs. The gray lines indicate a reactivity difference of a factor two. The structures of the *N*-oxide metabolites are shown below, with the the *N*-oxide moiety added compared to the parent highlighted in green. S144

SI-B87 Ratios of measured second-order rate constants for the reactions with ozone of carboxylated metabolite-parent pairs. The gray lines indicate a reactivity difference of a factor two. The structures are shown below, with the moieties removed compared to the parent highlighted in yellow and the moieties added compared to the parent in green. S145
SI-B88 Ratios of measured second-order rate constants for the reactions with ozone of conjugated metabolite-parent pairs. The gray lines indicate a reactivity difference of a factor two. The comparison also includes the parents bisacodyl and diltiazem, as well as the metabolites desacetylbiscaodyl and desacetyldiltiazem. As for the other compound pairs, the second-order rate constant of the acetylated/conjugated compound is divided by the one of the non-acetylated/non-conjugated compound. Compared to the other pairs however, the roles of parent and metabolite are reversed. The structures are shown below, with the moieties removed compared to the parent highlighted in yellow and the moieties added compared to the parent in green. Figure continued on next page. S146
SI-B89 Ratios of measured second-order rate constants for the reactions with ozone of dealkylated metabolite-parent pairs. The gray lines indicate a reactivity difference of a factor two. The comparison also includes the parent pregabalin and the conjugated metabolite *N*-methylpregabalin. As for the other compound pairs, the second-order rate constant of the dealkylated compound is divided by the one of the alkylated compound. Compared to the other pairs however, the roles of parent and metabolite are reversed. The structures are shown below, with the moieties removed compared to the parent highlighted in yellow and the moieties added compared to the parent in green. Figure continued on next page. S148
SI-B90 Prediction of $k_{^{\bullet}\mathrm{OH}}$. Violin plots of the logarithmic ratios of literature and predicted $k_{^{\bullet}\mathrm{OH}}$ values of 44 compounds. The white diamonds indicate the mean values. Compounds with more than a factor two deviation from literature are labeled: allopurinol (ALP), amoxicillin (AMX), atenolol acid (ATE-COOH), atrovastatin (ATV), clarithromycin (CLR), clindamycin (CLI), erythromycin (ERY), fluoxetine (FXT), gabapentin (GBP), hydrocortisone (HCORT), iopromide (IOP), lidocaine (LID), prednisolone (PSL), succinic acid (SCA), ranitidine (RNT) and sulfadimethoxine (SDM). S150
SI-B91 Measured relative abatements of studied compounds in the ozonation of wastewater treatment plant (WWTP) Werdhoelzli versus modeled relative abatements using experimental $k_{\mathrm{O_3}}$ (A) or predicted $k_{\mathrm{O_3}}$ values (B). The specific ozone dose was $0.6\,\mathrm{g_{O_3}/g_{DOC}}$ and the ozone exposure was set to 2.8×10^{-4} Ms. The error bars of the model were determined with Monte Carlo sampling, while the error bars from the wastewater treatment plant (WWTP) removal correspond to standard deviations from the triplicate analysis and the five consecutive sampling days. Compounds with common functional groups refer to anilines, amines, benzenes, olefins and phenol(ate)s. S152

SI-B92 Measured relative abatements of studied compounds in the ozonation of wastewater treatment plant (WWTP) Altenrhein versus modeled relative abatements using experimental k_{O_3} (A) or predicted k_{O_3} values (B). The specific ozone dose was $0.1\,g_{O_3}/g_{DOC}$ and the ozone exposure was set to 1.3×10^6 Ms. The error bars of the model were determined with Monte Carlo sampling, while the error bars from the wastewater treatment plant (WWTP) removal correspond to standard deviations from the triplicate analysis and the five consecutive sampling days. Compounds with common functional groups refer to anilines, amines, benzenes, olefins and phenol(ate)s. S153
SI-B93 Influence of k_{O_3}, $k_{^{\bullet}OH}$, O_3 exposure and $^{\bullet}OH$ exposure on abatement of micropollutants during wastewater ozonation. (A) Abatement during ozonation as a function of k_{O_3} for the three wastewater treatment plants (WWTPs) Altenrhein, Neugut and Werdhoelzli, assuming their $^{\bullet}OH$ and O_3 exposures as constant based on the laboratory experiments and literature values, respectively. Three different $k_{^{\bullet}OH}$ values in the predicted range were considered. (B) Derivative-based local sensitivity analysis with respect to k_{O_3} and O_3 exposure and (C) with respect to $k_{^{\bullet}OH}$ and $^{\bullet}OH$ exposure for wastewater treatment plant (WWTP) Neugut. Light green diamonds indicate mean values. S154
SI-B94 Influence of k_{O_3}, $k_{^{\bullet}OH}$, O_3 exposure and $^{\bullet}OH$ exposure on abatement of micropollutants during wastewater ozonation. Derivative-based local sensitivity analysis with respect to k_{O_3} and O_3 exposure (B) and with respect to $k_{^{\bullet}OH}$ and $^{\bullet}OH$ exposure (C) for wastewater treatment plant (WWTP) Altenrhein. Light blue diamonds indicate mean values. S155
SI-B95 Influence of k_{O_3}, $k_{^{\bullet}OH}$, O_3 exposure and $^{\bullet}OH$ exposure on abatement of micropollutants during wastewater ozonation. Derivative-based local sensitivity analysis with respect to k_{O_3} and O_3 exposure (B) and with respect to $k_{^{\bullet}OH}$ and $^{\bullet}OH$ exposure (C) for wastewater treatment plant (WWTP) Werdhoelzli. Yellow diamonds indicate mean values. S155

List of Tables

SI-B1 Chemicals and Solvents. S20

SI-B2 List of 15 selected competitors with molecular structures and species-specific second order rate constants for their reactions with ozone. S21

SI-B3 Time schedule of the liquid chromatography in HPLC-HRMS/MS analysis. . S23

SI-B4 ESI-HRMS/MS settings. S23

SI-B5 Parameters characterizing the secondary effluent of wastewater treatment plants (WWTPs) Altenrhein, Neugut and Werdhoelzli used for $^{\bullet}$OH and O_3 exposure experiments. S24

SI-B6 Time schedule of the liquid chromatography in HPLC-UV/Vis analysis. . . . S24

SI-B7 Comparison of literature and experimental $k_{\mathrm{app},\mathrm{O}_3}$ values at pH 7. Of the 27 compounds, 14 compound have a deviation of less than a factor 2, 9 compounds of less than a factor of 5, 3 compounds are within one order of magnitude and one compound (telmisartan) deviates by more than one order of magnitude. S129

SI-B8 Outliers in the comparison between determined (multi-compound competition kinetics) and predicted values for $k_{\mathrm{app},\mathrm{O}_3}$ with the Q$k_{\mathrm{app},\mathrm{O}_3}$ defined as $\log_{10}(k_{\mathrm{O}_3,\mathrm{measured}}/k_{\mathrm{O}_3,\mathrm{estimated}})$. The compounds are ordered along increasing $\Delta k_{\mathrm{app},\mathrm{O}_3}$ S134

SI-B9 Second-order rate constants of $^{\bullet}$OH with DOM, (bi(carbonate) alkalinity, nitrite and bromide and the relative contributions of $^{\bullet}$OH scavengers in the three wastewater matrices S151

SI-B1 Materials & Methods

SI-B1.1 Chemicals and Solvents

Table SI-B1: Chemicals and Solvents.

Chemical, solvent	Purity [%]	Molecular formula	Molecular weight [g/mol]	CAS-No.	Manufacturer	Article number
Acetonitrile	LC/MS grade	C_2H_3N	41.05	75-05-8	Fisher Scientific GmbH	A955-212
Ammonium acetate	99.999	$C_2H_7NO_2$	77.08	631-61-8	Sigma-Aldrich	372331-100G
Ammonium formate	≥99.0	CH_5NO_2	63.06	540-69-2	Sigma-Aldrich	70221-100G-F
Disodium hydrogen citrate sesquihydrate	99.0	$Na_2C_6H_6O_7 \cdot 1.5H_2O$	254.10	6132-05-4	Fluka	71635
Formic acid	98-100	CH_2O_2	46.03	64-18-6	Merck	1.00264.0100
Methanol	LC/MS grade	CH_4O	32.04	67-56-1	Fisher Scientific GmbH	A456-212
para-Chlorobenzoic acid	99	$C_7H_5ClO_2$	156.56	74-11-3	Aldrich	135585-50G
Phosphoric acid	≥85	H_3PO_4	98.00	7664-38-2	Sigma-Aldrich	30417-1L
Sodium citrate tribasic dihydrate	≥99.0	$Na_3C_6H_5O_7 \cdot 2H_2O$	294.10	6132-04-3	Sigma-Aldrich	71405-250G
Sodium dihydrogen phosphate monohydrate	99.0-102.0	$NaH_2PO_4 \cdot H_2O$	137.99	10049-21-5	Merck	6346.0500
Sodium phosphate dibasic dihydrate	98.5-101.0	$Na_2HPO_4 \cdot 2H_2O$	177.99	10028-24-7	Sigma-Aldrich	30435-1KG
tert-Butanol	≥99.7	$C_4H_{10}O$	74.12	75-65-0	Sigma-Aldrich	19460-500ML

SI-B1.2 Ozonation Experiments

SI-B1.2.1 Competitor Compounds

The same competitor compounds as in the study of Rougé *et al.*[1] were used, excluding ciprofloxacin due to difficulties in analysis. All competitors with their molecular structures and second-order rate constants for the reactions with ozone are listed in Table SI-B2.

Table SI-B2: List of 15 selected competitors with molecular structures and species-specific second order rate constants for their reactions with ozone.

Competitor	Structure	pK_a	k_{O_3} [M^{-1} s^{-1}]	k_{app,O_3} pH 7	Reference
Alachlor	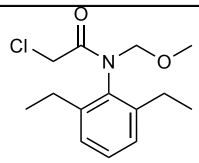		3.8 ± 0.4	3.8 ± 0.4	2
Bezafibrate	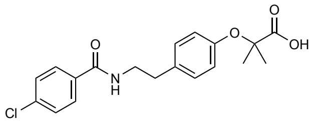		$(5.9 \pm 0.5) \cdot 10^2$	$(5.9 \pm 0.5) \cdot 10^2$	3
Carbamazepine	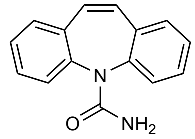		$(6.1 \pm 0.1) \cdot 10^5$	$(6.1 \pm 0.1) \cdot 10^5$	4
Carbofuran	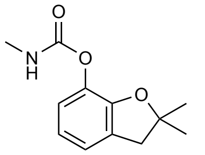		$2.1 \cdot 10^2$	$2.1 \cdot 10^2$	1,2
Diazepam	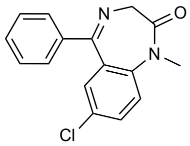		$(7.5 \pm 0.15) \cdot 10^{-1}$	$(7.5 \pm 0.2) \cdot 10^{-1}$	3
Dibromomethyl-paraben	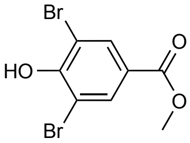	4.7	$k_1 = 2.2 \cdot 10^1$<br>$k_2 = (4.3 \pm 0.3) \cdot 10^6$	$(4.3 \pm 0.3) \cdot 10^6$	1,5
N^4-Acetylsulfa-methoxazole	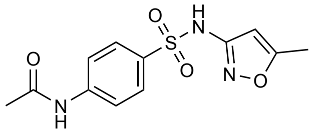	5.5	$k_1 = (2.0 \pm 0.2) \cdot 10^1$<br>$k_2 = (2.6 \pm 0.1) \cdot 10^2$	$(2.5 \pm 0.1) \cdot 10^2$	6
Penicillin G	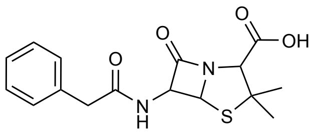		$(4.8 \pm 0.1) \cdot 10^3$	$(4.8 \pm 0.1) \cdot 10^3$	6
Picloram	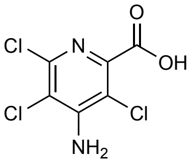	3.6	$k_1 = (5.0 \pm 1.0) \cdot 10^1$<br>$k_2 = (1.4 \pm 0.2) \cdot 10^2$	$(1.4 \pm 0.2) \cdot 10^2$	2
Roxithromycin	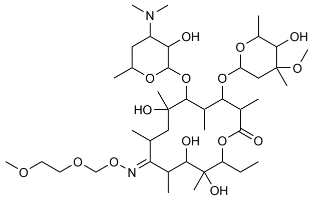	9.2	$k_1 < 1$<br>$k_2 = (1.0 \pm 0.1) \cdot 10^7$	$(6.3 \pm 1.4) \cdot 10^{s}4$	6

Sulfamethoxazole	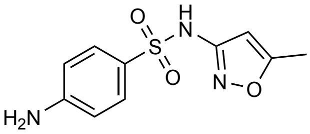	5.6	$k_1 = (9.4 \pm 1.8) \cdot 10^4$<br>$k_2 = (1.1 \pm 0.2) \cdot 10^6$	$(1.1 \pm 0.2) \cdot 10^6$	[1,6]
Tramadol	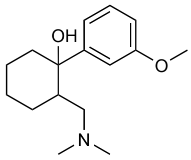	9.4	$k_1 = (7.7 \pm 0.2) \cdot 10^1$<br>$k_2 = (1.0 \pm 0.1) \cdot 10^6$	$(4.0 \pm 0.9) \cdot 10^3$	[7]
Triclosan	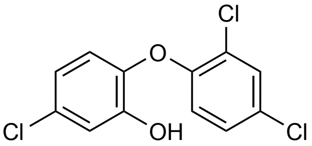	8.1	$k_1 = (1.3 \pm 0.1) \cdot 10^3$<br>$k_2 = (5.1 \pm 0.1) \cdot 10^8$	$(3.8 \pm 0.8) \cdot 10^7$	[8]
Trimethoprim	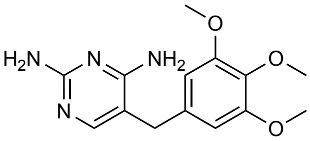	3.2 7.1	$k_1 = (6.6 \pm 6.0) \cdot 10^4$<br>$k_2 = (1.5 \pm 0.4) \cdot 10^5$<br>$k_3 = (1.0 \pm 0.2) \cdot 10^6$	$(5.4 \pm 1.1) \cdot 10^5$	[1,6]
Tylosin	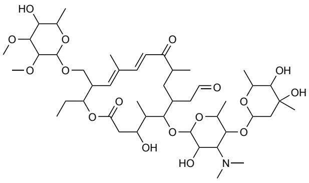	7.7	$k_1 = (1.5 \pm 0.3) \cdot 10^5$<br>$k_2 = (5.4 \pm 1.0) \cdot 10^6$	$(1.0 \pm 0.2) \cdot 10^5$	[1,6]

SI-B1.2.2 HPLC-HRMS/MS

The high performance liquid chromatography (HPLC) gradient is provided in Tables SI-B3 and SI-B4. Mass spectra were acquired separately in ESI positive and negative mode on a high-resolution mass spectrometer (Q Exactive Plus, Thermo Fisher Scientific, U.S.). Full scan MS1 acquisition (m/z: 100 to 1000) was performed with a mass resolution of 140 000 at 200 m/z, followed by five data-dependent MS/MS scans with a resolution of 17 500 at 200 m/z using higher energy collision-induced dissociation (HCD) and an isolation window of 1 Da. MS/MS data acquisitions were triggered by m/z of target ions. Normalized collision energies (NCEs) were calculated for each compound based on Equation 0.1 and rounded to the nearest number divisible by five lying between 15 and 120. If no suspect was detected, the five most intense ions present in the MS1 spectrum were fragmented. Table SI-B4 provides more detailed mass spectrometer settings.

$$\mathrm{NCE} = \begin{cases} \mathrm{m/z} \cdot (-0.41) + 160 & \mathrm{m/z} < 350 \\ 15 & \mathrm{m/z} > 350 \end{cases} \tag{0.1}$$

Table SI-B3: Time schedule of the liquid chromatography in HPLC-HRMS/MS analysis.

**Time** [min]	**Eluent A** [µL/min]	**Eluent B** [µL/min]
0.0	285	15
1.0	285	15
17.0	15	285
25.0	15	285
25.1	285	15
30.0	285	15

Table SI-B4: ESI-HRMS/MS settings.

Ionization source	Spray voltage (positive/negative mode)	+4/-3
	Sheath gas flow rate (nitrogen)	40 L/min
	Auxiliary gas flow rate (nitrogen)	15 L/min
	Transfer capillary temperature	320 °C
	Auxiliary gas heater temperature	50 °C
MS^1	Mass resolution	140 000
	Scan range (m/z)	100 - 1000
	AGC target	100 000
	Maximal injection time	100 ms
MS^2	Mass resolution	17 500
	AGC target	10 000
	Maximal injection time	50 ms

SI-B1.2.3 Secondary effluent characterization

To characterize the secondary effluent prior to $^{\bullet}OH$ exposure measurements, the pH value and the concentrations of dissolved organic carbon (DOC), nitrite (NO_2^-), nitrate (NO_3^-), ammonium (NH_4^+), bromide (Br^-) and the alkalinity were determined. For all analyses, the secondary effluents were first filtered through glass microfiber filters of pore size 0.7 µm (Whatman, U.K.) before filtering through polypropylene syringe filters with a pore size of 0.45 µm (BGB Analytik, Switzerland). DOC concentrations were obtained from a total organic carbon analyzer (Shimadzu TOCL CSH, Japan) with a limit of quantification (LOQ) of 0.5 mg/L. Bromide and nitrate concentrations were obtained by ion chromatography (Metrohm 930 Compact IC Flex, Switzerland) with LOQs corresponding to 0.05 mg/L and 0.1 mg/L, respectively. Nitrite and ammonium concentrations were determined spectrophotometrically (Agilent Cary 60, U.S.) with a LOQ of 1 µg/L for nitrite and 5 µg/L for ammonium. The pH value and the alkalinity were measured with a potentiometric titrator (Metrohm 809 Titrando, Switzerland), with a LOQ of 0.2 mmol/L for the alkalinity. All obtained parameters are summarized in Table SI-B5.

Table SI-B5: Parameters characterizing the secondary effluent of wastewater treatment plants (WWTPs) Altenrhein, Neugut and Werdhoelzli used for $^{\bullet}$OH and O_3 exposure experiments.

	Altenrhein	Neugut	Werdhoelzli
pH	7.99	8.22	8.22
DOC [mg/L]	6.6	4.4	6.4
Bromide [mg/L]	0.055	<0.05	<0.05
Nitrite [µg/L]	23.88	6.13	75.00
Nitrate [mg/L]	41.69	7.28	5.02
Ammonium [µg/L]	40.27	41.73	202.91
Alkalinity [mmol/L]	4.2	5.3	3.4

SI-B1.2.4 $^{\bullet}$OH exposure

$^{\bullet}$OH exposures for the three different wastewater treatment plants (WWTPs) were obtained by adding the respective ozone doses (g_{O_3}/g_{DOC}) applied at the three WWTPs to 50 mL aliquots of secondary effluents. For WWTP Altenrhein, this dose corresponds to 0.1 g_{O_3}/g_{DOC}, while for Neugut and Werdhoelzli higher doses of 0.4 g_{O_3}/g_{DOC} and 0.6 g_{O_3}/g_{DOC} are applied, respectively. As $^{\bullet}$OH probe for $^{\bullet}$OH exposure determination, *para*-chlorobenzoic acid (*p*CBA) was used. A stock solution of *p*CBA in ethanol was added to empty 100 mL borosilicate bottles (previously annealed at 500 °C; Duran, Germany or SIMAX Kavalier, Czech Republic) and evaporated to dryness over night, before 50 mL of secondary effluent were added, yielding a *p*CBA concentration of 1 µM. The aliquots were ozonated from a 0.2 mM stock solution.

The $^{\bullet}$OH exposures were calculated from the elimination of the ozone-recalcitrant compound *p*CBA, as previously described by Lee *et al.*:[9]

$$\int [^{\bullet}\mathrm{OH}]dt = \frac{-\ln\left(\frac{[pCBA]}{[pCBA]_0}\right)}{k_{^{\bullet}\mathrm{OH}}} \quad , \qquad (0.2)$$

where $k_{^{\bullet}OH}$ is the second-order rate constant for the reaction of *p*CBA with $^{\bullet}$OH (5×10^9 $M^{-1}s^{-1}$).[10] Analysis of *p*CBA was performed by HPLC (Ultimate 3000, Thermo Fisher Scientific, U.S.) with a reversed-phase C18 column at 30 °C (XBridge, 3.5 µm, 2.1 x 50 mm, Waters, U.S.) and a diode array detector (DAD). The concentrations before the addition of ozone ($[pCBA]_0$) and after complete ozone consumption ([pCBA]) were replaced by the areas obtained from the UV/Vis spectra measured at 240 nm, whereby *p*CBA has a retention time of 4.9 min. Eluent A was ultrapure water with 10 mM H_3PO_4 and eluent B pure methanol. The detailed gradient is given in Table SI-B6.

Table SI-B6: Time schedule of the liquid chromatography in HPLC-UV/Vis analysis.

**Time [min]**	**Eluent A** [µL/min]	**Eluent B** [µL/min]
0.0	420	180
1.0	420	180
5.0	270	330
15.0	120	480
16.5	420	180
20.0	420	180

SI-B1.3 $k_{^\bullet OH}$ prediction

SI-B1.3.1 Group contribution method

To apply the group contribution method (GCM) developed by Minakata *et al.* (2009) for predicting aqueous-phase hydroxyl radical reaction rate constants, the process involves systematically breaking down a molecule into its functional groups and using predefined contribution values to estimate its overall reactivity. The first step is to determine which of the four key reaction mechanisms apply to the compound: (i) H-atom abstraction, (ii) hydroxyl radical addition to alkenes, (iii) hydroxyl radical addition to aromatic rings (iv) or reactions with sulfur, nitrogen or phosphorus-containing moieties. Once identified, the molecule is decomposed into its α- and β-positioned functional groups, which influence its reactivity. Each of these groups has an associated base activation energy (E_a^0) and a modifying contribution ($E_a^{R_i}$), both of which are provided in the model's dataset. The supporting information of the original publication provides an Excel spread sheet, which can be used to predict $k_{^\bullet OH}$ values. This sheet was used for our study compounds and the Excel SI-C provides a filled sheet for each compound.

The following excerpts describe based on the structure of atenolol how $k_{^\bullet OH}$ values are determined with the GCM. First, the four key reaction mechanisms possible for atenolol are identified (see Figure SI-B1). It becomes visible that several sites for H-atom abstraction are present in the molecule (yellow), but also a 1,4-substituted benzene ring (blue) for hydroxyl radical addition to aromatic rings and a secondary amine and a primary amide (green), covering the fourth reaction type. No alkenes are present in the structure, meaning the second reaction type does not contribute and can be neglected.

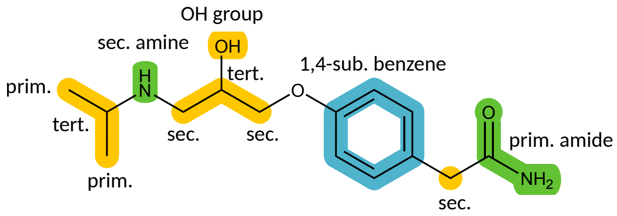

Figure SI-B1: Three of the four key reaction mechanisms included in GCM are possible for atenolol: H-atom abstraction (yellow), hydroxyl radical addition to aromatic rings (blue) and reaction with nitrogen-containing moieties (green).

Figure SI-B2 shows the section in the Excel sheet relevant for H-atom abstraction. It is subdivided into reactions for primary, secondary and tertiary carbon atoms, and contains additional potential H-atom abstraction sites, e.g. hydroxy and carboxylic acid groups. The total contribution of H-atom abstraction is given by the sum of these four subcontributions. It is thereby considered that primary carbon atoms have three bound H-atoms which can be abstracted, while tertiary carbon atoms only have one. The effect of the neighboring substituents can be considered by adding the group contribution factors X to the table, which are factor by which the base group rate constant is multiplied. The two primary carbon atoms of atenolol are bound to a tertiary carbon atom, corresponding to an X of 1.174. Since we have two identical primary carbon atoms, this value is filled twice (into the second and third column). Next, the secondary carbon atoms are considered, which have two non-H substituents. Hence, two different contribution factors X have to be considered. One secondary carbon atom is bound to a tertiary carbon ($X = 1.174$) and to a secondary amine ($X = 1.63$). The other secondary carbon atoms are bound to a tertiary carbon ($X = 1.174$) and an ether ($X = 0.551$) or to an aromatic ring ($X = 1$) and a primary amide. Since no group contribution factor X for a primary amide is available, it was approximated by the one of -COO/-COOH ($X = 0.043$). Additionally, atenolol has one hydroxy group, which is added in the third last row. According to the equation displayed in Figure SI-B2, this yields a contribution from H-atom abstraction for atenolol of 9.24×10^9 $M^{-1}s^{-1}$.

Back to overview

Atenolol

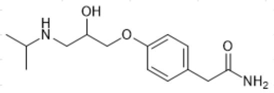

$k_{overall}$ 1.49E+10

Hydrogen-Atom Abstraction: $$k_{abs} = 3\sum_{0}^{I} k^{0}_{prim} X_{R_1} + 2\sum_{0}^{J} k^{0}_{sec} X_{R_1} X_{R_2} + \sum_{0}^{K} k^{0}_{tert} X_{R_1} X_{R_2} X_{R_3} + k_{R_4}$$

k_{abs}	9.24E+09									
k^0_{prim}	1.18E+08									
k_{CH3R1}	8.28E+08									
X_{R1}	1.174	1.174	0	0	0	0	0	0	0	0
k^0_{sec}	5.11E+08									
$k_{CH2R1R2}$	2.66E+09									
X_{R1}	1.174	0.5510	1	0	0	0	0	0	0	0
X_{R2}	1.63	1.174	0.043	0	0	0	0	0	0	0
k^0_{tert}	1.99E+09									
$k_{CHR1R2R3}$	5.66E+09									
X_{R1}	1.12	0.5780	0	0	0	0	0	0	0	0
X_{R2}	1.12	1.174	0	0	0	0	0	0	0	0
X_{R3}	1.63	1.174	0	0	0	0	0	0	0	0
k_{OH}	1.00E+08									
# of -OH functional groups	1									
k_{COOH}	0.00E+00		kcoo-	0.00E+00		kI	0.00E+00		kBr	0.00E+00
# of -COOH functional groups	0			0			0			0

Group contribution factor, X	
$-CH_3$	1.120
$-CH_2$, >CH-, >C<	1.174
-(aromatic ring)	1.0000
-(C=C double bond)	1.0000
->C-(oxygenated)-	0.6810
-OH	0.5780
-O-	0.5510
-CO-	0.1540
-CHO	0.6016
=O	0.3600
-COO, -COOH	0.0430
-O-second	0.9450
-OCOR	0.0000
-RS5 (5 ring strain)	0.8601
-RS3 (3 ring strain)	0.0520

Group contribution factor, X	
-Cl	0.2032
-Br	0.3767
$-CF_3$	0.1018
$-CCl_3$	0.1123
$-CH_2Cl$, $-CHCl_2$, -CHCl-,	0.3671
-S-, -SS-, -HS-	2.400
-SO	0.4450
-CN	0.00292
$-NO_2$	0.0000
$-NH_2$, -NH-, -N<	1.630
-N-CO-	3.190
-N-NO	0.0105
$-N-NO_2$	0.1760
->P=O	0.1030
-O-P<-	0.00004

Group rate constant ($\cdot 10^8$ $M^{-1}s^{-1}$)	
k^0_{prim}	1.18
k^0_{sec}	5.11
k^0_{tert}	19.9
k_{OH}	1.00
k_{COOH}	0.007

Figure SI-B2: Excerpt of the Excel sheet to predict $k_{^\bullet OH}$ values by GCM, considering the contribution of H-atom abstraction.

As previously pointed out, atenolol contains no alkene functionalities, which is why all the group contribution factors Y are set to zero in Figure SI-B3. Similar to H-atom abstraction where primary, secondary and tertiary carbon atoms are differentiated, hydroxyl radical addition to alkenes are differentiated depending on the number of substituents and the configuration.

OH Addition to Alkenes:

$$k_{\text{add-alkene}} = g\, k^{0}_{(\text{structure})-h}\, Y_{R_l}$$

$k_{\text{add-alkene}}$	0.00E+00
$k^0_{(HH>C=C<H)-1}$	1.00E+10
$k^0_{(HH>C=C<H)-2}$	1.01E+08
Y_{R1}	0
$k^0_{(HH>C=C<)-1}$	9.79E+10
$k^0_{(HH>C=C<)-2}$	3.16E+09
Y_{R1}	0
Y_{R2}	0
$k^0_{(H>C=C<H)(cis)}$	3.01E+10
Y_{R1}	0
Y_{R2}	0
$k^0_{(H>C=C<H)(trans)}$	5.21E+10
Y_{R1}	0
Y_{R2}	0
$k^0_{>C=C<}$	5.14E+11
Y_{R1}	0
Y_{R2}	0
Y_{R3}	0
Y_{R4}	0

Group contribution factor, Y	
-Alkane (-CH_3)	0.171
-CH_2	0.388
-CO-	0.6000
-CHO	0.6000
-COOH, -COOR	0.2340
-F	0.0000
-Cl	0.2100
-CN	0.1710

Group rate constant ($\cdot 10^{-9}$)	
$k^0_{(HH>C=C<H)-1}$	10.0
$k^0_{(HH>C=C<H)-2}$	0.10
$k^0_{(HH>C=C<)-1}$	97.9
$k^0_{(HH>C=C<)-2}$	3.16
$k^0_{(H>C=C<H)(cis)}$	30.1
$k^0_{(H>C=C<H)(trans)}$	52.1
$k^0_{>C=C<}$	514

Figure SI-B3: Excerpt of the Excel sheet to predict $k_{\bullet OH}$ values by GCM, considering the contribution of hydroxyl radical addition to alkenes.

For the hydroxyl radical addition to aromatic rings, different base structures (benzene, pyridine, furan, imidazole, triazine) and within these structures different substitution patterns are differentiated. For each base structure, different group contribution factors Z have to be used. Atenolol features a 1,4-substituted benzene ring. Since it has two substituents, the two group contributions factors of an alkyl chain ($Z = 1.00078$) and of an ether ($Z = 1.034$) are filled in the corresponding fields (see Figure SI-B4). This yields a contribution of 5.45×10^9 $M^{-1}s^{-1}$ for the aromatic ring. The formula takes into account that positions 2 and 6 ($k^0_{(1,4-benz)-2,6}$) and 3 and 5 ($k^0_{(1,4-benz)-3,5}$) are identical.

OH Addition to Aromatic Compounds:

$$k_{\text{add-aromatic}} = \sum n k^{0}_{(i\text{-name})\text{-}j} Z_{\mathrm{R}_m}$$

$k_{\text{add-aromatic}}$	5.45E+09
$k_{\text{benzene-related}}$	5.45E+09
$k^{0}_{\text{(1-benz)-2,6}}$	1.02E+09
$k^{0}_{\text{(1-benz)-3,5}}$	1.29E+09
$k^{0}_{\text{(1-benz)-4}}$	9.14E+08
Z_{R1}	0
$k^{0}_{\text{(1,2-benz)-3,6}}$	1.78E+09
$k^{0}_{\text{(1,2-benz)-4,5}}$	7.06E+08
Z_{R1}	0
Z_{R2}	0
$k^{0}_{\text{(1,3-benz)-2}}$	9.89E+08
$k^{0}_{\text{(1,3-benz)-4,6}}$	1.70E+09
$k^{0}_{\text{(1,3-benz)-5}}$	1.91E+09
Z_{R1}	0
Z_{R2}	0
$k^{0}_{\text{(1,4-benz)-2,6}}$	7.13E+08
$k^{0}_{\text{(1,4-benz)-3,5}}$	1.92E+09
Z_{R1}	1.034
Z_{R2}	1.001
$k^{0}_{\text{(1,2,3-benz)-4,6}}$	2.15E+09
$k^{0}_{\text{(1,2,3-benz)-5}}$	1.64E+09
Z_{R1}	0
Z_{R2}	0
Z_{R3}	0

Group contribution factor, Z (benzene)	
-Alk	1.001
-OH	1.269
-O-	1.034
-CHO	0.6718
-COOH	0.6797
-CO-	0.9813
-CONH2	0.8422
-F	0.9726
-Cl	0.9781
-Br	0.8784
-I	0.8211
-NH-	1.105
-NH-CO-	0.8553
-NH2	1.481
-CN	0.4111
-NO2	0.4052
-SO3H	0.3733
-SO	0.6560

Group rate constant ($\cdot 10^{-9}$ $M^{-1}s^{-1}$) (benzene)	
$k^{0}_{\text{(1-benz)-2,6}}$	1.02
$k^{0}_{\text{(1-benz)-3,5}}$	1.29
$k^{0}_{\text{(1-benz)-4}}$	0.91
$k^{0}_{\text{(1,2-benz)-3,6}}$	1.78
$k^{0}_{\text{(1,2-benz)-4,5}}$	0.71
$k^{0}_{\text{(1,3-benz)-2}}$	0.99
$k^{0}_{\text{(1,3-benz)-4,6}}$	1.70
$k^{0}_{\text{(1,3-benz)-5}}$	1.91
$k^{0}_{\text{(1,4-benz)-2,6}}$	0.71
$k^{0}_{\text{(1,4-benz)-3,5}}$	1.92
$k^{0}_{\text{(1,2,3-benz)-4,6}}$	2.15
$k^{0}_{\text{(1,2,3-benz)-5}}$	1.64
$k^{0}_{\text{(1,2,4-benz)-3}}$	2.80
$k^{0}_{\text{(1,2,4-benz)-5}}$	0.31
$k^{0}_{\text{(1,2,4-benz)-6}}$	1.13
$k^{0}_{\text{(1,3,5-benz)-2,4,6}}$	1.68
$k^{0}_{\text{(1,2,3,4-benz)-5,6}}$	3.68
$k^{0}_{\text{(1,2,3,5-benz)-4,6}}$	2.80
$k^{0}_{\text{(1,2,4,5-benz)-3,6}}$	3.53
$k^{0}_{\text{(1,2,3,4,5-benz)-6}}$	7.06
$k^{0}_{\text{(1,2,3,4,5,6-benz)-1,2,3,4,5,6}}$	0.31

Figure SI-B4: Excerpt of the Excel sheet to predict $k_{\bullet OH}$ values by GCM, considering the contribution of hydroxyl radical addition to aromatic rings.

In the last step, addition to N-, S- and P-atoms are evaluated. The number of each functional group, here one secondary amine and one primary amide, are filled into the table (see Figure SI-B5). These heteroatom additions have a contribution of $2.01 \times 10^{8}\ \mathrm{M^{-1}s^{-1}}$. Summing up all four contributions results in a predicted $k_{\bullet OH}$ value of $1.49 \times 10^{10}\ \mathrm{M^{-1}s^{-1}}$.

OH Interaction with S-, N- and P-Atom Containing Compounds:

k_{int}	2.01E+08
k_{S-}	0.00E+00
# of -S-	0
k_{S-S-}	0.00E+00
# of -S-S-	0
k_{SO}	0.00E+00
# of -SO	0
k_{HS-}	0.00E+00
# of -HS-	0
k_{CN}	0.00E+00
# of -CN	0
k_{NO2}	0.00E+00
# of -NO2	0
k_{CO-NH2}	9.98E+07
# of -CO-NH2	1
k_{CO-NH-}	0.00E+00
# of -CO-NH-	0
$k_{CO-N<}$	0.00E+00
# of -CO-N<	0
k_{NH2}	0.00E+00
# of -NH2	0
k_{NH-}	1.01E+08
# of -NH-	1
$k_{N<}$	0.00E+00
# of -N<	0
$k_{N-CO-N-}$	0.00E+00
# of -N-CO-N-	0
$k_{P\equiv}$	0.00E+00
# of -P	0

Group rate constant ($\cdot 10^{-8}$ $M^{-1}s^{-1}$)	
k_{S-}	23.6
k_{S-S-}	36.7
k_{SO}	19.2
k_{HS-}	9.93
k_{CN}	0.0555
k_{NO2}	1.33
k_{CO-NH2}	0.998
k_{CO-NH-}	5.00
$k_{CO-N<}$	9.98
k_{NH2}	40.0
k_{NH-}	1.00
$k_{N<}$	35.3
$k_{N-CO-N-}$	0.00409
$k_{P\equiv}$	0.258

Figure SI-B5: Excerpt of the Excel sheet to predict $k_{\bullet OH}$ values by GCM, considering the contribution of reactions with sulfur, nitrogen or phosphorus-containing moieties.

SI-B1.3.2 Quantitative structure property relationship

In the quantitative structure-property relationship (QSPR) model, multiple linear regression was used to identify a linear relationship between $k_{^\bullet\mathrm{OH}}$ and molecular descriptors (DRAGON descriptors).[11] The final QSPR considers seven different DRAGON descriptors and the $k_{^\bullet\mathrm{OH}}$ value can be calculated based on Equation 0.3.

$$\begin{aligned} \log k_{^\bullet\mathrm{OH}} = 16.451 - 6.932 \times \mathrm{Me} + 0.159 \times \mathrm{nDB} - 0.679 \times \mathrm{CH2RX} + 0.401 \times \mathrm{NHAcc} \\ -0.460 \times \mathrm{Vindex} - 0.363 \times \mathrm{MATS2m} - 0.362 \times \mathrm{Mor27p} \end{aligned} \tag{0.3}$$

These seven DRAGON descriptors include the mean atomic Sanderson electronegativity (Me), and describes the tendency of a atom or functional group to attract electrons. For a molecule with high electronegativity, removing electrons requires a significantly high amount of energy, which makes electron transfer induced by hydroxyl radicals challenging.[11] The number of double bonds (nDB), the number of acceptor atoms for hydrogen bonds (nHAcc) and the number of primary alkyl halide substructures (nCH2RX) with X corresponding to Cl, Br or I, are considered as well. These three discrete descriptors were binarized in the model, meaning set to zero or one if such a functional moiety is present in the molecule or not. The inclusion of nDB in the QSPR can be rationalized by the addition of hydroxyl radicals to double bonds, while functional groups described by nHAcc can positively affected the H-atom abstraction by withdrawing electrons from the C-H bond. Similarly, halogens are electron withdrawing groups, making carbon atoms more electrophilic and hence less prone to attack by electrophilic hydroxyl radicals.[11] The descriptors Mor27p, MATS2m and Vindex are related to the topological structure of the molecule. Mor27p considers the polarizability, while MATS2m gives information of the distribution of molecular mass along the topological structure.[11]

Originally, these DRAGON descriptors could be predicted by the DRAGON software, Since this software has been discontinued, a combination of multiple other software for the prediction of the seven included descriptors was necessary. The descriptor MATS2m was calculated with the ChemoPy python package[12] which is part of ChemDes.[13] The other six descriptors (Me, nDB, CH2RX, nHAcc, Vindex and Mor27p) were calculated with alvaDesc,[14] whereby Mor27p required previous 3D structure generation, which was conducted with CORINA classic from the SMILES representation.[15–17]

SI-B1.4 Machine learning model - pySiRC

The machine learning model pySiRC for predicting $k_{^\bullet\mathrm{OH}}$ values was trained on 1374 organic contaminants and is based on molecular fingerprints, whereby two different representations were used. On one hand Morgan fingerprints, developed by H.L. Morgan in 1965,[18] which are based on a circular neighborhood approach, where each atom in a molecule is treated as a node, and its chemical environment (neighboring atoms and bonds) is iteratively expanded outward to create unique structural identifiers. The process starts with the assignment of an initial identifier to each atom, a so-called hash, which can be the atomic number, valence, charge or hybridization. The atom's local chemical environment is then expanded outward in concentric layers. The neighboring information of bonds and connected atoms is encoded into the hash and the atom's identifier is updated at each step. This process continues for a defined number of iterations. These hashed identifiers from all atoms are then combined into a fixed-length bit vector, whereby each position represents the presence or absence of a specific structural feature. The final fingerprint captures a detailed molecular connectivity information which is used as input for the machine learning models. In contrast to Morgan fingerprints, which generate unique descriptors for each molecule, Molecular ACCess Systems (MACCS) fingerprints use a standardized set of 166 structural features (e.g. aromatic rings, hydroxyl groups, halogens, carbonyls) resulting in a fixed-length bit string representation.[19] These two fingerprints were used in combination with three different machine learning (ML) algorithms, including Neural Network,[20] Random Forest[21] and XGBoost.[22] Applied to the two types of molecular fingerprints, this results in six ML models. This method is distinct from the GCM and QSPR models in that it does not rely on predefined mechanistic pathways or a small fixed set of descriptors. The molecular fingerprints are weighted differently based on their contribution to the prediction, enabling the model to emphasize the most relevant features for predicting the second-order rate constants for the reactions with $^\bullet$OH.[23,24]

SI-B1.5 Wastewater Treatment Plants

Samples were taken from secondary effluents of three Swiss wastewater treatment plants, WWTP Altenrhein, WWTP Neugut in Duebendorf and WWTP Werdhoelzli in Zurich. All three plants are equipped with an advanced treatment, encompassing ozonation followed by sand filtration at Neugut and Werdhoelzli, and ozonation followed by granular activated carbon filtration at Altenrhein. Samples from influent, after the primary and secondary clarifier, after ozonation (O_3) and after sand or granular activated carbon (GAC) filtration were collected from Monday, February 28 to Friday, March 4, 2022, as 24 h-composite samples during dry weather conditions for previous studies.[25,26] Besides conventional activated sludge treatment, WWTP Altenrhein is additionally equipped with a fixed-bed reactor, which treats one third of the total wastewater volume. Biologically treated wastewater from both processes is combined and flows through a sand filter before ozonation. WWTP Altenrhein treats its wastewater with a GAC filter after ozonation. A schematic overview of the three WWTPs including sampling locations and characteristics such as specific ozone doses, is provided in Figure SI-B6.

Sampling devices from MAXX (MAXX Mess- u. Probenahmetechnik GmbH, Germany) were pre-installed from the operators at all sampling locations for flow-proportional sampling, except for Neugut influent and Werdhoelzli after ozonation, where MAXX TP5 C Active autosamplers were positioned for time-proportional sampling.

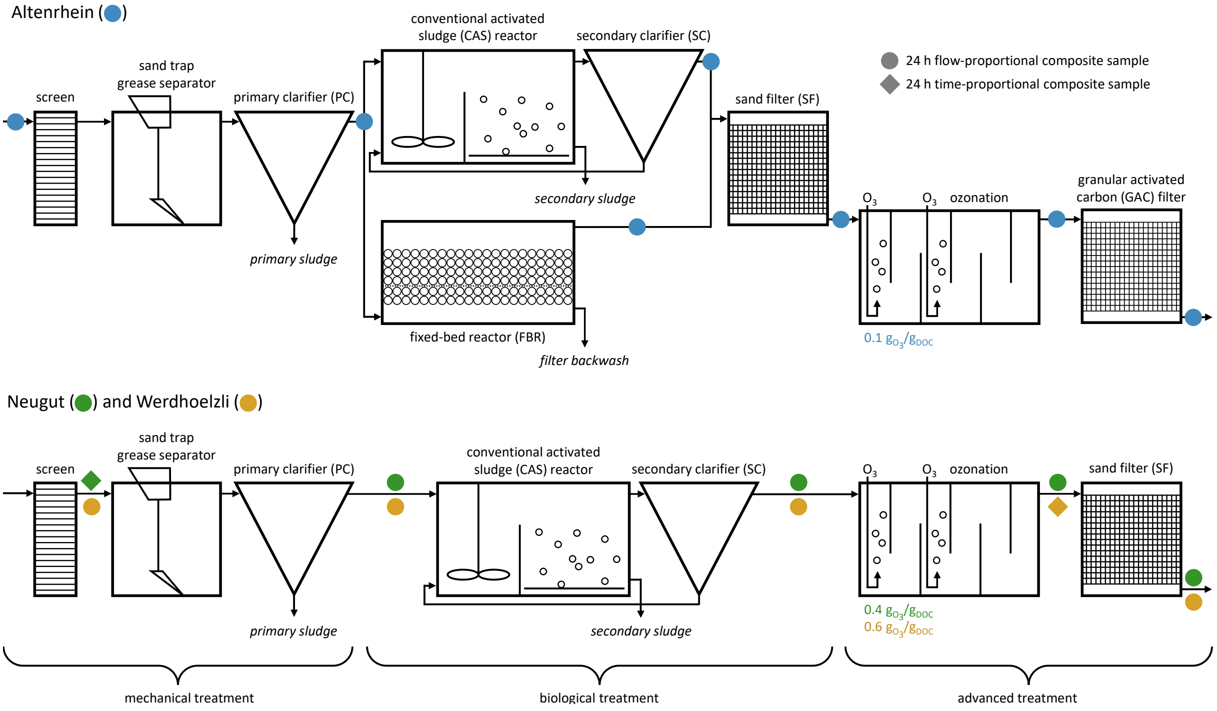

Figure SI-B6: Flow scheme of the WWTP Altenrhein (above), Neugut and Werdhoelzli (below), including sampling locations. At WWTP Altenrhein, conventional activated sludge treatment (2/3 of wastewater volume) and the fixed-bed reactor (1/3 of wastewater volume) are operated in parallel. The GAC filter was sampled after a run time of 22 700 bed volumes. The circles refer to 24 h flow proportional composite samples, while the diamonds indicate 24 h time-proportional sampling.

SI-B1.6 Sensitivity analysis

A sensitivity analysis was performed to understand which changes of the four input variables (k_{O_3}, $k_{^\bullet OH}$, O_3 exposure and $^\bullet$OH exposure) have the most significant effect on the removal in the WWTP. For this purpose, a derivative-based sensitivity analysis was chosen, which quantifies the influence of a parameter on the removal by evaluating the derivative of the removal with respect to that parameter. The derivative acts as a measure of how sensitive the removal is to small changes in the parameter. Combining the formula for the elimination of a pharmaceutical during ozonation (Equation 0.4[9]) and the formula for removal calculation (Equation 0.5) yields Equation 0.6, which is directly used for the sensitivity analysis.

$$-\ln\left(\frac{[c]}{[c]_0}\right) = k_{O_3}\int[O_3]\mathrm{d}t + k_{^\bullet OH}\int[^\bullet OH]\mathrm{d}t \tag{0.4}$$

$$R = \left(1 - \frac{[c]}{[c]_0}\right)\cdot 100 \tag{0.5}$$

$$R = \left(1 - e^{-k_{O_3}\int[O_3]\mathrm{d}t - k_{^\bullet OH}\int[^\bullet OH]\mathrm{d}t}\right)\cdot 100 \tag{0.6}$$

Taking the partial derivatives with respect to each of the four input parameters yields Equations 0.7 to 0.10.

$$\frac{\partial R}{\partial k_{O_3}} = 100\cdot e^{-k_{O_3}\int[O_3]dt - k_{^\bullet OH}\int[^\bullet OH]\mathrm{d}t}\cdot\int[O_3]\mathrm{d}t \tag{0.7}$$

$$\frac{\partial R}{\partial\int[O_3]\mathrm{d}t} = 100\cdot e^{-k_{O_3}\int[O_3]\mathrm{d}t - k_{^\bullet OH}\int[^\bullet OH]\mathrm{d}t}\cdot k_{O_3} \tag{0.8}$$

$$\frac{\partial R}{\partial k_{^\bullet OH}} = 100\cdot e^{-k_{O_3}\int[O_3]\mathrm{d}t - k_{^\bullet OH}\int[^\bullet OH]dt}\cdot\int[^\bullet OH]\mathrm{d}t \tag{0.9}$$

$$\frac{\partial R}{\partial\int[^\bullet OH]\mathrm{d}t} = 100\cdot e^{-k_{O_3}\int[O_3]\mathrm{d}t - k_{^\bullet OH}\int[^\bullet OH]\mathrm{d}t}\cdot k_{^\bullet OH} \tag{0.10}$$

SI-B2 k_{O_3} value derivation using multi-compound competition kinetics

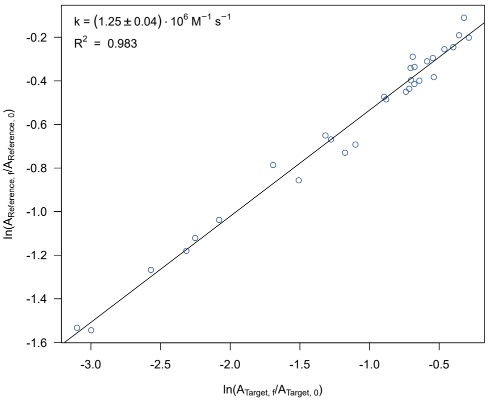

(a) Acemethacin - Carbamazepine

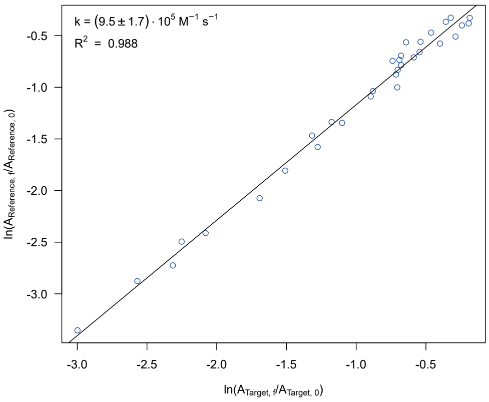

(b) Acemethacin - Sulfamethoxazole

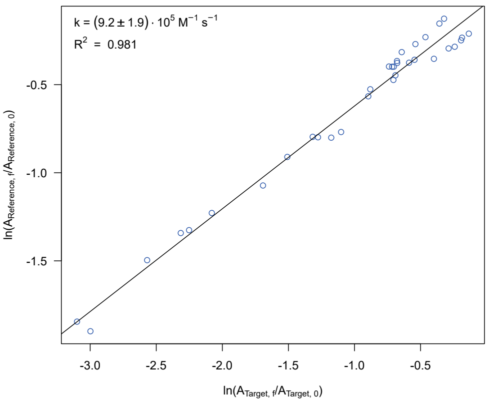

(c) Acemetacin - Trimethoprim

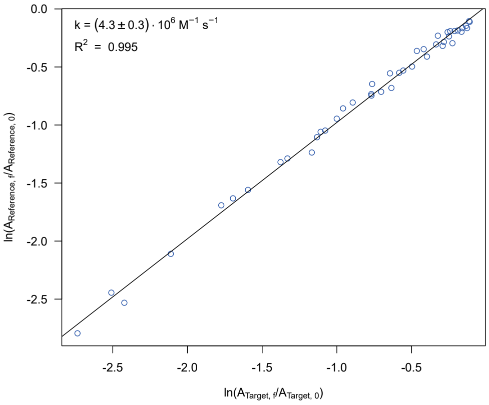

(d) Indomethacin - Dibromomethylparaben

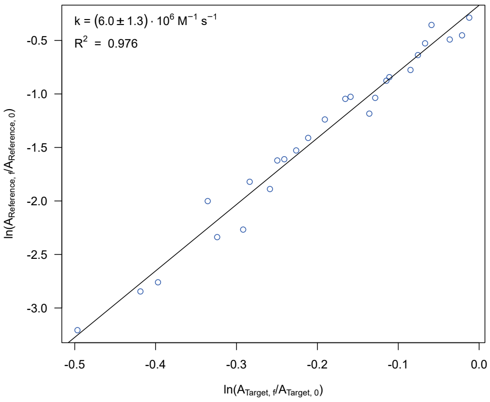

(e) Indomethacin - Triclosan

Figure SI-B7: Correlations between the natural logarithm of the relative residual peak areas of acemethacin (a - c) and indomethacin (d - e) with competitors upon ozonation at pH 7 (2 mM phosphate), 22 °C, and in presence of *t*BuOH (40 mM). The intercept was considered negligible (<10×slope) and the standard deviations of competitor k_{app,O_3} values were considered in the calculation of the standard deviations of the target k_{app,O_3} values. Note that the axes do not necessarily originate at zero.

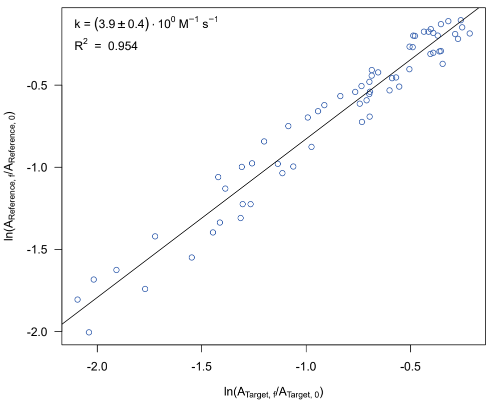

(a) Allopurinol - Alachlor

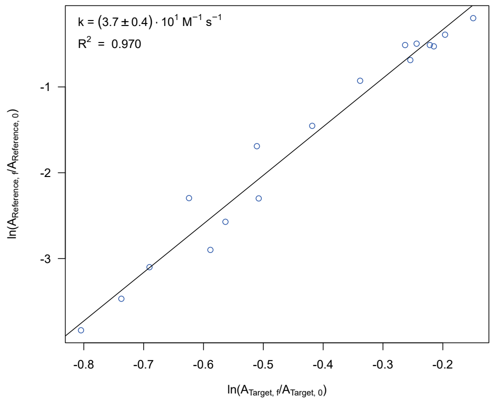

(b) Oxypurinol - Carbofuran

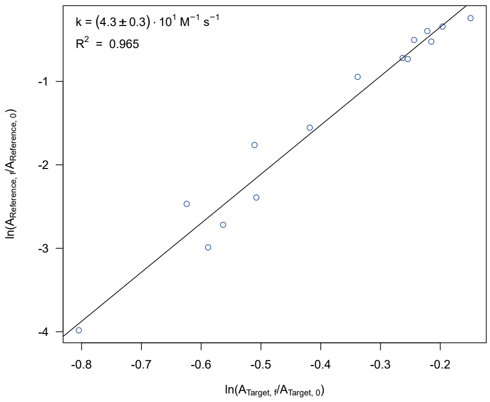

(c) Oxypurinol - N^4-Acetylsulfamethoxazole

Figure SI-B8: Correlations between the natural logarithm of the relative residual peak areas of allopurinol (a) and oxypurinol (b - c) with competitors upon ozonation at pH 7 (2 mM phosphate), 22 °C, and in presence of *t*BuOH (40 mM). The intercept was considered negligible (<10×slope) and the standard deviations of competitor $k_{\mathrm{app,O_3}}$ values were considered in the calculation of the standard deviations of the target $k_{\mathrm{app,O_3}}$ values. Note that the axes do not necessarily originate at zero.

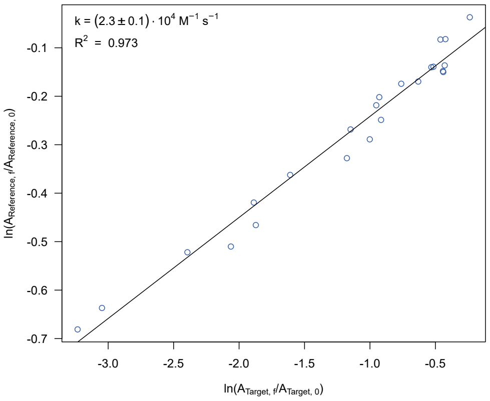

(a) Amisulpride - Penicillin G

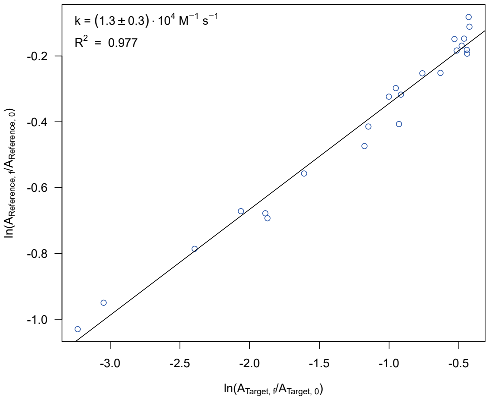

(b) Amisulpride - Tramadol

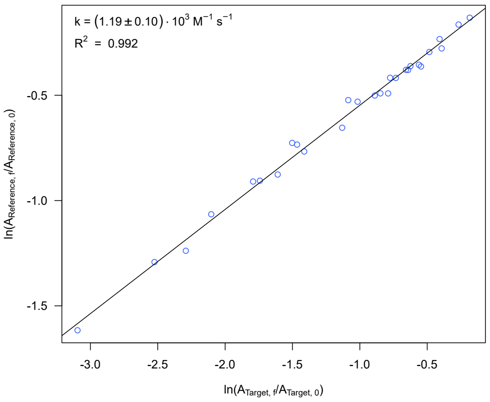

(c) Amisulpride-*N*-oxide - Bezafibrate

Figure SI-B9: Correlations between the natural logarithm of the relative residual peak areas of amisulpride (a - b) and amisulpride-*N*-oxide (c) with competitors upon ozonation at pH 7 (2 mM phosphate), 22 °C, and in presence of *t*BuOH (40 mM). The intercept was considered negligible (<10×slope) and the standard deviations of competitor $k_{\mathrm{app,O_3}}$ values were considered in the calculation of the standard deviations of the target $k_{\mathrm{app,O_3}}$ values. Note that the axes do not necessarily originate at zero.

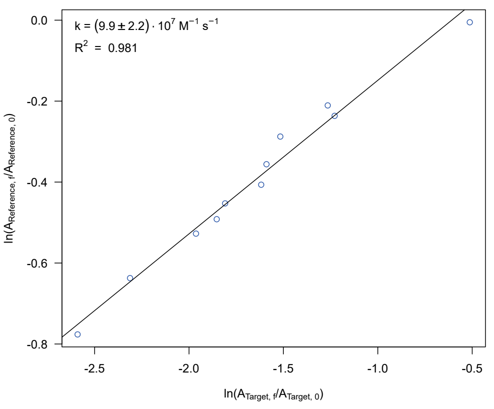

(a) Amlodipine - Triclosan

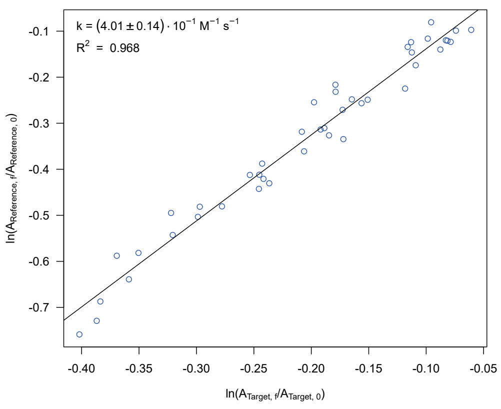

(b) *O*-Des(2-aminoethyl)-*O*-carboxymethyl-dehydroamlodipine - Diazepam

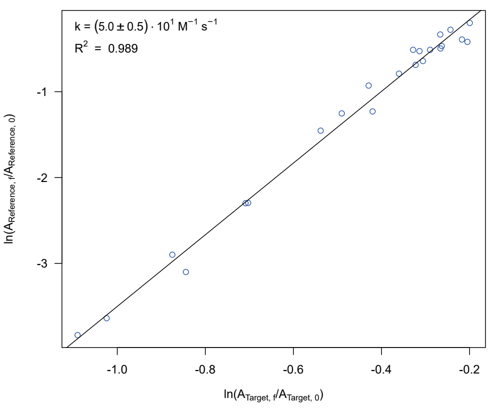

(c) Dehydroamlodipine - Carbofuran

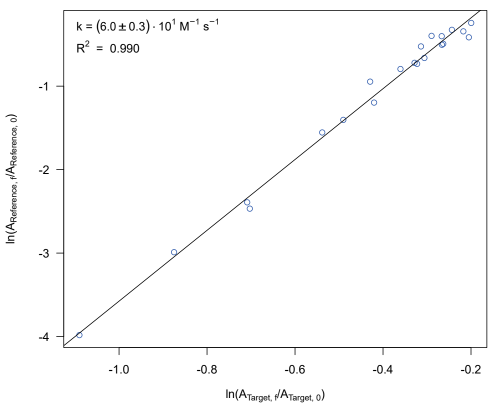

(d) Dehydroamlodipine - N^4-Acetylsulfamethoxazole

Figure SI-B10: Correlations between the natural logarithm of the relative residual peak areas of amlodipine (a), *O*-des(2-aminoethyl)-*O*-carboxymethyl-dehydroamlodipine (b) and dehydroamlodipine (c - d) with competitors upon ozonation at pH 7 (2 mM phosphate), 22 °C, and in presence of *t*BuOH (40 mM). The intercept was considered negligible (<10×slope) and the standard deviations of competitor $k_{\mathrm{app,O_3}}$ values were considered in the calculation of the standard deviations of the target $k_{\mathrm{app,O_3}}$ values. Note that the axes do not necessarily originate at zero.

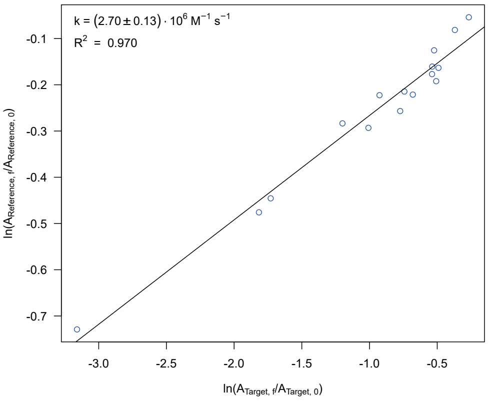

(a) Amoxicillin - Carbamazepine

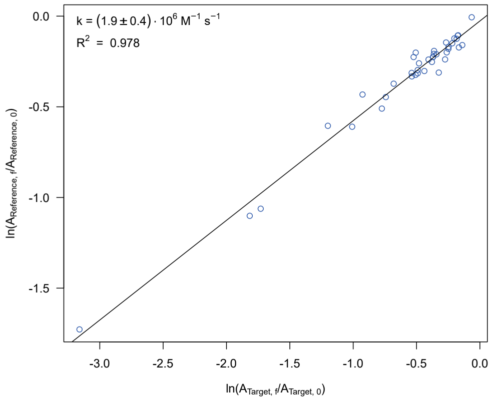

(b) Amoxicillin - Sulfamethoxazole

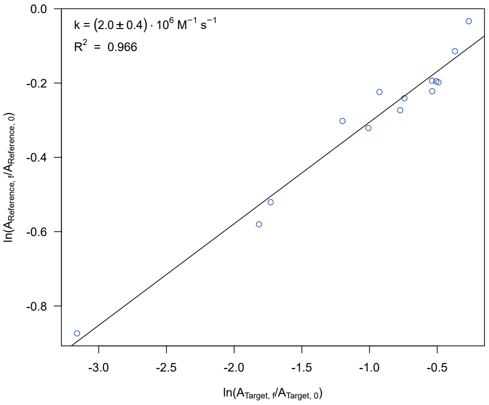

(c) Amoxicillin - Trimethoprim

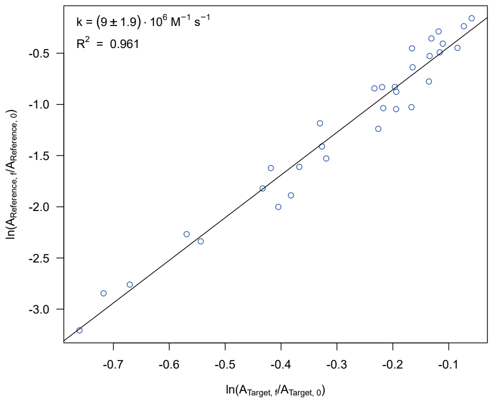

(d) Amoxicillin impurity E - Triclosan

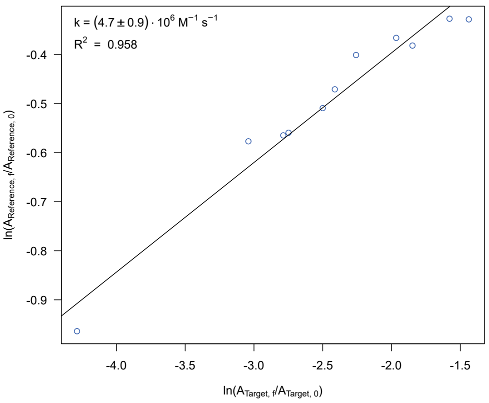

(e) Amoxicillin impurity F - Sulfamethoxazole

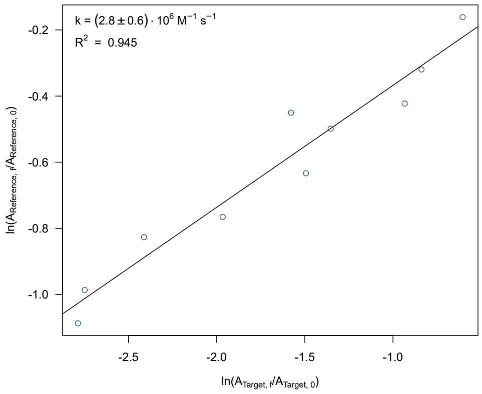

(f) Amoxicillin impurity F - Tylosin

Figure SI-B11: Correlations between the natural logarithm of the relative residual peak areas of amoxicillin (a - c), amoxicillin impurity E (d) and amoxicillin impurity F (e - f) with competitors upon ozonation at pH 7 (2 mM phosphate), 22 °C, and in presence of *t*BuOH (40 mM). The intercept was considered negligible (<10×slope) and the standard deviations of competitor k_{app,O_3} values were considered in the calculation of the standard deviations of the target k_{app,O_3} values. Note that the axes do not necessarily originate at zero.

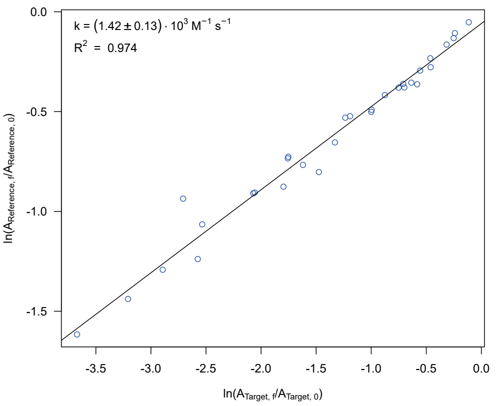

(a) Atenolol - Bezafibrate

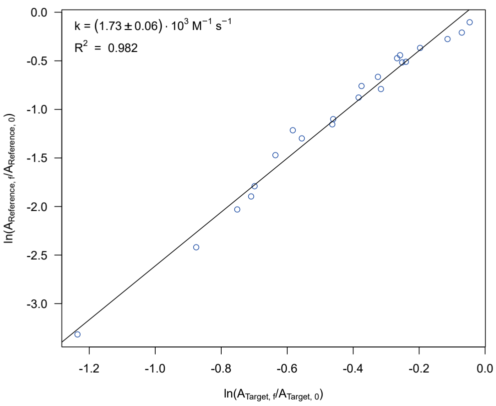

(b) Atenolol - Penicillin G

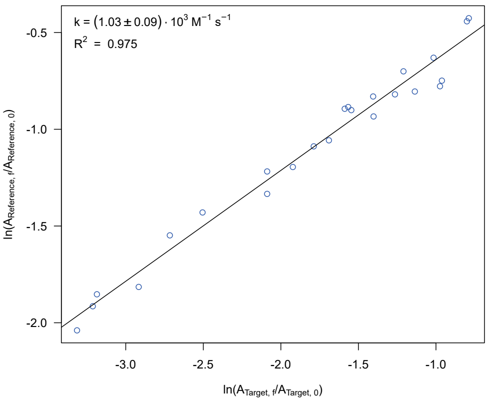

(c) Atenolol acid - Bezafibrate

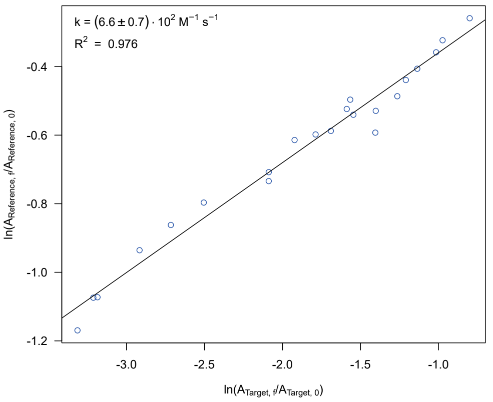

(d) Atenolol acid - Carbofuran

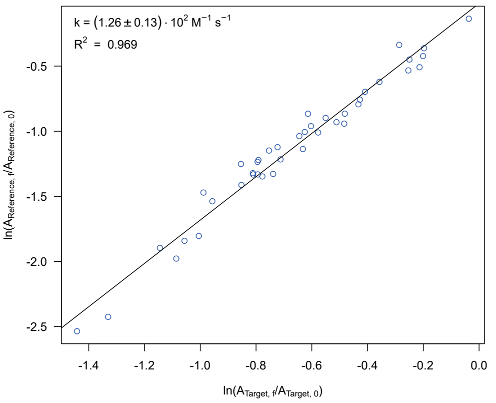

(e) Atenolol-desisopropyl - Carbofuran

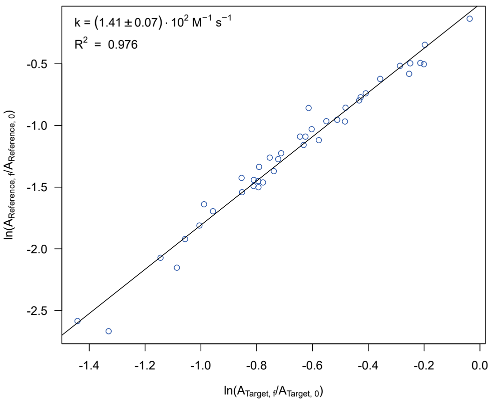

(f) Atenolol-desisopropyl - N^4-Acetylsulfamethoxazole

Figure SI-B12: Correlations between the natural logarithm of the relative residual peak areas of atenolol (a - b), atenolol acid (c - d) and atenolol-desisopropyl (e - f) with competitors upon ozonation at pH 7 (2 mM phosphate), 22 °C, and in presence of *t*BuOH (40 mM). The intercept was considered negligible (<10×slope) and the standard deviations of competitor k_{app,O_3} values were considered in the calculation of the standard deviations of the target k_{app,O_3} values. Note that the axes do not necessarily originate at zero.

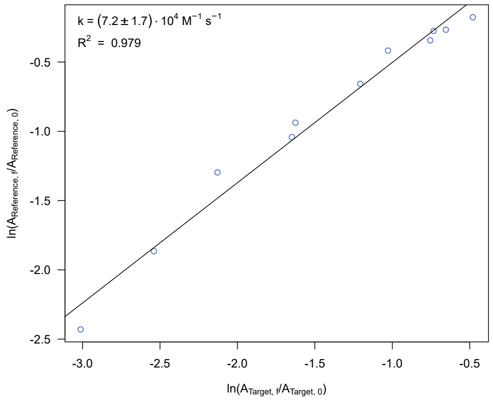

(a) Azithromycin - Roxithromycin

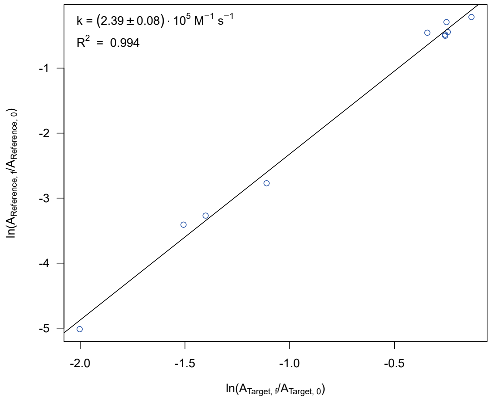

(b) Desosaminylazithromycin - Carbamazepine

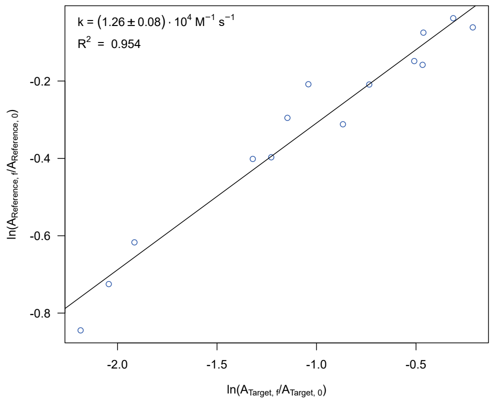

(c) *N*-Desmethylazithromycin - Penicillin G

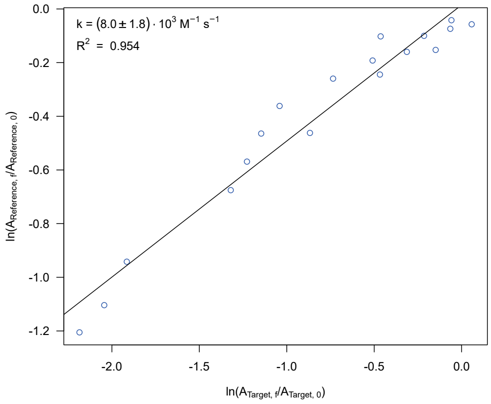

(d) *N*-Desmethylazithromycin - Tramadol

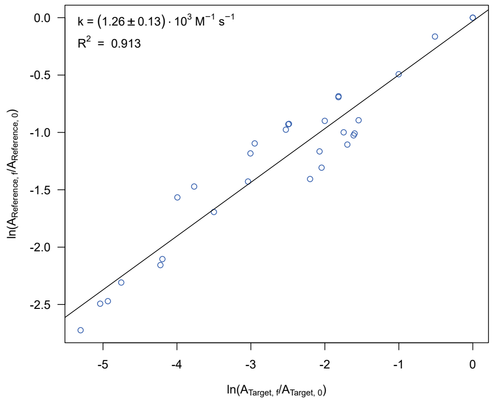

(e) Azithromycin-*N*-oxide - Bezafibrate

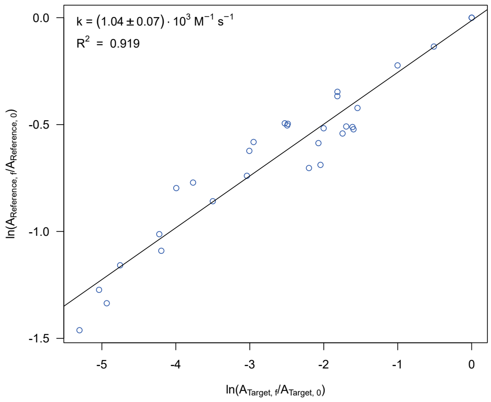

(f) Azithromycin-*N*-oxide - N^4-Acetylsulfamethoxazole

Figure SI-B13: Correlations between the natural logarithm of the relative residual peak areas of azithromycin (a), desosaminylazithromycin (b), *N*-desmethylazithromycin (c - d) and azithromycin-*N*-oxide (e - f) with competitors upon ozonation at pH 7 (2 mM phosphate), 22 °C, and in presence of *t*BuOH (40 mM). The intercept was considered negligible (<10×slope) and the standard deviations of competitor $k_{\mathrm{app,O_3}}$ values were considered in the calculation of the standard deviations of the target $k_{\mathrm{app,O_3}}$ values. Note that the axes do not necessarily originate at zero.

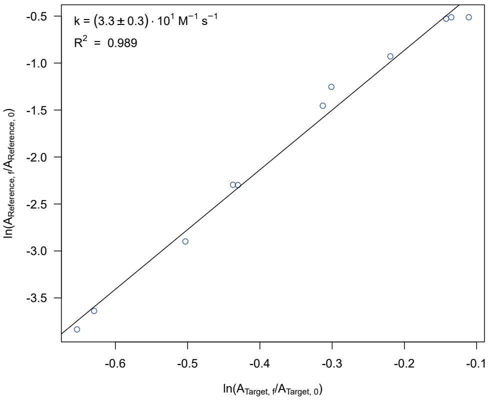

(a) Betamethasone - Carbofuran

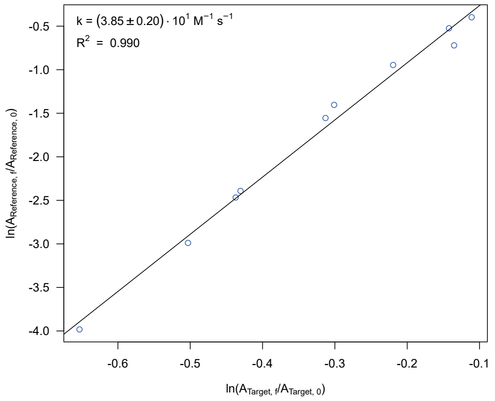

(b) Betamethasone - N^4-Acetylsulfamethoxazole

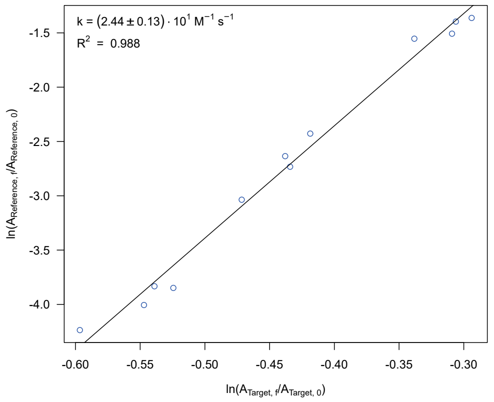

(c) Betamethasone-21-acetate - N^4-Acetylsulfamethoxazole

Figure SI-B14: Correlations between the natural logarithm of the relative residual peak areas of betamethasone (a - b) and betamethasone-21-acetate (c) with competitors upon ozonation at pH 7 (2 mM phosphate), 22 °C, and in presence of *t*BuOH (40 mM). The intercept was considered negligible (<10×slope) and the standard deviations of competitor k_{app,O_3} values were considered in the calculation of the standard deviations of the target k_{app,O_3} values. Note that the axes do not necessarily originate at zero.

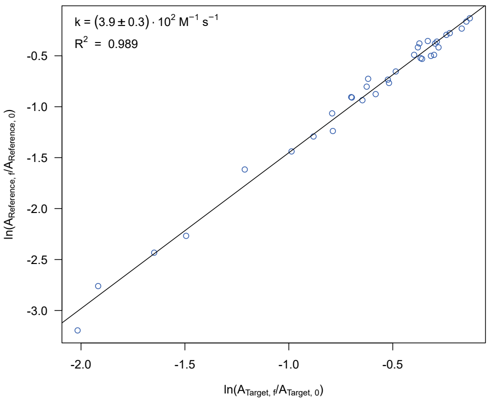

(a) Bupropion - Bezafibrate

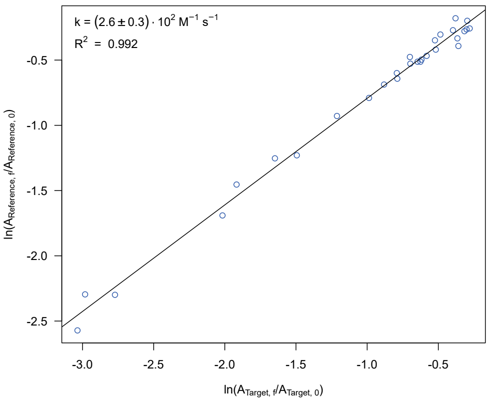

(b) Bupropion - Carbofuran

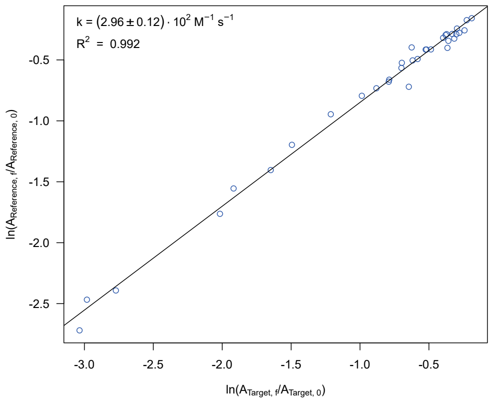

(c) Bupropion - N^4-Acetylsulfamethoxazole

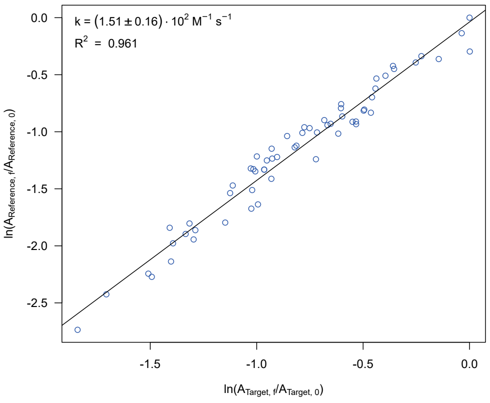

(d) Dihydrobupropion - Carbofuran

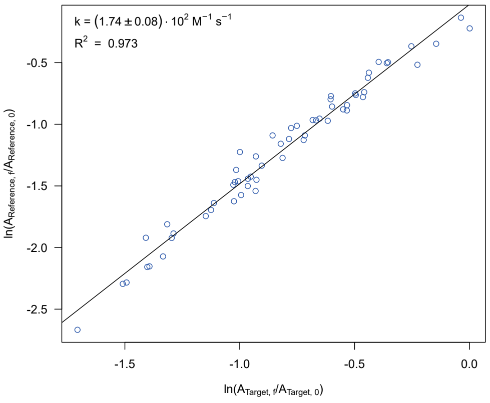

(e) Dihydrobupropion - N^4-Acetylsulfamethoxazole

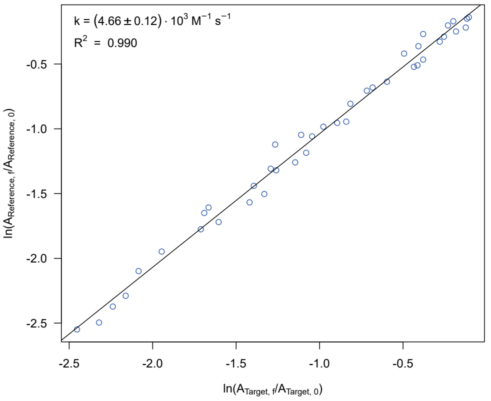

(f) Hydroxybupropion - Penicillin G

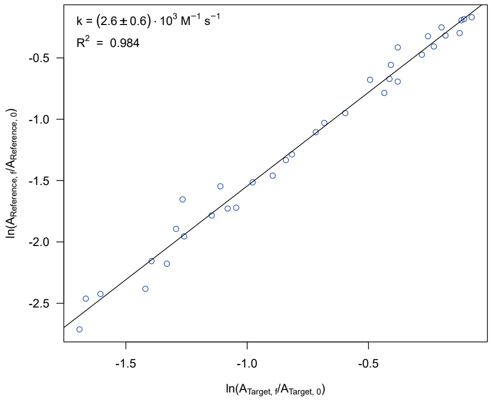

(g) Hydroxybupropion - Tramadol

Figure SI-B14: Correlations between the natural logarithm of the relative residual peak areas of bupropion (a - c), dihydrobupropion (d - e) and hydroxybupropion (f - g) with competitors upon ozonation at pH 7 (2 mM phosphate), 22 °C, and in presence of *t*BuOH (40 mM). The intercept was considered negligible (<10×slope) and the standard deviations of competitor $k_{\mathrm{app,O_3}}$ values were considered in the calculation of the standard deviations of the target $k_{\mathrm{app,O_3}}$ values. Note that the axes do not necessarily originate at zero.

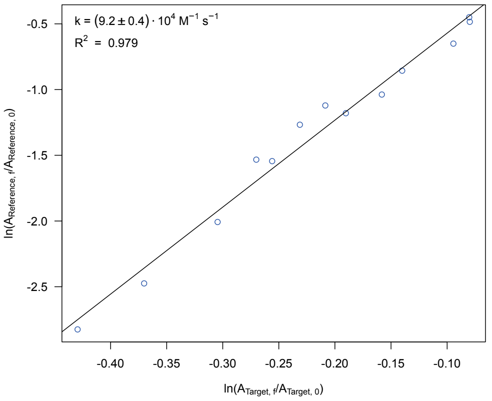

(h) Emtricitabine - Carbamazepine

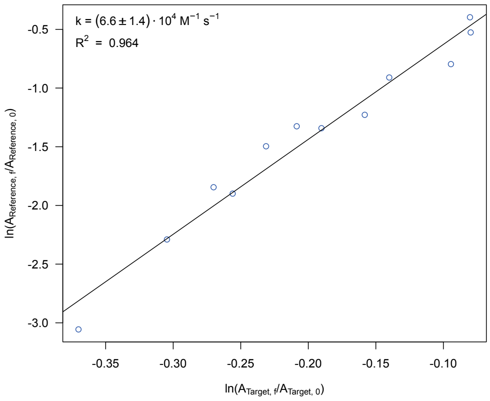

(i) Emtricitabine - Trimethoprim

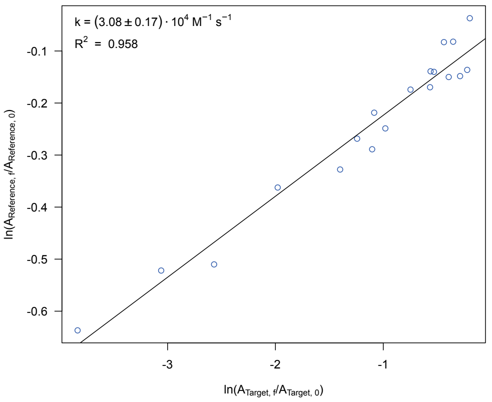

(j) 5-Fluorocytosine - Penicillin G

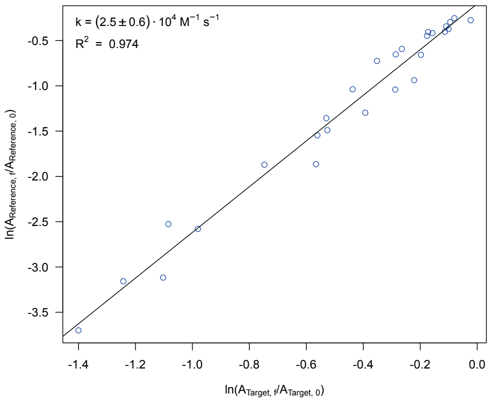

(k) 5-Fluorocytosine - Roxithromycin

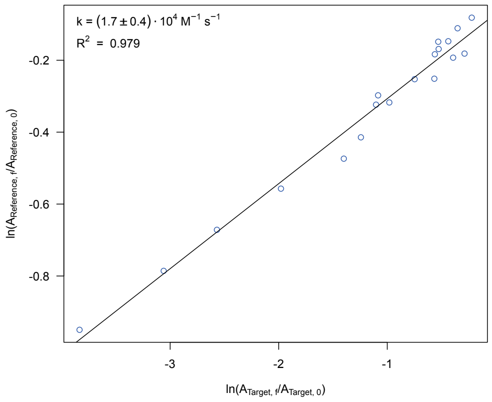

(l) 5-Fluorocytosine - Tramadol

Figure SI-B15: Correlations between the natural logarithm of the relative residual peak areas of emtricitabine (a - b) and 5-fluorocytosine (c - e) with competitors upon ozonation at pH 7 (2 mM phosphate), 22 °C, and in presence of *t*BuOH (40 mM). The intercept was considered negligible (<10×slope) and the standard deviations of competitor $k_{\mathrm{app,O_3}}$ values were considered in the calculation of the standard deviations of the target $k_{\mathrm{app,O_3}}$ values. Note that the axes do not necessarily originate at zero.

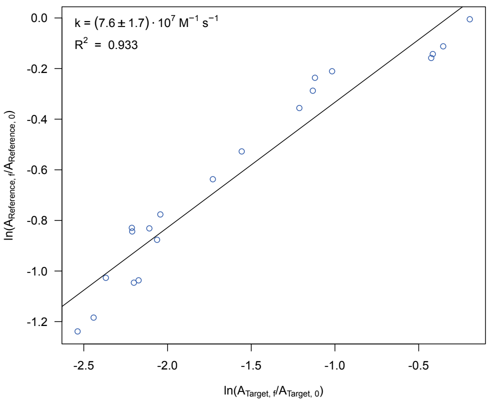

(a) Iminostilbene -Triclosan

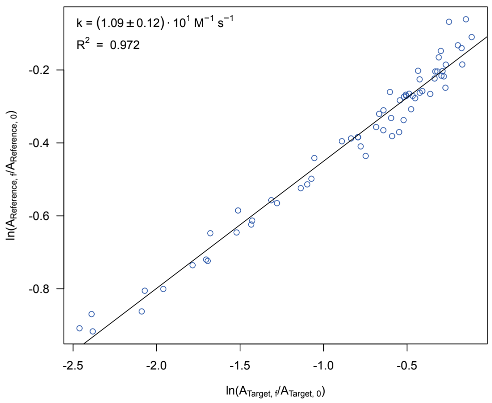

(b) Licarbazepine - Alachlor

Figure SI-B16: Correlations between the natural logarithm of the relative residual peak areas of iminostilbene (a) and licarbazepine (b) with competitors upon ozonation at pH 7 (2 mM phosphate), 22 °C, and in presence of *t*BuOH (40 mM). The intercept was considered negligible (<10×slope) and the standard deviations of competitor $k_{\mathrm{app,O_3}}$ values were considered in the calculation of the standard deviations of the target $k_{\mathrm{app,O_3}}$ values. Note that the axes do not necessarily originate at zero.

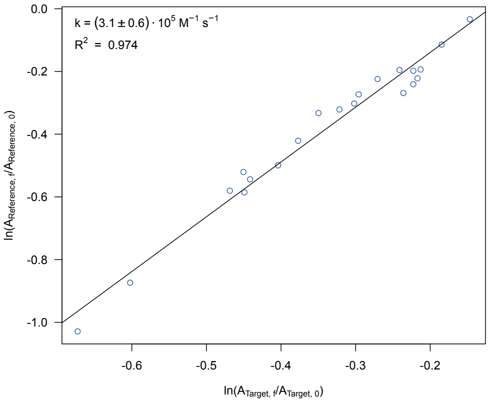

(a) Cetirizine - Trimethoprim

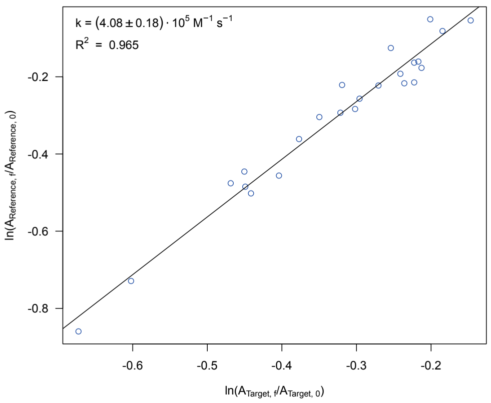

(b) Cetirizine - Carbamazepine

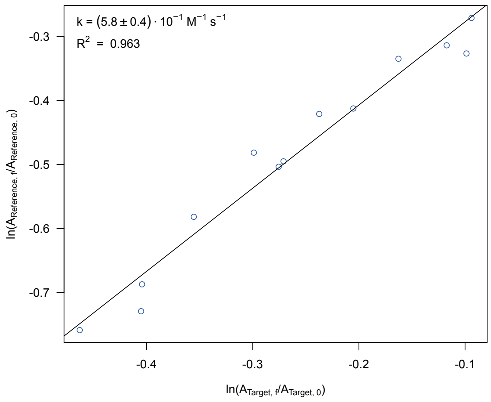

(c) 4-Chlorobenzophenone - Diazepam

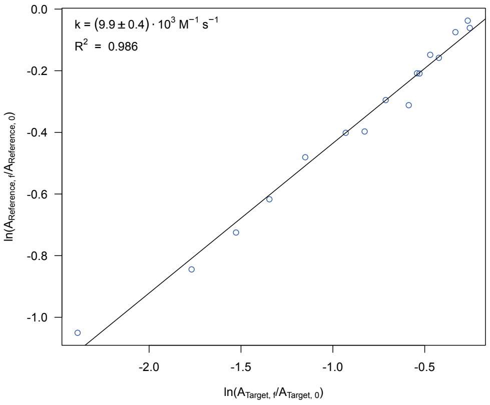

(d) 1-(4-Chlorobenzhydryl)piperazine - Penicillin G

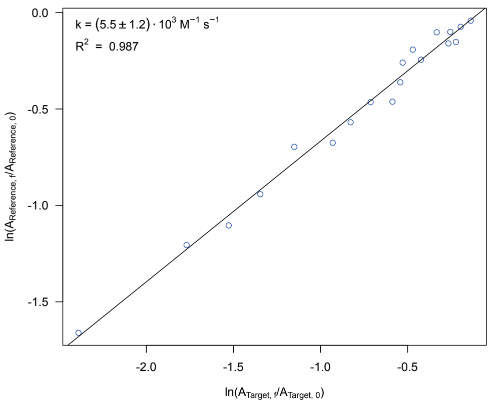

(e) 1-(4-Chlorobenzhydryl)piperazine - Tramadol

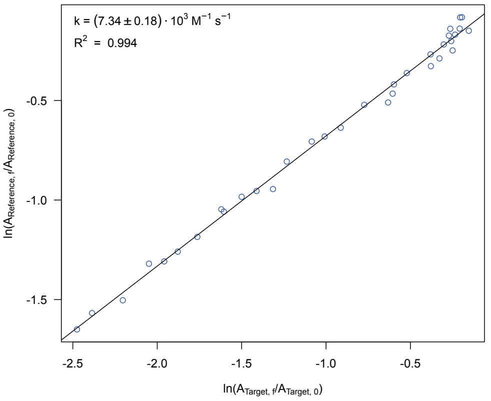

(f) Cetirizine-*N*-oxide - Penicillin G

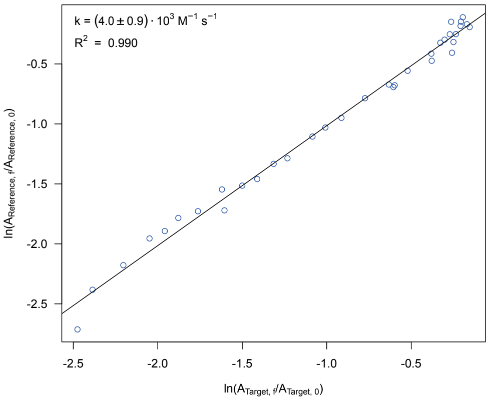

(g) Cetirizine-*N*-oxide - Tramadol

Figure SI-B16: Correlations between the natural logarithm of the relative residual peak areas of cetirizine (a - b), 4-chlorobenzophenone (c), 1-(4-chlorobenzhydryl)piperazine (d - e) and cetirizine-*N*-oxide (f - g) with competitors upon ozonation at pH 7 (2 mM phosphate), 22 °C, and in presence of *t*BuOH (40 mM). The intercept was considered negligible (<10×slope) and the standard deviations of competitor k_{app,O_3} values were considered in the calculation of the standard deviations of the target k_{app,O_3} values. Note that the axes do not necessarily originate at zero.

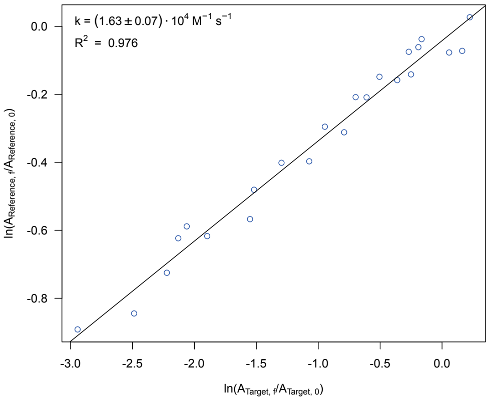

(h) Citalopram - Penicillin G

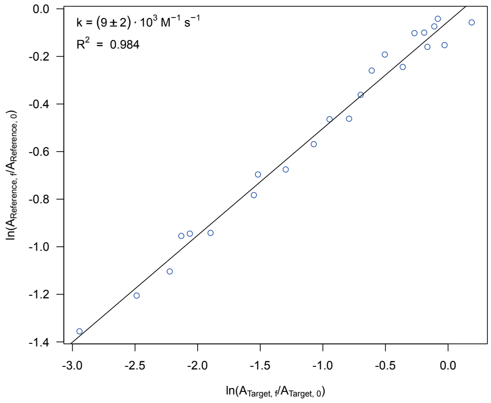

(i) Citalopram - Tramadol

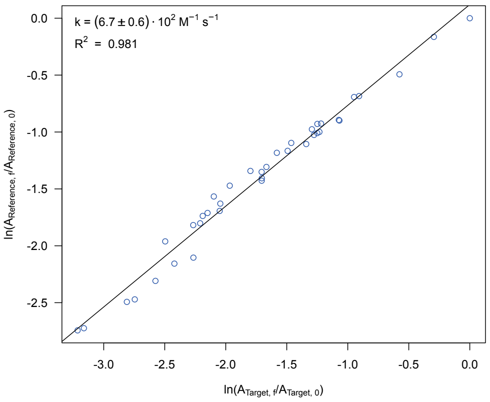

(j) *N*-Desmethylcitalopram - Bezafibrate

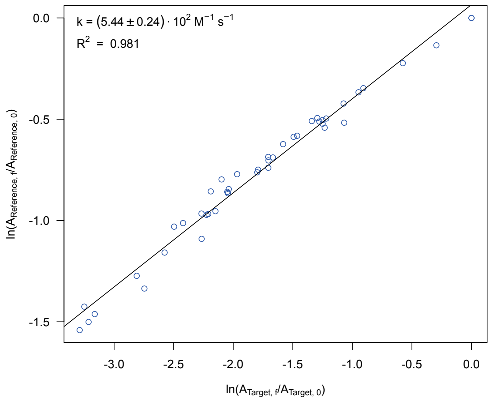

(k) *N*-Desmethylcitalopram - N^4-Acetylsulfamethoxazole

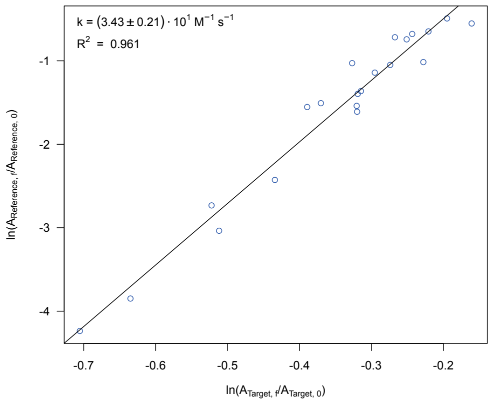

(l) *N*-Didesmethylcitalopram - N^4-Acetylsulfamethoxazole

Figure SI-B17: Correlations between the natural logarithm of the relative residual peak areas of citalopram (a - b), *N*-desmethylcitalopram (c - d) and *N*-didesmethylcitalopram (e) with competitors upon ozonation at pH 7 (2 mM phosphate), 22 °C, and in presence of *t*BuOH (40 mM). The intercept was considered negligible (<10×slope) and the standard deviations of competitor k_{app,O_3} values were considered in the calculation of the standard deviations of the target k_{app,O_3} values. Note that the axes do not necessarily originate at zero.

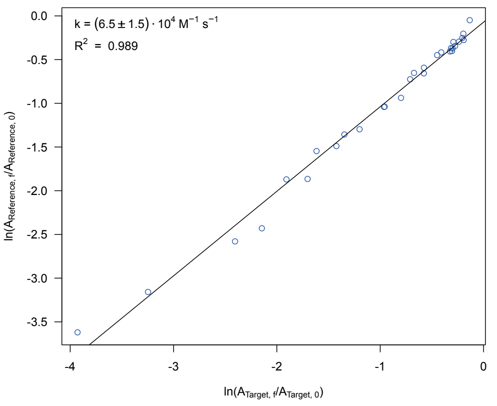

(a) Clarithromycin - Roxithromycin

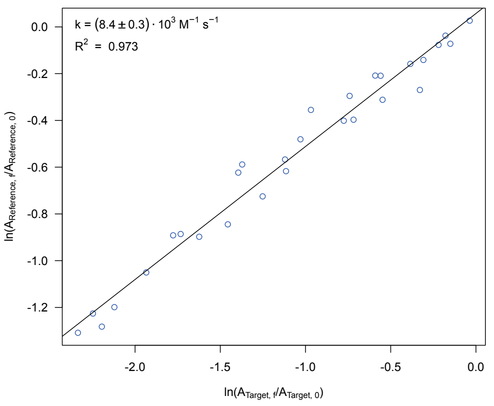

(b) *N*-Desmethylclarithromycin - Penicillin G

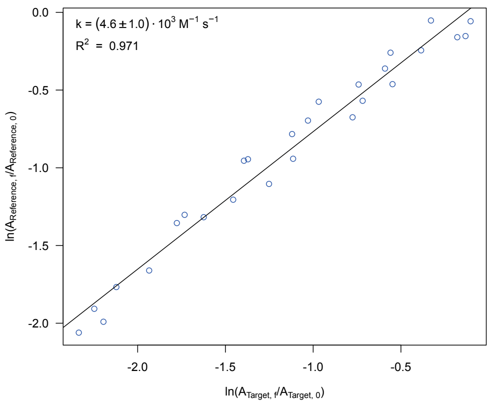

(c) *N*-Desmethylclarithromycin - Tramadol

Figure SI-B18: Correlations between the natural logarithm of the relative residual peak areas of clarithromycin (a) and *N*-desmethylclarithromycin (b - c) with competitors upon ozonation at pH 7 (2 mM phosphate), 22 °C, and in presence of *t*BuOH (40 mM). The intercept was considered negligible (<10×slope) and the standard deviations of competitor k_{app,O_3} values were considered in the calculation of the standard deviations of the target k_{app,O_3} values. Note that the axes do not necessarily originate at zero.

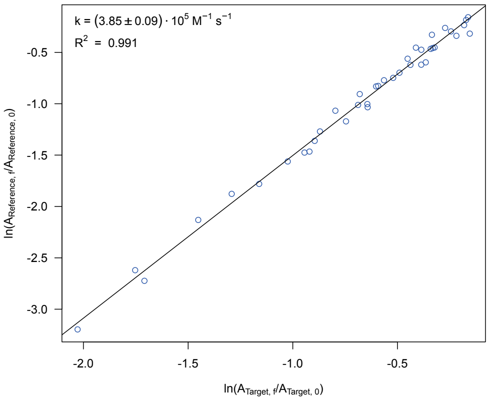

(a) Clopidogrel - Carbamazepine

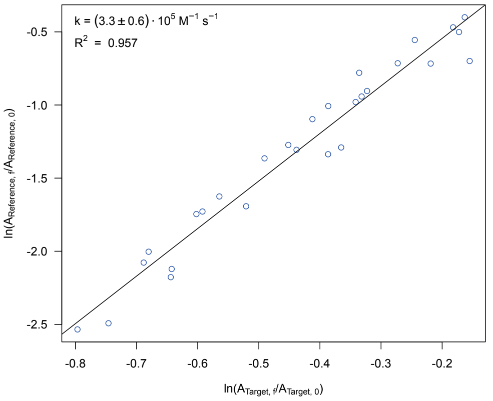

(b) Clopidogrel - Sulfamethoxazole

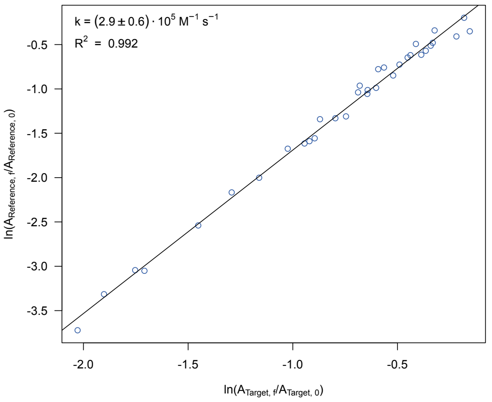

(c) Clopidogrel - Trimethoprim

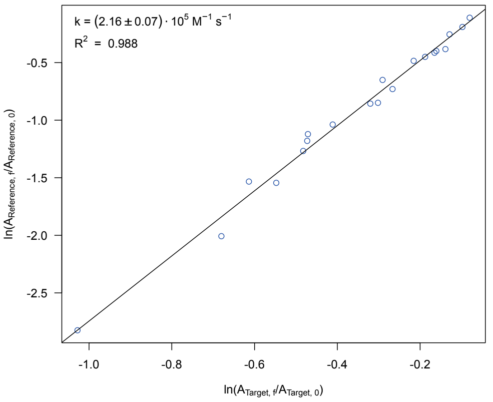

(d) Clopidogrel carboxylic acid - Carbamazepine

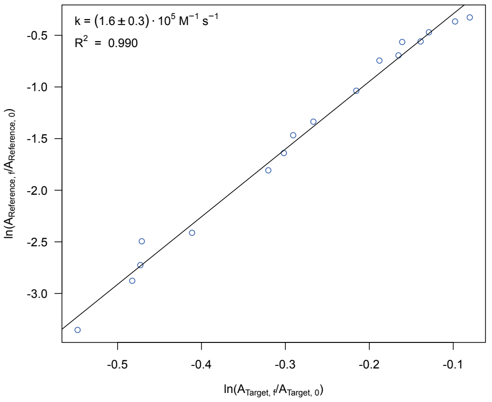

(e) Clopidogrel carboxylic acid - Sulfamethoxazole

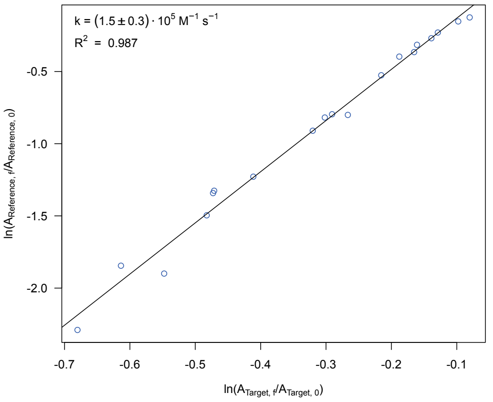

(f) Clopidogrel carboxylic acid - Trimethoprim

Figure SI-B19: Correlations between the natural logarithm of the relative residual peak areas of clopidogrel (a - c) and clopidogrel carboxylic acid (d - f) with competitors upon ozonation at pH 7 (2 mM phosphate), 22 °C, and in presence of *t*BuOH (40 mM). The intercept was considered negligible (<10×slope) and the standard deviations of competitor $k_{\mathrm{app,O_3}}$ values were considered in the calculation of the standard deviations of the target $k_{\mathrm{app,O_3}}$ values. Note that the axes do not necessarily originate at zero.

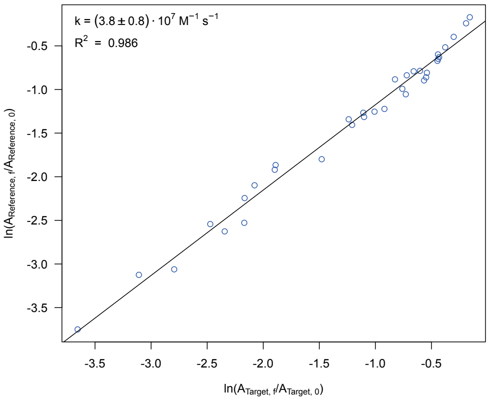

(a) Moprhine - Triclosan

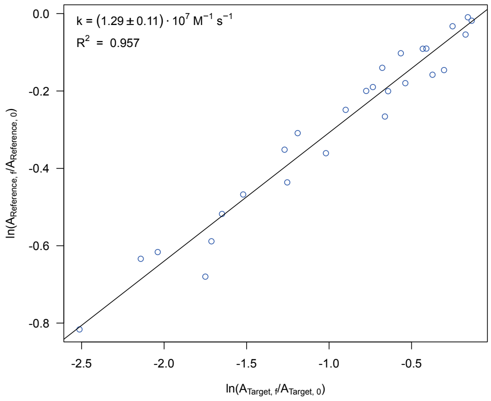

(b) 6-Acetylmorphine - Dibromomethylparaben

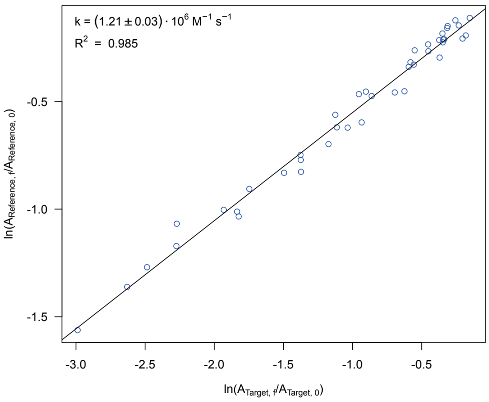

(c) Codeine - Carbamazepine

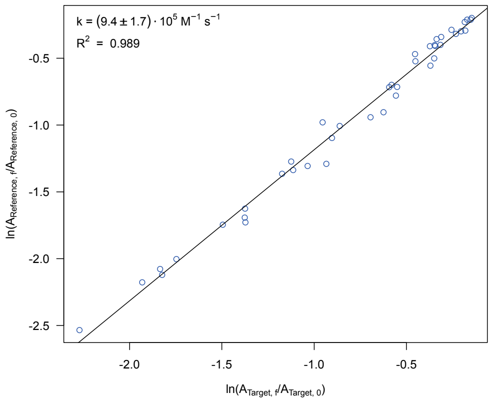

(d) Codeine - Sulfamethoxazole

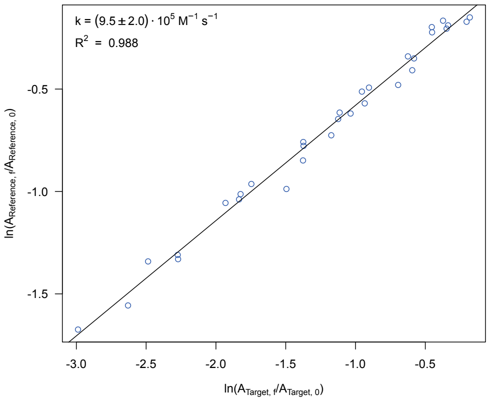

(e) Codeine - Trimethoprim

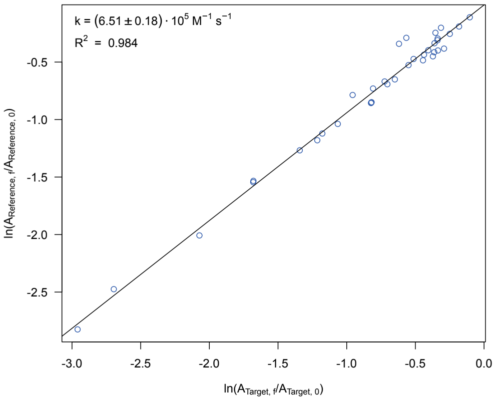

(f) Norcodeine - Carbamazepine

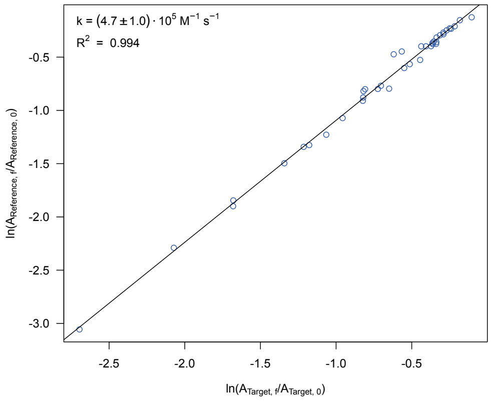

(g) Norcodeine - Trimethoprim

Figure SI-B19: Correlations between the natural logarithm of the relative residual peak areas of morphine (a), 6-acetylmorphine (b), codeine (c - e) and norcodeine (f - g) with competitors upon ozonation at pH 7 (2 mM phosphate), 22 °C, and in presence of *t*BuOH (40 mM). The intercept was considered negligible (<10×slope) and the standard deviations of competitor k_{app,O_3} values were considered in the calculation of the standard deviations of the target k_{app,O_3} values. Note that the axes do not necessarily originate at zero.

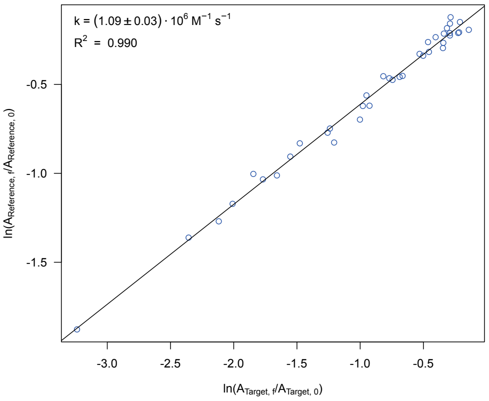

(h) Deprenyl - Carbamazepine

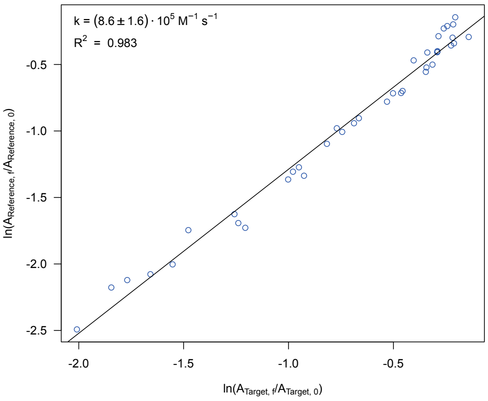

(i) Deprenyl - Sulfamethoxazole

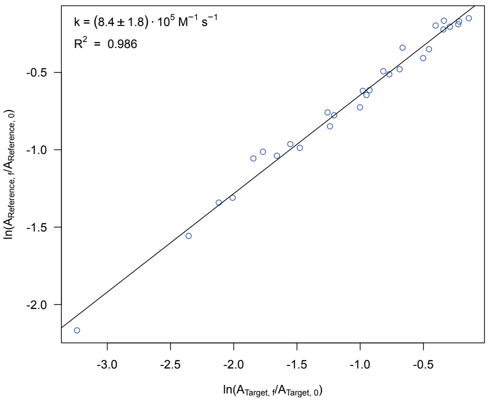

(j) Deprenyl - Trimethoprim

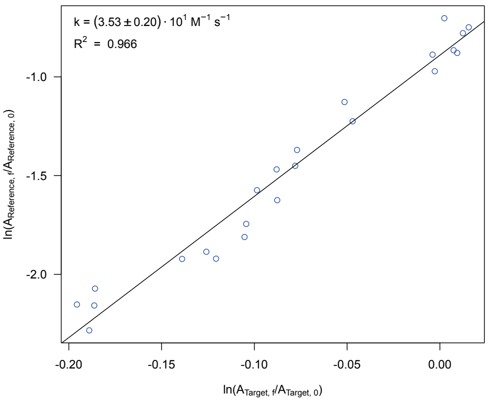

(k) Deprenyl-*N*-oxide - N^4-Acetylsulfamethoxazole

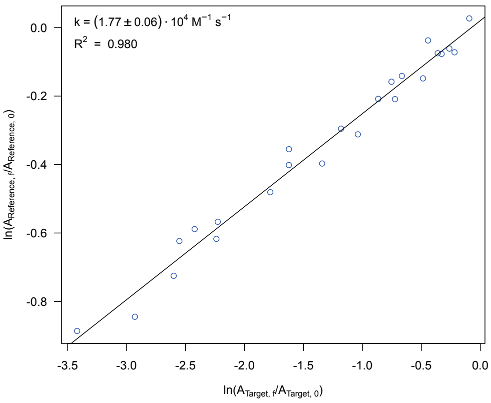

(l) Nordeprenyl - Penicillin G

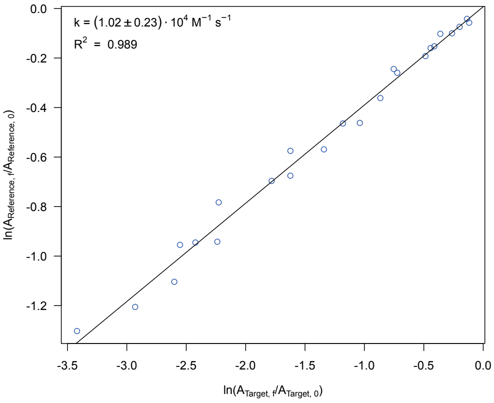

(m) Nordeprenyl - Tramadol

Figure SI-B20: Correlations between the natural logarithm of the relative residual peak areas of deprenyl (a - c), deprenyl-*N*-oxide (d) and nordeprenyl (e - f) with competitors upon ozonation at pH 7 (2 mM phosphate), 22 °C, and in presence of *t*BuOH (40 mM). The intercept was considered negligible (<10×slope) and the standard deviations of competitor k_{app,O_3} values were considered in the calculation of the standard deviations of the target k_{app,O_3} values. Note that the axes do not necessarily originate at zero.

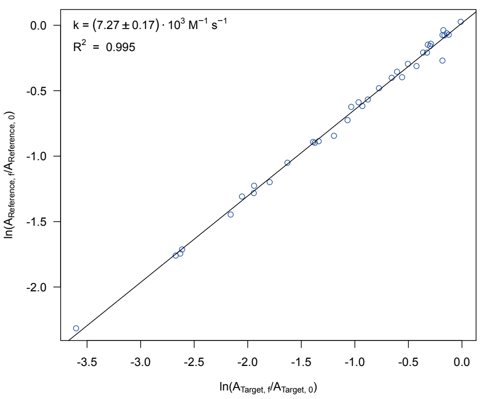

(a) Dextromethorphan - Penicillin G

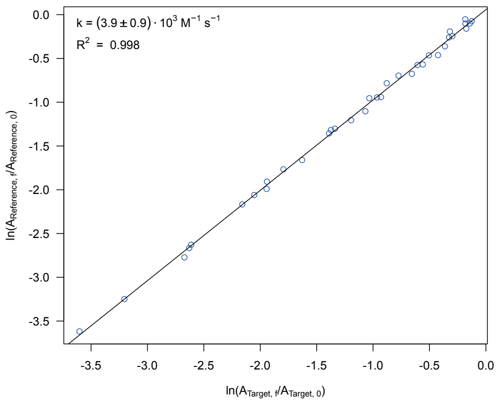

(b) Dextromethorphan - Tramadol

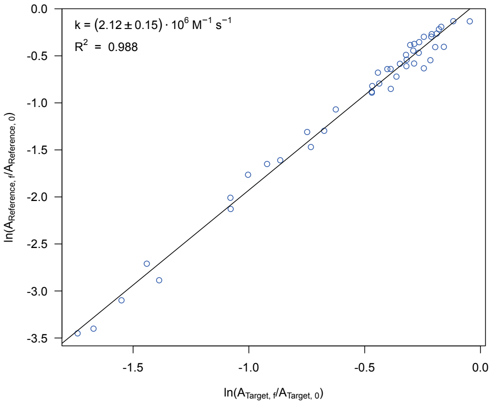

(c) Dextrorphan - Dibromomethylparaben

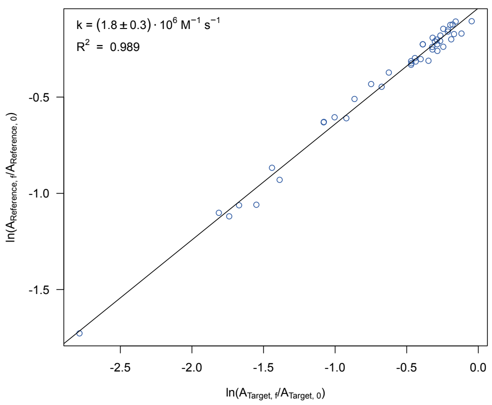

(d) Dextrorphan - Sulfamethoxazole

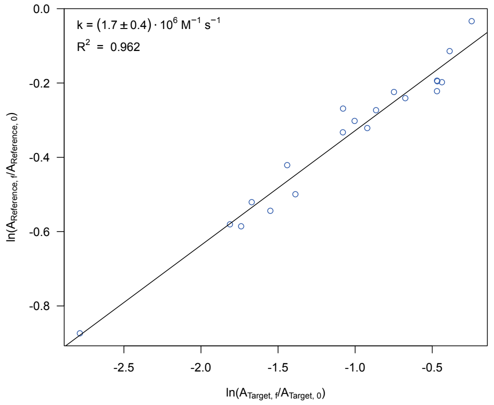

(e) Dextrorphan - Trimethoprim

Figure SI-B21: Correlations between the natural logarithm of the relative residual peak areas of dextromethorphan (a - b) and dextrorphan (c - e) with competitors upon ozonation at pH 7 (2 mM phosphate), 22 °C, and in presence of *t*BuOH (40 mM). The intercept was considered negligible (<10×slope) and the standard deviations of competitor $k_{\mathrm{app,O_3}}$ values were considered in the calculation of the standard deviations of the target $k_{\mathrm{app,O_3}}$ values. Note that the axes do not necessarily originate at zero.

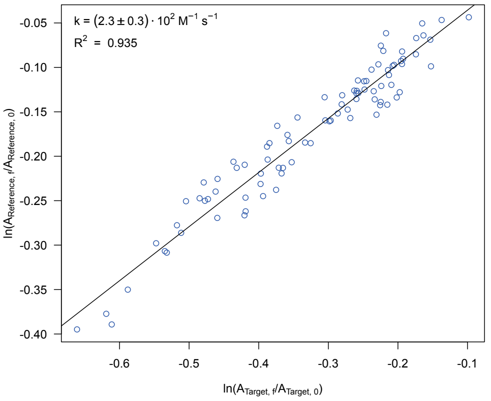

(a) Diatrizoate - Picloram

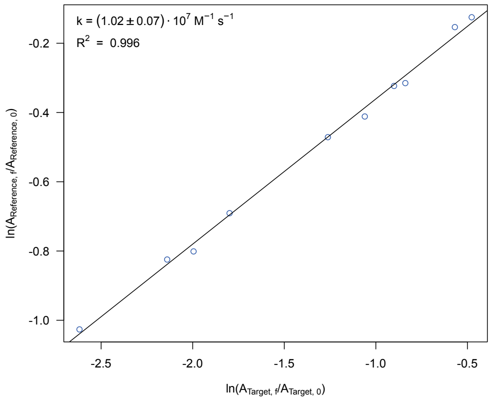

(b) 3,5-Diamino-2,4,6-triiodobenzoic acid - Dibromomethylparaben

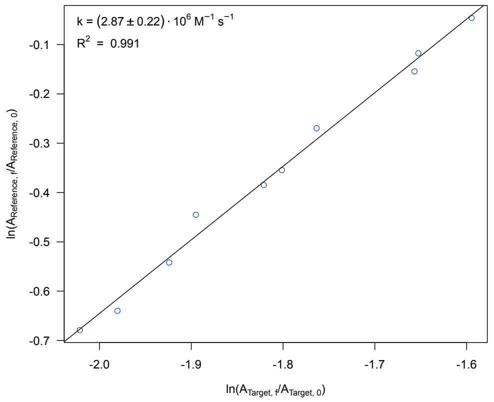

(c) 3,5-Diiodo-L-tyrosine - Dibromomethylparaben

Figure SI-B22: Correlations between the natural logarithm of the relative residual peak areas of diatrizoate (a), 3,5-siamino-2,4,6-triiodobenzoic acid (b) and 3,5-diiodo-L-tyrosine (c) with competitors upon ozonation at pH 7 (2 mM phosphate), 22 °C, and in presence of *t*BuOH (40 mM). The intercept was considered negligible (<10×slope) and the standard deviations of competitor $k_{\mathrm{app,O_3}}$ values were considered in the calculation of the standard deviations of the target $k_{\mathrm{app,O_3}}$ values. Note that the axes do not necessarily originate at zero.

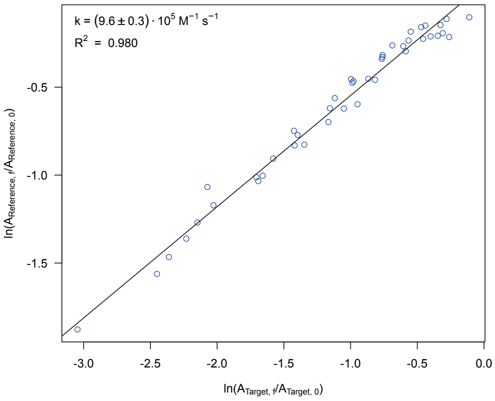

(a) Diclofenac - Carbamazepine

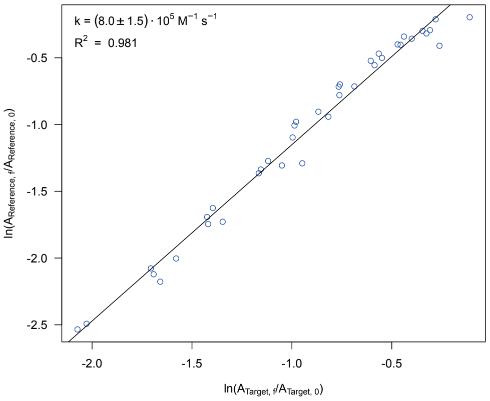

(b) Diclofenac - Sulfamethoxazole

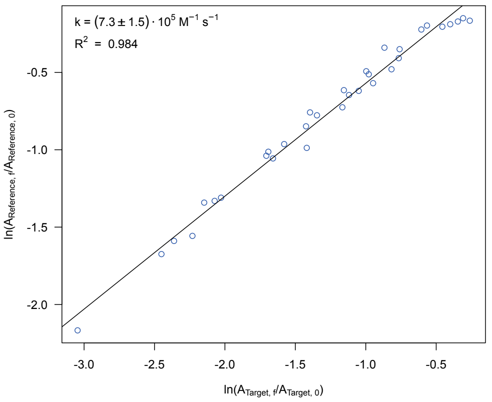

(c) Diclofenac - Trimethoprim

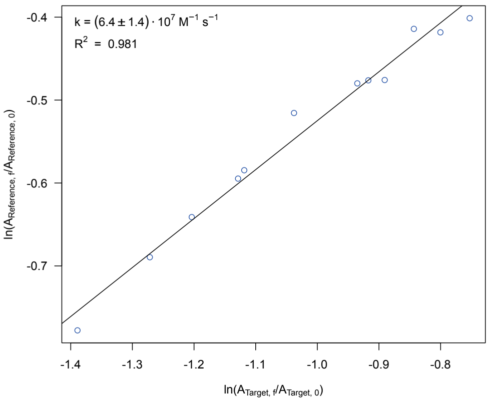

(d) 5-hydroxydiclofenac - Triclosan

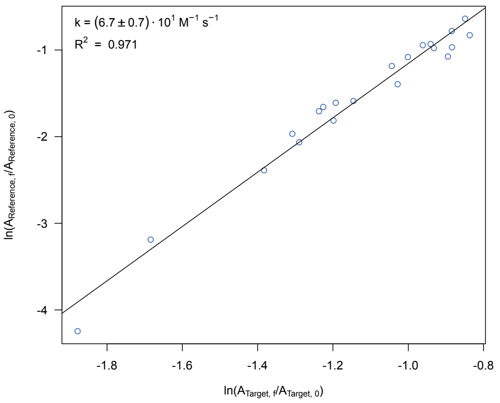

(e) 4'-hydroxydiclofenac - Carbofuran

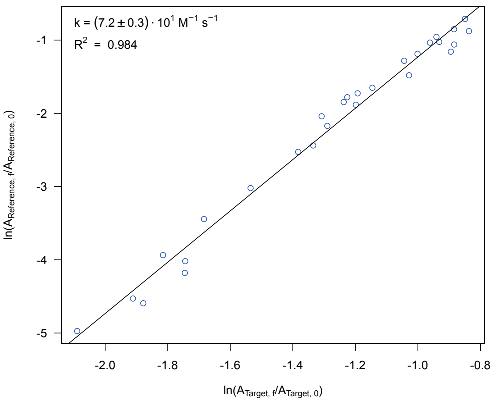

(f) 4'-hydroxydiclofenac - N^4-Acetylsulfamethoxazole

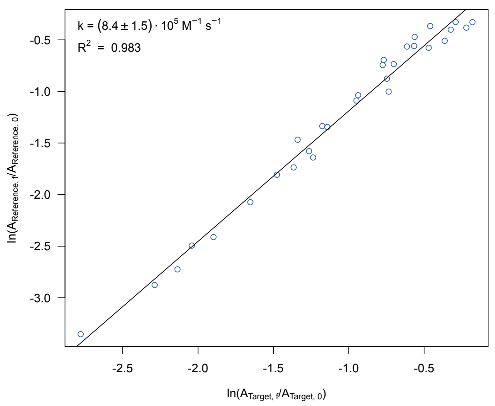

(g) Diclofenac carboxylic acid - Sulfamethoxazole

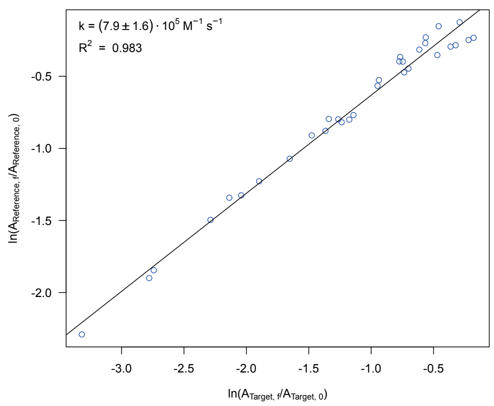

(h) Diclofenac carboxylic acid - Trimethoprim

Figure SI-B22: Correlations between the natural logarithm of the relative residual peak areas of diclofenac (a - c), 5-hydroxydiclofenac (d), 4'-hydroxydiclofenac (e - f) and diclofenac carboxylic acid (g -h) with competitors upon ozonation at pH 7 (2 mM phosphate), 22 °C, and in presence of *t*BuOH (40 mM). The intercept was considered negligible (<10×slope) and the standard deviations of competitor k_{app,O_3} values were considered in the calculation of the standard deviations of the target k_{app,O_3} values. Note that the axes do not necessarily originate at zero.

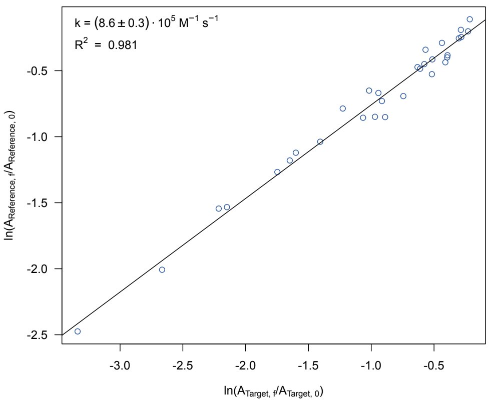

(i) Diltiazem - Carbamazepine

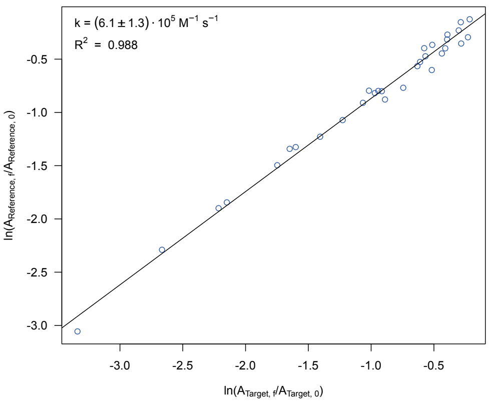

(j) Diltiazem - Trimethoprim

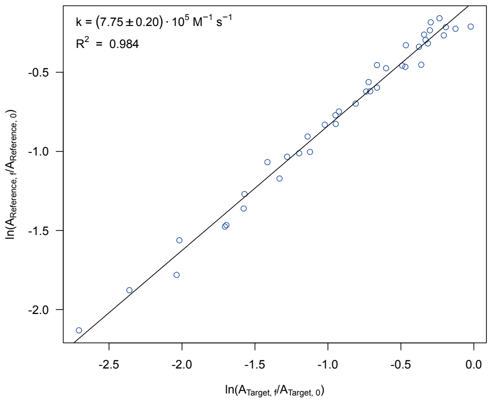

(k) Desacetyldiltiazem - Carbamazepine

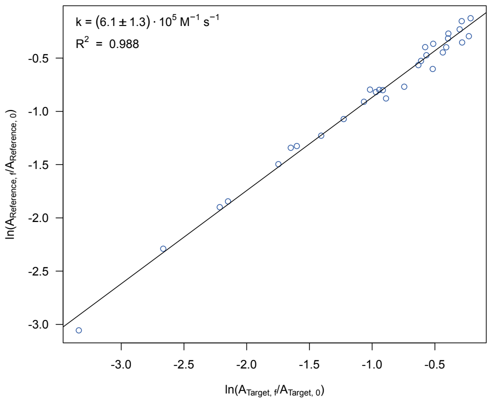

(l) Desacetyldiltiazem - Trimethoprim

Figure SI-B23: Correlations between the natural logarithm of the relative residual peak areas of diltiazem (a - b) and desacetyldiltiazem (c - d) with competitors upon ozonation at pH 7 (2 mM phosphate), 22 °C, and in presence of *t*BuOH (40 mM). The intercept was considered negligible (<10×slope) and the standard deviations of competitor $k_{\mathrm{app,O_3}}$ values were considered in the calculation of the standard deviations of the target $k_{\mathrm{app,O_3}}$ values. Note that the axes do not necessarily originate at zero.

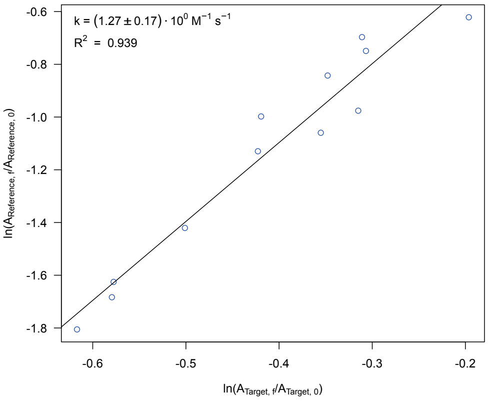

(a) Fenofibrate - Alachlor

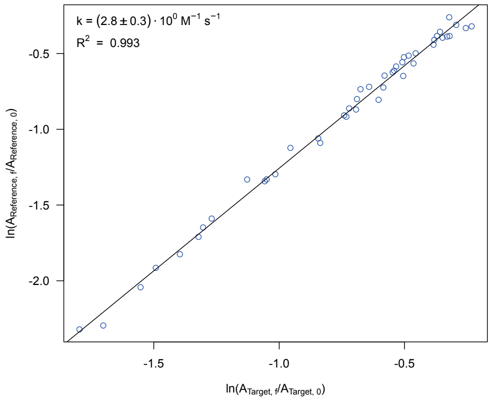

(b) Fenofibric acid - Alachlor

Figure SI-B24: Correlations between the natural logarithm of the relative residual peak areas of fenofibrate (a) and fenofibric acid (b) with competitors upon ozonation at pH 7 (2 mM phosphate), 22 °C, and in presence of *t*BuOH (40 mM). The intercept was considered negligible (<10×slope) and the standard deviations of competitor $k_{\mathrm{app,O_3}}$ values were considered in the calculation of the standard deviations of the target $k_{\mathrm{app,O_3}}$ values. Note that the axes do not necessarily originate at zero.

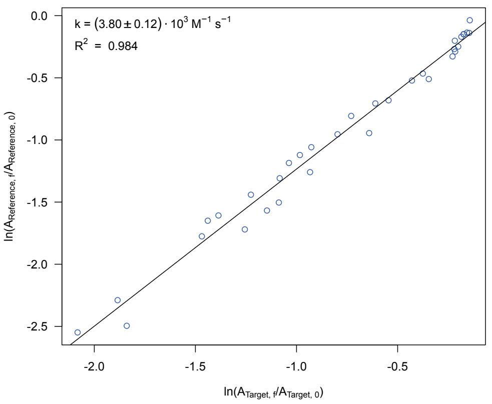

(a) Fexofenadine - Penicillin G

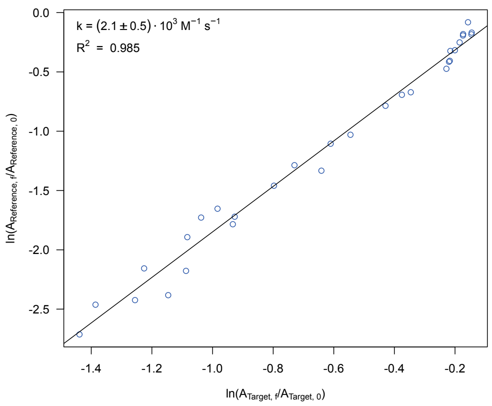

(b) Fexofenadine - Tramadol

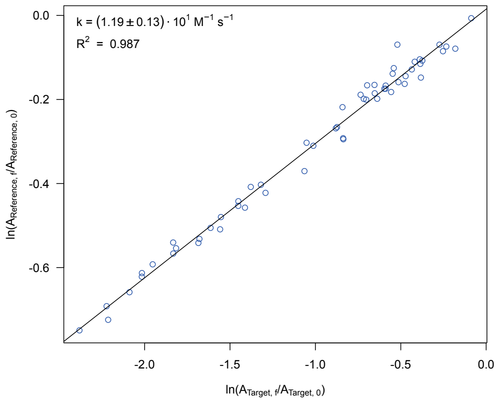

(c) Fexofenadine-*N*-oxide - Alachlor

Figure SI-B25: Correlations between the natural logarithm of the relative residual peak areas of fexofenadine (a - b) and fexofenadine-*N*-oxide (c) with competitors upon ozonation at pH 7 (2 mM phosphate), 22 °C, and in presence of *t*BuOH (40 mM). The intercept was considered negligible (<10×slope) and the standard deviations of competitor k_{app,O_3} values were considered in the calculation of the standard deviations of the target k_{app,O_3} values. Note that the axes do not necessarily originate at zero.

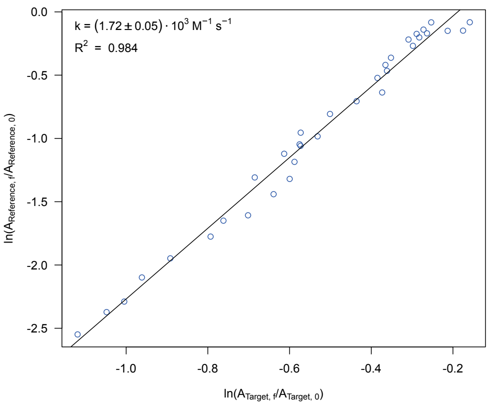

(a) Fluoxetine - Penicillin G

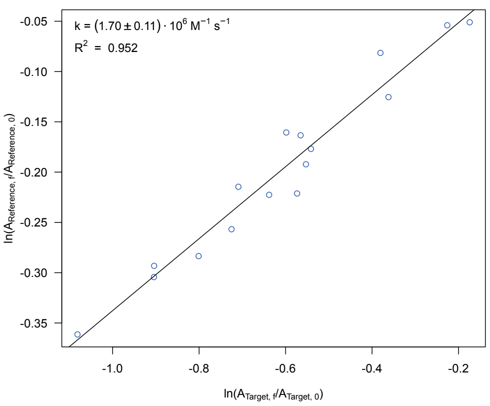

(b) 4-Trifluoromethylphenol - Carbamazepine

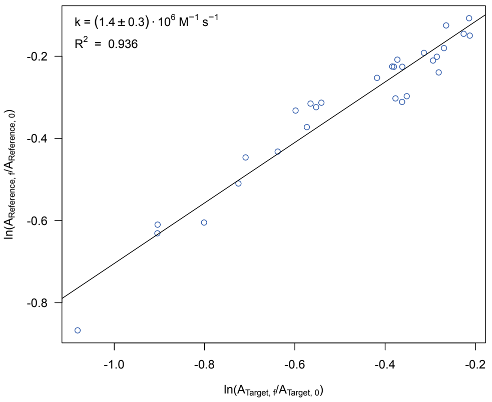

(c) 4-Trifluoromethylphenol - Sulfamethoxazole

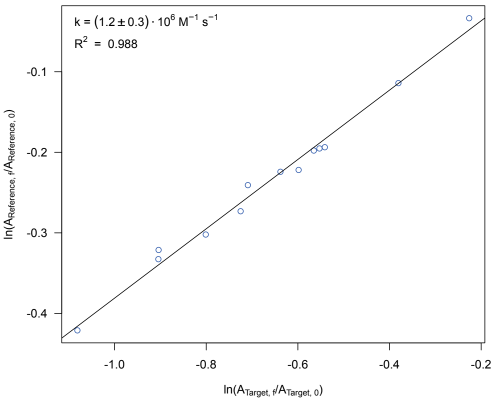

(d) 4-Trifluoromethylphenol - Trimethoprim

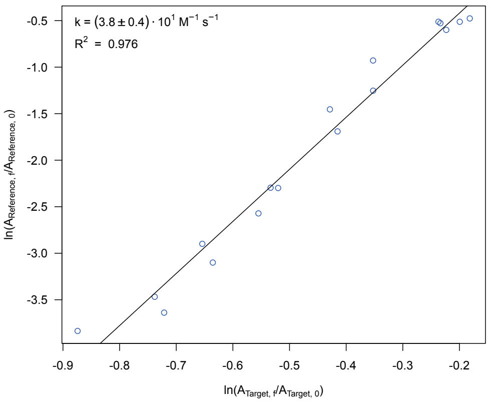

(e) Norfluoxetine - Carbofuran

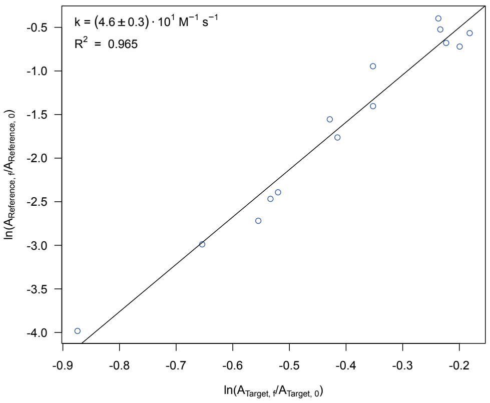

(f) Norfluoxetine - N^4-Acetylsulfamethoxazole

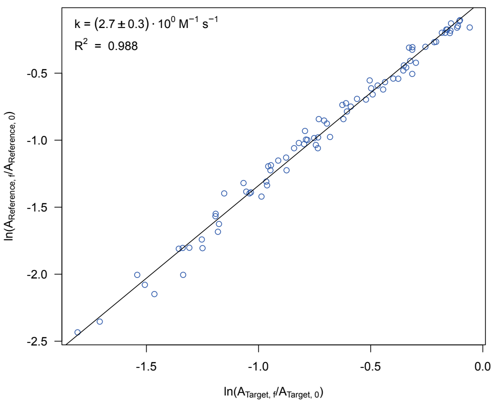

(g) Fluoxetine formamide - Alachlor

Figure SI-B25: Correlations between the natural logarithm of the relative residual peak areas of fluoxetine (a - c), 4-trifluoromethylphenol (d), norfluoxetine (e - f) and fluoxetine formamide (g) with competitors upon ozonation at pH 7 (2 mM phosphate), 22 °C, and in presence of *t*BuOH (40 mM). The intercept was considered negligible (<10×slope) and the standard deviations of competitor $k_{\mathrm{app,O_3}}$ values were considered in the calculation of the standard deviations of the target $k_{\mathrm{app,O_3}}$ values. Note that the axes do not necessarily originate at zero.

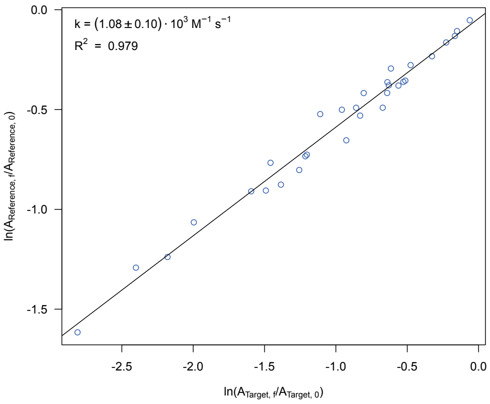

(h) Gemcitabine - Bezafibrate

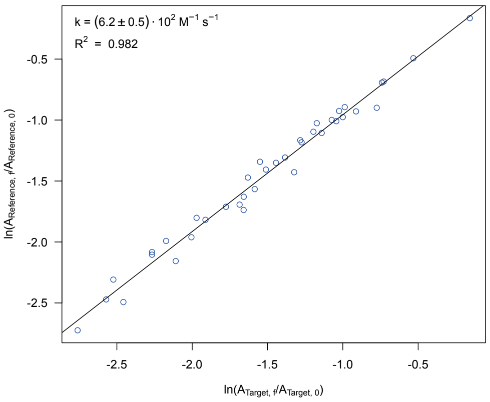

(i) 2-Deoxy-2,2-difluorouridine - Bezafibrate

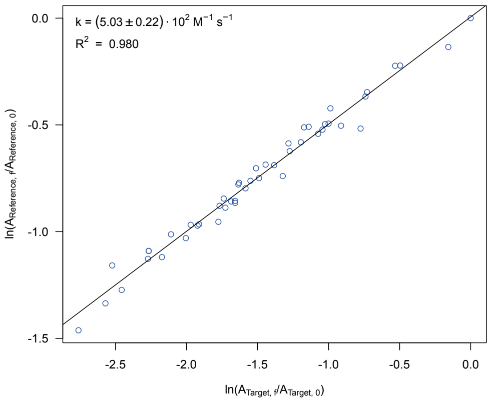

(j) 2-Deoxy-2,2-difluorouridine - N^4-Acetylsulfamethoxazole

Figure SI-B26: Correlations between the natural logarithm of the relative residual peak areas of gemcitabine (a) and 2-deoxy-2,2-difluorouridine (b - c) with competitors upon ozonation at pH 7 (2 mM phosphate), 22 °C, and in presence of *t*BuOH (40 mM). The intercept was considered negligible (<10×slope) and the standard deviations of competitor $k_{\mathrm{app,O_3}}$ values were considered in the calculation of the standard deviations of the target $k_{\mathrm{app,O_3}}$ values. Note that the axes do not necessarily originate at zero.

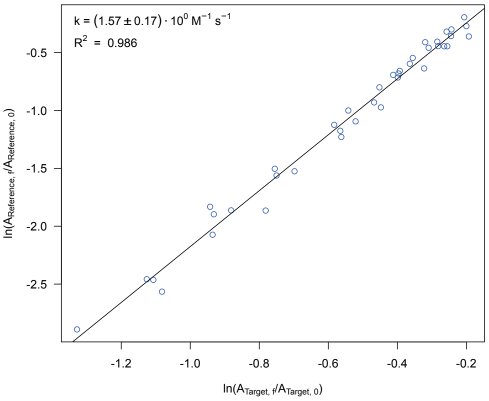

(a) Chlorothiazide - Alachlor

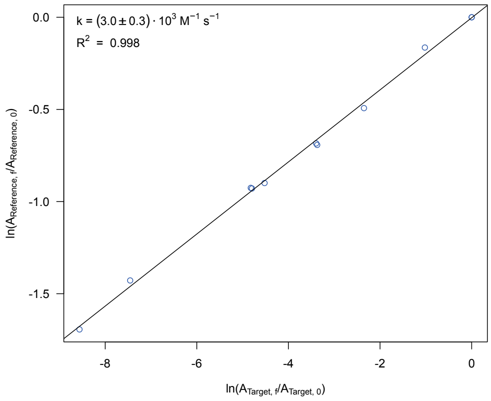

(b) Hydrochlorothiazide - Bezafibrate

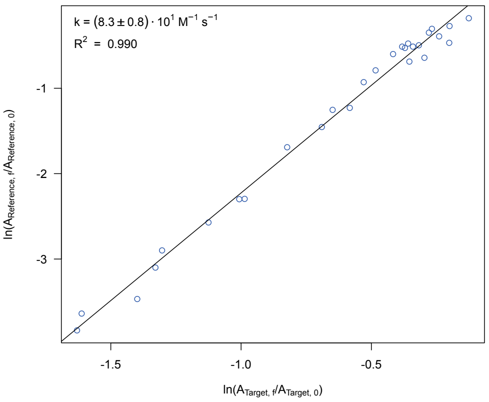

(c) 4-Amino-6-chlorobenzene-1,3-disulfonamide - Carbofuran

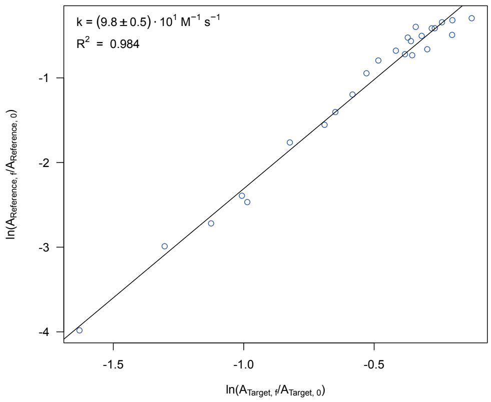

(d) 4-Amino-6-chlorobenzene-1,3-disulfonamide - N^4-Acetylsulfamethoxazole

Figure SI-B27: Correlations between the natural logarithm of the relative residual peak areas of chlorothiazide (a), hydrochlorothiazide (b) and 4-amino-6-chlorobenzene-1,3-disulfonamide (c - d) with competitors upon ozonation at pH 7 (2 mM phosphate), 22 °C, and in presence of *t*BuOH (40 mM). The intercept was considered negligible (<10×slope) and the standard deviations of competitor k_{app,O_3} values were considered in the calculation of the standard deviations of the target k_{app,O_3} values. Note that the axes do not necessarily originate at zero.

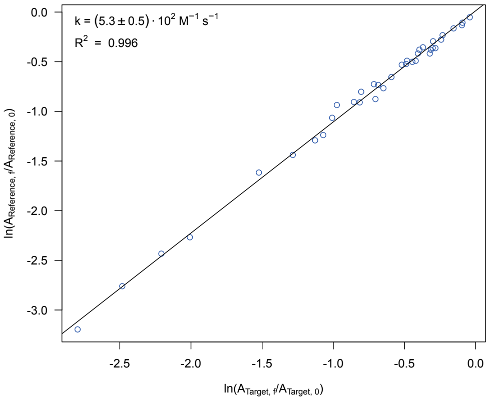

(a) Cortisone - Bezafibrate

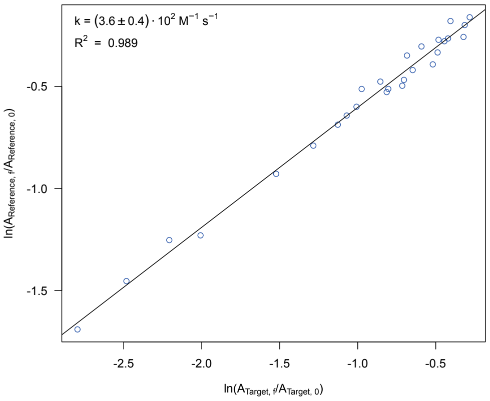

(b) Cortisone - Carbofuran

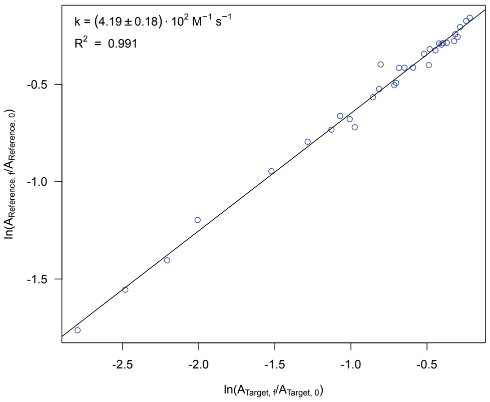

(c) Cortisone - N^4-Acetylsulfamethoxazole

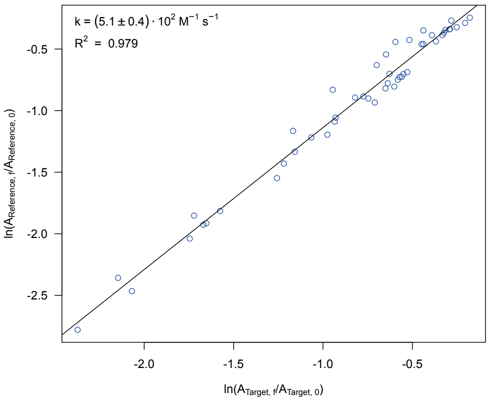

(d) Hydrocortisone - Bezafibrate

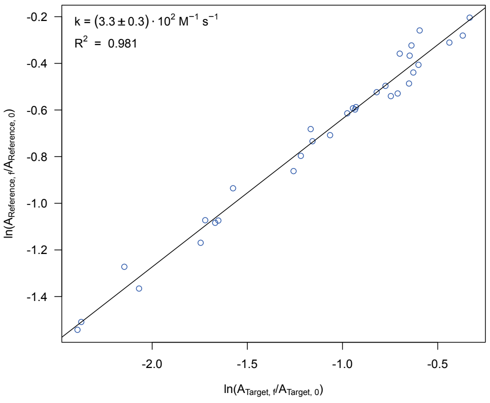

(e) Hydrocortisone - Carbofuran

Figure SI-B28: Correlations between the natural logarithm of the relative residual peak areas of cortisone (a - c) and hydrocortisone (d - e) with competitors upon ozonation at pH 7 (2 mM phosphate), 22 °C, and in presence of *t*BuOH (40 mM). The intercept was considered negligible (<10×slope) and the standard deviations of competitor k_{app,O_3} values were considered in the calculation of the standard deviations of the target k_{app,O_3} values. Note that the axes do not necessarily originate at zero.

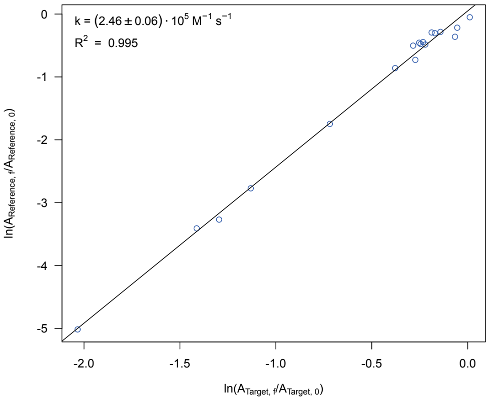

(a) Lidocaine - Carbamazepine

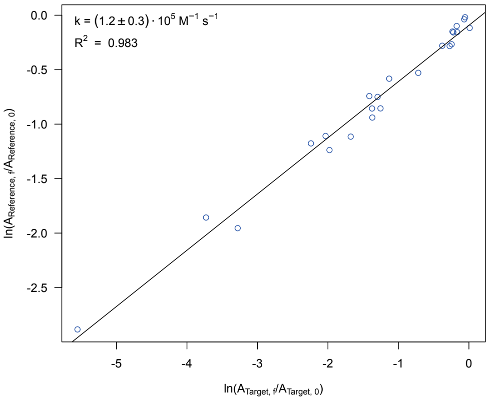

(b) Lidocaine - Roxithromycin

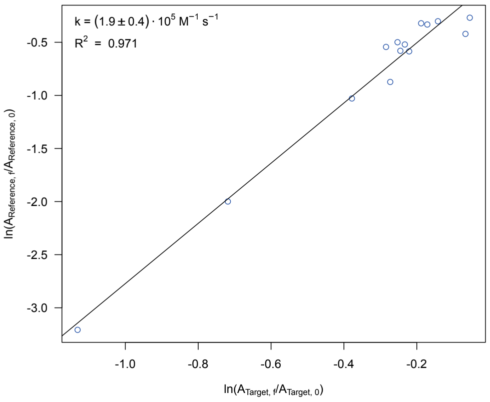

(c) Lidocaine - Trimethoprim

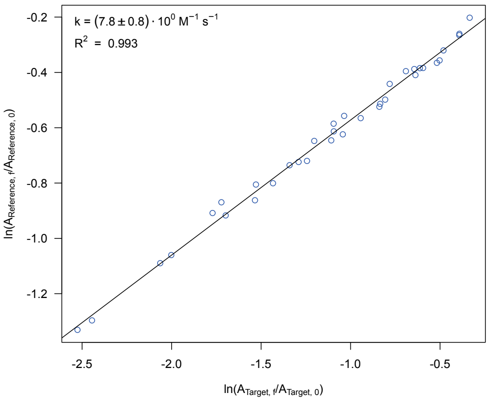

(d) Lidocaine-*N*-oxide - Alachlor

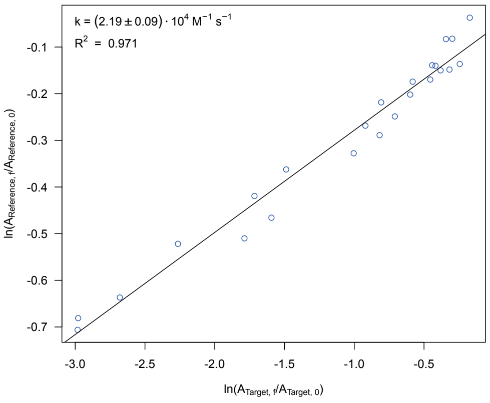

(e) Lidocaine-*N*-desethyl - Penicillin G

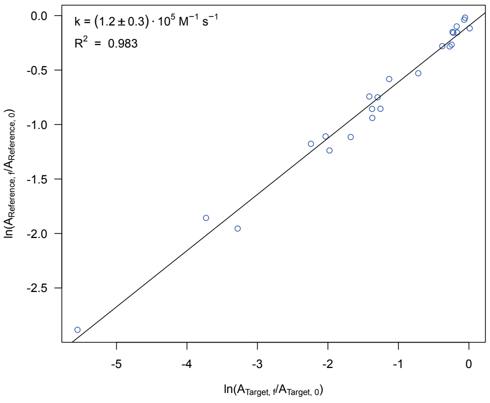

(f) Lidocaine-*N*-desethyl - Roxithromycin

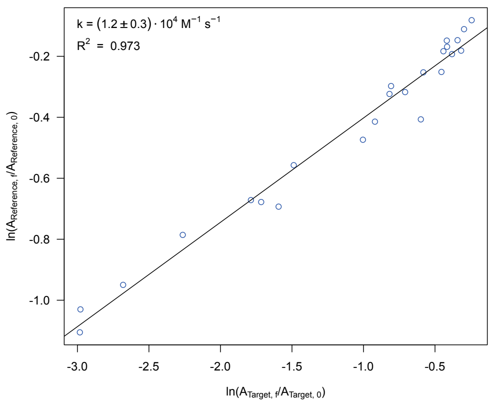

(g) Lidocaine-*N*-desethyl - Tramadol

Figure SI-B28: Correlations between the natural logarithm of the relative residual peak areas of lidocaine (a - c), lidocaine-*N*-oxide (d) and lidocaine-*N*-desethyl (e - g) with competitors upon ozonation at pH 7 (2 mM phosphate), 22 °C, and in presence of *t*BuOH (40 mM). The intercept was considered negligible (<10×slope) and the standard deviations of competitor $k_{\mathrm{app,O_3}}$ values were considered in the calculation of the standard deviations of the target $k_{\mathrm{app,O_3}}$ values. Note that the axes do not necessarily originate at zero.

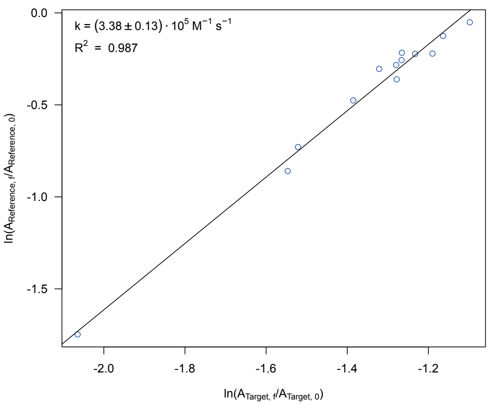

(h) 4-Aminoantipyrine - Carbamazepine

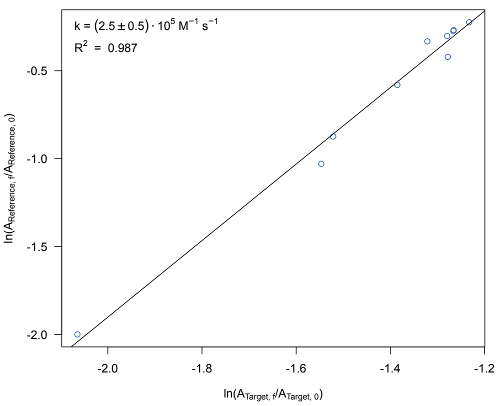

(i) 4-Aminoantipyrine - Trimethoprim

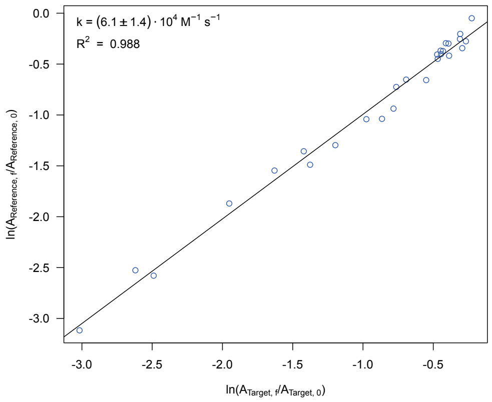

(j) 4-Formylaminantipyrine - Roxithromycin

Figure SI-B29: Correlations between the natural logarithm of the relative residual peak areas of 4-aminoantipyrine (a) and 4-formylaminantipyrine (b) with competitors upon ozonation at pH 7 (2 mM phosphate), 22 °C, and in presence of *t*BuOH (40 mM). The intercept was considered negligible (<10×slope) and the standard deviations of competitor k_{app,O_3} values were considered in the calculation of the standard deviations of the target k_{app,O_3} values. Note that the axes do not necessarily originate at zero.

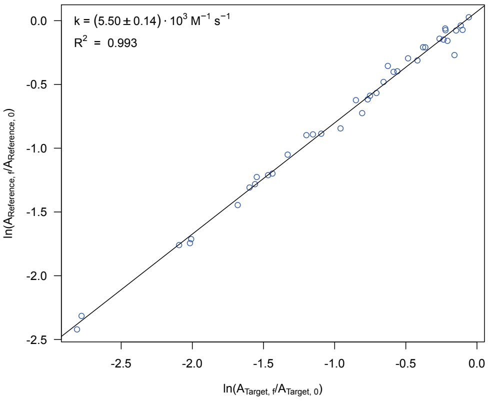

(a) Methadone - Penicillin G

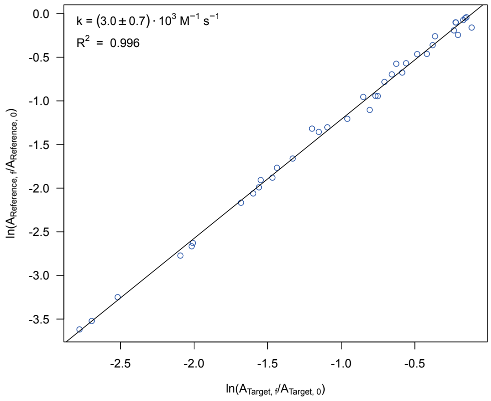

(b) Methadone - Tramadol

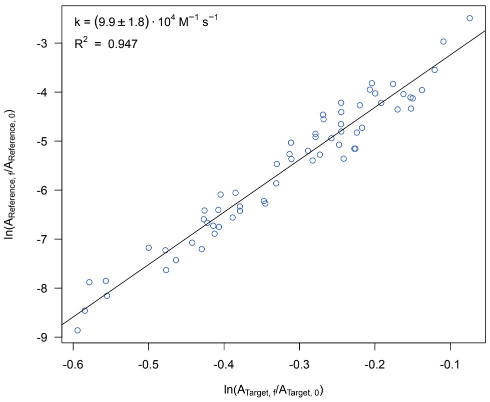

(c) EDDP - Sulfamethoxazole

Figure SI-B30: Correlations between the natural logarithm of the relative residual peak areas of methadone (a - b) and EDDP (c) with competitors upon ozonation at pH 7 (2 mM phosphate), 22 °C, and in presence of *t*BuOH (40 mM). The intercept was considered negligible (<10×slope) and the standard deviations of competitor k_{app,O_3} values were considered in the calculation of the standard deviations of the target k_{app,O_3} values. Note that the axes do not necessarily originate at zero.

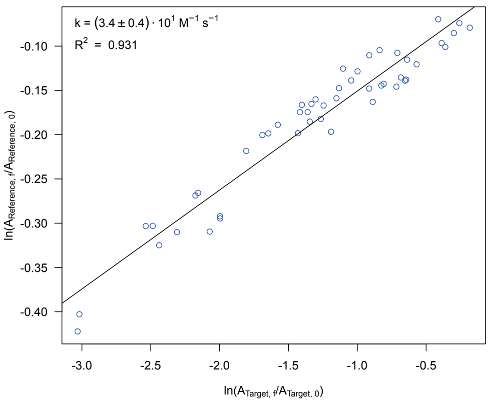

(a) Amphethamine - Alachlor

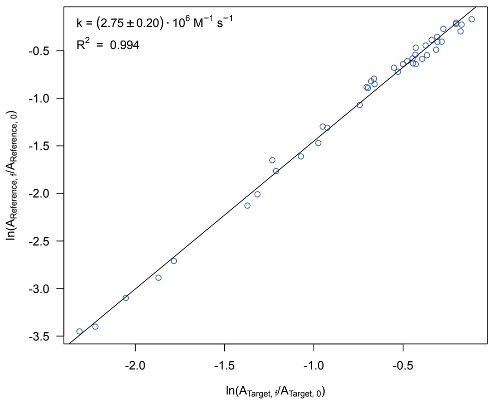

(b) 4-Hydroxyamphetamine - Dibromomethylparaben

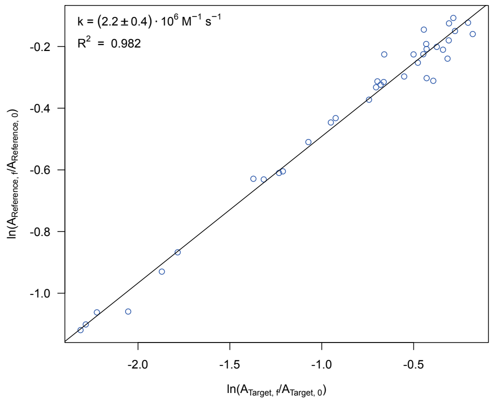

(c) 4-Hydroxyamphetamine - Sulfamethoxazole

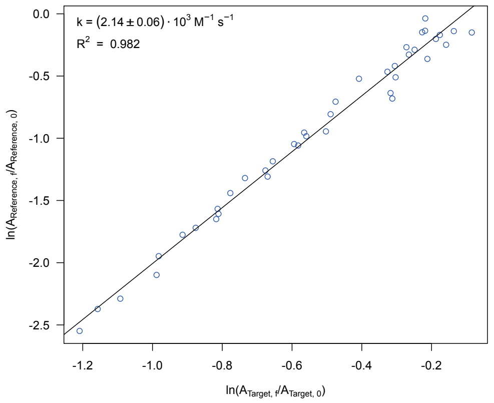

(d) Ephedrine - Penicillin G

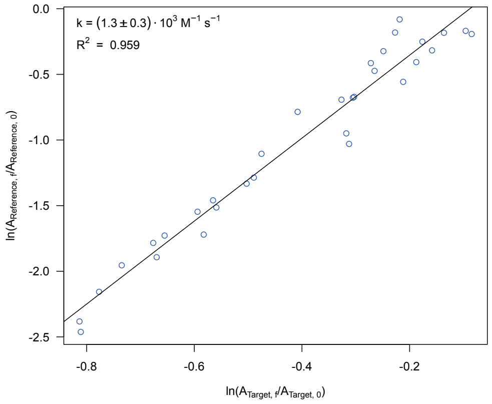

(e) Ephedrine - Tramadol

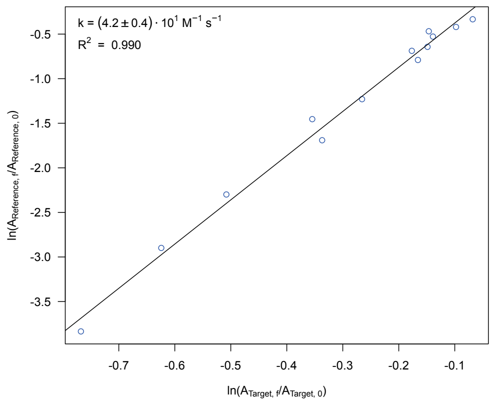

(f) Norephedrine - Carbofruan

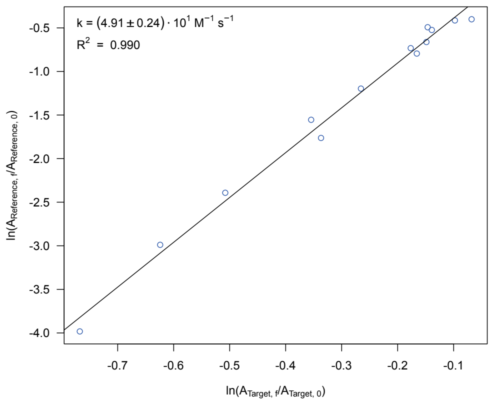

(g) Norephedrine - N^4-Acetylsulfamethoxazole

Figure SI-B30: Correlations between the natural logarithm of the relative residual peak areas of amphetamine (a), 4-hydroxyamphetamine (b), ephedrine (c) and norephedrine (d - e) with competitors upon ozonation at pH 7 (2 mM phosphate), 22 °C, and in presence of *t*BuOH (40 mM). The intercept was considered negligible (<10×slope) and the standard deviations of competitor $k_{\mathrm{app,O_3}}$ values were considered in the calculation of the standard deviations of the target $k_{\mathrm{app,O_3}}$ values. Note that the axes do not necessarily originate at zero.

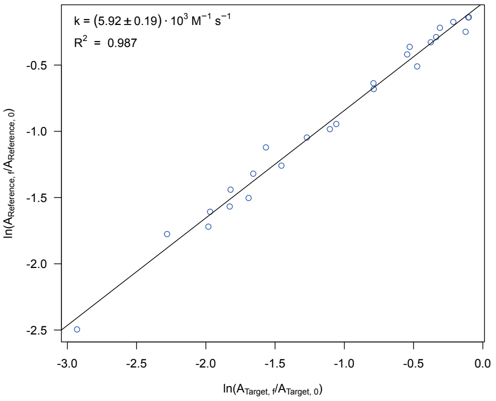

(h) Methylphenidate - Penicillin G

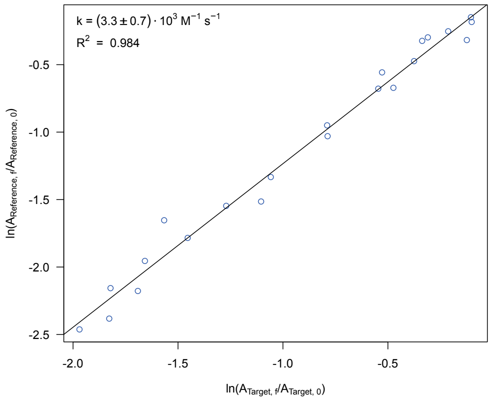

(i) Methylphenidate - Tramadol

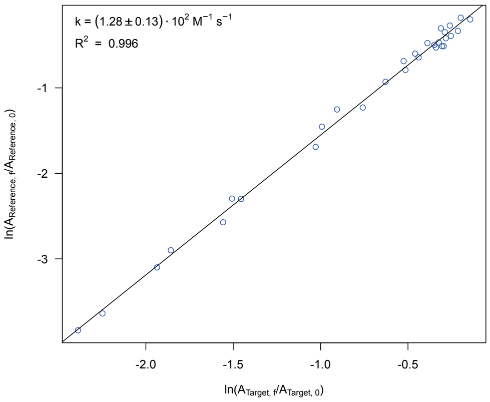

(j) Ritalinic acid - Carbofuran

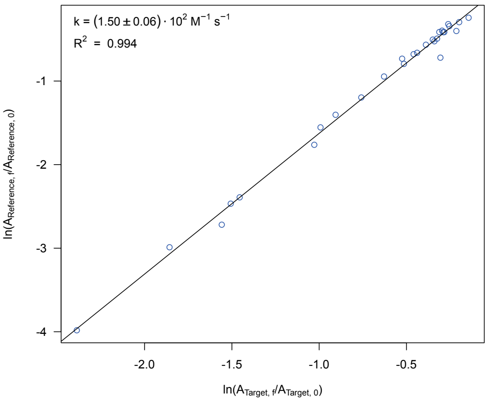

(k) Ritalinic acid - N^4-Acetylsulfamethoxazole

Figure SI-B31: Correlations between the natural logarithm of the relative residual peak areas of methylphenidate (a - b) and ritalinic acid (c - d) with competitors upon ozonation at pH 7 (2 mM phosphate), 22 °C, and in presence of *t*BuOH (40 mM). The intercept was considered negligible (<10×slope) and the standard deviations of competitor $k_{\mathrm{app,O_3}}$ values were considered in the calculation of the standard deviations of the target $k_{\mathrm{app,O_3}}$ values. Note that the axes do not necessarily originate at zero.

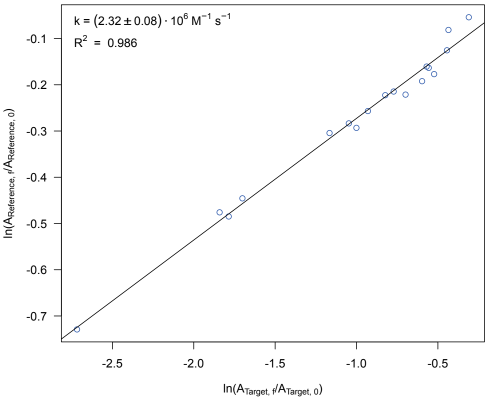

(a) Mianserin - Carbamazepine

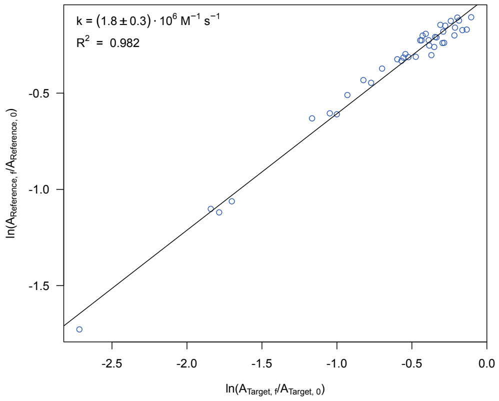

(b) Mianserin - Sulfamethoxazole

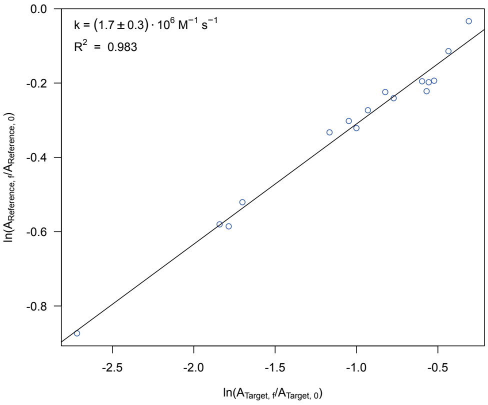

(c) Mianserin - Trimethoprim

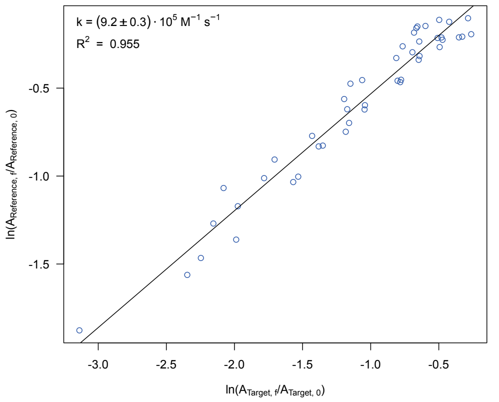

(d) Normianserin - Carbamazepine

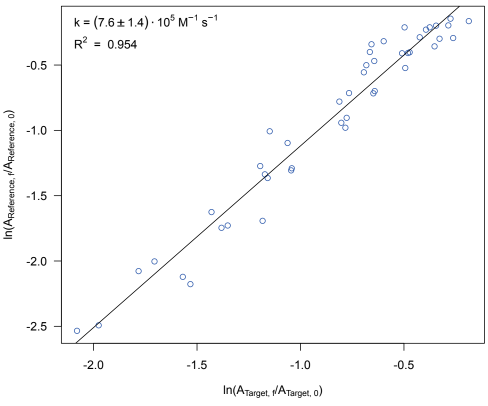

(e) Normianserin - Sulfamethoxazole

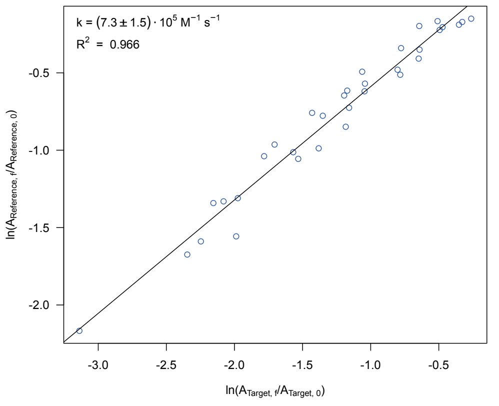

(f) Normianserin - Trimethoprim

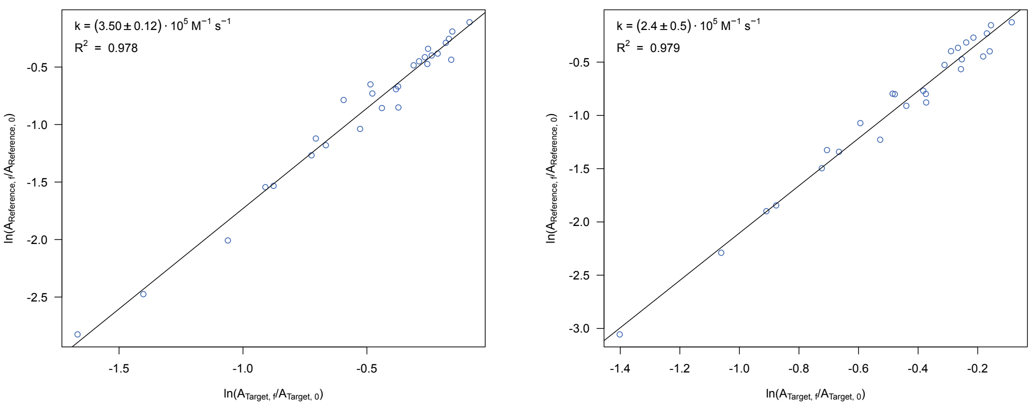

(g) Mianserin-*N*-oxide - Carbamazepine

(h) Mianserin-*N*-oxide - Trimethoprim

Figure SI-B31: Correlations between the natural logarithm of the relative residual peak areas of mianserin (a - c), normianserin (d - f) and mianserin-*N*-oxide (g - h) with competitors upon ozonation at pH 7 (2 mM phosphate), 22 °C, and in presence of *t*BuOH (40 mM). The intercept was considered negligible (<10×slope) and the standard deviations of competitor $k_{\mathrm{app,O_3}}$ values were considered in the calculation of the standard deviations of the target $k_{\mathrm{app,O_3}}$ values. Note that the axes do not necessarily originate at zero.

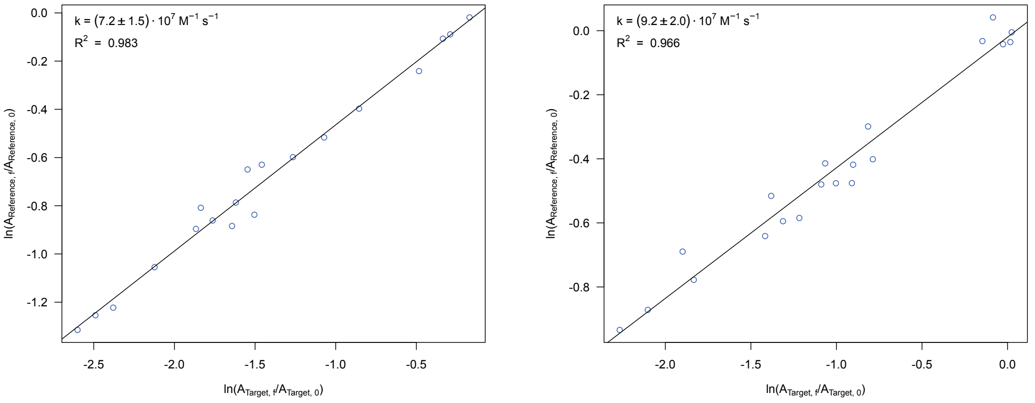

(i) Mycophenolic acid - Triclosan

(j) 6-*O*-Desmethylmycophenolic acid - Triclosan

Figure SI-B32: Correlations between the natural logarithm of the relative residual peak areas of mycophenolic acid (a), and 6-*O*-desmethylmycophenolic acid (b) with competitors upon ozonation at pH 7 (2 mM phosphate), 22 °C, and in presence of *t*BuOH (40 mM). The intercept was considered negligible (<10×slope) and the standard deviations of competitor $k_{\mathrm{app,O_3}}$ values were considered in the calculation of the standard deviations of the target $k_{\mathrm{app,O_3}}$ values. Note that the axes do not necessarily originate at zero.

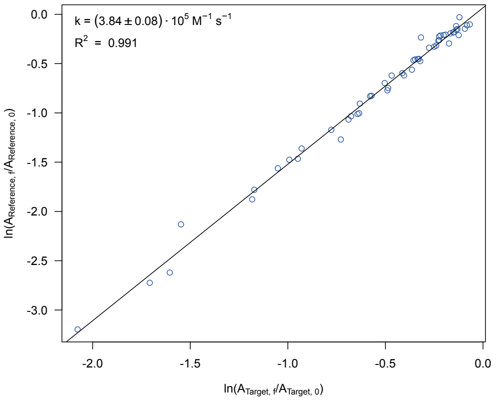

(a) Nicotine - Carbamazepine

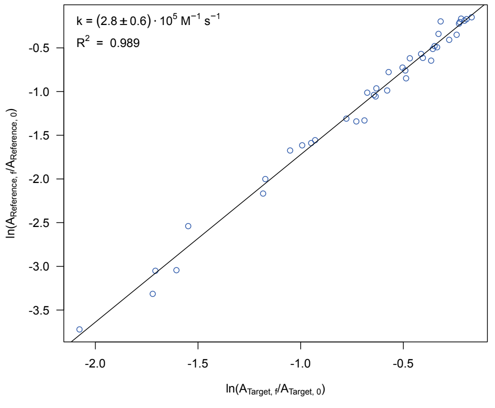

(b) Nicotine - Trimethoprim

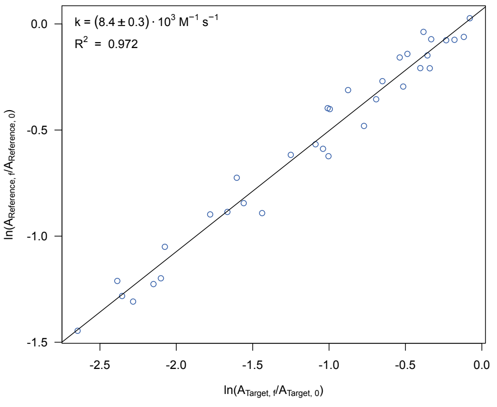

(c) Nornicotine - Penicillin G

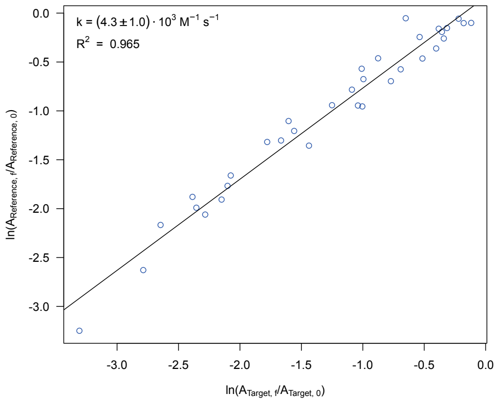

(d) Nornicotine - Tramadol

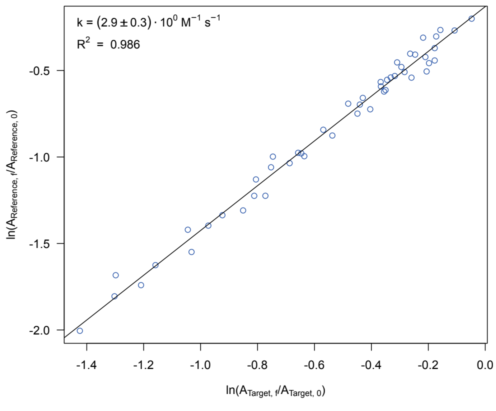

(e) Cotinine - Alachlor

Figure SI-B33: Correlations between the natural logarithm of the relative residual peak areas of nicotine (a - b), nornicotine (c - d) and cotinine (e) with competitors upon ozonation at pH 7 (2 mM phosphate), 22 °C, and in presence of *t*BuOH (40 mM). The intercept was considered negligible (<10×slope) and the standard deviations of competitor $k_{\mathrm{app,O_3}}$ values were considered in the calculation of the standard deviations of the target $k_{\mathrm{app,O_3}}$ values. Note that the axes do not necessarily originate at zero.

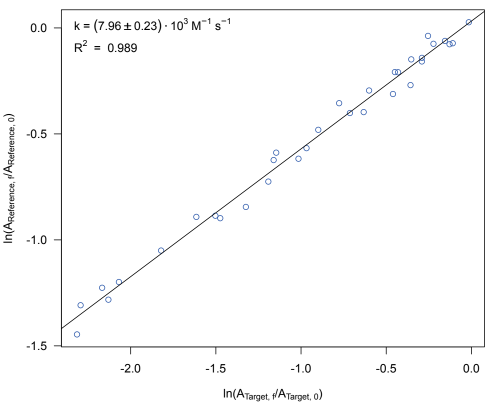

(a) Oseltamivir - Penicillin G

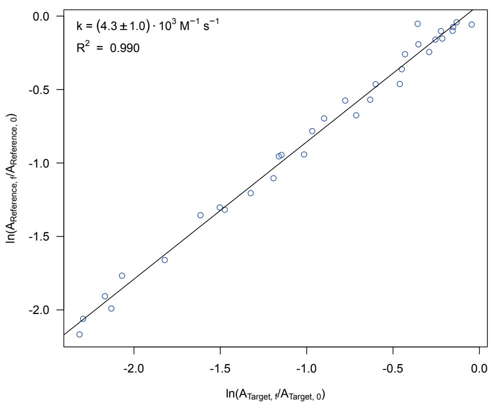

(b) Oseltamivir - Tramadol

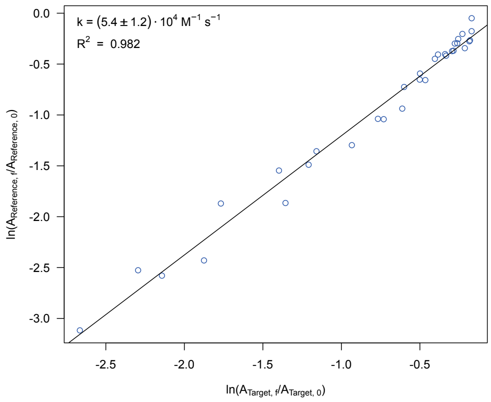

(c) Oseltamivir acid - Roxithromycin

Figure SI-B34: Correlations between the natural logarithm of the relative residual peak areas of oseltamivir (a - b) and oseltamivir acid (c) with competitors upon ozonation at pH 7 (2 mM phosphate), 22 °C, and in presence of *t*BuOH (40 mM). The intercept was considered negligible (<10×slope) and the standard deviations of competitor $k_{\mathrm{app,O_3}}$ values were considered in the calculation of the standard deviations of the target $k_{\mathrm{app,O_3}}$ values. Note that the axes do not necessarily originate at zero.

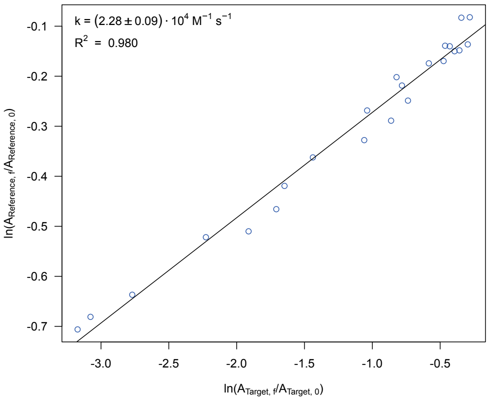

(a) Pheniramine - Penicillin G

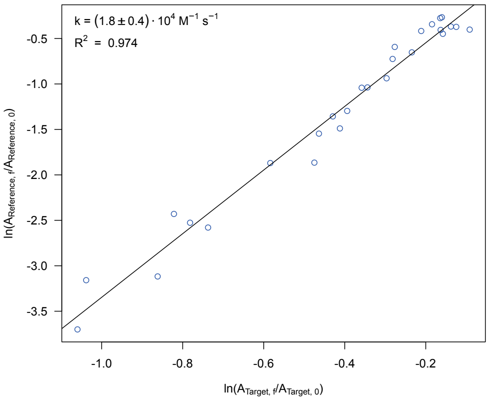

(b) Pheniramine - Roxithromycin

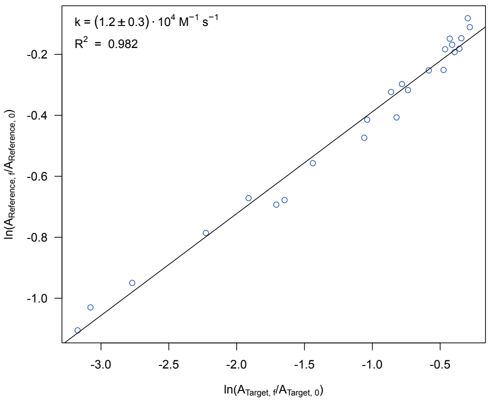

(c) Pheniramine - Tramadol

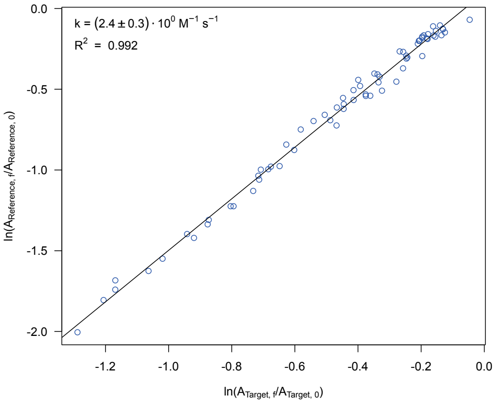

(d) Pheniramine-*N*-oxide - Alachlor

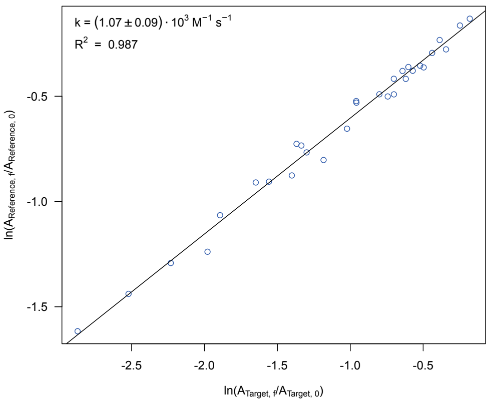

(e) *N*-Desmethylpheniramine - Bezafibrate

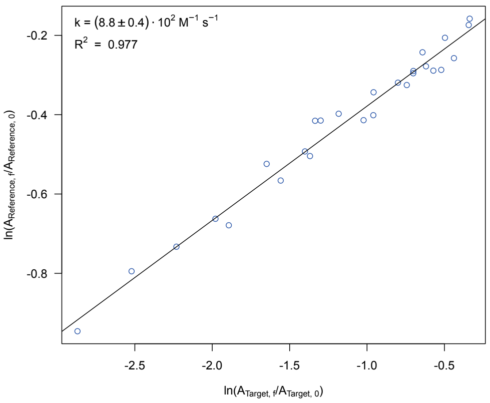

(f) *N*-Desmethylpheniramine - N^4-Acetylsulfamethoxazole

Figure SI-B35: Correlations between the natural logarithm of the relative residual peak areas of pheniramine (a - c), pheniramine-*N*-oxide (d), *N*-desmethylpheniramine (e - f) and with competitors upon ozonation at pH 7 (2 mM phosphate), 22 °C, and in presence of *t*BuOH (40 mM). The intercept was considered negligible (<10×slope) and the standard deviations of competitor $k_{\mathrm{app,O_3}}$ values were considered in the calculation of the standard deviations of the target $k_{\mathrm{app,O_3}}$ values. Note that the axes do not necessarily originate at zero.

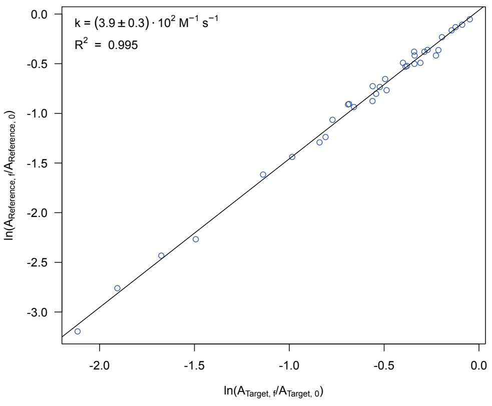

(a) Prednisone - Bezafibrate

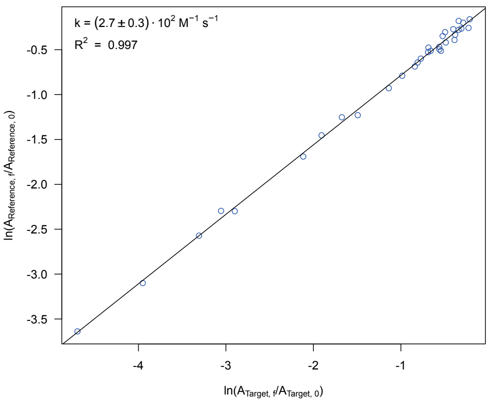

(b) Prednisone - Carbofuran

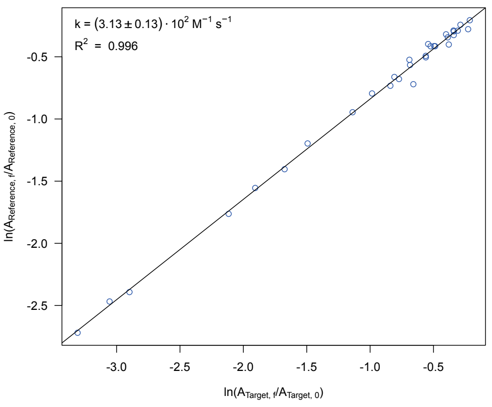

(c) Prednisone - N^4-Acetylsulfamethoxazole

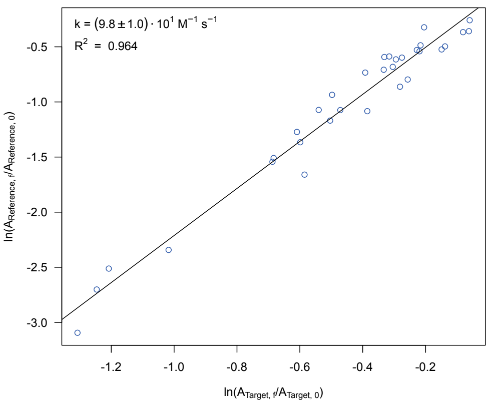

(d) Prednisolone - Carbofuran

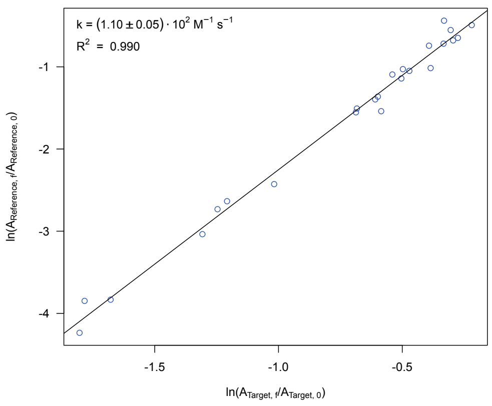

(e) Prednisolone - N^4-Acetylsulfamethoxazole

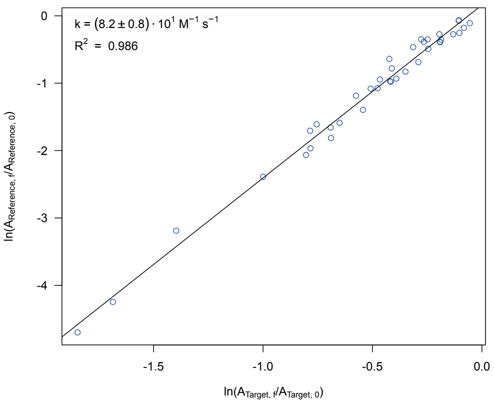

(f) Methylprednisolone - Carbofuran

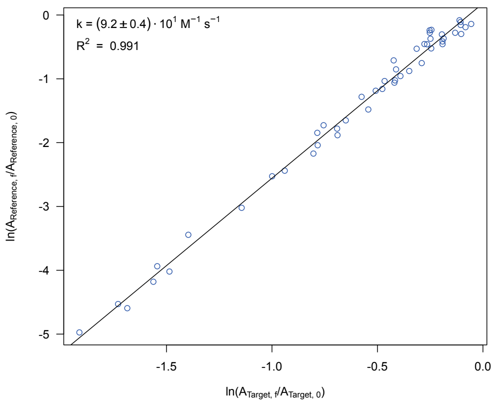

(g) Methylprednisolone - N^4-Acetylsulfamethoxazole

Figure SI-B35: Correlations between the natural logarithm of the relative residual peak areas of prednisone (a - c), prednisolone (d) and methylprednisolone (e - f) with competitors upon ozonation at pH 7 (2 mM phosphate), 22 °C, and in presence of *t*BuOH (40 mM). The intercept was considered negligible (<10×slope) and the standard deviations of competitor k_{app,O_3} values were considered in the calculation of the standard deviations of the target k_{app,O_3} values. Note that the axes do not necessarily originate at zero.

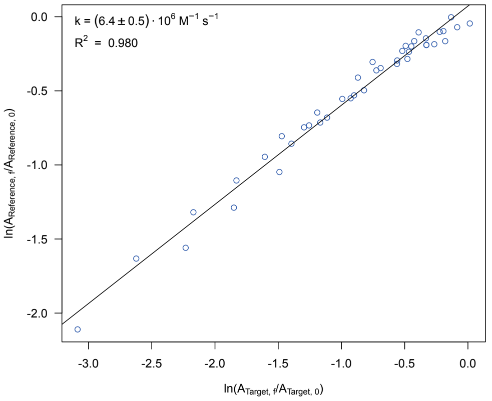

(h) Ranitidine - Dibromomethylparaben

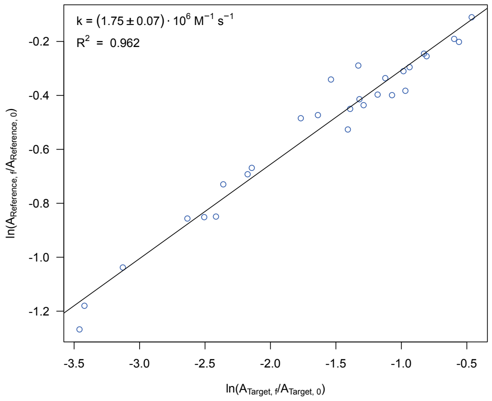

(i) Ranitidine-*S*-oxide - Carbamazepine

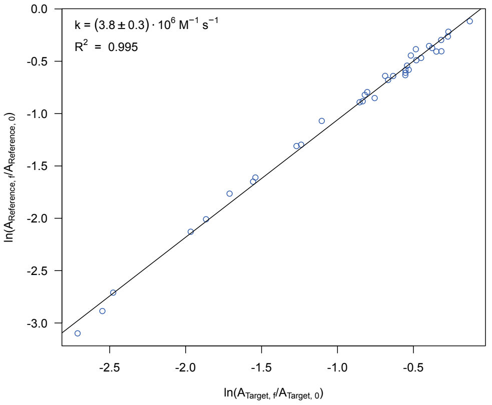

(j) Ranitidine-*N*-oxide - Dibromomethylparaben

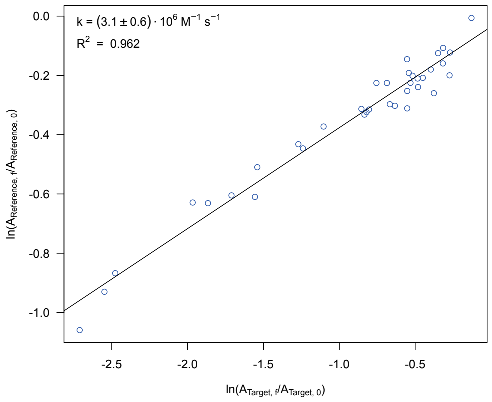

(k) Ranitidine-*N*-oxide - Sulfamethoxazole

Figure SI-B36: Correlations between the natural logarithm of the relative residual peak areas of ranitidine (a), ranitidine-*S*-oxide (b) and ranitidine-*N*-oxide (c - d) and with competitors upon ozonation at pH 7 (2 mM phosphate), 22 °C, and in presence of *t*BuOH (40 mM). The intercept was considered negligible (<10×slope) and the standard deviations of competitor $k_{\mathrm{app,O_3}}$ values were considered in the calculation of the standard deviations of the target $k_{\mathrm{app,O_3}}$ values. Note that the axes do not necessarily originate at zero.

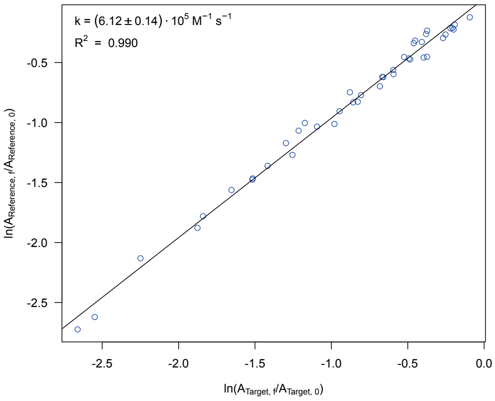

(a) Sulfapyridine - Carbamazepine

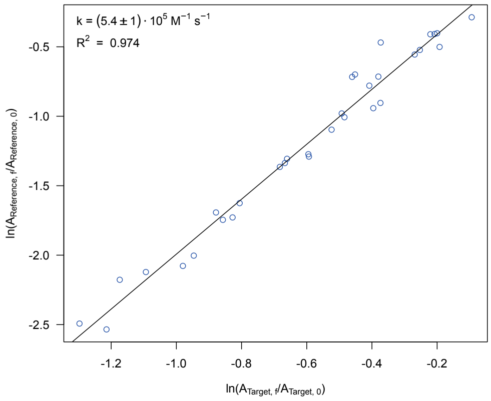

(b) Sulfapyridine - Sulfamethoxazole

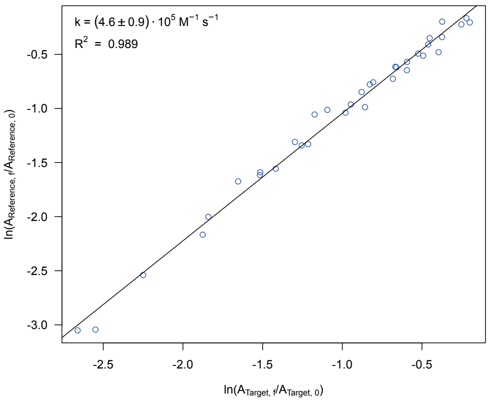

(c) Sulfapyridine - Trimethoprim

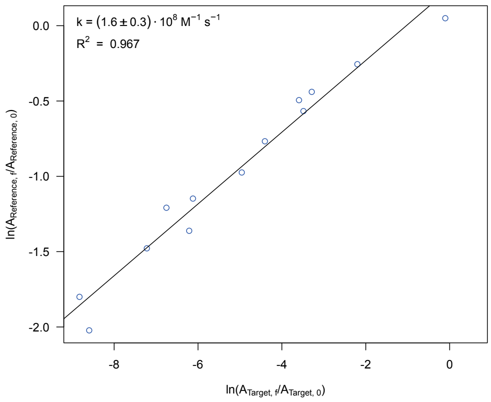

(d) 5-Hydroxysulfapyridine - Triclosan

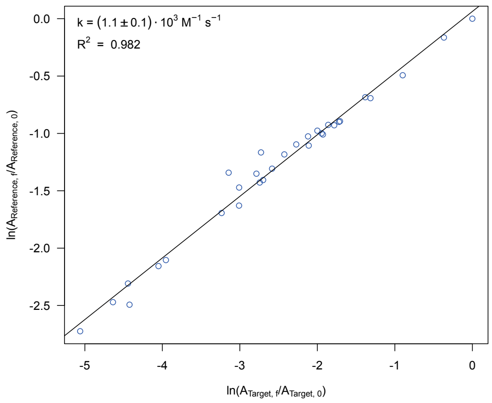

(e) N^4-Acetylsulfapyridine - Bezafibrate

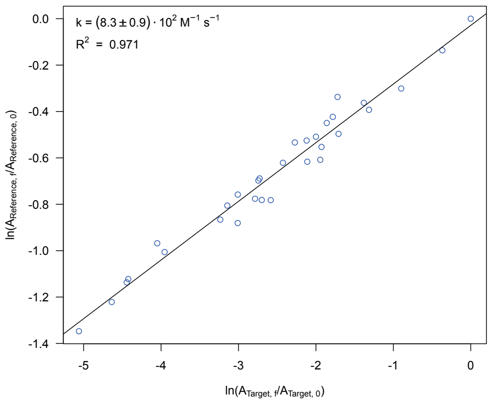

(f) N^4-Acetylsulfapyridine - Carbofuran

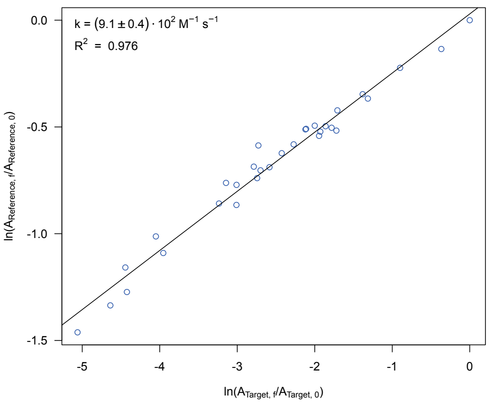

(g) N^4-Acetylsulfapyridine - N^4-Acetylsulfamethoxazole

Figure SI-B36: Correlations between the natural logarithm of the relative residual peak areas of sulfapyridine (a - c), 5-hydroxysulfapyridine (d) and N^4-acetylsulfapyridine (e - g) with competitors upon ozonation at pH 7 (2 mM phosphate), 22 °C, and in presence of tBuOH (40 mM). The intercept was considered negligible (<10×slope) and the standard deviations of competitor $k_{\mathrm{app,O_3}}$ values were considered in the calculation of the standard deviations of the target $k_{\mathrm{app,O_3}}$ values. Note that the axes do not necessarily originate at zero.

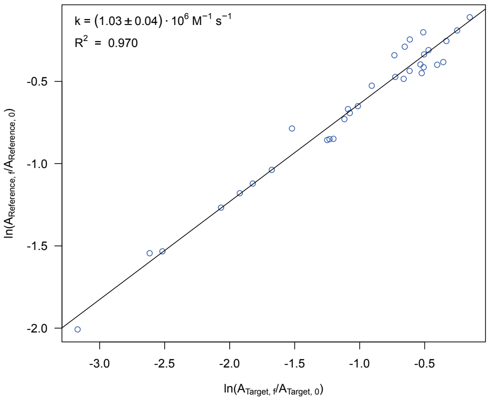

(h) Theophylline - Carbamazepine

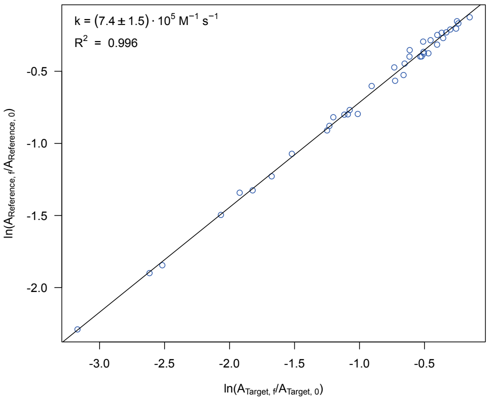

(i) Theophylline - Trimethoprim

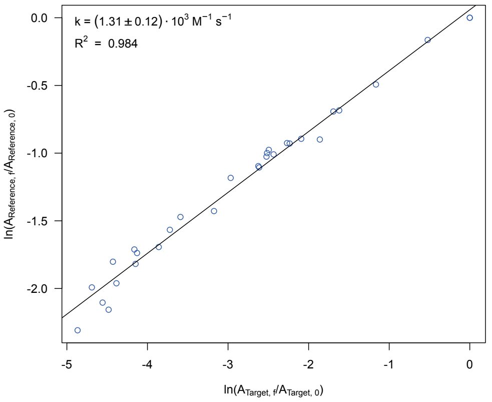

(j) Theobromine - Bezafibrate

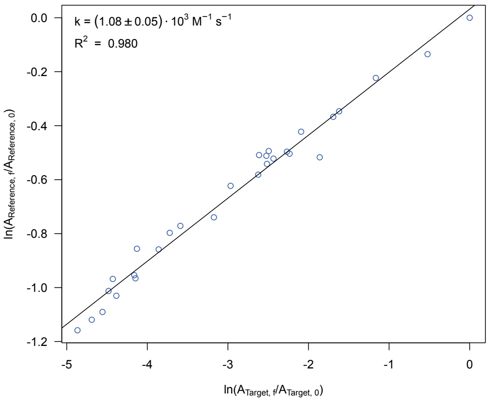

(k) Theobromine - N^4-Acetylsulfamethoxazole

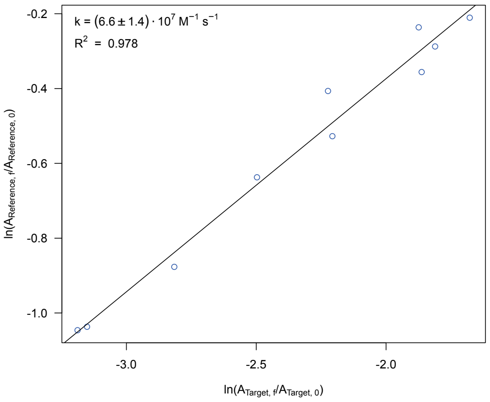

(l) 1,7-Dimethyluric acid - Triclosan

Figure SI-B37: Correlations between the natural logarithm of the relative residual peak areas of theophylline (a - b), theobromine (c - d) and 1,7-dimethyluric acid (e) with competitors upon ozonation at pH 7 (2 mM phosphate), 22 °C, and in presence of *t*BuOH (40 mM). The intercept was considered negligible (<10×slope) and the standard deviations of competitor $k_{\mathrm{app,O_3}}$ values were considered in the calculation of the standard deviations of the target $k_{\mathrm{app,O_3}}$ values. Note that the axes do not necessarily originate at zero.

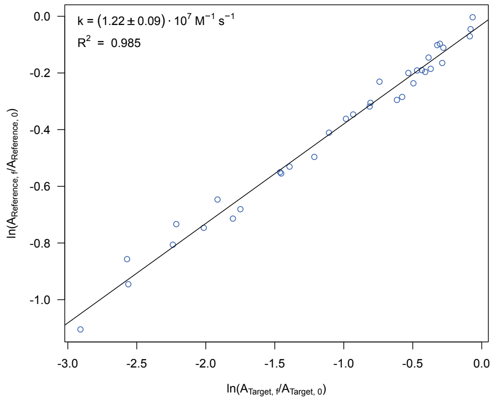

(a) Trazodone - Dibromomethylparaben

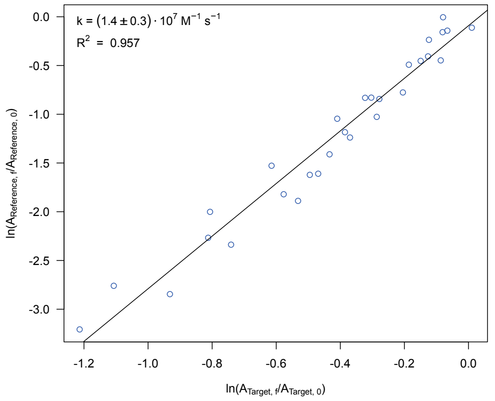

(b) Trazodone - Triclosann

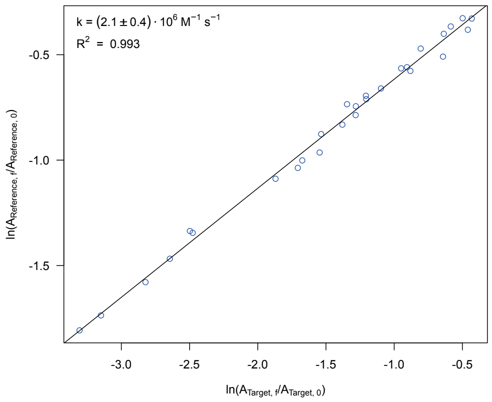

(c) 1-(3-Chlorophenyl)piperazine - Sulfamethoxazole

Figure SI-B38: Correlations between the natural logarithm of the relative residual peak areas of trazodone (a) and 1-(3-chlorophenyl)piperazine (b) with competitors upon ozonation at pH 7 (2 mM phosphate), 22 °C, and in presence of *t*BuOH (40 mM). The intercept was considered negligible (<10×slope) and the standard deviations of competitor k_{app,O_3} values were considered in the calculation of the standard deviations of the target k_{app,O_3} values. Note that the axes do not necessarily originate at zero.

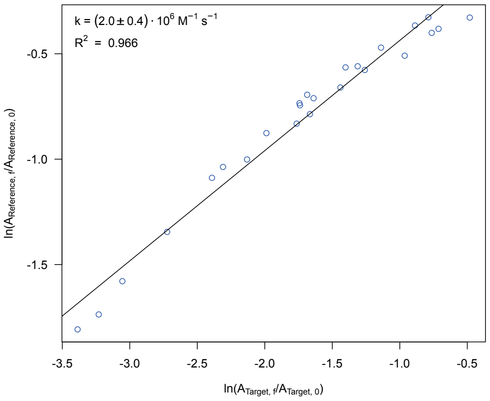

(a) Trimipramine - Sulfamethoxazole

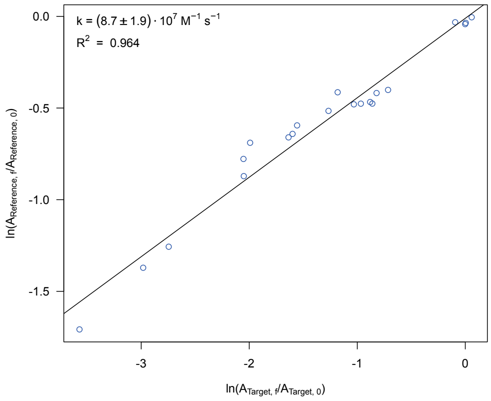

(b) 2-Hydroxytrimipramine - Triclosan

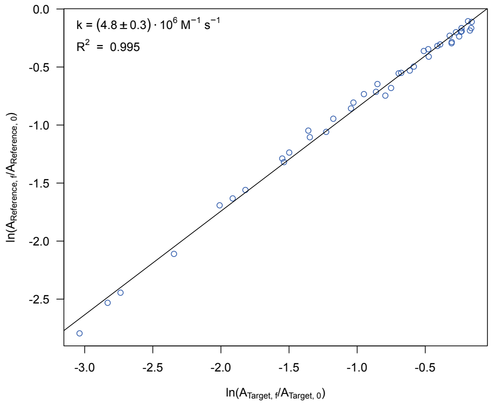

(c) Trimipramine-*N*-oxide - Dibromomethylparaben

Figure SI-B39: Correlations between the natural logarithm of the relative residual peak areas of trimipramine (a), 2-hydroxytrimipramine (b) and trimipramine-*N*-oxide (c) with competitors upon ozonation at pH 7 (2 mM phosphate), 22 °C, and in presence of *t*BuOH (40 mM). The intercept was considered negligible (<10×slope) and the standard deviations of competitor k_{app,O_3} values were considered in the calculation of the standard deviations of the target k_{app,O_3} values. Note that the axes do not necessarily originate at zero.

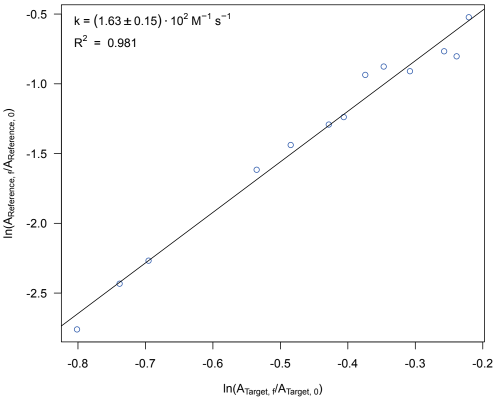

(a) Telmisartan - Bezafibrate

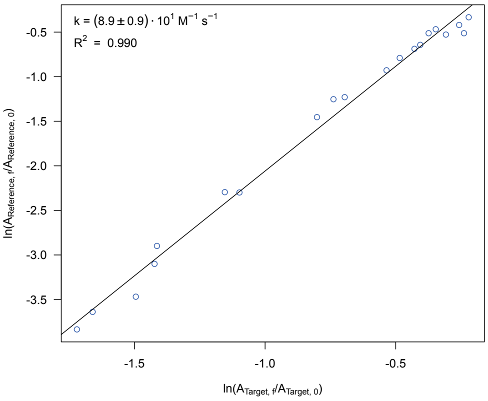

(b) Telmisartan - Carbofuran

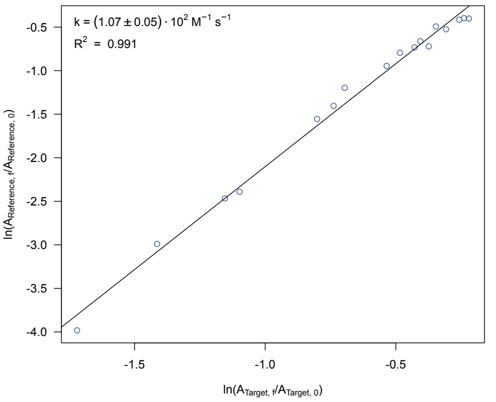

(c) Telmisartan - N^4-Acetylsulfamethoxazole

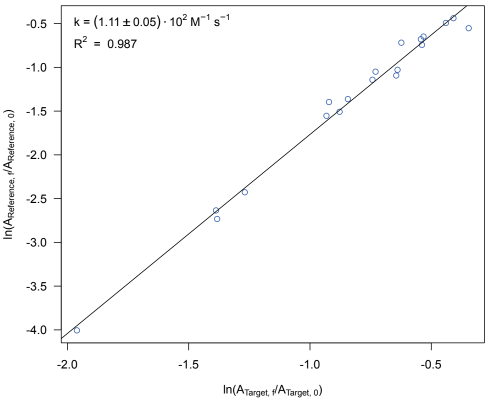

(d) Telmisartan-*O*-acyl-glucuronide - N^4-Acetylsulfamethoxazole

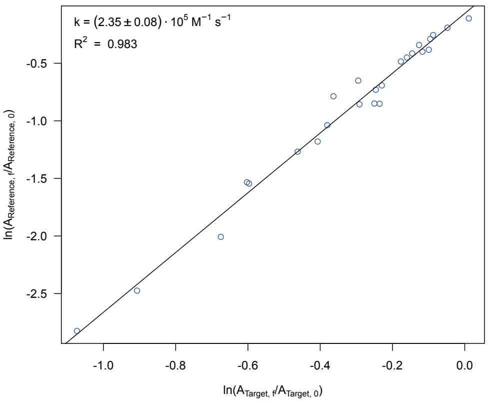

(e) Losartan - Carbamazepine

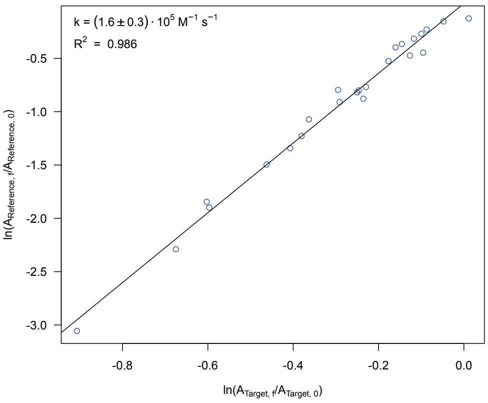

(f) Losartan - Trimethoprim

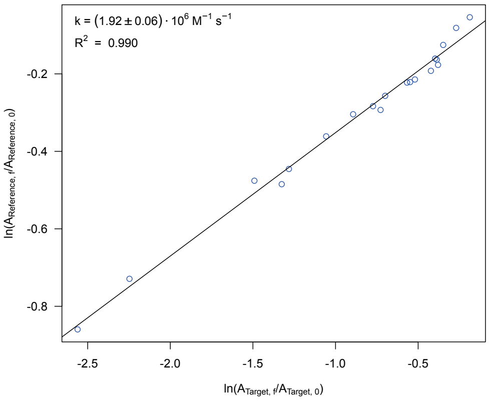

(g) Eprosartan - Carbamazepine

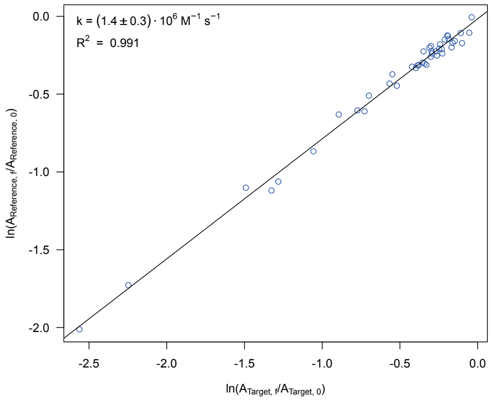

(h) Eprosartan - Sulfamethoxazole

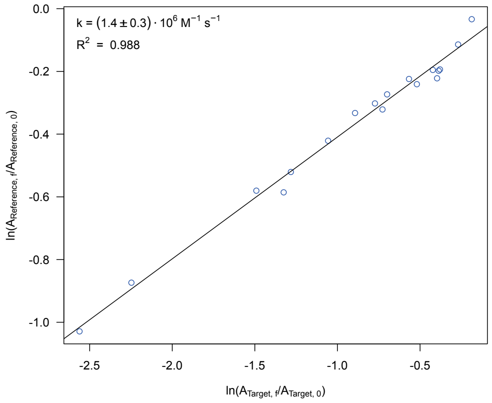

(i) Eprosartan - Trimethoprim

Figure SI-B39: Correlations between the natural logarithm of the relative residual peak areas of telmisartan (a - c), telmisartan-*O*-acyl-glucuronide (d), losartan (e - f) and eprosartan (g - i) with competitors upon ozonation at pH 7 (2 mM phosphate), 22 °C, and in presence of *t*BuOH (40 mM). The intercept was considered negligible (<10×slope) and the standard deviations of competitor $k_{\mathrm{app,O_3}}$ values were considered in the calculation of the standard deviations of the target $k_{\mathrm{app,O_3}}$ values. Note that the axes do not necessarily originate at zero.

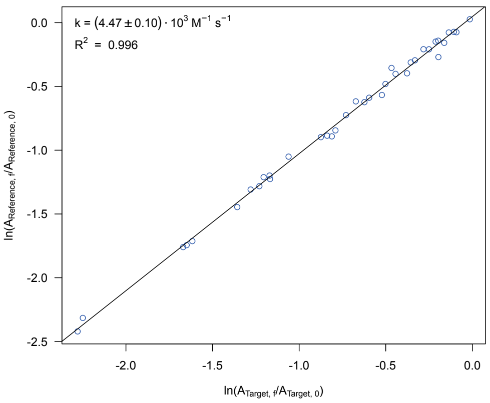

(j) Venlafaxine - Penicillin G

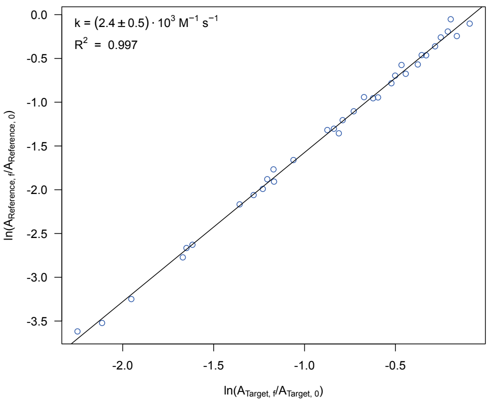

(k) Venlafaxine - Tramadol

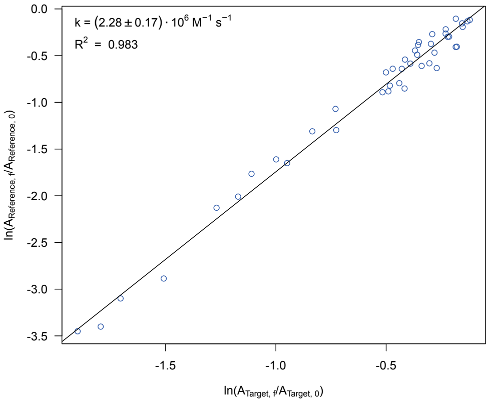

(l) *O*-Desvenlafaxine - Dibromomethylparaben

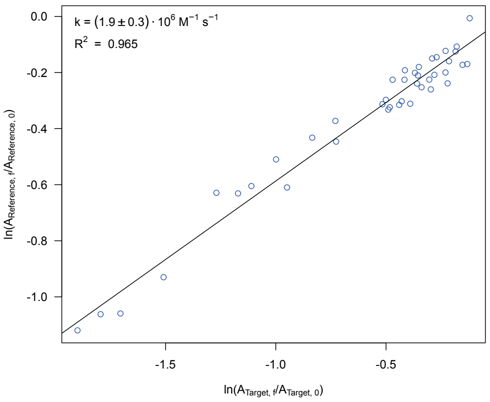

(m) *O*-Desvenlafaxine - Sulfamethoxazole

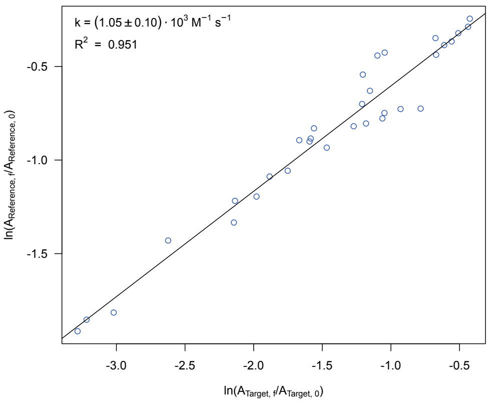

(n) *N*-Desvenlafaxine - Bezafibrate

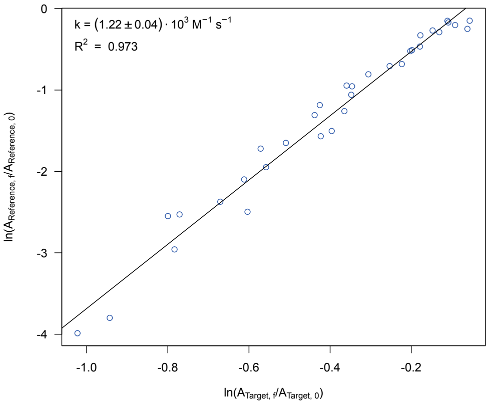

(o) *N*-Desvenlafaxine - Penicillin G

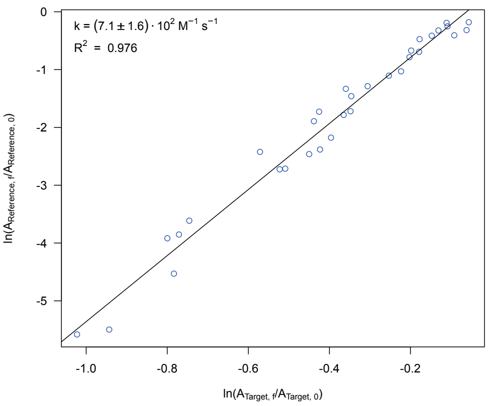

(p) *N*-Desvenlafaxine - Tramadol

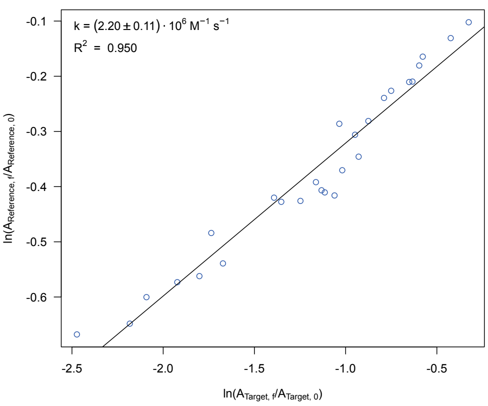

(q) *N,O*-Didesvenlafaxine - Carbamazepine

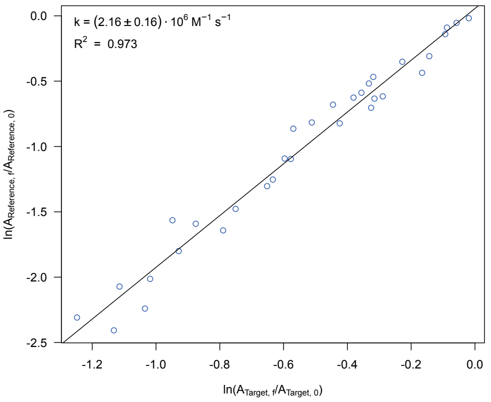

(r) *N,O*-Didesvenlafaxine - Dibromomethylparaben

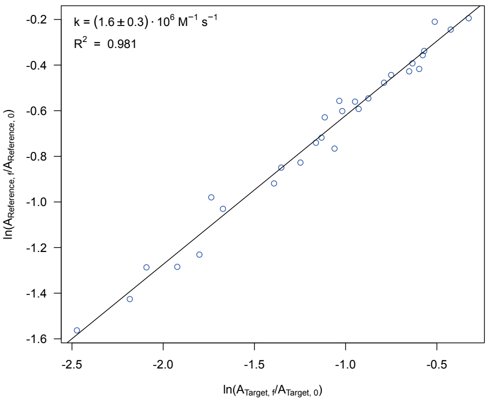

(s) *N,O*-Didesvenlafaxine - Sulfamethoxazole

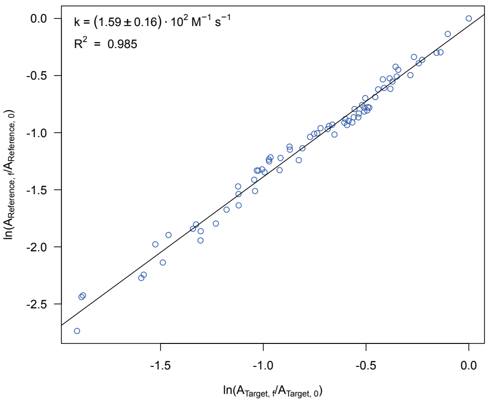

(t) Venlafaxine-*N*-oxide - Carbofuran

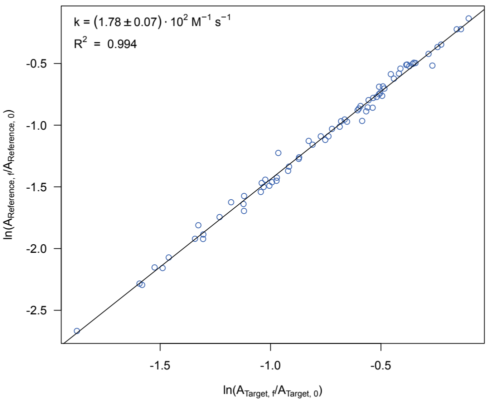

(u) Venlafaxine-*N*-oxide - N^4-Acetylsulfamethoxazole

Figure SI-B39: Correlations between the natural logarithm of the relative residual peak areas of venlafaxine (a - b), *N*-desvenlafaxine (c - d), *O*-desvenlafaxine (e), *N,O*-didesvenlafaxine (f - g) and venlafaxine-*N*-oxide (h - i) with competitors upon ozonation at pH 7 (2 mM phosphate), 22 °C, and in presence of *t*BuOH (40 mM). The intercept was considered negligible (<10×slope) and the standard deviations of competitor $k_{\mathrm{app,O_3}}$ values were considered in the calculation of the standard deviations of the target $k_{\mathrm{app,O_3}}$ values. Note that the axes do not necessarily originate at zero.

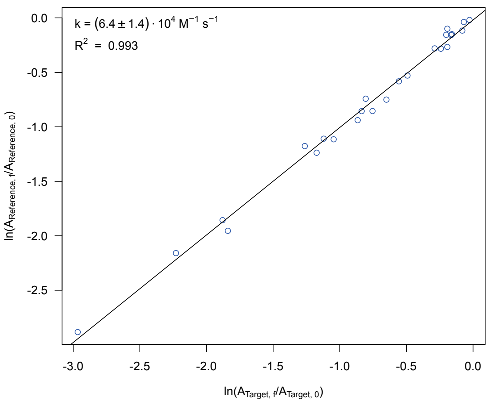

(v) Verapamil - Roxithromycin

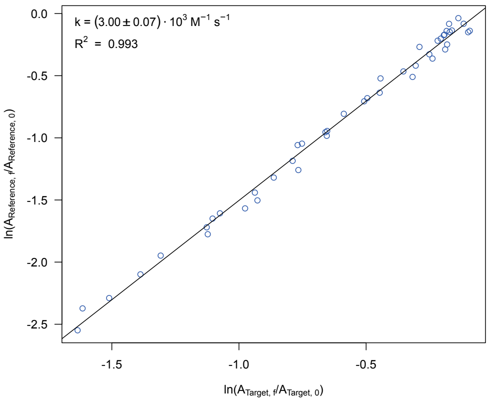

(w) D617 - Penicillin G

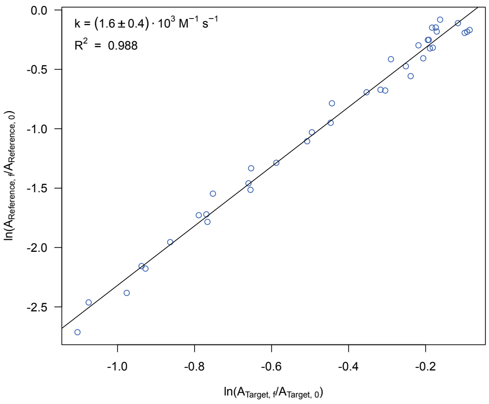

(x) D617 - Tramadol

Figure SI-B40: Correlations between the natural logarithm of the relative residual peak areas of verapamil (a) and D617 (b - c) with competitors upon ozonation at pH 7 (2 mM phosphate), 22 °C, and in presence of *t*BuOH (40 mM). The intercept was considered negligible (<10×slope) and the standard deviations of competitor $k_{\mathrm{app,O_3}}$ values were considered in the calculation of the standard deviations of the target $k_{\mathrm{app,O_3}}$ values. Note that the axes do not necessarily originate at zero.

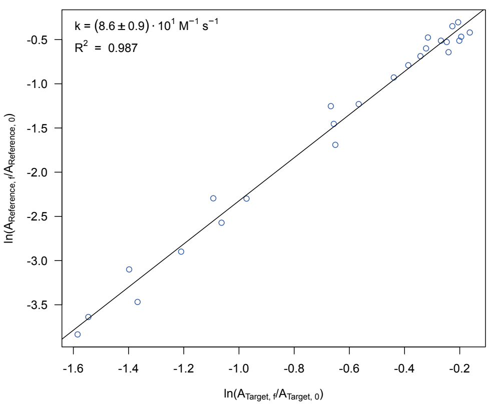

(a) Nevirapine - Carbofuran

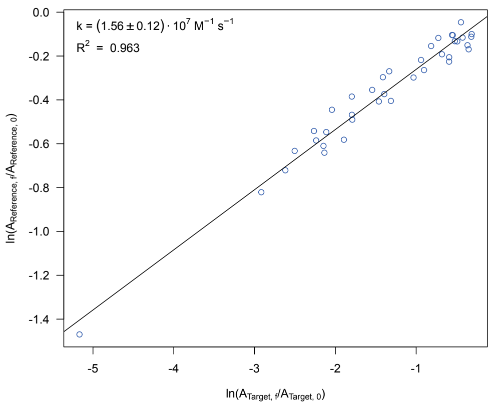

(b) 2-Hydroxynevirapine - Dibromomethylparaben

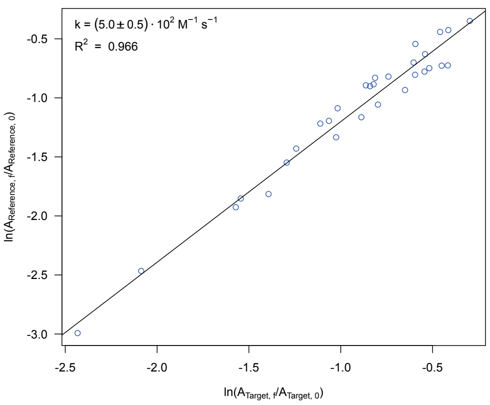

(c) 12-Hydroxynevirapine - Bezafibrate

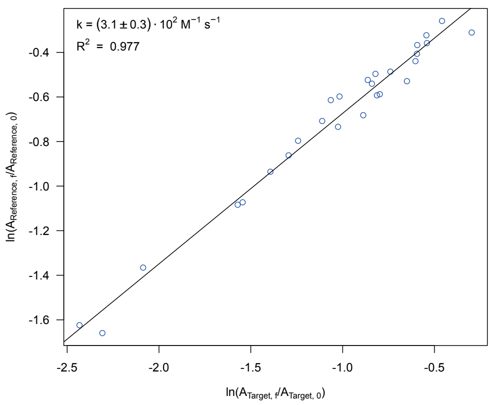

(d) 12-Hydroxynevirapine - Carbofuran

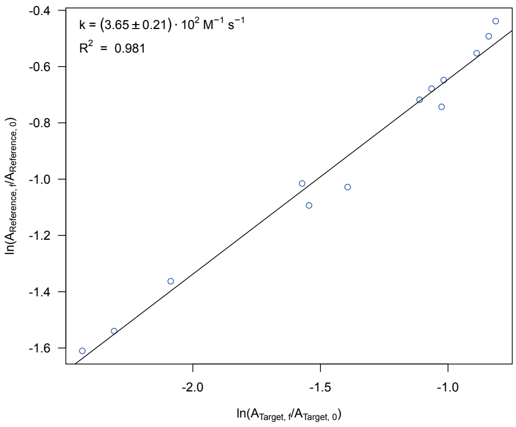

(e) 12-Hydroxynevirapine - N^4-Acetylsulfamethoxazole

Figure SI-B41: Correlations between the natural logarithm of the relative residual peak areas of nevirapine (a), 2-hydroxynevirapine (b) and 12-hydroxynevirapine (c - d) with competitors upon ozonation at pH 7 (2 mM phosphate), 22 °C, and in presence of *t*BuOH (40 mM). The intercept was considered negligible (<10×slope) and the standard deviations of competitor $k_{\text{app,O}_3}$ values were considered in the calculation of the standard deviations of the target $k_{\text{app,O}_3}$ values. Note that the axes do not necessarily originate at zero.

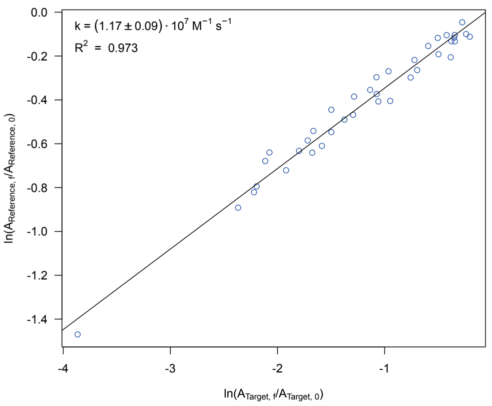

(a) 3-Desmethyltrimethoprim - Dibromomethylparaben

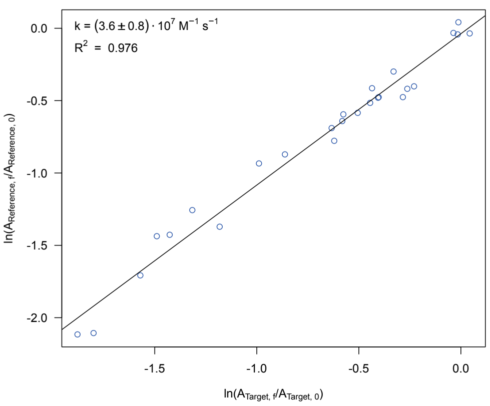

(b) 4-Desmethyltrimethoprim - Triclosan

Figure SI-B42: Correlations between the natural logarithm of the relative residual peak areas of 3-desmethyltrimethoprim (a) and 4-desmethyltrimethoprim (b) with competitors upon ozonation at pH 7 (2 mM phosphate), 22 °C, and in presence of *t*BuOH (40 mM). The intercept was considered negligible (<10×slope) and the standard deviations of competitor k_{app,O_3} values were considered in the calculation of the standard deviations of the target k_{app,O_3} values. Note that the axes do not necessarily originate at zero.

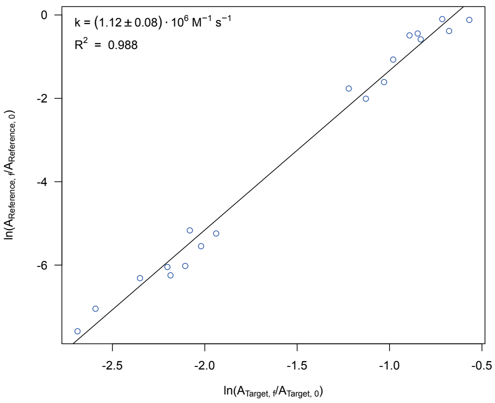

(a) Mefenamic acid - Dibromomethylparaben

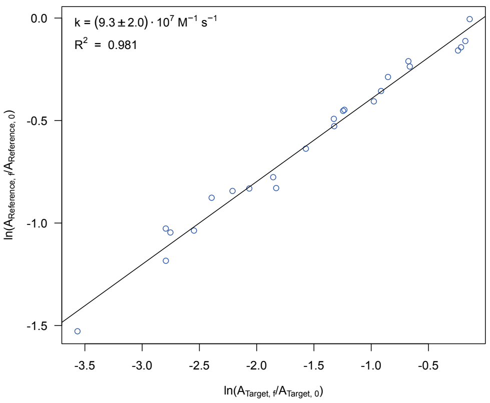

(b) 3-Hydroxymethylmefenamic acid - Triclosan

Figure SI-B43: Correlations between the natural logarithm of the relative residual peak areas of mefenamic acid (a) and 3-hydroxymethylmefenamic acid (b) with competitors upon ozonation at pH 7 (2 mM phosphate), 22 °C, and in presence of *t*BuOH (40 mM). The intercept was considered negligible (<10×slope) and the standard deviations of competitor k_{app,O_3} values were considered in the calculation of the standard deviations of the target k_{app,O_3} values. Note that the axes do not necessarily originate at zero.

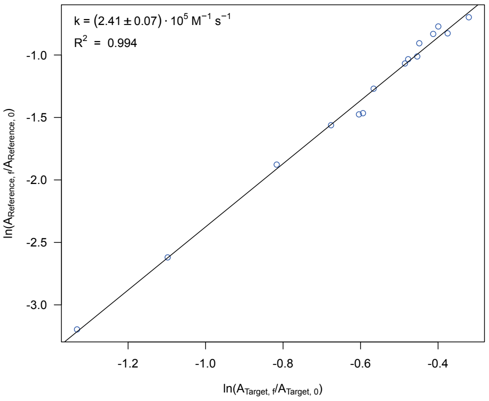

(a) Propanolol - Carbamazepine

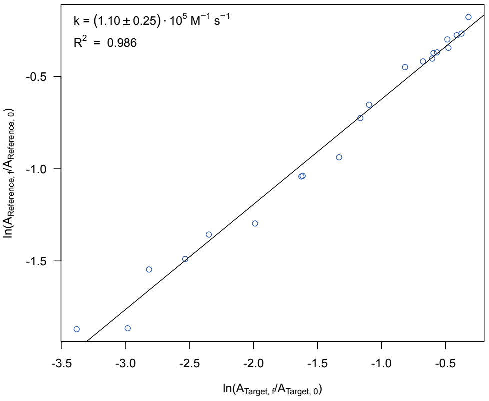

(b) Propanolol - Roxithromycin

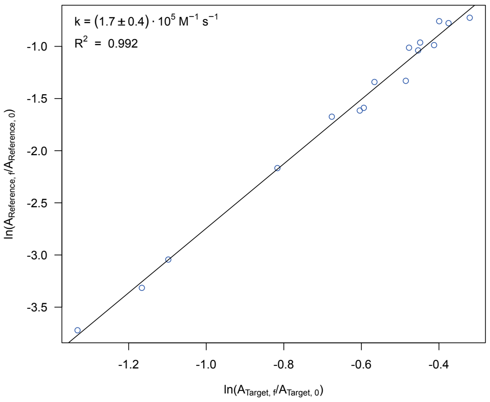

(c) Propanolol - Trimethoprim

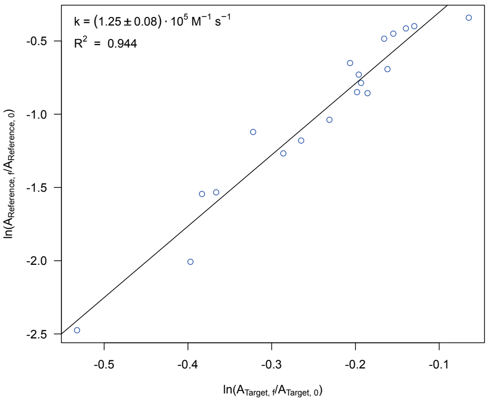

(d) 4-Hydroxypropanolol sulfate - Carbamazepine

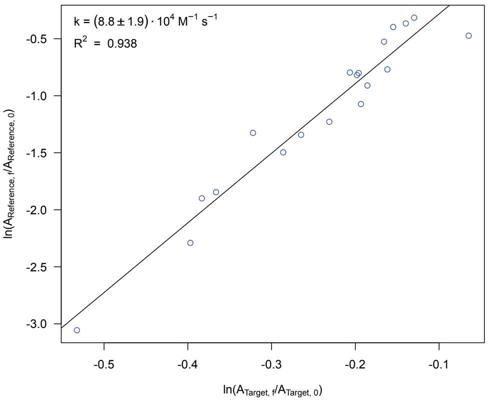

(e) 4-Hydroxypropanolol sulfate - Trimethoprim

Figure SI-B44: Correlations between the natural logarithm of the relative residual peak areas of propanolol (a) and 4-hydroxypropanolol sulfate (b - c) with competitors upon ozonation at pH 7 (2 mM phosphate), 22 °C, and in presence of *t*BuOH (40 mM). The intercept was considered negligible (<10×slope) and the standard deviations of competitor k_{app,O_3} values were considered in the calculation of the standard deviations of the target k_{app,O_3} values. Note that the axes do not necessarily originate at zero.

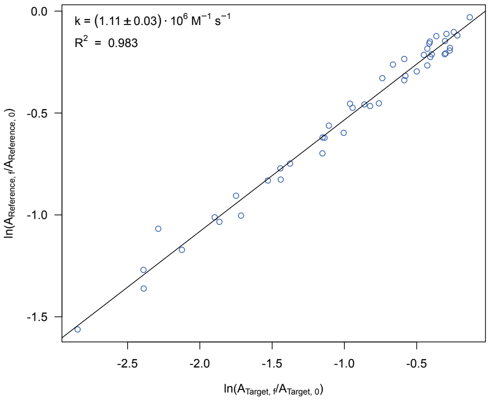

(a) Mirtazapine - Carbamazepine

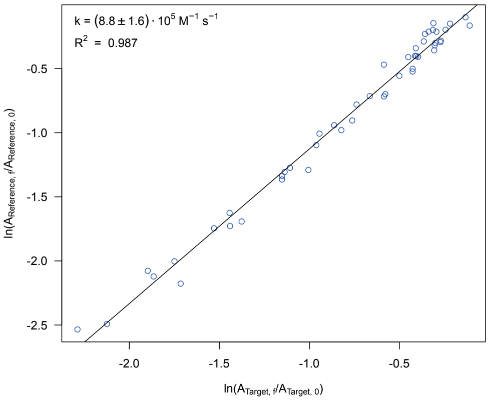

(b) Mirtazapine - Sulfamethoxazole

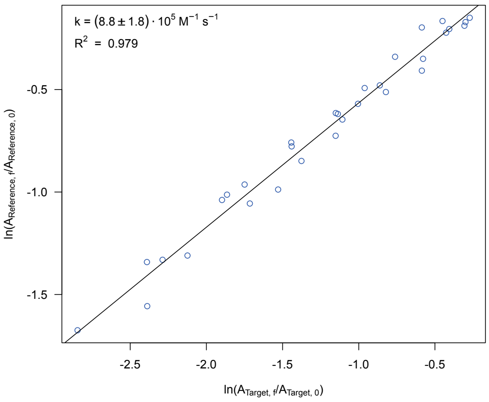

(c) Mirtazapine - Trimethoprim

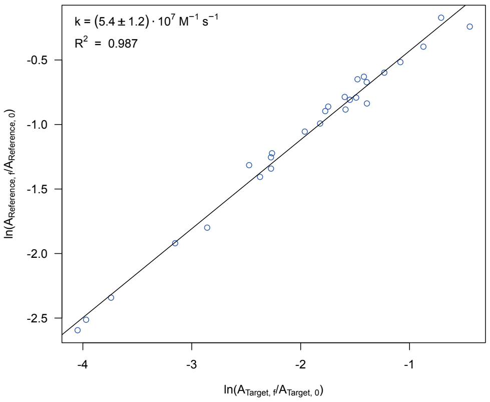

(d) 8-Hydroxymirtazapine - Triclosan

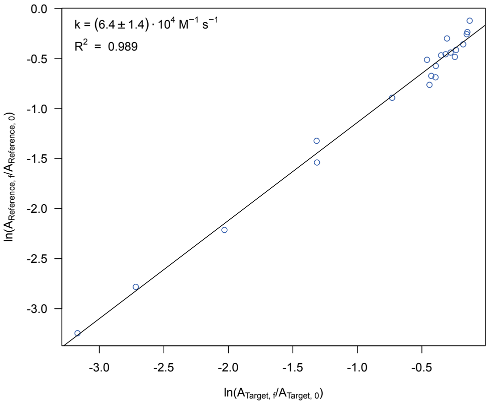

(e) 1-Oxo-mirtazapine - Roxithromycin

Figure SI-B45: Correlations between the natural logarithm of the relative residual peak areas of mirtazapine (a - c), 8-hydroxymirtazapine (d) and 1-oxo-mirtazapine (e) with competitors upon ozonation at pH 7 (2 mM phosphate), 22 °C, and in presence of *t*BuOH (40 mM). The intercept was considered negligible (<10×slope) and the standard deviations of competitor $k_{\mathrm{app,O_3}}$ values were considered in the calculation of the standard deviations of the target $k_{\mathrm{app,O_3}}$ values. Note that the axes do not necessarily originate at zero.

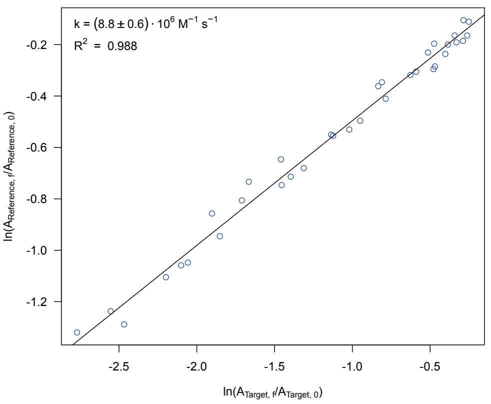

(a) Abacavir - Dibromomethylparaben

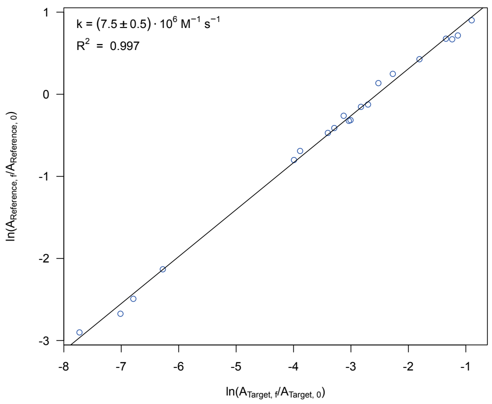

(b) Abacavir-5'-carboxylate - Dibromomethylparaben

Figure SI-B46: Correlations between the natural logarithm of the relative residual peak areas of abacavir (a) and abacavir-5'-carboxylate (b) with competitors upon ozonation at pH 7 (2 mM phosphate), 22 °C, and in presence of *t*BuOH (40 mM). The intercept was considered negligible (<10×slope) and the standard deviations of competitor k_{app,O_3} values were considered in the calculation of the standard deviations of the target k_{app,O_3} values. Note that the axes do not necessarily originate at zero.

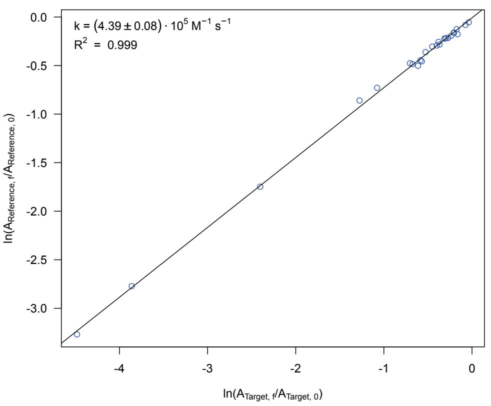

(a) Clindamycin - Carbamazepine

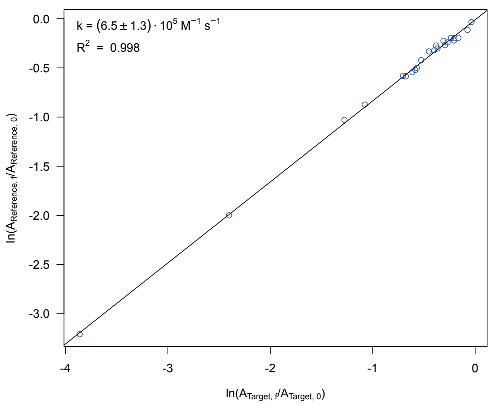

(b) Clindamycin - Trimethoprim

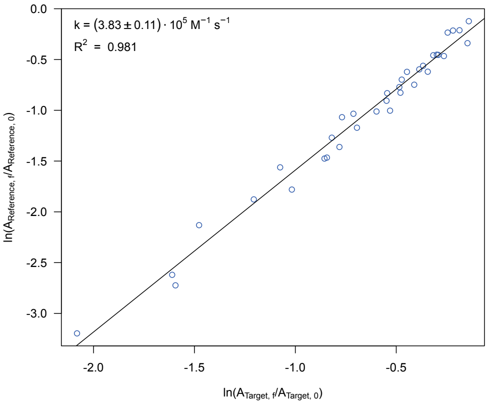

(c) Clindamycin-sulfoxide - Carbamazepine

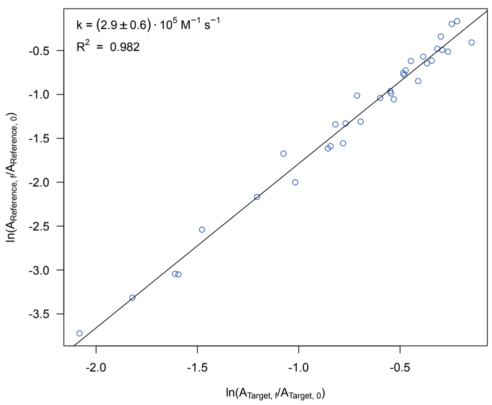

(d) Clindamycin-sulfoxide - Trimethoprim

Figure SI-B47: Correlations between the natural logarithm of the relative residual peak areas of clindamycin (a - b) and clindamycin-sulfoxide (c - d) with competitors upon ozonation at pH 7 (2 mM phosphate), 22 °C, and in presence of *t*BuOH (40 mM). The intercept was considered negligible (<10×slope) and the standard deviations of competitor $k_{\mathrm{app,O_3}}$ values were considered in the calculation of the standard deviations of the target $k_{\mathrm{app,O_3}}$ values. Note that the axes do not necessarily originate at zero.

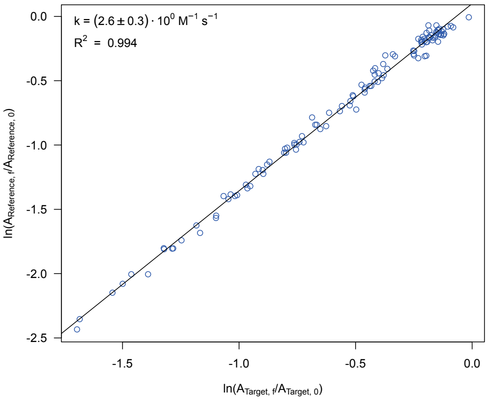

(a) Bisacodyl - Alachlor

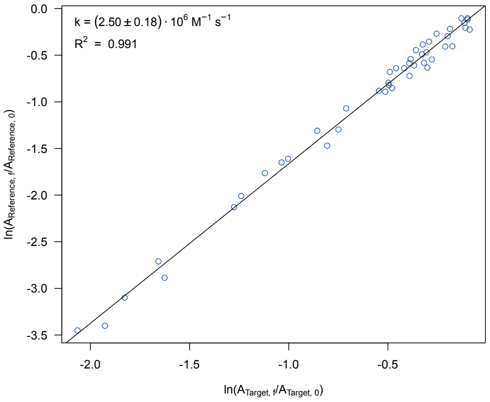

(b) Desacetylbisacodyl - Dibromomethylparaben

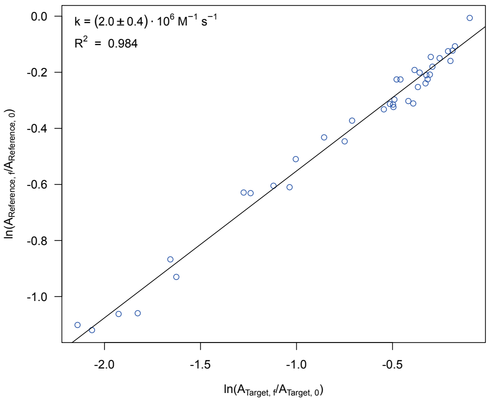

(c) Desacetylbisacodyl - Sulfamethoxazole

Figure SI-B48: Correlations between the natural logarithm of the relative residual peak areas of bisacodyl (a) and desacetylbisacodyl (b) with competitors upon ozonation at pH 7 (2 mM phosphate), 22 °C, and in presence of *t*BuOH (40 mM). The intercept was considered negligible (<10×slope) and the standard deviations of competitor k_{app,O_3} values were considered in the calculation of the standard deviations of the target k_{app,O_3} values. Note that the axes do not necessarily originate at zero.

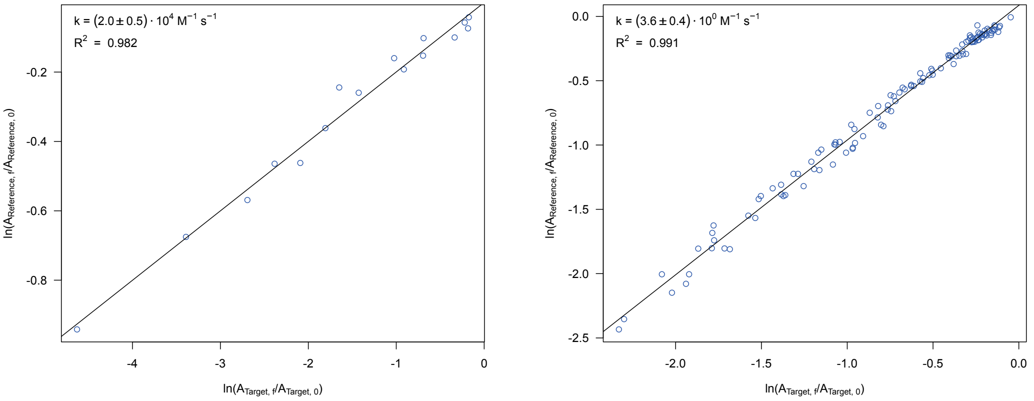

(a) Diphenhydramine - Tramadol

(b) Diphenhydramine-*N*-glucuronide - Alachlor

Figure SI-B49: Correlations between the natural logarithm of the relative residual peak areas of diphenhydramine (a) and diphenhydramine-*N*-glucuronide (b) with competitors upon ozonation at pH 7 (2 mM phosphate), 22 °C, and in presence of *t*BuOH (40 mM). The intercept was considered negligible (<10×slope) and the standard deviations of competitor k_{app,O_3} values were considered in the calculation of the standard deviations of the target k_{app,O_3} values. Note that the axes do not necessarily originate at zero.

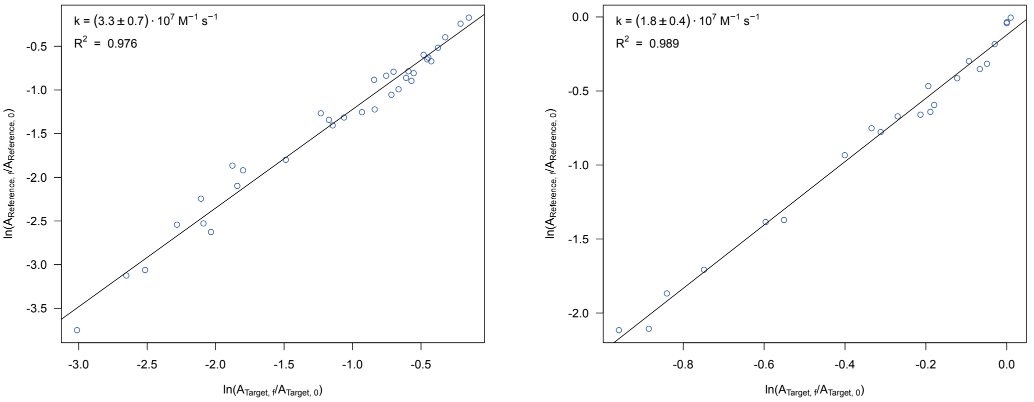

(a) Hesperitin - Triclosan

(b) Eriodictyol - Triclosan

Figure SI-B50: Correlations between the natural logarithm of the relative residual peak areas of hesperitin (a) and eriodictyol (b) with competitors upon ozonation at pH 7 (2 mM phosphate), 22 °C, and in presence of *t*BuOH (40 mM). The intercept was considered negligible (<10×slope) and the standard deviations of competitor k_{app,O_3} values were considered in the calculation of the standard deviations of the target k_{app,O_3} values. Note that the axes do not necessarily originate at zero.

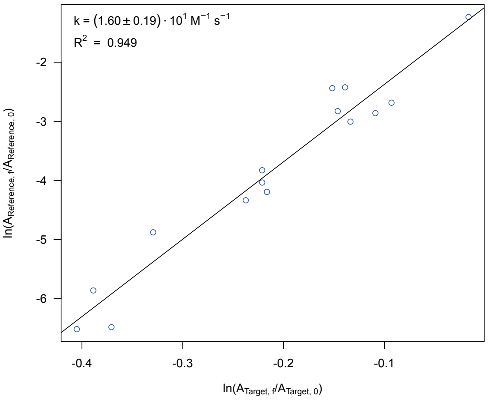

(a) Lamotrigine - Carbofuran

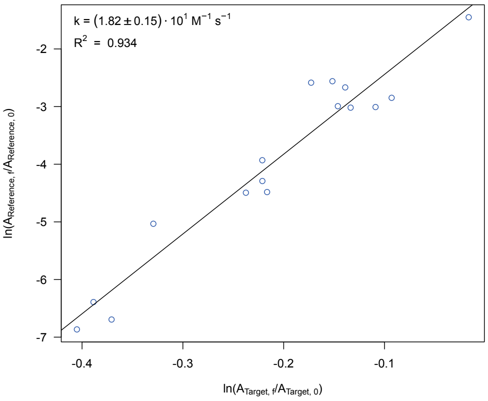

(b) Lamotrigine - N^4-Acetylsulfamethoxazole

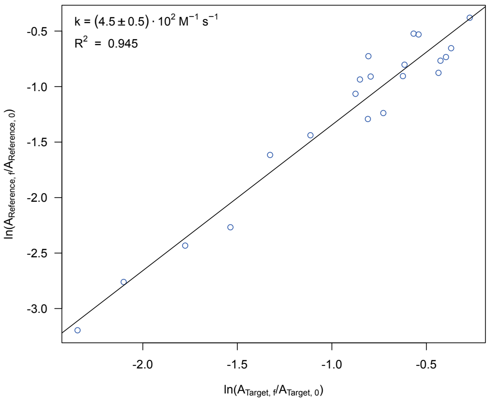

(c) Lamotrigine-N2-glucuronide - Bezafibrate

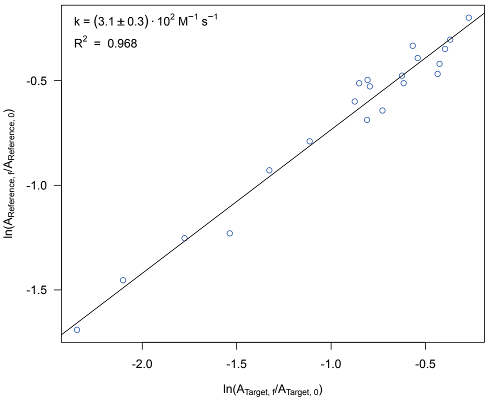

(d) Lamotrigine-N2-glucuronide - Carbofuran

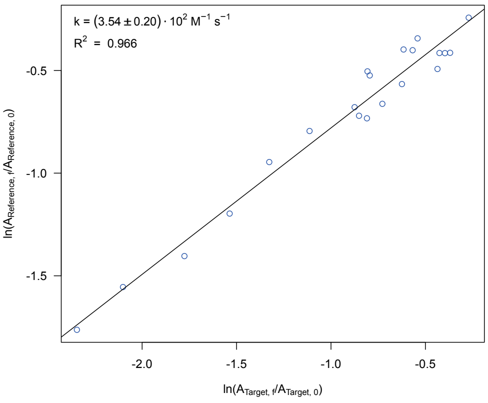

(e) Lamotrigine-N2-glucuronide - N^4-Acetylsulfamethoxazole

Figure SI-B51: Correlations between the natural logarithm of the relative residual peak areas of lamotrigine (a) and lamotirgine-N2-glucuronide (b - d) with competitors upon ozonation at pH 7 (2 mM phosphate), 22 °C, and in presence of *t*BuOH (40 mM). The intercept was considered negligible (<10×slope) and the standard deviations of competitor k_{app,O_3} values were considered in the calculation of the standard deviations of the target k_{app,O_3} values. Note that the axes do not necessarily originate at zero.

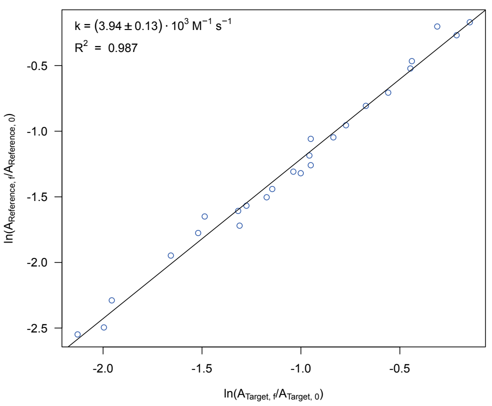

(a) Cocaine - Penicillin G

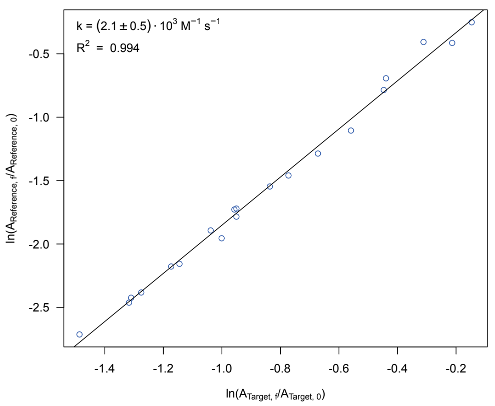

(b) Cocaine - Tramadol

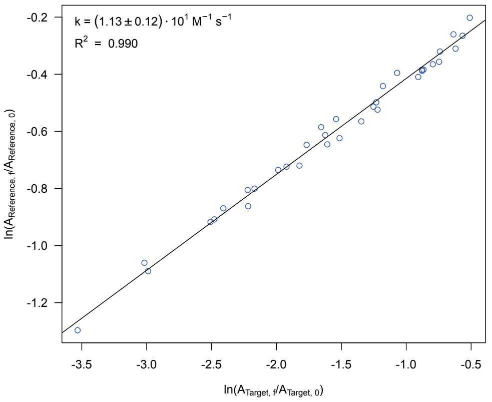

(c) Benzoylecgonine - Alachlor

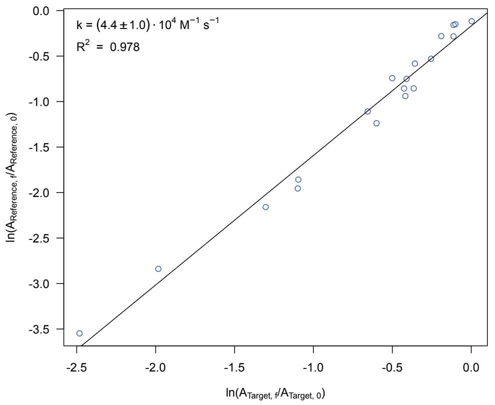

(d) Anhydroecgonine methyl ester - Roxithromycin

Figure SI-B52: Correlations between the natural logarithm of the relative residual peak areas of cocaine (a - b), benzoylecgonine (c) and anhydroecgonine methyl ester (d) with competitors upon ozonation at pH 7 (2 mM phosphate), 22 °C, and in presence of *t*BuOH (40 mM). The intercept was considered negligible (<10×slope) and the standard deviations of competitor k_{app,O_3} values were considered in the calculation of the standard deviations of the target k_{app,O_3} values. Note that the axes do not necessarily originate at zero.

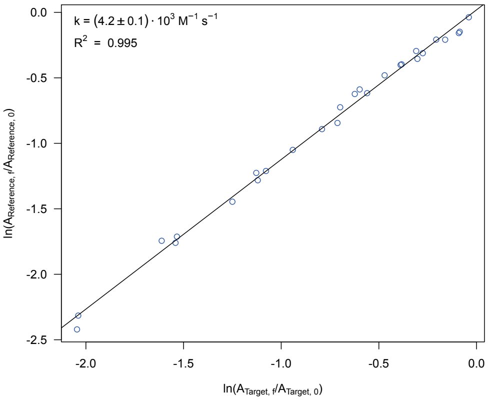

(a) Rosuvastatin - Penicillin G

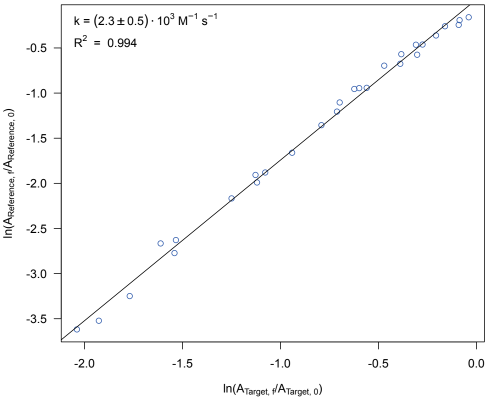

(b) Rosuvastatin - Tramadol

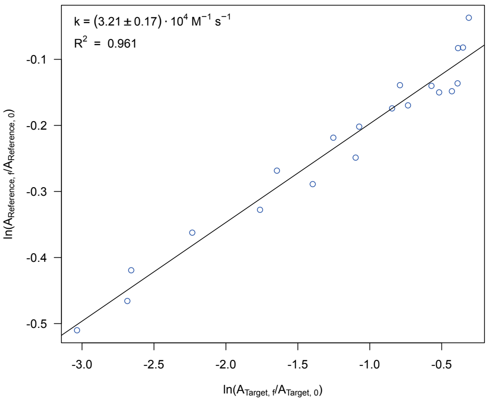

(c) *N*-Desmethylrosuvastatin - Penicillin G

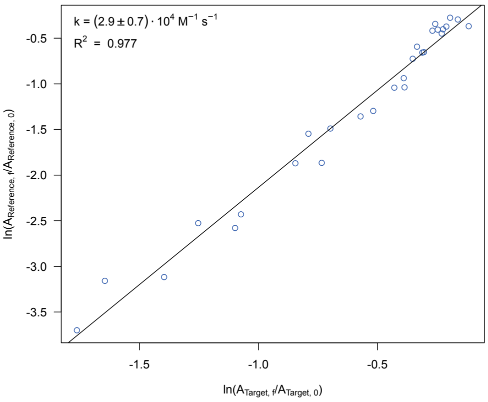

(d) *N*-Desmethylrosuvastatin - Roxithromycin

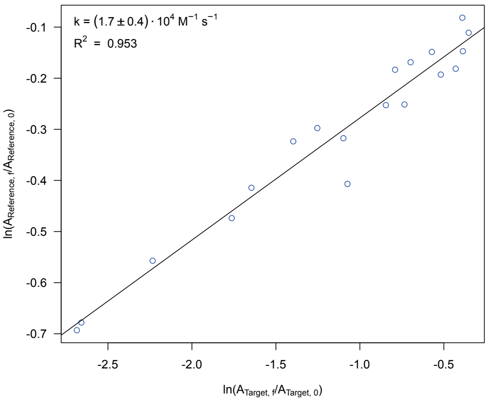

(e) *N*-Desmethylrosuvastatin - Tramadol

Figure SI-B53: Correlations between the natural logarithm of the relative residual peak areas of rosuvastatin (a - b) and *N*-desmethylrosuvastatin (c - e) with competitors upon ozonation at pH 7 (2 mM phosphate), 22 °C, and in presence of *t*BuOH (40 mM). The intercept was considered negligible (<10×slope) and the standard deviations of competitor $k_{\mathrm{app,O_3}}$ values were considered in the calculation of the standard deviations of the target $k_{\mathrm{app,O_3}}$ values. Note that the axes do not necessarily originate at zero.

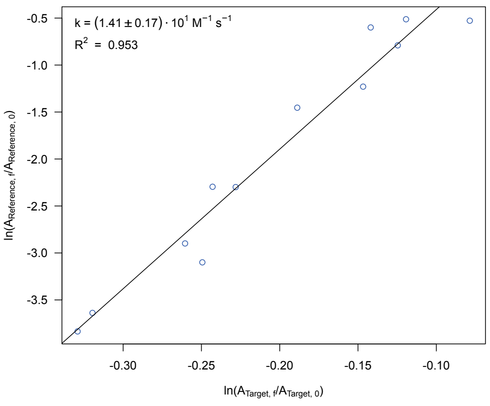

(a) Pregabalin - Carbofuran

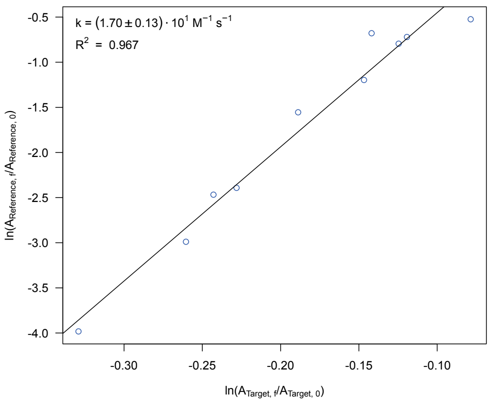

(b) Pregabalin - N^4-Acetylsulfamethoxazole

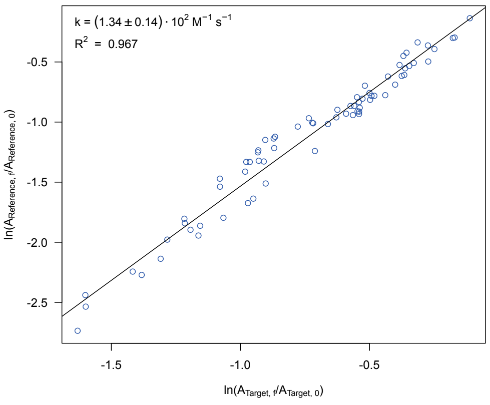

(c) *N*-Methylpregabalin - Carbofuran

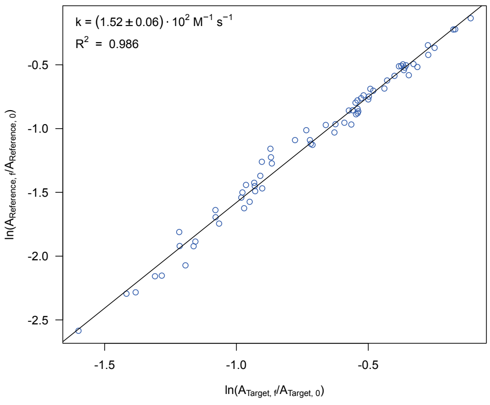

(d) *N*-Methylpregabalin - N^4-Acetylsulfamethoxazole

Figure SI-B54: Correlations between the natural logarithm of the relative residual peak areas of pregabalin (a - b) and *N*-methylpregabalin (c - d) with competitors upon ozonation at pH 7 (2 mM phosphate), 22 °C, and in presence of *t*BuOH (40 mM). The intercept was considered negligible (<10×slope) and the standard deviations of competitor $k_{\mathrm{app,O_3}}$ values were considered in the calculation of the standard deviations of the target $k_{\mathrm{app,O_3}}$ values. Note that the axes do not necessarily originate at zero.

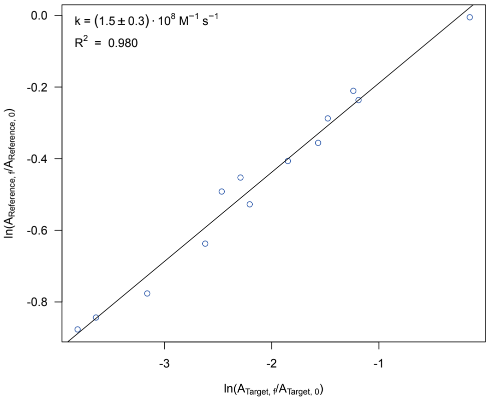

(a) Atorvastatin - Triclosan

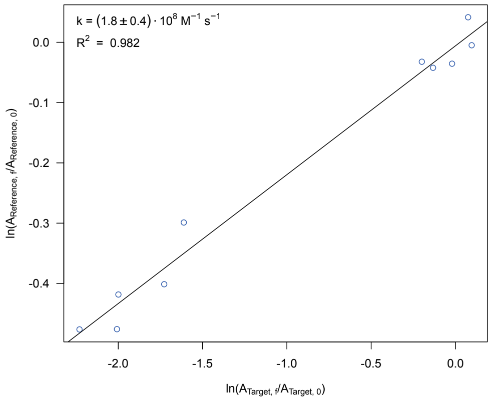

(b) ortho-Hydroxyatorvastatin - Triclosan

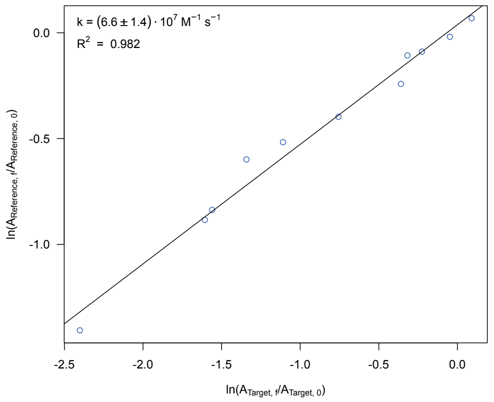

(c) para-Hydroxyatrivastatin - Triclosan

Figure SI-B55: Correlations between the natural logarithm of the relative residual peak areas of atorvastatin (a), ortho-hydroxyatorvastatin (b) and para-hydroxyatrovastatin (c) with competitors upon ozonation at pH 7 (2 mM phosphate), 22 °C, and in presence of tBuOH (40 mM). The intercept was considered negligible (<10×slope) and the standard deviations of competitor $k_{\mathrm{app,O_3}}$ values were considered in the calculation of the standard deviations of the target $k_{\mathrm{app,O_3}}$ values. Note that the axes do not necessarily originate at zero.

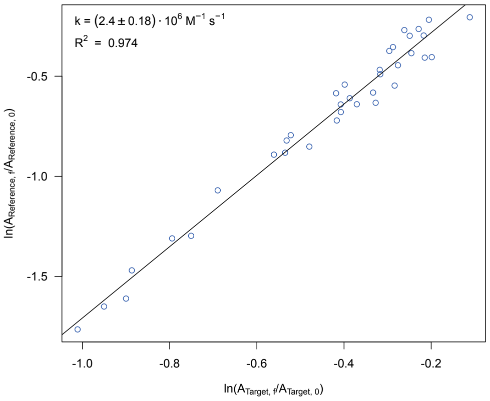

(a) Paracetamol - Dibromomethylparaben

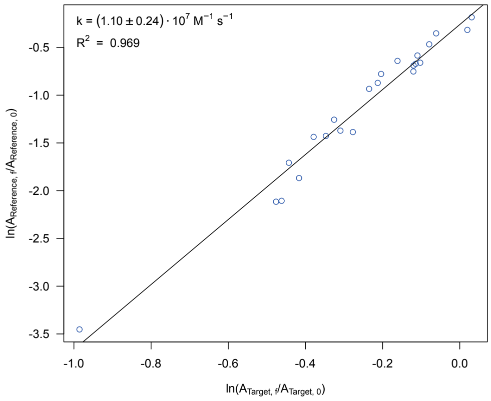

(b) Paracetamol-glutathione - Triclosan

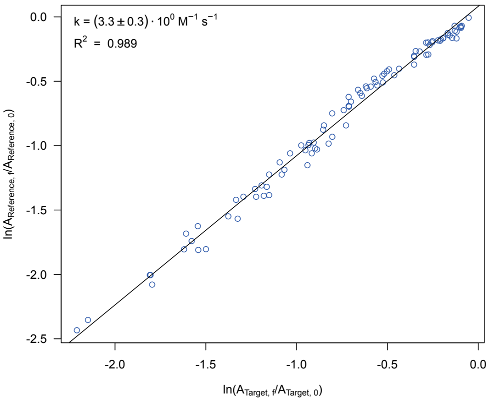

(c) Paracetamol-sulfate - Alachlor

Figure SI-B56: Correlations between the natural logarithm of the relative residual peak areas of paracetamol (a), paracetamol-glutathione (b) and paracetamol-sulfate (c) with competitors upon ozonation at pH 7 (2 mM phosphate), 22 °C, and in presence of *t*BuOH (40 mM). The intercept was considered negligible (<10×slope) and the standard deviations of competitor $k_{\mathrm{app,O_3}}$ values were considered in the calculation of the standard deviations of the target $k_{\mathrm{app,O_3}}$ values. Note that the axes do not necessarily originate at zero.

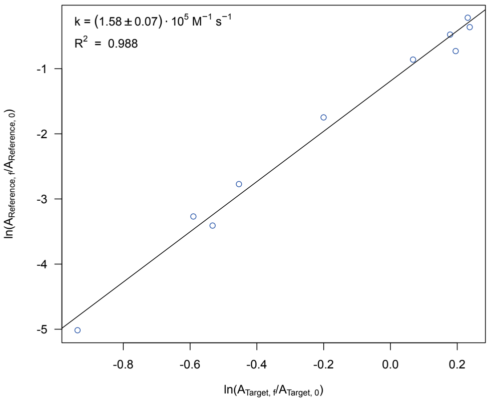

(a) Phenylephrine - Carbamazepine

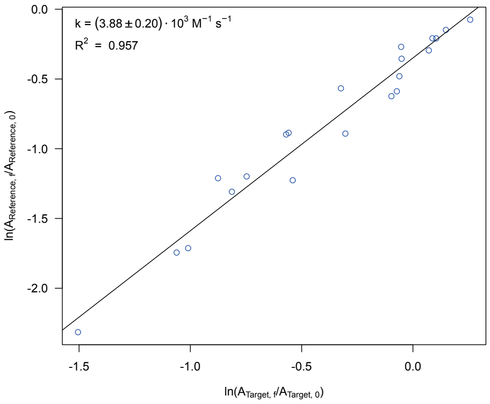

(b) Phenylephrine-3-*O*-sulfate - Penicillin G

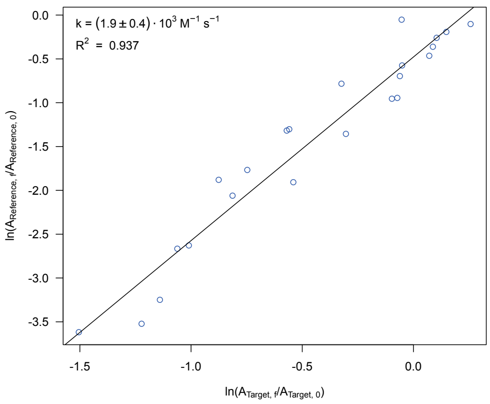

(c) Phenylephrine-3-*O*-sulfate - Tramadol

Figure SI-B57: Correlations between the natural logarithm of the relative residual peak areas of phenylephrine (a) and phenylephrine-3-*O*-sulfate (b - c) with competitors upon ozonation at pH 7 (2 mM phosphate), 22 °C, and in presence of *t*BuOH (40 mM). The intercept was considered negligible (<10×slope) and the standard deviations of competitor $k_{\mathrm{app,O_3}}$ values were considered in the calculation of the standard deviations of the target $k_{\mathrm{app,O_3}}$ values. Note that the axes do not necessarily originate at zero.

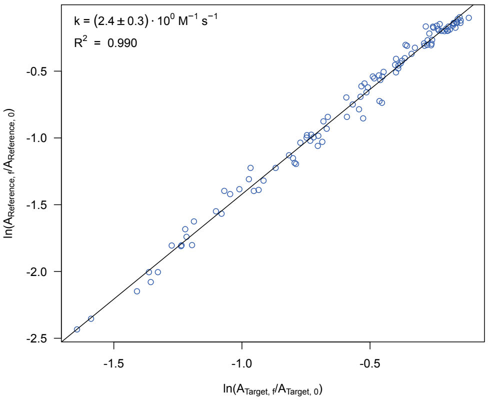

(a) Primidone - Alachlor

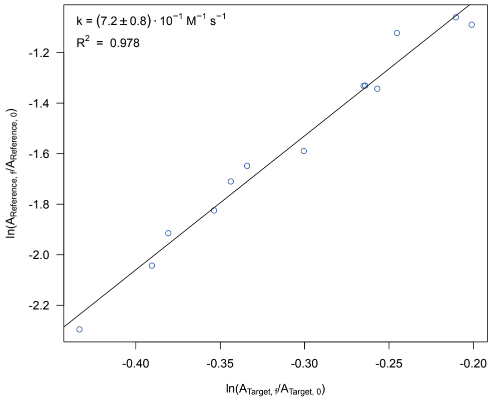

(b) PEMA - Alachlor

Figure SI-B58: Correlations between the natural logarithm of the relative residual peak areas of primidone (a) and PEMA (b) with competitors upon ozonation at pH 7 (2 mM phosphate), 22 °C, and in presence of *t*BuOH (40 mM). The intercept was considered negligible (<10×slope) and the standard deviations of competitor $k_{\mathrm{app,O_3}}$ values were considered in the calculation of the standard deviations of the target $k_{\mathrm{app,O_3}}$ values. Note that the axes do not necessarily originate at zero.

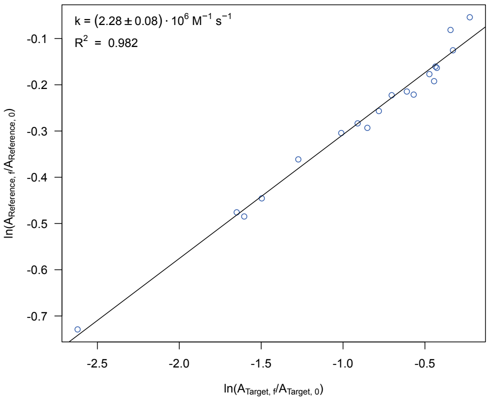

(a) Tapentadol - Carbamazepine

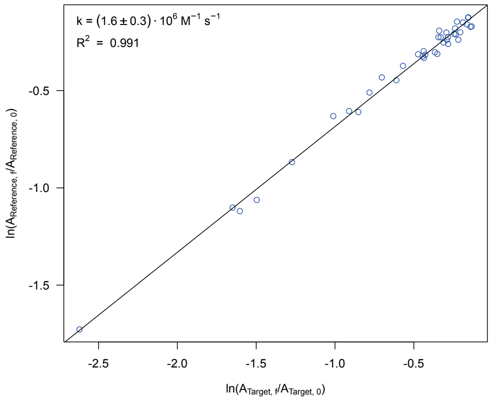

(b) Tapentadol - Sulfamethoxazole

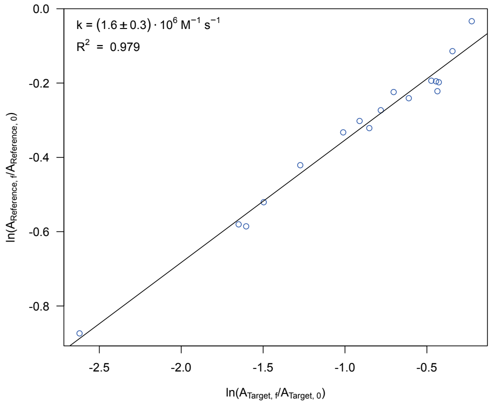

(c) Tapentadol - Trimethoprim

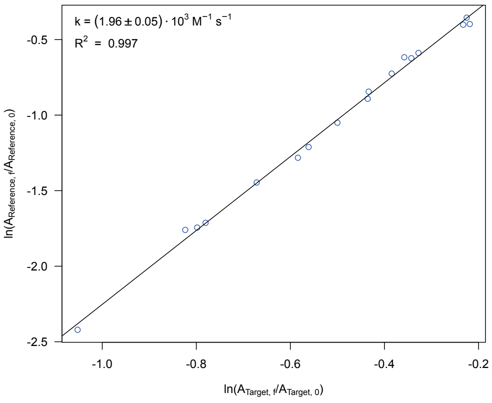

(d) Tapentadol-*O*-sulfate - Penicillin G

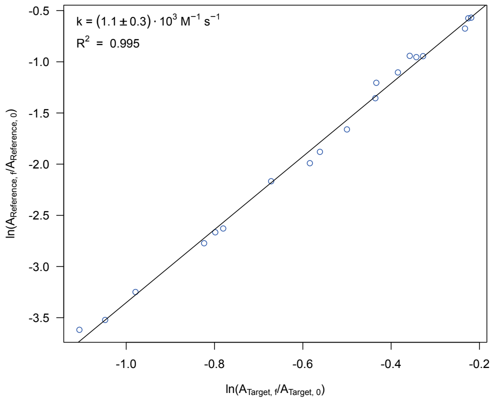

(e) Tapentadol-*O*-sulfate - Tramadol

Figure SI-B59: Correlations between the natural logarithm of the relative residual peak areas of tapentadol (a - c) and tapentadol-*O*-sulfate (d - e) with competitors upon ozonation at pH 7 (2 mM phosphate), 22 °C, and in presence of *t*BuOH (40 mM). The intercept was considered negligible (<10×slope) and the standard deviations of competitor k_{app,O_3} values were considered in the calculation of the standard deviations of the target k_{app,O_3} values. Note that the axes do not necessarily originate at zero.

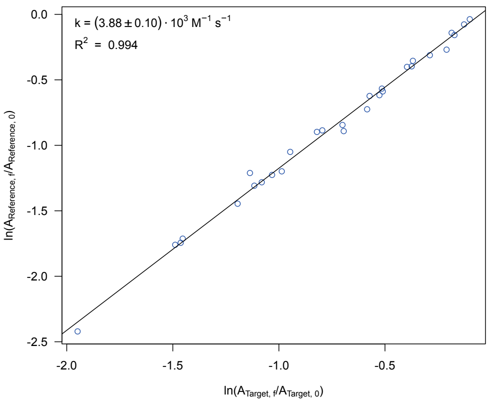

(a) Torasemide - Penicillin G

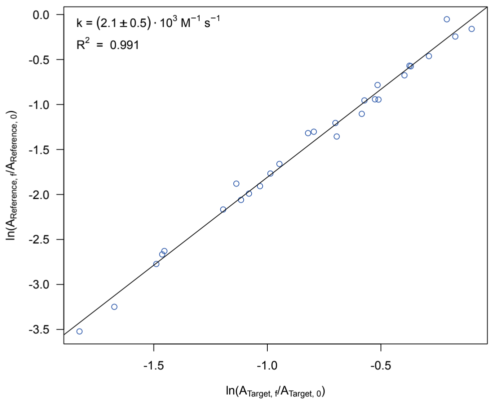

(b) Torasemide - Tramadol

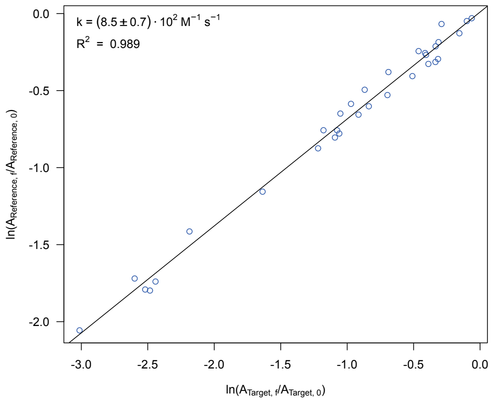

(c) Hydroxytorasemide - Bezafibrate

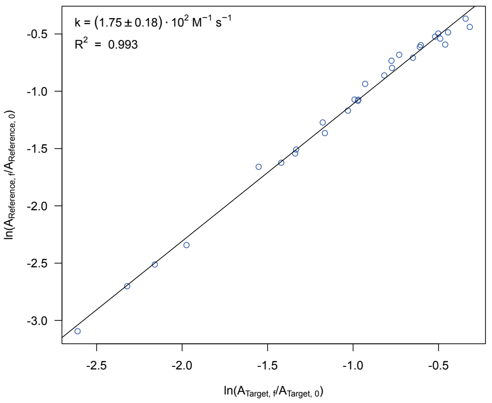

(d) Torasemide carboxylic acid - Carbofuran

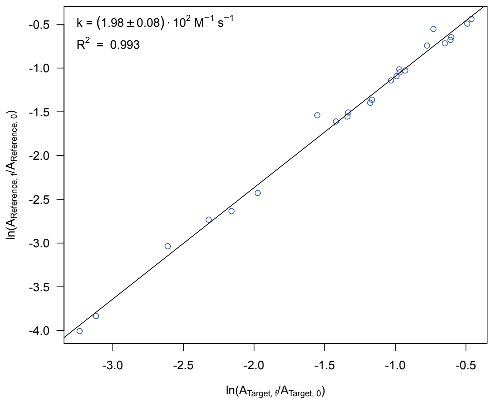

(e) Torasemide carboxylic acid - N^4-Acetylsulfamethoxazole

Figure SI-B60: Correlations between the natural logarithm of the relative residual peak areas of torasemide (a - b), hydroxytorasemide (c) and torasemide carboxylic acid (d - e) with competitors upon ozonation at pH 7 (2 mM phosphate), 22 °C, and in presence of *t*BuOH (40 mM). The intercept was considered negligible (<10×slope) and the standard deviations of competitor k_{app,O_3} values were considered in the calculation of the standard deviations of the target k_{app,O_3} values. Note that the axes do not necessarily originate at zero.

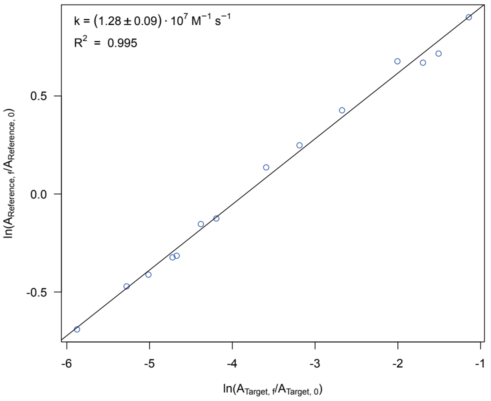

(a) Zolpidem - Dibromomethylparaben

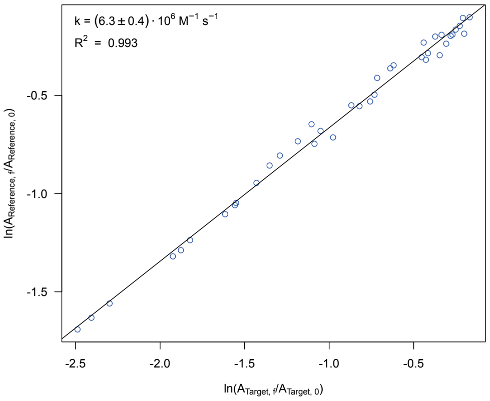

(b) Zolpidem carboxylic acid - Dibromomethylparaben

Figure SI-B61: Correlations between the natural logarithm of the relative residual peak areas of zolpidem (a) and zolpidem carboxylic acid (b) with competitors upon ozonation at pH 7 (2 mM phosphate), 22 °C, and in presence of *t*BuOH (40 mM). The intercept was considered negligible (<10×slope) and the standard deviations of competitor $k_{\mathrm{app,O_3}}$ values were considered in the calculation of the standard deviations of the target $k_{\mathrm{app,O_3}}$ values. Note that the axes do not necessarily originate at zero.

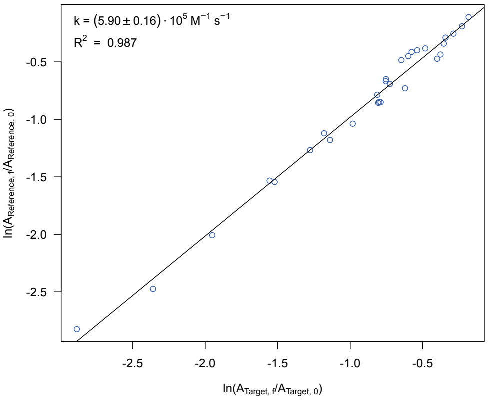

(a) Naproxen - Carbamazepine

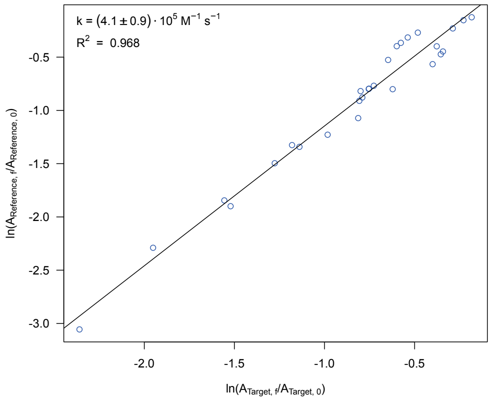

(b) Naproxen - Trimethoprim

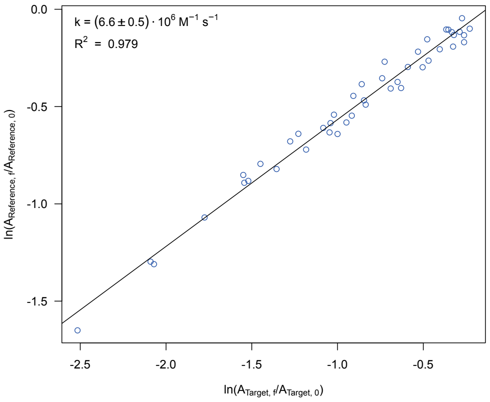

(c) *O*-Desmethylnaproxen - Dibromomethylparaben

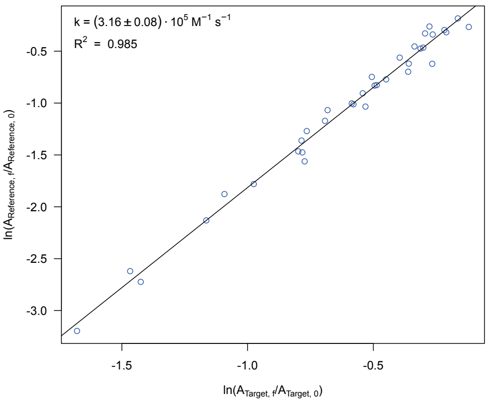

(d) Naproxen methyl ester - Carbamazepine

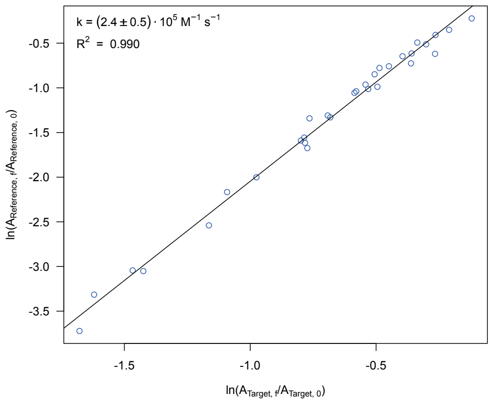

(e) Naproxen methyl ester - Trimethoprim

Figure SI-B62: Correlations between the natural logarithm of the relative residual peak areas of naproxen (a - b), *O*-desmethylnaproxen (c) and naproxen methyl ester (d - e) with competitors upon ozonation at pH 7 (2 mM phosphate), 22 °C, and in presence of *t*BuOH (40 mM). The intercept was considered negligible (<10×slope) and the standard deviations of competitor $k_{\mathrm{app,O_3}}$ values were considered in the calculation of the standard deviations of the target $k_{\mathrm{app,O_3}}$ values. Note that the axes do not necessarily originate at zero.

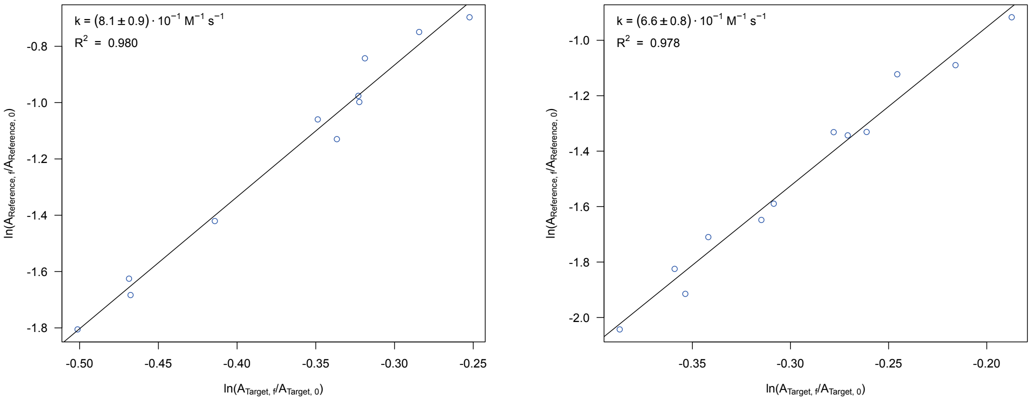

(a) Levetiracetam - Alachlor

(b) Levetiracetam acid - Alachlor

Figure SI-B63: Correlations between the natural logarithm of the relative residual peak areas of levetiracetam (a) and levetiracetam acid (b) with competitors upon ozonation at pH 7 (2 mM phosphate), 22 °C, and in presence of *t*BuOH (40 mM). The intercept was considered negligible (<10×slope) and the standard deviations of competitor $k_{\mathrm{app,O_3}}$ values were considered in the calculation of the standard deviations of the target $k_{\mathrm{app,O_3}}$ values. Note that the axes do not necessarily originate at zero.

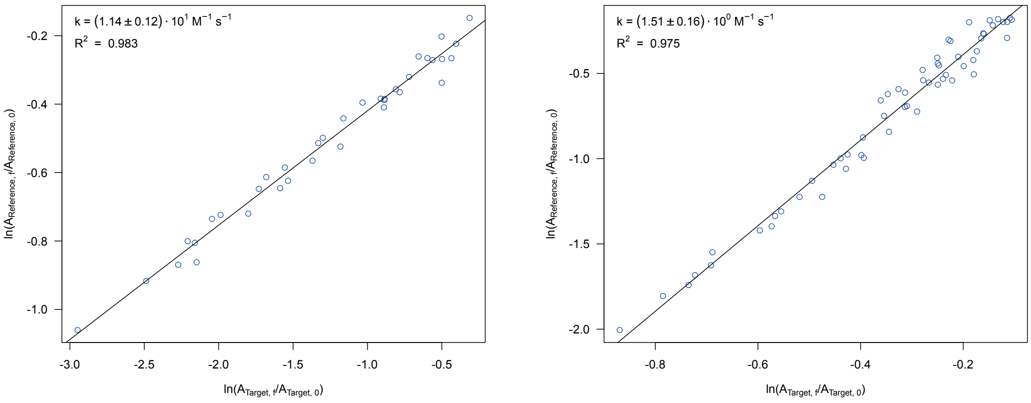

(a) Gabapentin - Alachlor

(b) Gabapentin-lactam - Alachlor

Figure SI-B64: Correlations between the natural logarithm of the relative residual peak areas of gabapentin (a) and gabapentin-lactam (b) with competitors upon ozonation at pH 7 (2 mM phosphate), 22 °C, and in presence of *t*BuOH (40 mM). The intercept was considered negligible (<10×slope) and the standard deviations of competitor $k_{\mathrm{app,O_3}}$ values were considered in the calculation of the standard deviations of the target $k_{\mathrm{app,O_3}}$ values. Note that the axes do not necessarily originate at zero.

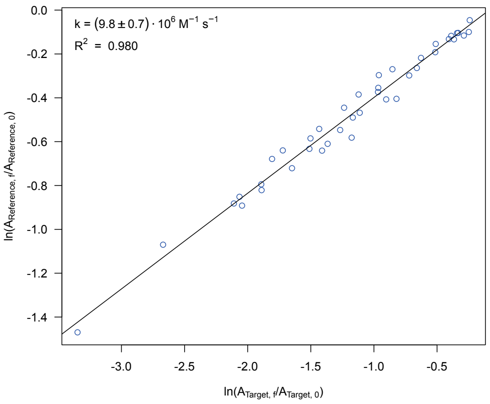

(a) Sulfathiazole - Dibromomethylparaben

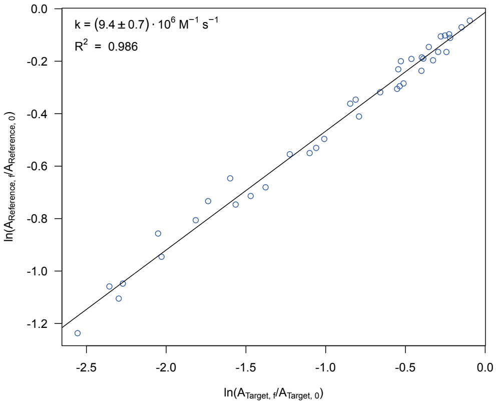

(b) N^4-Acetylsulfathiazole - Dibromomethylparaben

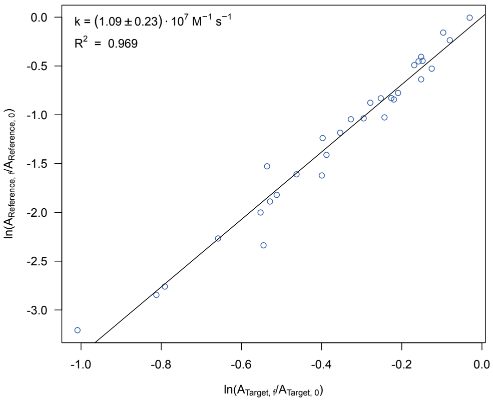

(c) N^4-Acetylsulfathiazole - Triclosan

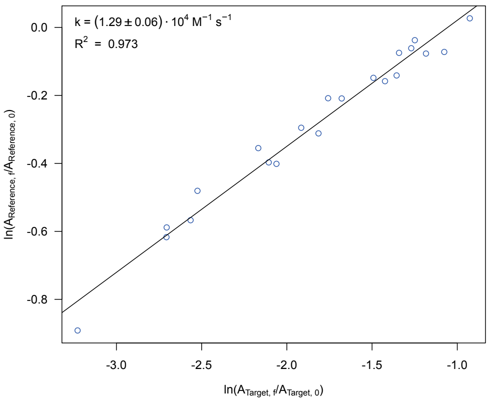

(d) Pterin-sulfathiazole - Penicillin G

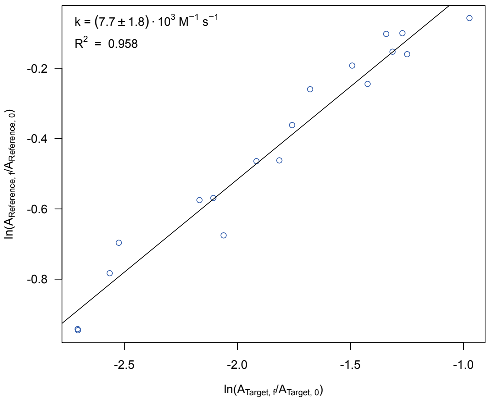

(e) Pterin-sulfathiazole - Tramadol

Figure SI-B65: Correlations between the natural logarithm of the relative residual peak areas of sulfathiazole (a), N^4-acetylsulfathiazole (b - c) and pterin-sulfathiazole (d - e) with competitors upon ozonation at pH 7 (2 mM phosphate), 22 °C, and in presence of *t*BuOH (40 mM). The intercept was considered negligible (<10×slope) and the standard deviations of competitor $k_{\mathrm{app,O_3}}$ values were considered in the calculation of the standard deviations of the target $k_{\mathrm{app,O_3}}$ values. Note that the axes do not necessarily originate at zero.

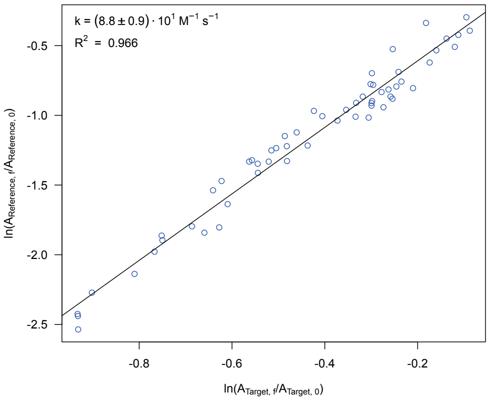

(a) Benzotriazole - Carbofuran

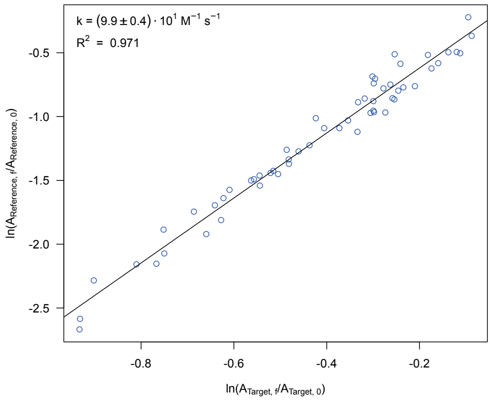

(b) Benzotriazole - N^4-Acetylsulfamethoxazole

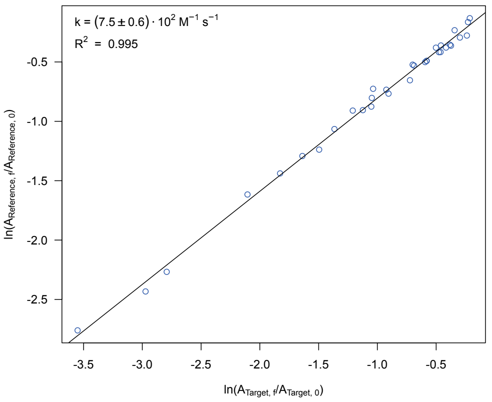

(c) 4-Methylbenzotriazole - Bezafibrate

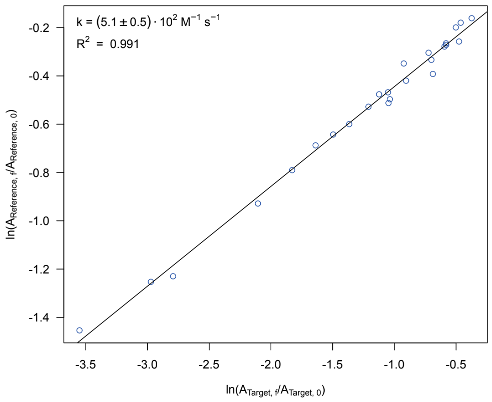

(d) 4-Methylbenzotriazole - Carbofuran

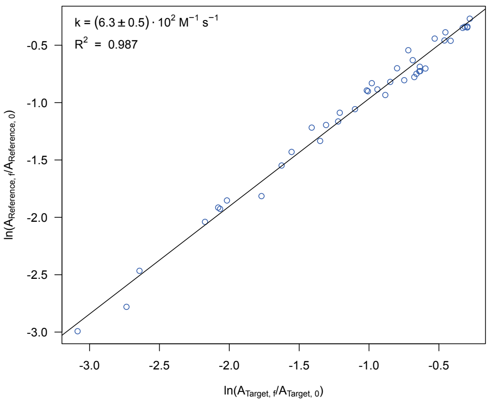

(e) 5-Methylbenzotriazole - Bezafibrate

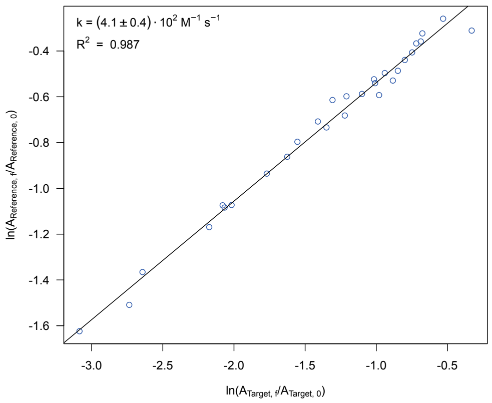

(f) 5-Methylbenzotriazole - Carbofuran

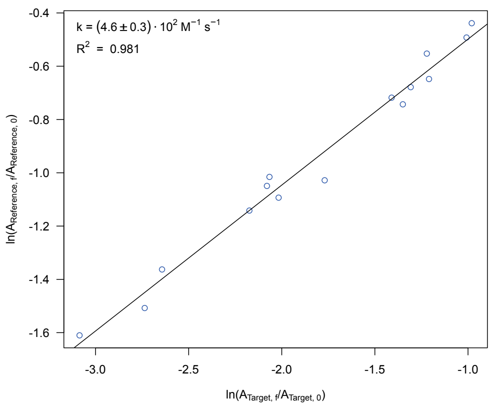

(g) 5-Methylbenzotriazole - N^4-Acetylsulfamethoxazole

Figure SI-B65: Correlations between the natural logarithm of the relative residual peak areas of benzotriazole (a - b), 4-methylbenzotriazole (c - d) and 5-methylbenzotriazole (e - f) with competitors upon ozonation at pH 7 (2 mM phosphate), 22 °C, and in presence of *t*BuOH (40 mM). The intercept was considered negligible (<10×slope) and the standard deviations of competitor k_{app,O_3} values were considered in the calculation of the standard deviations of the target k_{app,O_3} values. Note that the axes do not necessarily originate at zero.

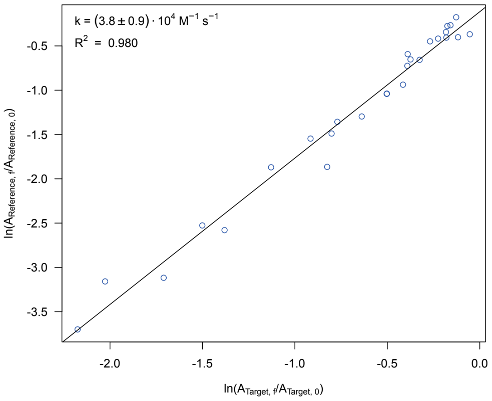

(h) Azoxystrobin - Roxithromycin

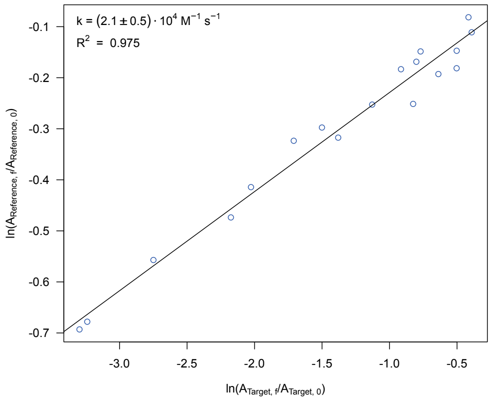

(i) Azoxystrobin - Tramadol

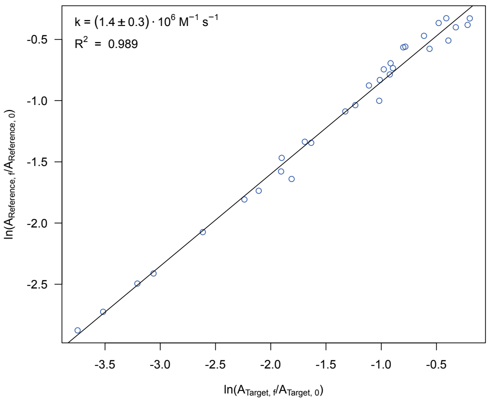

(j) Azoxystrobin acid - Sulfamethoxazole

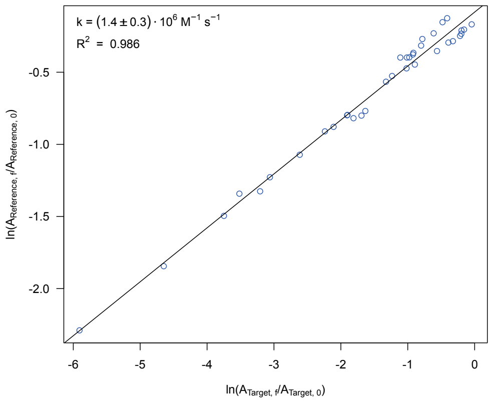

(k) Azoxystrobin acid - Trimethoprim

Figure SI-B66: Correlations between the natural logarithm of the relative residual peak areas of azoxystrobin (a) and azoxystrobin acid (b - c) with competitors upon ozonation at pH 7 (2 mM phosphate), 22 °C, and in presence of *t*BuOH (40 mM). The intercept was considered negligible (<10×slope) and the standard deviations of competitor $k_{\mathrm{app,O_3}}$ values were considered in the calculation of the standard deviations of the target $k_{\mathrm{app,O_3}}$ values. Note that the axes do not necessarily originate at zero.

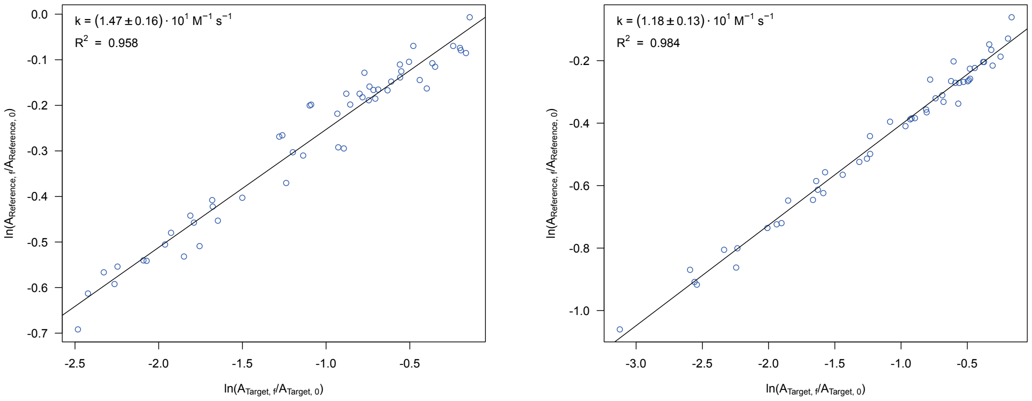

(a) 1-Hydroxyibuprofen - Alachlor

(b) Carboxyibuprofen - Alachlor

Figure SI-B67: Correlations between the natural logarithm of the relative residual peak areas of 1-hydroxyibuprofen (a) and caroxyibuprofen (b) with competitors upon ozonation at pH 7 (2 mM phosphate), 22 °C, and in presence of *t*BuOH (40 mM). The intercept was considered negligible (<10×slope) and the standard deviations of competitor $k_{\mathrm{app,O_3}}$ values were considered in the calculation of the standard deviations of the target $k_{\mathrm{app,O_3}}$ values. Note that the axes do not necessarily originate at zero.

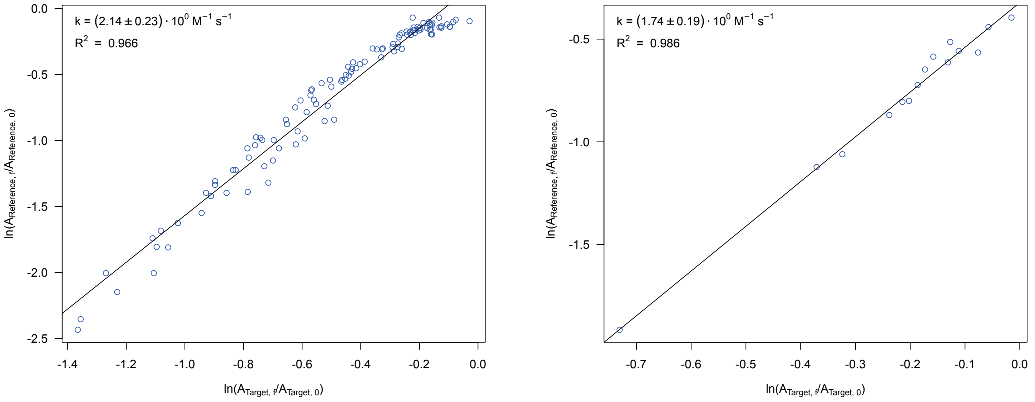

(a) Iopromide - Alachlor

(b) Desmethoxyiopromide - Alachlor

Figure SI-B68: Correlations between the natural logarithm of the relative residual peak areas of iopromide (a) and desmethoxyiopromide (b) with competitors upon ozonation at pH 7 (2 mM phosphate), 22 °C, and in presence of *t*BuOH (40 mM). The intercept was considered negligible (<10×slope) and the standard deviations of competitor $k_{\mathrm{app,O_3}}$ values were considered in the calculation of the standard deviations of the target $k_{\mathrm{app,O_3}}$ values. Note that the axes do not necessarily originate at zero.

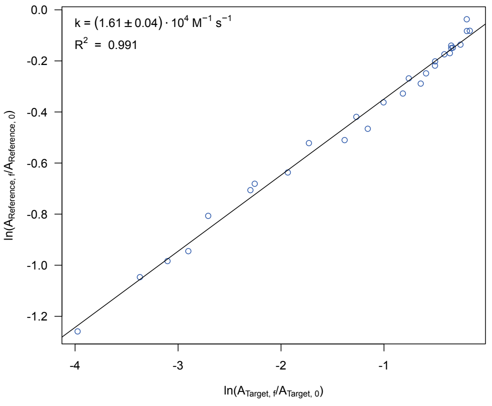

(a) Ketamine - Penicillin G

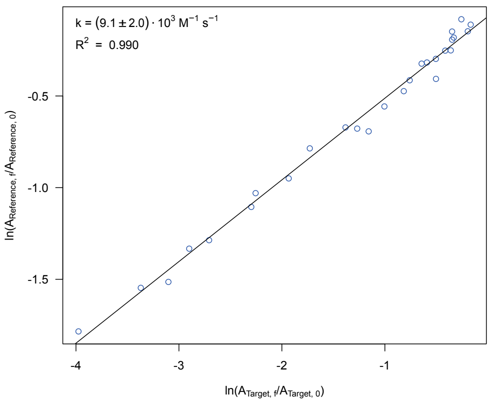

(b) Ketamine - Tramadol

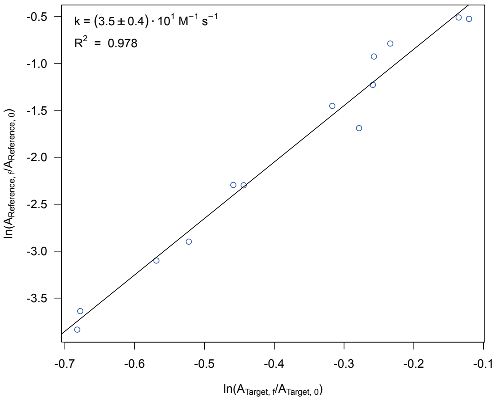

(c) Norketamine - Carbofuran

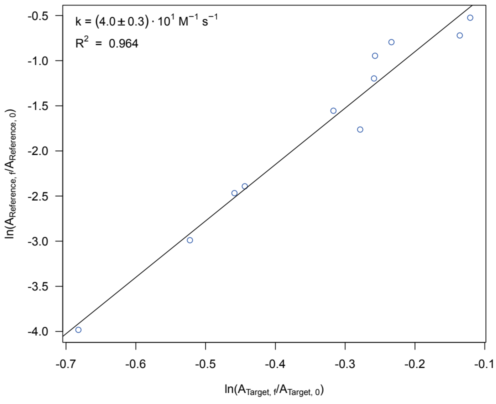

(d) Norketamine - N^4-Acetylsulfamethoxazole

Figure SI-B69: Correlations between the natural logarithm of the relative residual peak areas of ketamine (a - b) and norketamine (c - d) with competitors upon ozonation at pH 7 (2 mM phosphate), 22 °C, and in presence of *t*BuOH (40 mM). The intercept was considered negligible (<10×slope) and the standard deviations of competitor $k_{\mathrm{app,O_3}}$ values were considered in the calculation of the standard deviations of the target $k_{\mathrm{app,O_3}}$ values. Note that the axes do not necessarily originate at zero.

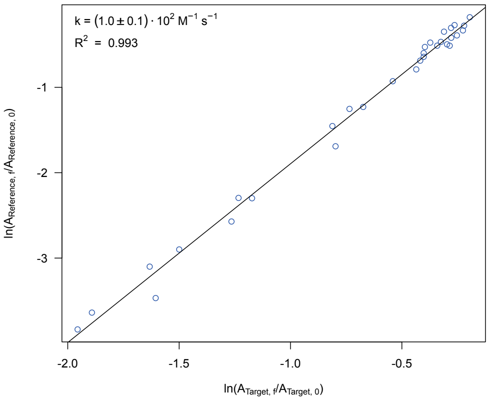

(a) Pantoprazole - Carbofuran

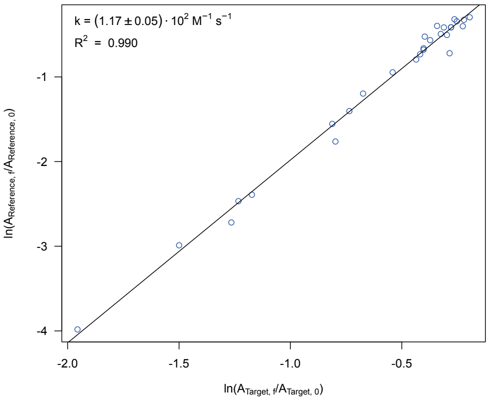

(b) Pantoprazole - N^4-Acetylsulfamethoxazole

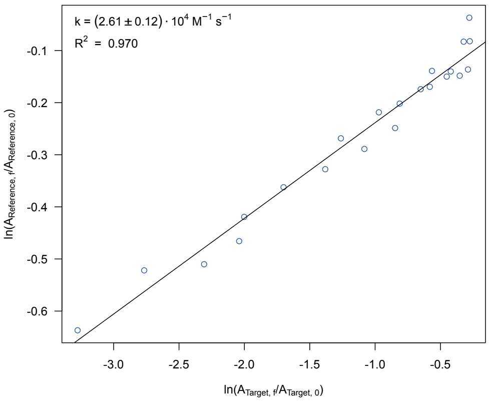

(c) 4-*O*-Desmethylpantoprazole-sulfide - Penicllin G

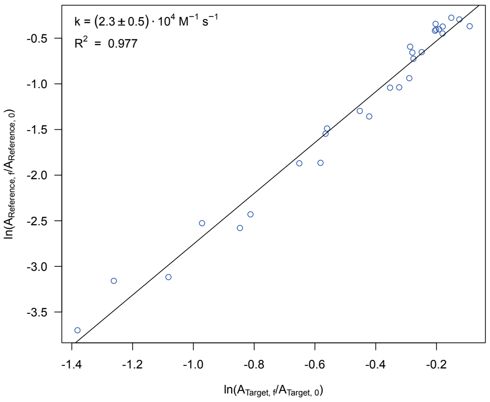

(d) 4-*O*-Desmethylpantoprazole-sulfide - Roxithromycin

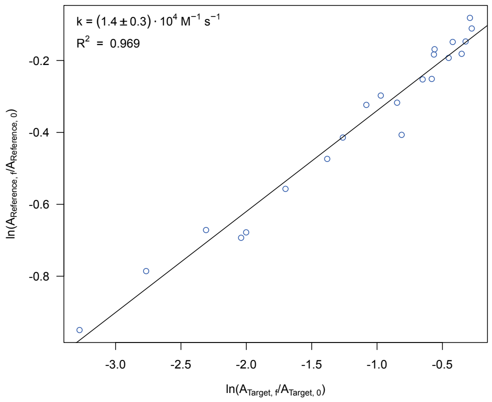

(e) 4-*O*-Desmethylpantoprazole-sulfide - Tramadol

Figure SI-B70: Correlations between the natural logarithm of the relative residual peak areas of pantoprazole (a - b) and 4-*O*-desmethylpantoprazole-sulfide (c - e) with competitors upon ozonation at pH 7 (2 mM phosphate), 22 °C, and in presence of *t*BuOH (40 mM). The intercept was considered negligible (<10×slope) and the standard deviations of competitor k_{app,O_3} values were considered in the calculation of the standard deviations of the target k_{app,O_3} values. Note that the axes do not necessarily originate at zero.

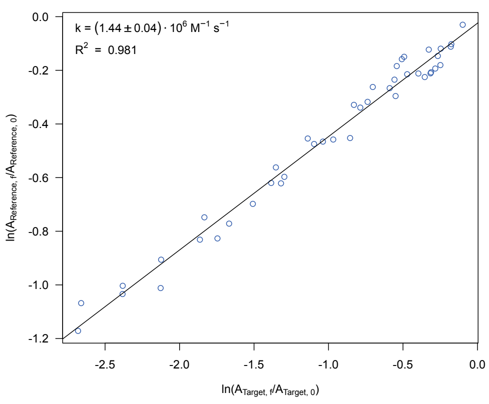

(a) Sulfadiazine - Carbamazepine

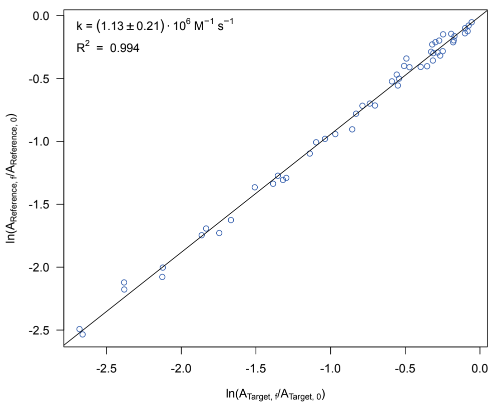

(b) Sulfadiazine - Sulfamethoxazole

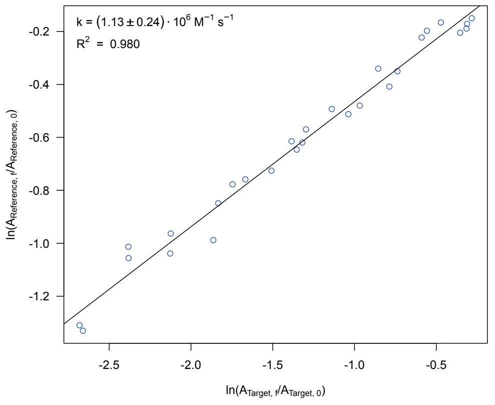

(c) Sulfadiazine - Trimethoprim

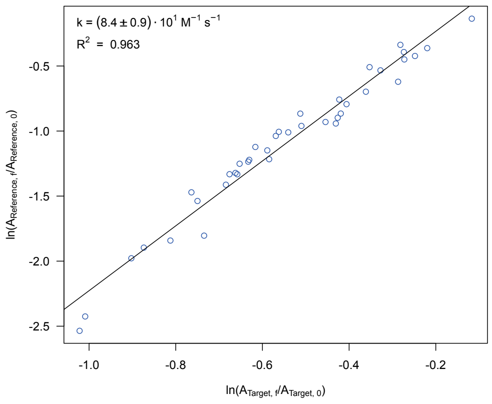

(d) N^4-Acetylsulfadiazine - Carbofuran

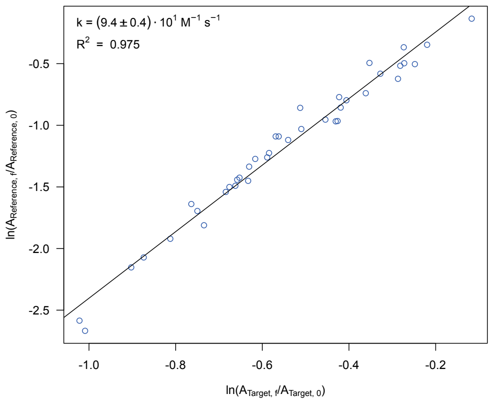

(e) N^4-Acetylsulfadiazine - N^4-Acetylsulfamethoxazole

Figure SI-B71: Correlations between the natural logarithm of the relative residual peak areas of sulfadiazine (a - c) and N^4-acetylsulfamethoxazole (d - e) with competitors upon ozonation at pH 7 (2 mM phosphate), 22 °C, and in presence of *t*BuOH (40 mM). The intercept was considered negligible (<10×slope) and the standard deviations of competitor $k_{\mathrm{app,O_3}}$ values were considered in the calculation of the standard deviations of the target $k_{\mathrm{app,O_3}}$ values. Note that the axes do not necessarily originate at zero.

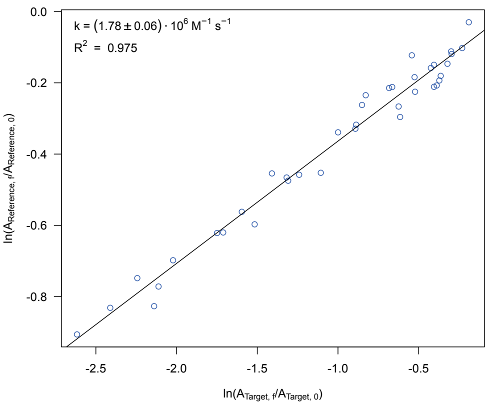

(a) Sulfadimethoxine - Carbamazepine

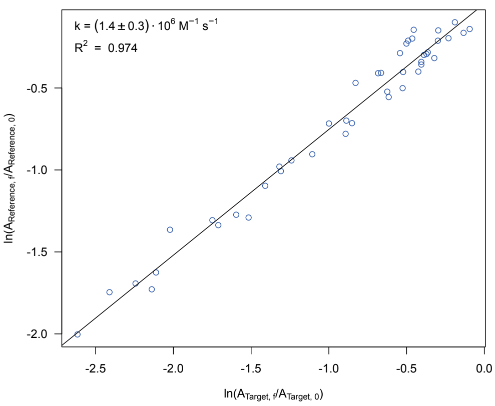

(b) Sulfadimethoxine - Sulfamethoxazole

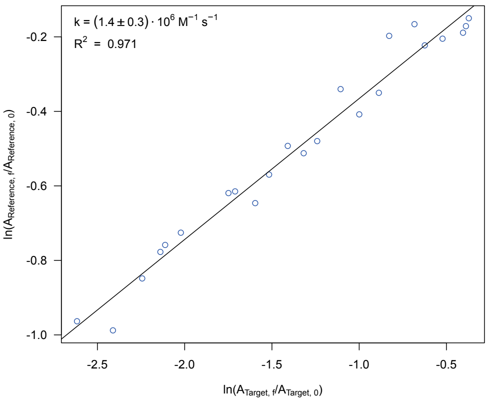

(c) Sulfadimethoxine - Trimethoprim

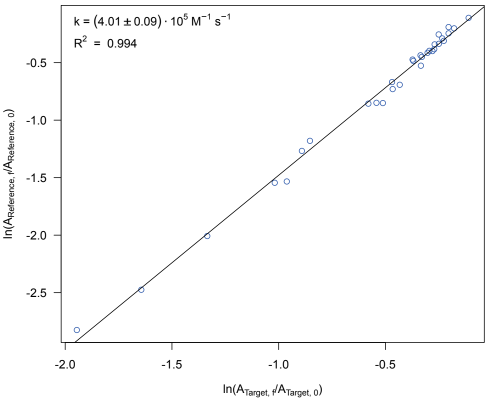

(d) N^4-Acetylsulfadimethoxine - Carbamazepine

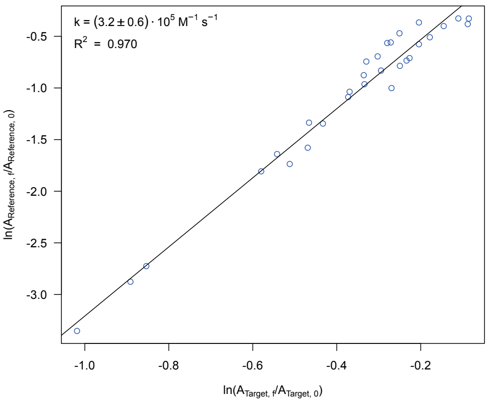

(e) N^4-Acetylsulfadimethoxine - Sulfamethoxazole

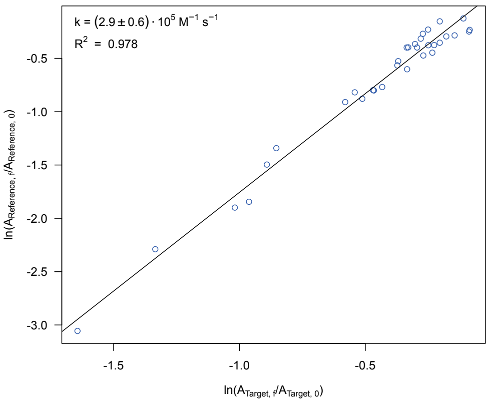

(f) N^4-Acetylsulfadimethoxine - Trimethoprim

Figure SI-B72: Correlations between the natural logarithm of the relative residual peak areas of sulfadimethoxine (a - c) and N^4-acetylsulfadimethoxine (d - f) with competitors upon ozonation at pH 7 (2 mM phosphate), 22 °C, and in presence of *t*BuOH (40 mM). The intercept was considered negligible (<10×slope) and the standard deviations of competitor $k_{\mathrm{app,O_3}}$ values were considered in the calculation of the standard deviations of the target $k_{\mathrm{app,O_3}}$ values. Note that the axes do not necessarily originate at zero.

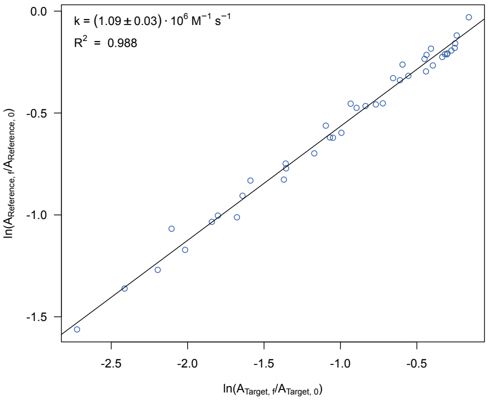

(a) Sulfamethazine - Carbamazepine

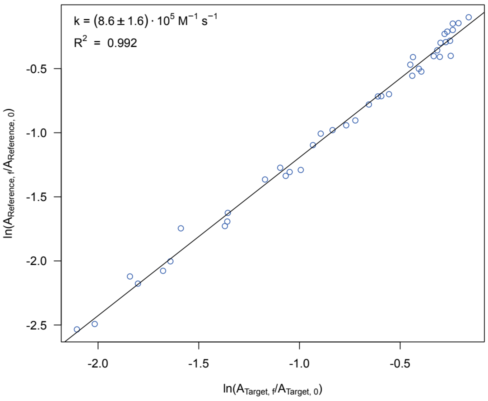

(b) Sulfamethazine - Sulfamethoxazole

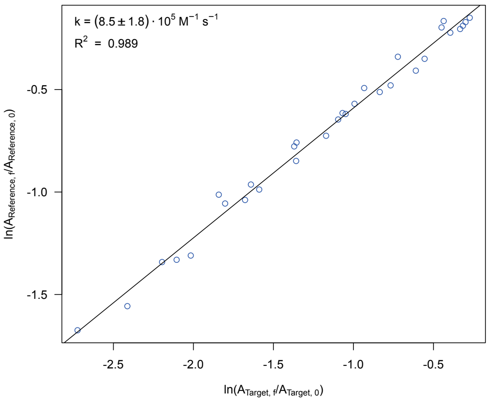

(c) Sulfamethazine - Trimethoprim

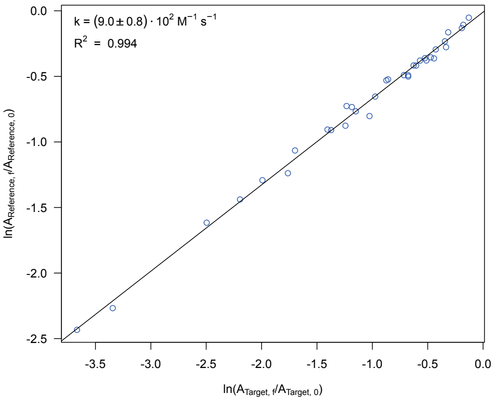

(d) N^4-Acetylsulfamethazine - Bezafibrate

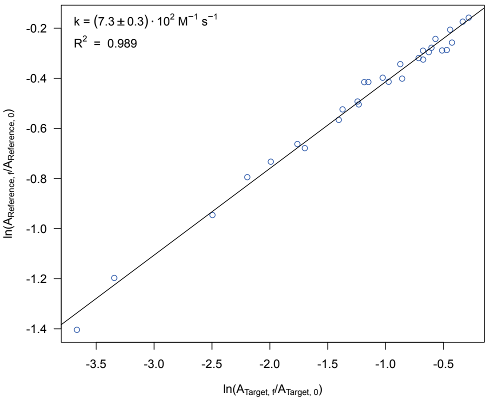

(e) N^4-Acetylsulfamethazine - N^4-Acetylsulfamethoxazole

Figure SI-B73: Correlations between the natural logarithm of the relative residual peak areas of sulfamethazine (a - c) and N^4-acetylsulfamethazine (d - e) with competitors upon ozonation at pH 7 (2 mM phosphate), 22 °C, and in presence of *t*BuOH (40 mM). The intercept was considered negligible (<10×slope) and the standard deviations of competitor $k_{\mathrm{app,O_3}}$ values were considered in the calculation of the standard deviations of the target $k_{\mathrm{app,O_3}}$ values. Note that the axes do not necessarily originate at zero.

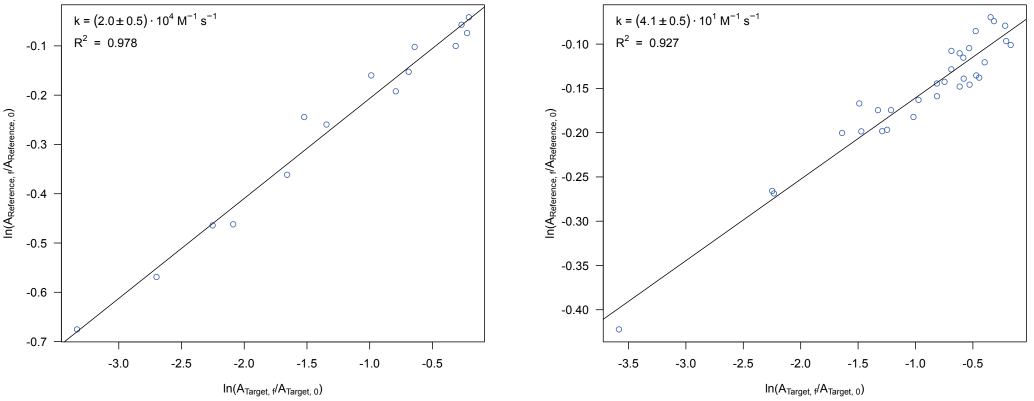

(a) Sulpiride - Tramadol

(b) Sulpiride-*N*-oxide - Alachlor

Figure SI-B74: Correlations between the natural logarithm of the relative residual peak areas of sulpiride (a) and sulpiride-*N*-oxide (b) with competitors upon ozonation at pH 7 (2 mM phosphate), 22 °C, and in presence of *t*BuOH (40 mM). The intercept was considered negligible (<10×slope) and the standard deviations of competitor $k_{\mathrm{app,O_3}}$ values were considered in the calculation of the standard deviations of the target $k_{\mathrm{app,O_3}}$ values. Note that the axes do not necessarily originate at zero.

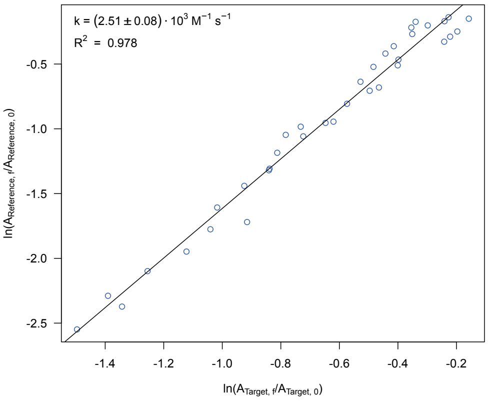

(a) Desloratadine - Penicillin G

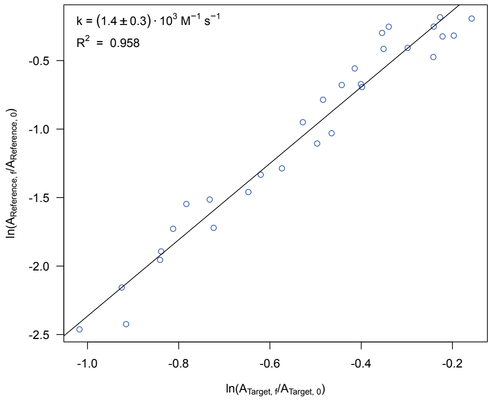

(b) Desloratadine - Tramadol

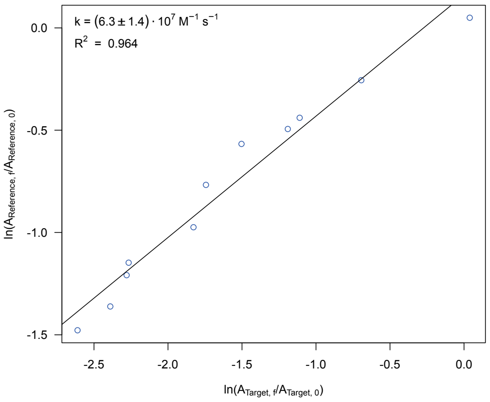

(c) 3-Hydroxydesloratadine - Triclosan

Figure SI-B75: Correlations between the natural logarithm of the relative residual peak areas of desloratadine (a - b) and 3-hydroxydesloratadine (c) with competitors upon ozonation at pH 7 (2 mM phosphate), 22 °C, and in presence of *t*BuOH (40 mM). The intercept was considered negligible (<10×slope) and the standard deviations of competitor k_{app,O_3} values were considered in the calculation of the standard deviations of the target k_{app,O_3} values. Note that the axes do not necessarily originate at zero.

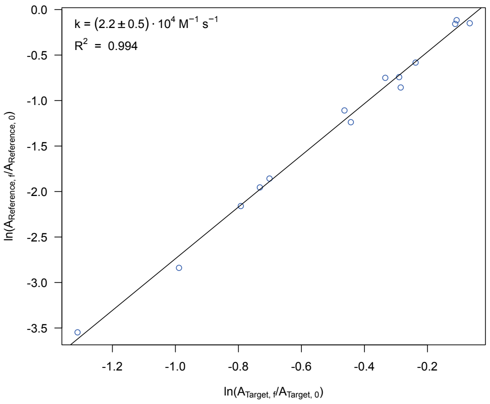

(a) Aliskiren - Roxithromycin

Figure SI-B76: Correlations between the natural logarithm of the relative residual peak areas of aliskiren with competitor roxithromycin upon ozonation at pH 7 (2 mM phosphate), 22 °C, and in presence of *t*BuOH (40 mM). The intercept was considered negligible (<10×slope) and the standard deviations of competitor $k_{\mathrm{app,O_3}}$ values were considered in the calculation of the standard deviations of the target $k_{\mathrm{app,O_3}}$ values. Note that the axes do not necessarily originate at zero.

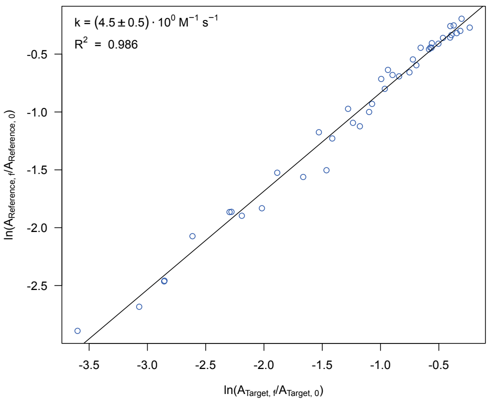

(a) Didanosine - Alachlor

Figure SI-B77: Correlations between the natural logarithm of the relative residual peak areas of didanosine with competitor alachlor upon ozonation at pH 7 (2 mM phosphate), 22 °C, and in presence of *t*BuOH (40 mM). The intercept was considered negligible (<10×slope) and the standard deviations of competitor $k_{\mathrm{app,O_3}}$ values were considered in the calculation of the standard deviations of the target $k_{\mathrm{app,O_3}}$ values. Note that the axes do not necessarily originate at zero.

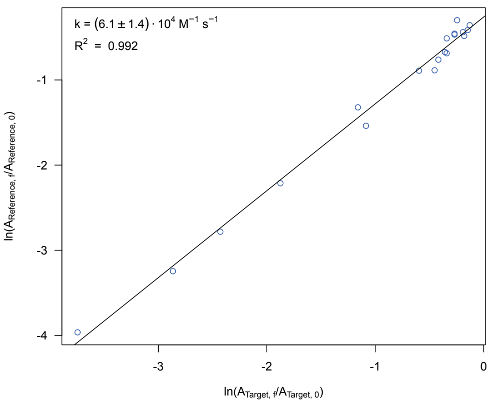

(a) Lamivudine - Roxithromycin

Figure SI-B78: Correlations between the natural logarithm of the relative residual peak areas of lamivudine with competitor roxithromycin upon ozonation at pH 7 (2 mM phosphate), 22 °C, and in presence of *t*BuOH (40 mM). The intercept was considered negligible (<10×slope) and the standard deviations of competitor $k_{\mathrm{app,O_3}}$ values were considered in the calculation of the standard deviations of the target $k_{\mathrm{app,O_3}}$ values. Note that the axes do not necessarily originate at zero.

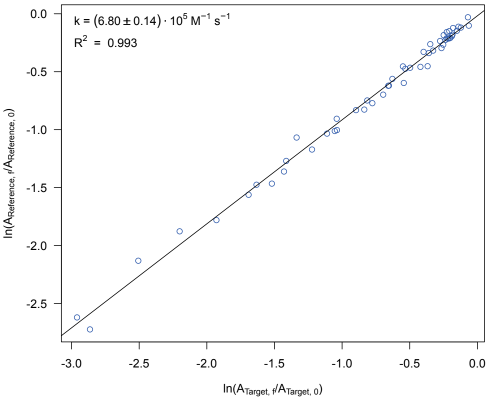

(a) *O*-Desarylranolazine - Carbamazepine

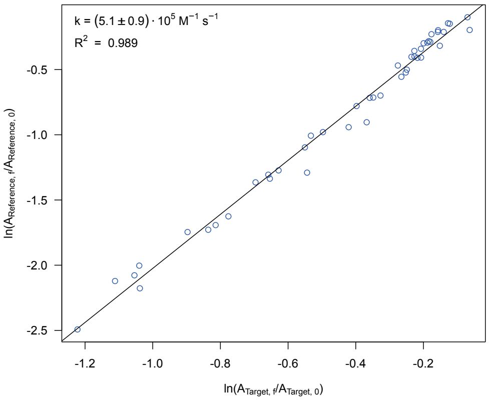

(b) *O*-Desarylranolazine - Sulfamethoxazole

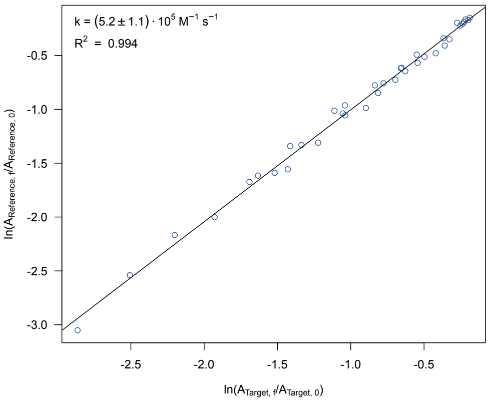

(c) *O*-Desarylranolazine - Trimethoprim

Figure SI-B79: Correlations between the natural logarithm of the relative residual peak areas of *O*-desarylranolazine with competitors upon ozonation at pH 7 (2 mM phosphate), 22 °C, and in presence of *t*BuOH (40 mM). The intercept was considered negligible (<10×slope) and the standard deviations of competitor k_{app,O_3} values were considered in the calculation of the standard deviations of the target k_{app,O_3} values. Note that the axes do not necessarily originate at zero.

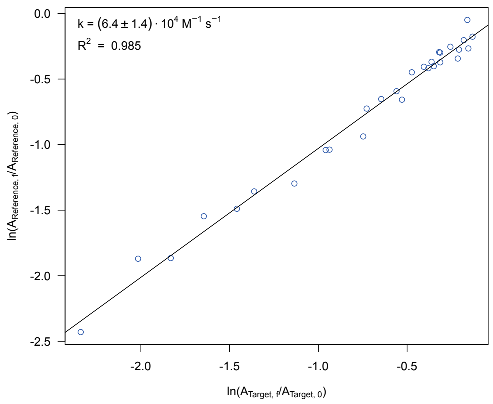

(a) Omeprazole - Roxithromycin

Figure SI-B80: Correlations between the natural logarithm of the relative residual peak areas of omeprazole with competitor roxithromycin upon ozonation at pH 7 (2 mM phosphate), 22 °C, and in presence of *t*BuOH (40 mM). The intercept was considered negligible (<10×slope) and the standard deviations of competitor $k_{\mathrm{app,O_3}}$ values were considered in the calculation of the standard deviations of the target $k_{\mathrm{app,O_3}}$ values. Note that the axes do not necessarily originate at zero.

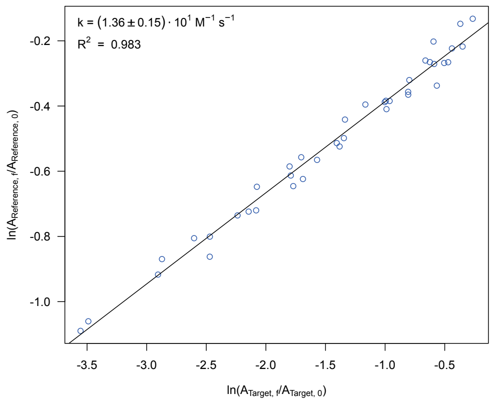

(a) Phenolic glucuronide - Alachlor

Figure SI-B81: Correlations between the natural logarithm of the relative residual peak areas of phenolic glucuronide with competitor alachlor upon ozonation at pH 7 (2 mM phosphate), 22 °C, and in presence of *t*BuOH (40 mM). The intercept was considered negligible (<10×slope) and the standard deviations of competitor $k_{\mathrm{app,O_3}}$ values were considered in the calculation of the standard deviations of the target $k_{\mathrm{app,O_3}}$ values. Note that the axes do not necessarily originate at zero.

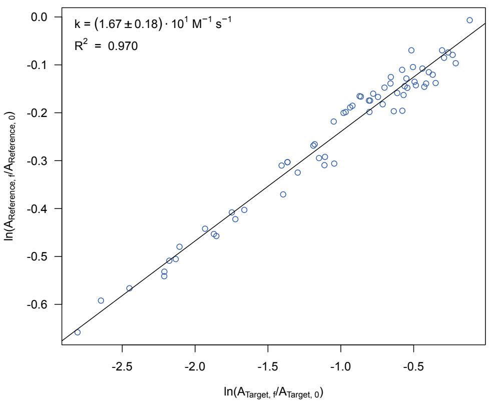

(a) Sacubitrilat - Alachlor

Figure SI-B82: Correlations between the natural logarithm of the relative residual peak areas of sacubitrilat (LBQ657) with competitor alachlor upon ozonation at pH 7 (2 mM phosphate), 22 °C, and in presence of *t*BuOH (40 mM). The intercept was considered negligible (<10×slope) and the standard deviations of competitor $k_{\mathrm{app,O_3}}$ values were considered in the calculation of the standard deviations of the target $k_{\mathrm{app,O_3}}$ values. Note that the axes do not necessarily originate at zero.

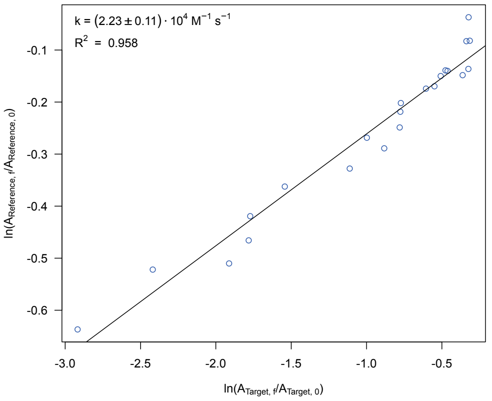

(a) Tolperisone - Penicillin G

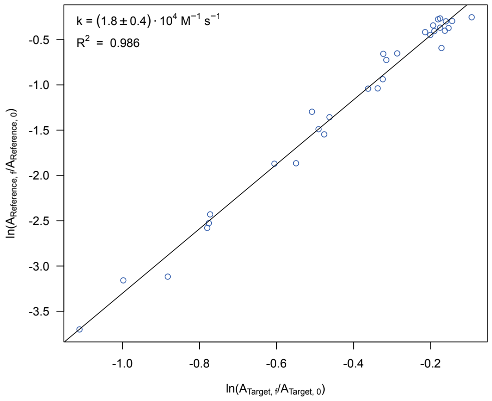

(b) Tolperisone - Roxithromycin

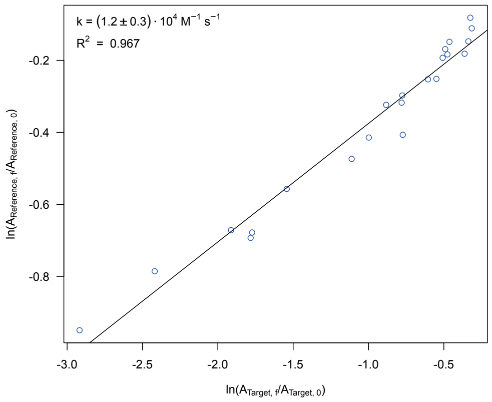

(c) Tolperisone - Tramadol

Figure SI-B83: Correlations between the natural logarithm of the relative residual peak areas of tolperisone with competitors penicillin G, roxithromycin and tramadol upon ozonation at pH 7 (2 mM phosphate), 22 °C, and in presence of *t*BuOH (40 mM). The intercept was considered negligible (<10×slope) and the standard deviations of competitor k_{app,O_3} values were considered in the calculation of the standard deviations of the target k_{app,O_3} values. Note that the axes do not necessarily originate at zero.

SI-B3 Comparing Literature and Experimental $k_{\mathrm{app,O_3}}$

Table SI-B7: Comparison of literature and experimental $k_{\mathrm{app,O_3}}$ values at pH 7. Of the 27 compounds, 14 compound have a deviation of less than a factor 2, 9 compounds of less than a factor of 5, 3 compounds are within one order of magnitude and one compound (telmisartan) deviates by more than one order of magnitude.

Compound	Structure	Deviation factor	$k_{\mathrm{app,O_3}}$ Experimental $[M^{-1}s^{-1}]$	$k_{\mathrm{app,O_3}}$ Literature $[M^{-1}s^{-1}]$	Reference
Less than factor 2 deviation					
Venlafaxine-*N*-oxide	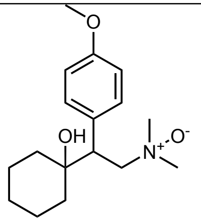	0.5	$(1.69 \pm 0.09) \times 10^2$	3.1×10^2	27
Azithromycin	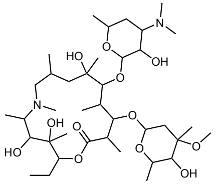	0.7	$(7.20 \pm 0.17) \times 10^4$	1.1×10^5	28
Amoxicillin	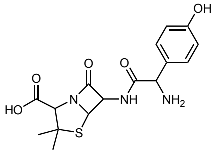	0.7	$(2.20 \pm 0.19) \times 10^6$	3×10^6	29
Paracetamol	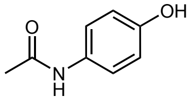	0.9	$(2.40 \pm 0.18) \times 10^6$	2.6×10^6	30
Atenolol	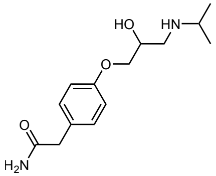	1.0	$(1.58 \pm 0.07) \times 10^3$	1.6×10^3	31

Cetirizine	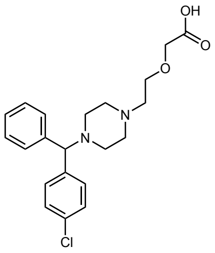	1.1	$(3.59 \pm 0.31) \times 10^5$	3.4×10^5	[32]
Diclofenac	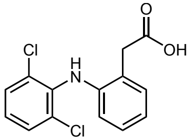	1.2	$(8.30 \pm 0.71) \times 10^5$	6.8×10^5	[33]
Venlafaxine	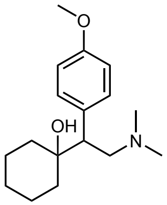	1.3	$(3.44 \pm 0.25) \times 10^3$	2.6×10^3	[27]
Ketoprofen	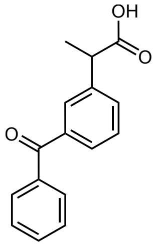	1.3	$(5.29 \pm 0.16) \times 10^{-1}$	4.0×10^{-1}	[34]
Ranitidine	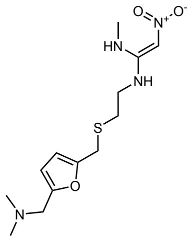	1.5	$(6.40 \pm 0.50) \times 10^6$	4.1×10^6	[35]

Eprosartan	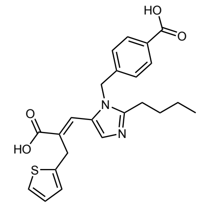	1.6	$(1.57 \pm 0.14) \times 10^6$	9.8×10^5	[36]
Clarithromycin	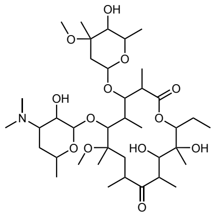	1.6	$(6.5 \pm 1.5) \times 10^4$	4.0×10^4	[37]
Propanolol	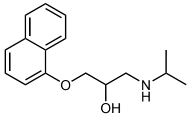	1.7	$(1.74 \pm 0.16) \times 10^5$	1.0×10^5	[31]
Fexofenadine-*N*-oxide	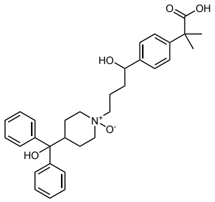	2.0	$(1.19 \pm 0.13) \times 10^1$	6.0	[32]
Naproxen	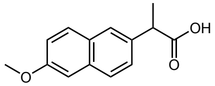	2.0	$(5.00 \pm 0.46) \times 10^5$	2.5×10^5	[38]

Less than factor 5 deviation					
Succinic acid	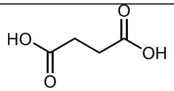	<0.3	$< k_{\min}$	<3	[39]
Oseltamivir acid	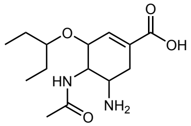	0.3	$(8.70 \pm 0.77) \times 10^4$	3.4×10^5	[40]
Fexofenadine	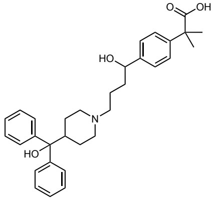	0.3	$(2.95 \pm 0.26) \times 10^3$	9.0×10^3	[32]
Hydrochlorothiazide	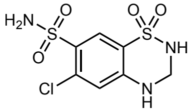	0.3	$(3.00 \pm 0.30) \times 10^3$	9.8×10^2	[32]
Losartan	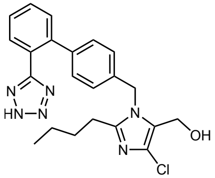	0.5	$(1.98 \pm 0.16) \times 10^5$	4.3×10^5	[36]
4-Formylaminoantipyrine	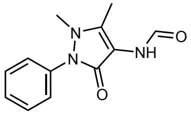	0.9	$(6.10 \pm 0.01) \times 10^4$	1.3×10^5	[41]
Iopromide	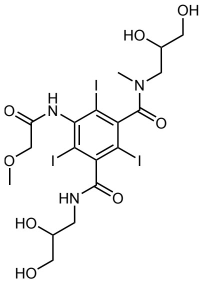	2.7	$(2.14 \pm 0.23) \times 10^0$	<0.8	[3]

Lamotrigine	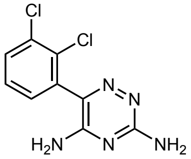	4.3	$(1.71 \pm 0.12) \times 10^1$	4	[42]
More than one order of magnitude deviation					
Telmisartan	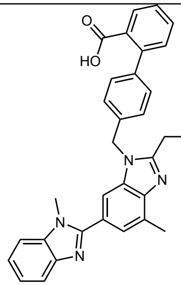	0.001	$(1.20 \pm 0.23) \times 10^2$	1.2×10^5	[36]

SI-B4 Measured vs Predicted $k_{\mathrm{app,O_3}}$: Outliers

Despite the generally good agreement between measured and literature or predicted $k_{\mathrm{app,O_3}}$ and literature values, some outliers are noticeable. All measured $k_{\mathrm{app,O_3}}$ values deviating by more than |2.5| orders of magnitude with respect to the comparative value (literature, quantitative structure-activity relationships (QSARs), derived values) will be discussed in the following and are listed with their measured and comparative $k_{\mathrm{app,O_3}}$ values in Table SI-B8.

Table SI-B8: Outliers in the comparison between determined (multi-compound competition kinetics) and predicted values for $k_{\mathrm{app,O_3}}$ with the $\mathrm{Q}k_{\mathrm{app,O_3}}$ defined as $\log_{10}(k_{\mathrm{O_3,measured}}/k_{\mathrm{O_3,estimated}})$. The compounds are ordered along increasing $\Delta k_{\mathrm{app,O_3}}$.

Compound	Determined $k_{\mathrm{app,O_3}}$ [$\mathrm{M^{-1}s^{-1}}$]	Comparative $k_{\mathrm{app,O_3}}$ [$\mathrm{M^{-1}s^{-1}}$]	Source of comparative value	$\mathbf{Q}k_{\mathrm{app,O_3}}$
4'-Hydroxydiclofenac	6.95 ± 0.38	2.67×10^8	QSAR phenol and phenolate	-7.58
Betamethasone 21-acetate	$(2.44 \pm 0.13) \times 10^1$	1.43×10^5	QSAR olefin	-3.77
Betamethasone	$(3.58 \pm 0.18) \times 10^1$	1.43×10^5	QSAR olefin	-3.60
Norketamine	$(3.75 \pm 0.25) \times 10^1$	1.38×10^5	QSAR amine	-3.56
Methylprednisolone	$(8.70 \pm 0.45) \times 10^1$	1.96×10^5	QSAR olefin	-3.35
Benzoylecgonine	$(1.13 \pm 0.12) \times 10^1$	2.33×10^4	QSAR amine	-3.31
Prednisolone	$(1.04 \pm 0.56) \times 10^2$	1.96×10^5	QSAR olefin	-3.28
Nevirapine	$(8.60 \pm 0.90) \times 10^1$	1.48×10^5	QSAR amine	-3.24
Chlorothiazide	1.57 ± 0.17	2.30×10^3	Derived from similar compound (hydrochlorothiazide)	-3.17
Telmisartan-*O*-acyl-glucuronide	$(1.11 \pm 0.05) \times 10^2$	1.20×10^5	Derived from parent	-3.03
Telmisartan	$(1.20 \pm 0.06) \times 10^2$	1.20×10^5	Literature (competition kinetics)	-3.00
Pantoprazole	$(1.09 \pm 0.06) \times 10^2$	1.00×10^5	Derived from similar compound (imidazole)	-2.96
Lidocaine-*N*-oxide	$(1.09 \pm 0.12) \times 10^1$	9.55×10^3	QSAR benzene	-2.94
D617	$(2.30 \pm 0.20) \times 10^3$	1.14×10^6	QSAR benzene	-2.70
Hydrocortisone	$(4.20 \pm 0.25) \times 10^2$	1.81×10^5	QSAR olefin	-2.63
Cortisone	$(4.36 \pm 0.22) \times 10^2$	1.81×10^5	QSAR olefin	-2.62
12-Hydroxynevirapine	$(3.92 \pm 0.21) \times 10^2$	1.48×10^5	QSAR amine	-2.58
Amlodipine	$(9.90 \pm 2.20) \times 10^7$	2.91×10^5	QSAR olefin	2.53
N^4-Acetylsulfathiazole	$(1.02 \pm 0.12) \times 10^7$	2.04×10^4	Derived from similar compound (2-methylthiazole)	2.70
3,5-Diamino-2,4,6-triiodobenzoic acid	$(1.02 \pm 0.07) \times 10^7$	1.80×10^4	QSAR aniline	2.75
N^4-Acetylsulfadiazine	$(8.90 \pm 0.49) \times 10^1$	1.23×10^{-1}	QSAR benzene	2.86
Iminostilbene	$(7.60 \pm 1.70) \times 10^7$	9.22×10^4	QSAR olefin	2.92
1,7-Dimethyluric acid	$(6.60 \pm 1.40) \times 10^7$	3.00×10^4	Derived from parent compound (caffeine)	3.34
Sulpiride-*N*-oxide	$(4.10 \pm 0.50) \times 10^1$	1.21×10^{-2}	QSAR benzene	3.53
N^4-Acetylsulfapyridine	$(9.47 \pm 2.79) \times 10^2$	1.23×10^{-1}	QSAR benzene	3.89

SI-B4.1 4'-Hydroxydiclofenac

As already discussed in the main text, 4'-hydroxydiclofenac exhibits a much lower measured rate constant than predicted by the phenol and phenolate QSAR ($k_{\mathrm{app,O_3}} = 7.0 \pm 0.4\,\mathrm{M^{-1}s^{-1}}$ vs $k_{\mathrm{pred,O_3}} = 2.7 \times 10^8\,\mathrm{M^{-1}s^{-1}}$). Although the electron-withdrawing effect of the two chlorine atoms at the phenol/phenolate reduce the reactivity compared to the unsubstituted phenol ($k_{\mathrm{app,O_3}} = 1.4 \times 10^6\,\mathrm{M^{-1}s^{-1}}$ [39]), this effect is not able to account for the observed difference of six orders of magnitude. The low $k_{\mathrm{app,O_3}}$ of 4'-hydroxydiclofenac may be explained by a secondary reaction of its oxidation product. Reaction with ozone is expected to proceed via a two-electron oxidation to form the dichloro-benzoquinoneimine. Similar, 2,6-dihaloquinones are known to be unstable and can undergo reductive back-reactions,[43] either spontaneously or via interaction with other components in the solution, regenerating 4'-hydroxydiclofenac. This hypothesis is supported when comparing with the other hydroxylated metabolite of diclofenac. 5-Hydroxydiclofenac, which has a much higher $k_{\mathrm{app,O_3}}$ value of $(6.4 \pm 1.4) \times 10^7\,\mathrm{M^{-1}s^{-1}}$, lacks electron-withdrawing substituents, and its corresponding benzoquinoneimine is expected to be more stable and less prone to such back-reactions. This difference in stability may explain the selectively lower apparent reactivity of 4'-hydroxydiclofenac in the competition kinetics experiments.

Figure SI-B84 illustrates the abatement of 4- and 5-hydroxydiclofenac with increasing ozone dose in the range of 2 to 1560 mol of ozone per mol of 4-hydroxydiclofenac, and of 0.02 to 690 mol ozone per mol of 5-hydroxydiclofenac, together with the peak areas of the corresponding benzoquinoneimines. Note that the ozone doses (mol O_3 per mol compound) cannot be taken as absolute values, since other compounds and competitors are present in solution which contribute to ozone consumption. Both hydroxylated diclofenac derivatives form redox couples with their benzoquinoneimine counterparts, allowing interconversion through two-electron oxidation and reduction reactions.[44] For the 5-hydroxydiclofenac/benzoquinoneimine couple, there is an equilibrium for the two species prior to ozone addition (Figure SI-B84, left panel). With increasing ozone dose, 5-hydroxydiclofenac is oxidized to the benzoquinoneimine, resulting in a decline of the peak area of 5-hydroxydiclofenac, followed by a second equilibrium between 5-hydroxydiclofenac and the benzoquinoneimine. At higher doses, the benzoquinoneimine itself undergoes ozonation, leading in turn to a second decrease in 5-hydroxydiclofenac. The benzoquinoneimine reacts with a similar second-order rate constant with ozone as the competitor bezafibrate ($5.9 \times 10^2\,\mathrm{M^{-1}s^{-1}}$ [3]) consistent with the reactivity of deactivated olefins.[45] The equilibrium between 5-hydroxydiclofenac and the benzoquinoneimine counterpart is driven by the formation of the benzoquinoneimine from 5-hydroxydiclofenac by the reaction with ozone, and the reduction of the benzoquinoneimine to 5-hydroxydiclofenac by a reducing agent present in solution. Other potentially reducing compounds in the same mixture for $k_{\mathrm{app,O_3}}$ determination exhibit apparent second-order rate constants for the reaction with ozone $>10^6\,\mathrm{M^{-1}s^{-1}}$. Hence they are already in an oxidized state in the second equilibrium and cannot act as reducing agents to achieve the second equilibrium observed. Due to presence of *t*BuOH as hydroxyl radical quencher, notable concentrations of hydrogen peroxide are present in solution, as the yield of H_2O_2 from the reaction of *t*BuOH with hydroxyl radicals corresponds to around 30%.[46] It is hypothesized that this hydrogen peroxide, together with copper impurities present in solution, leads to Fenton-type reactions and the formation of superoxide,[47] which can act as reducing agent for the reduction of the benzoquinoneimine. Besides the potential Fenton-type reaction, molecular oxygen might play an important role for the establishment of the equilibrium between the phenol and the benzoquinoneimine species.[48] Additional experimental studies would be necessary to elucidate this further.

In contrast, the equilibrium between benzoquinoneimine and 4-hydroxydiclofenac is strongly on the phenol side, likely due to the electron-withdrawing effect of the two chlorine substituents (Figure SI-B84, right panel). As a result, even for increasing ozone doses, the benzoquinoneimine formed upon reaction of 4-hydroxydiclofenac with ozone is quantitatively reduced back to 4-hydroxydiclofenac. Nevertheless, the abatement of 4-hydroxydiclofenac is controlled by benzoquinoneimine. This leads to a decrease of 4-hydroxydiclofenac for similar molar ozone:target compound ratios as for 5-hydroxydiclofenac.

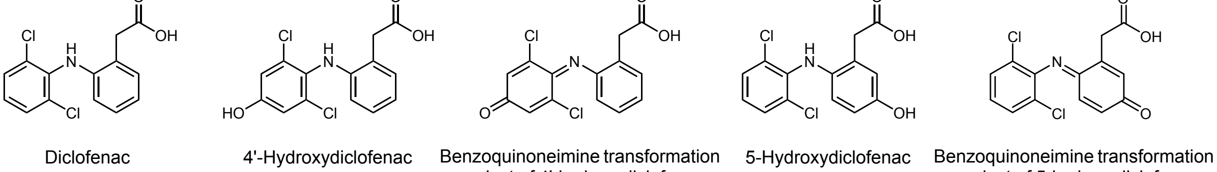

Diclofenac | 4'-Hydroxydiclofenac | Benzoquinoneimine transformation product of 4'-hydroxydiclofenac | 5-Hydroxydiclofenac | Benzoquinoneimine transformation product of 5-hydroxydiclofenac

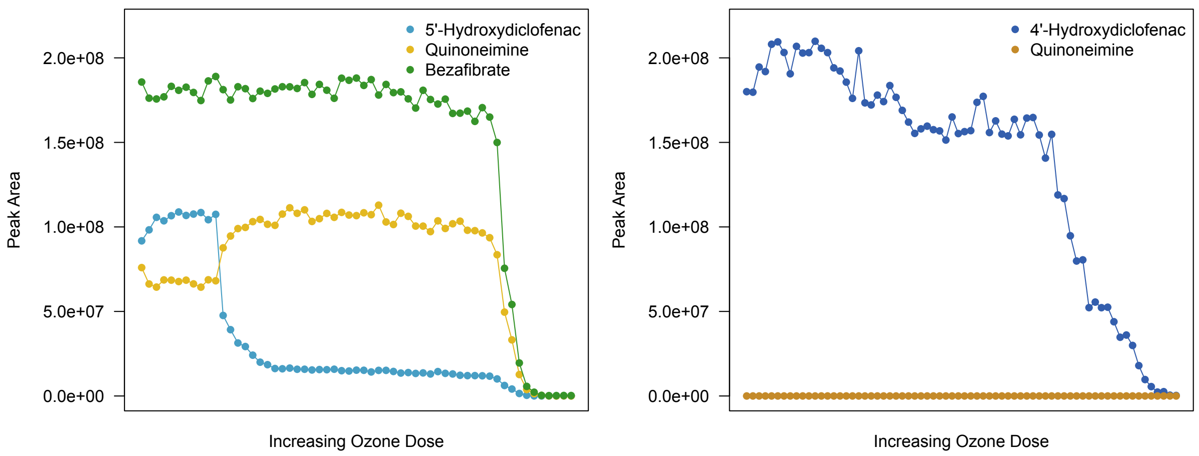

Figure SI-B84: Evolution of peak areas of 5-hydroxydiclofenac, the benzoquinoneimine counterpart, and the competitor bezafibrate (left), and of 4-hydroxydiclofenac and its benzoquinoneimine counterpart (right) with increasing applied specific ozone doses in ultrapure water at pH 7, and with excess of tBuOH to scavenge hydroxyl radicals. Specific ozone doses ranged from 0.02 to 690 mol ozone per mol of 5-hydroxydiclofenac (left), and from 2 to 1560 mol ozone per mol of 4-hydroxydiclofenac (right). Note that these specific ozone doses cannot be taken as absolute values, since other compounds and competitors are present in solution which contribute to ozone consumption.

SI-B4.2 Cortisone-like structures

For compounds with cortisone-like structures (betamethasone, betamethasone-21-acetate, cortisone, hydrocortisone, methylprednisolone, prednisolone and prednisone), second-order rate constants were predicted with the olefin QSAR, since the olefin is the most reactive site towards ozone. However, the predicted values ($k_{\mathrm{app,O_3}}$ = $1.4 \times 10^5\,\mathrm{M^{-1}s^{-1}}$ to $2.0 \times 10^5\,\mathrm{M^{-1}s^{-1}}$) are two to three orders of magnitude higher than the measured rate constants ($k_{\mathrm{app,O_3}} = 24\,\mathrm{M^{-1}s^{-1}}$ to $436\,\mathrm{M^{-1}s^{-1}}$). A structurally similar compound, the hormone progesterone, has a $k_{\mathrm{app,O_3}}$ value of $480\,\mathrm{M^{-1}s^{-1}}$,[49] which is more comparable to the measured rate constants. Hence, the olefin QSAR seems to be not applicable for rate constant prediction of compounds with cortisone-like structures.

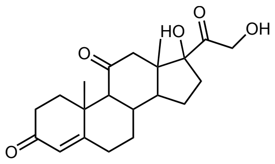

Cortisone

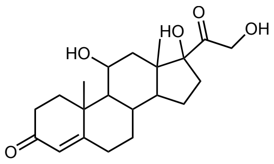

Hydrocortisone

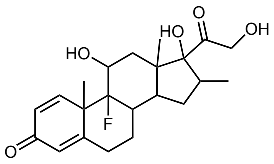

Betamethasone

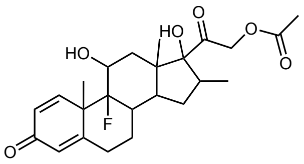

Betamethasone 21-acetate

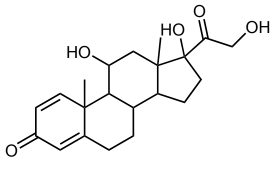

Prednisolone

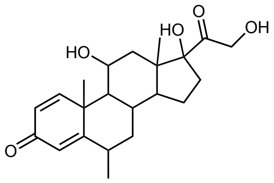

Methylprednisolone

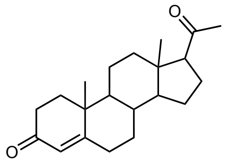

Progesterone, $k_{O3} = 480\ \mathrm{M^{-1}s^{-1}}$

SI-B4.3 Nevirapine and 12-hydroxynevirapine

For nevirapine and its human metabolite 12-hydroxynevirapine, the tertiary amine was expected to be the most reactive site, although deactivated by the two pyridine rings. Correspondingly, $k_{\mathrm{app,O_3}}$ was predicted by the amine QSAR, resulting in a value of $1.48 \times 10^5\,\mathrm{M^{-1}s^{-1}}$ for the neutral species of both compounds. Due to the low pK_a value of 2.8[50] for nevirapine, the tertiary amines are present in their neutral form at pH 7. However, the measured $k_{\mathrm{app,O_3}}$ values amount only to $(8.60 \pm 0.90) \times 10^1\,\mathrm{M^{-1}s^{-1}}$ and $(3.92 \pm 0.21) \times 10^2\,\mathrm{M^{-1}s^{-1}}$ for nevirapine and 12-hydroxynevirapine, respectively. The reaction of the tertiary amine with ozone would lead to the formation of a N-oxide[51] and thereby result in a quarternary nitrogen atom. The incorporation of this N-oxide in the

seven-membered ring would lead to an increased ring strain. Hence, the whole reaction has a higher activation energy and a correspondingly lower reaction rate constant compared to a non-cyclic tertiary amine with similar substituents. Since the QSAR predictions do not take such steric considerations into account, the $k_{\mathrm{app,O_3}}$ value was overestimated.

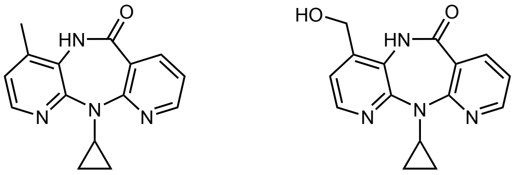

Nevirapine 12-Hydroxynevirapine

SI-B4.4 Benzoylecgonine and norketamine

The reactivity of benzoylecgonine towards ozone is expected to be driven by the tertiary amine. For the neutral amine, the QSAR prediction yielded a value of $3.24 \times 10^6\,\mathrm{M^{-1}s^{-1}}$. Considering that at pH 7 only a small fraction of the amine is present in the neutral form and that the protonated amine has no significant reactivity towards ozone, the apparent second order rate constant at pH 7 was calculated based on the speciation of benzoylecgonine and amounts to $2.33 \times 10^4\,\mathrm{M^{-1}s^{-1}}$. This pH corrected prediction is more than three orders of magnitude higher than the measured $k_{\mathrm{app,O_3}}$ value, which amounts to $(1.13 \pm 0.12)\times 10^1\,\mathrm{M^{-1}s^{-1}}$. This difference is assumed to originate from incorrect pK_a values, based on which the speciation was calculated. The pK_a of 9.14 was predicted using JChem for Excel.[52] However, this prediction does not take sterical considerations and hydrogen bonds into account. Since the protonated form of benzoylecgonine can undergo hydrogen bonds with the carboxylic acid functionality, the pK_a might be much higher than estimated, which in turn leads to a higher fraction of protonated amine present at pH 7. Consequently, the $k_{\mathrm{app,O_3}}$ value is lower and estimated to be in the same range than the value measured in this study. One measured pK_a value of benzoylecgonine from the year 1925 is available in literature, which amounts to 11.8.[53] Considering the difference between the two pK_a values of 2.66 and adjusting the speciation, a corrected predicted $k_{\mathrm{app,O_3}}$ value of $5.21 \times 10^1\,\mathrm{M^{-1}s^{-1}}$ results, which lies within one order of magnitude compared to the experimentally determined rate constant.

A similar reasoning as for benzoylecgonine is assumed to hold true for norketamine with a predicted pK_a value of 7.02.[52] Due to the electron-withdrawing effect of the keto-carbonyl group in β-position, the lower pK_a compared to other primary amines is reasonable.[54] However, the electron-withdrawing effect is offset due to hydrogen bonds with the carbonyl group of the cyclohexanone, as well as with the chlorine atom bound to the benzene ring. The unprotonated norketamine was assigned a predicted $k_{\mathrm{app,O_3}}$ of $2.82 \times 10^5\,\mathrm{M^{-1}s^{-1}}$ based on the amine QSAR. Considering speciation, the apparent second-order rate constant at pH 7 was calculated to be $1.38 \times 10^5\,\mathrm{M^{-1}s^{-1}}$, which is more than three orders of magnitude higher than what was determined in this study ($3.75 \pm 0.25\,\mathrm{M^{-1}s^{-1}}$). However, since no reference pK_a values are available in literature, the given hypothesis cannot be justified.

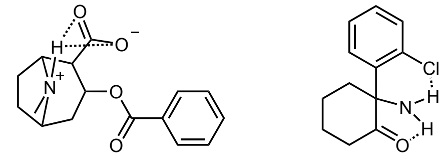

Benzoylecgonine Norketamine

Similar effects were thought to also be important for other compounds where the amine is responsible for the reaction with ozone. However, benzoylecgonine and norketamine are the only compounds for which this effect led to a deviation of more than 2.5 orders of magnitude compared to the predicted values. Other exemplary compounds with a carbonyl group in beta position to an amine and thus in the reach for hydrogen bonds are bupropion and lidocaine. The literature pK_a value of bupropion is 8.2,[55] while the predicted value equals 8.22.[52] For lidocaine, the literature pK_a values equals 7.86,[56] while the predicted value corresponds to 7.75.[52] Thus, pK_a predictions seem accurate with JChem for Excel,[52] as long as the special geometry as for benzoylecgonine, or additional H-bond acceptors, as chlorine in norketamine, do not lead to hydrogen bonds.

SI-B4.5 D617 and lidocaine-*N*-oxide

The reactivity towards ozone of D617 (2-(3,4-dimethoxyphenyl)-2-isopropyl-5-(methylamino)-pentanenitrile), a human metabolite of verapamil, was predicted with the amine and the benzene QSAR. Due to the electron-donating effect of the two methoxy groups and the quarternary carbon atom on the benzene ring, leading to its activation, a high $k_{\mathrm{O_3}}$ value of $1.14 \times 10^6\,\mathrm{M^{-1}s^{-1}}$ was predicted, exceeding the value predicted by the amine QSAR

and the experimental value determined in this study ($(2.30 \pm 0.20) \times 10^3\ M^{-1}s^{-1}$). The reactivity of a structurally similar compound to the benzene moiety present in D617, 1,3,5-trimethoxybenzene, was overestimated by two orders of magnitude by the benzene QSAR of *et al.*[57] For the development of the QSAR models, mostly mono-substituted compounds were included, due to the limited availability of species-specific k_{app,O_3} values.[57] Although these mono-substituted compounds allow a better mechanistic understanding, they are only partly able to capture more complex substitution patterns, as for example for D617. Since all three substituents are electron-donating, it might be that the sum of all electron-donating effects does not resemble reality. If the donating effect of one of the methoxy groups is neglected, a k_{app,O_3} value almost three orders of magnitude lower ($2.78 \times 10^3\ M^{-1}s^{-1}$) results, which is close to the experimental value.

The same reasoning is expected to be responsible for the overestimated k_{app,O_3} value of lidocaine-*N*-oxide, which was with the benzene QSAR predicted to be $9.55 \times 10^3\ M^{-1}s^{-1}$. In contrast, the measured value amounts to $(1.09 \pm 0.12) \times 10^1\ M^{-1}s^{-1}$. Again, with two methyl groups and one amide group, three electron-donating substituents are bound to the benzene ring.

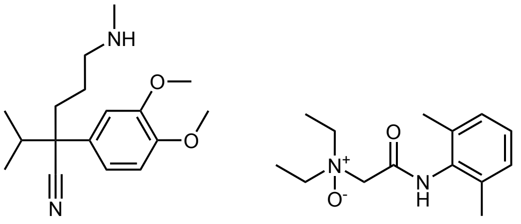

D617 Lidocaine-N-oxide

SI-B4.6 *N*⁴-Acetylsulfadiazine, *N*⁴-acetylsulfapyridine and *N*⁴-acetylsulfathiazole

In contrast to the parent sulfadiazine, for which the reactivity was predicted with the aniline QSAR, the k_{app,O_3} value of the metabolite N^4-acetylsulfadiazine was predicted with the benzene QSAR. The resulting k_{app,O_3} value was underestimated by almost three orders of magnitude ($1.23 \times 10^{-1}\ M^{-1}s^{-1}$) compared to the experimentally determined second-order rate constant ($(8.90 \pm 0.49) \times 10^1\ M^{-1}s^{-1}$). The partial deprotonation of the -SO_2NH_2 group at pH 7 with a pK_a of 6.9[52] and the resulting increased electron density on the benzene ring was not captured with the QSAR, since no Hammett constant for the -SO_2NH_2 group was available. Therefore, the Hammett constant of -$SO_2N(CH_3)_2$ was taken, which cannot be deprotonated. Since the pyrimidine itself has a low k_{app,O_3} value, it is not assumed that this ring contributes to the reactivity.

The same explanation is expected to hold true for N^4-acetylsulfapyridine, whose k_{app,O_3} value was predicted to be $1.23 \times 10^{-1}\ M^{-1}s^{-1}$ based on the benzene QSAR. The measured value is nearly four orders of magnitude larger and amounts to $(9.47 \pm 2.79) \times 10^2\ M^{-1}s^{-1}$. With a p$K_a$ of 6.9,[52] the sulfonamide is also partially deprotonated at pH 7, which was not captured with the Hammett constant of the -$SO_2N(CH_3)_2$ group in the QSAR prediction. Similar to N^4-acetylsulfadiazine with the pyrimidine ring, the pyridine ring in N^4-acetylsulfapyridine is not expected to contribute to the reactivity towards ozone, making the sulfonamide the reactive site.

For sulfathiazole, the second-order rate constant was predicted based on the aniline QSAR, while the value for its metabolite, N^4-acetylsulfathiazole, was predicted from a structurally similar compound (2-methylthiazole, $k_{app,O_3} = 2.04 \times 10^4\ M^{-1}s^{-1}$[58]). However, the experimentally determined k_{app,O_3} value for N^4-acetylsulfathiazole is almost three orders of magnitude larger and equals to $(1.02 \pm 0.12) \times 10^7\ M^{-1}s^{-1}$. The direct transfer of the rate constant from 2-methylthiazole seems to not correctly cover the reactivity of N^4-acetylsulfathiazole. This is due to the effect of the sulfonamide. Compared to the other two discussed metabolites N^4-acetylsulfadiazine and N^4-acetylsulfapyridine with pK_a values of 6.9 and 7.0,[52] respectively, the sulfonamide is mostly present in its deprotonated form at pH 7 (96 % as predicted by JChem for Excel, pK_a = 5.6[52]), increasing the electron density of the thiazole ring and making it more reactive towards ozone compared to the 2-methylthiazole. Moreover, it can be excluded that the reactivity of the deprotonated sulfonamide itself contributes to the overall high reactivity of N^4-acetylsulfathiazole, as evidenced by the low k_{app,O_3} value of $2.5 \times 10^2\ M^{-1}s^{-1}$ for N^4-acetylsulfamethoxazole,[6] which exhibits a very similar predicted pK_a value of 5.6.[52] As a consequence, it has to be assumed that the thiazole ring is responsible for the reactivity of N^4-acetylsulfathiazole. By comparison to its *N*-acetyl metabolite, the reactivity of the parent sulfathiazole is almost identical. From the reactivity of the aniline in sulfamethoxazole, sulfadiazine, sulfapyridine, sulfamethazine and sulfadimethoxine, the rate constant of the aniline in sulfathiazole is expected to be similar, around $10^6\ M^{-1}s^{-1}$ at pH 7,[1,6] which is around an order of magnitude lower than the k_{app,O_3} of sulfathiazole. We therefore hypothesize that the reactivity for sulfathiazole and N^4-acetylsulfathiazole is driven by the thiazole ring. In contrast, for the other five sulfonamide antibiotics, the reactivity is driven by the aniline, while it is the benzene ring for N^4-acetylsulfamethoxazole, N^4-acetylsulfadiazine, N^4-acetylsulfapyridine

and N^4-acetylsulfamethazine, while for N^4-acetsulfadimethoxine and N^4-acetylsulfathiazole, the reactivity is governed by the heterocycle.

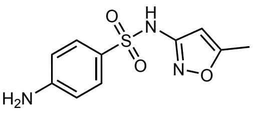

Sulfamethoxazole
$k_{app,O3}$ = (1.1 ± 0.2) x 10^6 $M^{-1}s^{-1}$
pK_a = 5.6

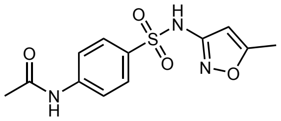

N^4-Acetylsulfamethoxazole
$k_{app,O3}$ = (2.5 ± 0.1) x 10^2 $M^{-1}s^{-1}$
pK_a = 5.6 (predicted)

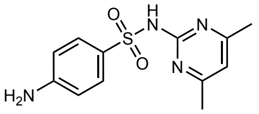

Sulfamethazine
$k_{app,O3}$ = (9.3 ± 0.8) x 10^5 $M^{-1}s^{-1}$
pK_a = 7.6

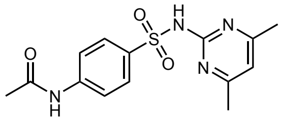

N^4-Acetylsulfamethazine
$k_{app,O3}$ = (8.2 ± 0.4) x 10^2 $M^{-1}s^{-1}$
pK_a = 6.9 (predicted)

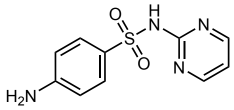

Sulfadiazine
$k_{app,O3}$ = (1.2 ± 0.1) x 10^6 $M^{-1}s^{-1}$
pK_a = 6.4

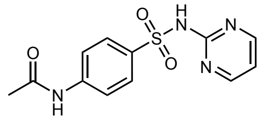

N^4-Acetylsulfadiazine
$k_{app,O3}$ = (8.9 ± 0.5) x 10^1 $M^{-1}s^{-1}$
pK_a = 6.9 (predicted)

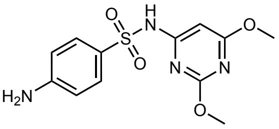

Sulfadimethoxine
$k_{app,O3}$ = (1.5 ± 0.1) x 10^6 $M^{-1}s^{-1}$
pK_a = 6.9 (predicted)

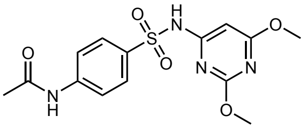

N^4-Acetylsulfadimethoxine
$k_{app,O3}$ = (3.4 ± 0.3) x 10^5 $M^{-1}s^{-1}$
pK_a = 6.3 (predicted)

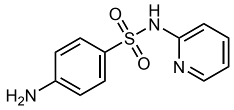

Sulfapyridine
$k_{app,O3}$ = (5.4 ± 1.8) x 10^5 $M^{-1}s^{-1}$
pK_a = 8.4

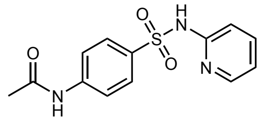

N^4-Acetylsulfapyridine
$k_{app,O3}$ = (9.5 ± 2.8) x 10^2 $M^{-1}s^{-1}$
pK_a = 7.0 (predicted)

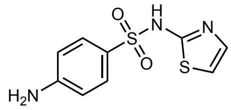

Sulfathiazole
$k_{app,O3}$ = (9.8 ± 0.7) x 10^6 $M^{-1}s^{-1}$
pK_a = 7.2

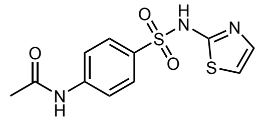

N^4-Acetylsulfathiazole
$k_{app,O3}$ = (1.0 ± 0.1) x 10^7 $M^{-1}s^{-1}$
pK_a = 5.6 (predicted)

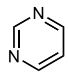

Pyrimidine
$k_{app,O3}$ = 0.07 $M^{-1}s^{-1}$

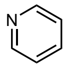

Pyridine
$k_{app,O3}$ < 3 $M^{-1}s^{-1}$

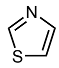

Thiazole
$k_{app,O3}$ = 1.5 x 10^2 $M^{-1}s^{-1}$

SI-B4.7 Telmisartan, telmisartan-*O*-acyl-glucuronide and pantoprazole

The reactivity of telmisartan towards ozone was three orders of magnitude lower when compared to the literature value ($k_{app,O_3} = 1.2 \times 10^5\,M^{-1}s^{-1}$), which was determined by competition kinetics, using *p*-cresol as reference compound.[36] *p*-Cresol has a second-order rate constant of $7 \times 10^5\,M^{-1}s^{-1}$ for the neutral species.[59] At pH 7 however, with a pK_a of 10.26,[60] 0.05 % of *p*-cresol is present in its deprotonated form, leading to a higher k_{app,O_3} value of $1.6 \times 10^6\,M^{-1}s^{-1}$ based on phenolate QSAR predictions.[57] This pH-dependence introduces an error into the k_{app,O_3} value of telmisartan. However, the k_{app,O_3} value for telmisartan determined with the competitors bezafibrate, carbofuran and N^4-acetylsulfamethoxazole in this study appears plausible, given the expected reactivity of its benzimidazole moieties ($2.2\,M^{-1}s^{-1}$[61]) and the alkyl substitution. This effect of increasing k_{O_3} with increasing degree of alkyl substitution is also observed when moving from benzene ($k_{O_3} = 2\,M^{-1}s^{-1}$[59]) to toluene ($k_{O_3} = 14\,M^{-1}s^{-1}$[59]) and further to xylene ($k_{O_3} = 90\,M^{-1}s^{-1}$ to $140\,M^{-1}s^{-1}$[39]). A similarly low second-order rate constant ($k_{app,O_3} = 111 \pm 5\,M^{-1}s^{-1}$) was determined in another mixture in this study for telmisartan-*O*-acyl-glucuronide, where the alkyl substituted benzimidazole moieties are also likely responsible for ozone reactivity. Hence, the low k_{app,O_3} values are not assumed to be a consequence of interactions with other compounds in the same mixture, one of the concerns when determining second-order rate constants in compound mixtures.

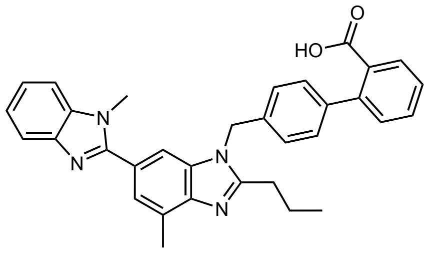

Telmisartan

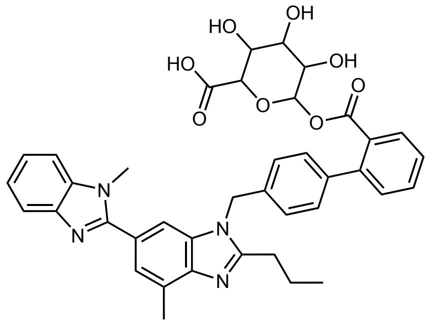

Telmisartan-O-acyl-glucuronide

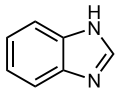

Benzimidazole

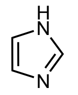

Imidazole

The $k_{\mathrm{app,O_3}}$ value of pantoprazole was directly predicted from imidazole and lowered by roughly a factor two to account for the electron-delocalizing of the attached benzene ring and the deactivation by the sulfoxide and the -OCF_2H group, leading to a value of $1.0 \times 10^5\,\mathrm{M^{-1}s^{-1}}$. However, the experimentally determined second-order rate constant equals to $(1.09 \pm 0.06)\times10^2\,\mathrm{M^{-1}s^{-1}}$ and is similarly to telmisartan and telmisartan-*O*-acyl-glucuronide three orders of magnitude lower than initially predicted. Considering the in the meantime determined second-order rate constant of benzimidazole ($2.2\,\mathrm{M^{-1}s^{-1}}$ [61]), the experimentally determined $k_{\mathrm{app,O_3}}$ of pantoprazole remains too high, since due to the electron-withdrawing effect of the substituents a lower rate constant than for benzimidazole is expected. It is therefore hypothesized that the pyridine is responsible for the reactivity, since it is activated by two methoxy groups.

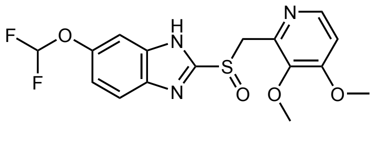

Pantoprazole

SI-B4.8 3,5-Diamino-2,4,6-triiodobenzoic acid

The $k_{\mathrm{app,O_3}}$ value of 3,5-diamino-2,4,6-triiodobenzoic acid, a human metabolite of diatrizoate, was predicted with the aniline QSAR and amounts to $1.80 \times 10^4\,\mathrm{M^{-1}s^{-1}}$, while the experimentally determined value is almost three orders of magnitude higher ($(1.02 \pm 0.07)\times10^7\,\mathrm{M^{-1}s^{-1}}$). A lower $k_{\mathrm{app,O_3}}$ value compared to aniline ($3.8\times10^7\,\mathrm{M^{-1}s^{-1}}$ [62]) initially seemed reasonable, due to the electron-withdrawing effect of the carboxylic acid and the iodine substituents. However, this assumption may overestimate the electron-withdrawing effect of iodine, which, despite its electronegativity, is also highly polarizable and can participate in weak resonance donation, potentially moderating its deactivating effect. Moreover, 3,5-diamino-2,4,6-triiodobenzoic acid is a heavily substituted molecule, and such structural complexity may challenge the reliability of additive QSAR approaches. In addition, 3,5-diamino-2,4,6-triiodobenzoic acid is composed of two amine groups bound to a benzene ring (*meta*-phenylenediamine). This offers more reaction pathways, potentially explaining the higher observed second-order rate constant. For *para*-phenylenediamine, a published experimental $k_{\mathrm{app,O_3}}$ value is available, which amounts to $(1.30 \pm 0.72)\times10^6\,\mathrm{M^{-1}s^{-1}}$. [62] However, due to the different substitution pattern, the transferability to *meta*-phenylenediamine is limited and it remains unclear if the second amine group is able to explain the higher observed reactivity of 3,5-diamino-2,4,6-triiodobenzoic acid than initially anticipated.

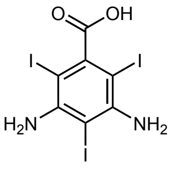

3,5-Diamino-2,4,6-triiodobenzoic acid

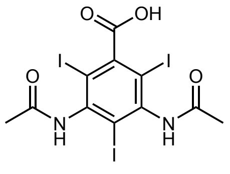

Diatrizoate

SI-B4.9 Iminostilbene

The reactivity towards ozone for iminostilbene was predicted with the olefin QSAR due to the structural similarity with carbamazepine, where the olefin is the main site for reaction with ozone. This QSAR prediction resulted in a value of $9.22 \times 10^4\,\mathrm{M^{-1}s^{-1}}$. The experimentally determined $k_{\mathrm{app,O_3}}$ value is higher and amounts to $(7.6 \pm 1.7)\times10^7\,\mathrm{M^{-1}s^{-1}}$. Since no Taft constant was available for a phenyl group substituted with an amine in the *ortho*-position, the QSAR prediction instead used the Taft constant for an unsubstituted phenyl group, which may have underestimated the electron-donating effect and thus the reactivity of the olefin. Alternatively, rather

the diphenylamine instead of the olefin might be responsible for the reaction with ozone. However, the predicted k_{app,O_3} is with $1.38\times10^4\ M^{-1}s^{-1}$ even lower. Moreover, the partial (due to not complete planarity of iminostilbene) conjugation with the double bond to the other aromatic ring could not be considered, potentially explaining the observed deviation between experimental and predicted value. Diclofenac features a similar diphenylamine structure, with a rate constant of $6.8 \times 10^5\ M^{-1}s^{-1}$,[33] which is still two orders of magnitude lower than the experimentally determined rate constant of iminostilbene, but is also lacking the connection of the two aromatic rings by the olefin.

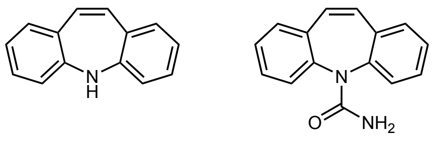

Iminostilbene Carbamazepine

SI-B4.10 Sulpiride-*N*-oxide

The k_{app,O_3} value at pH 7 for sulpiride was determined using the amine QSAR. Since this amine is not available for a reaction with ozone in sulpiride-*N*-oxide, its second-order rate constant was predicted with the benzene QSAR ($1.21 \times 10^{-2}\ M^{-1}s^{-1}$), since the benzene ring was assumed to be responsible for the reaction with ozone. For sulpiride-*N*-oxide, the predicted rate constant is three orders of magnitude lower than the rate constant determined experimentally ($(4.10 \pm 0.50)\times10^1\ M^{-1}s^{-1}$). Since for the Hammett constant σ^+_{para} of -SO_2NH_2 no value was available and was therefore replaced by the constant of -$SO_2N(CH_3)_2$, the effect of this substituent on the benzene ring of sulpiride-*N*-oxide is not be properly explained. The partial deprotonation of the -SO_2NH_2 group at pH 7 (pK_a = 10.23) compared to the -$SO_2N(CH_3)_2$ group leads to an increased electron density for the benzene ring. A similar activating but much stronger effect due to the lower pK_a of 5.86 is observed for sulfamethoxazole.[6]

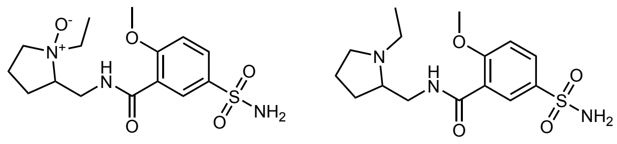

Sulpiride-N-oxide Sulpiride

SI-B4.11 Chlorothiazide

The k_{app,O_3} value of chlorothiazide was predicted from a structurally similar compound, hydrochlorothiazide, whose second-order rate constant amounts to $5.9 \times 10^2\ M^{-1}s^{-1}$ for the neutral species and to $3.0 \times 10^5\ M^{-1}s^{-1}$ for the negatively charged species.[32] To get the k_{app,O_3} at pH 7, the speciation of chlorothiazide was predicted with JChem for Excel (pK_a = 8.33),[52] resulting in a predicted value of $2.30 \times 10^3\ M^{-1}s^{-1}$. However, the experimentally determined value of chlorothiazide is three orders of magnitude lower and equals to $1.57\pm0.17\ M^{-1}s^{-1}$, which aligns with the literature value of $1.5 \pm 0.1\ M^{-1}s^{-1}$.[32] For both compounds, it is assumed that the aniline-type structure is responsible for the reaction. However, the electron-withdrawing effect of the sulfonimine in chlorothiazide is stronger than of the sulfonamide in hydrochlorothiazide, leading to a lower reactivity. Moreover, the ring sulfonamide of hydrochlorothiazide with a pK_a of 9.57 is partially deprotonated at pH 7, leading to an activation of the benzene ring and thus to a higher k_{app,O_3} value of hydrochlorothiazide compared to chlorothiazide. Moreover, chlorothiazide can undergo tautomerism, resulting in an imine-type structure and a sulfonamide, which are both not reactive with ozone. The overall reactivity likely depends on the position of the tautomeric equilibrium, as only the fraction of the molecule present in the reactive form would contribute to ozone abatement. As for hydrochlorothiazide, chlorothiazide is expected to react via electron transfer reactions.[32]

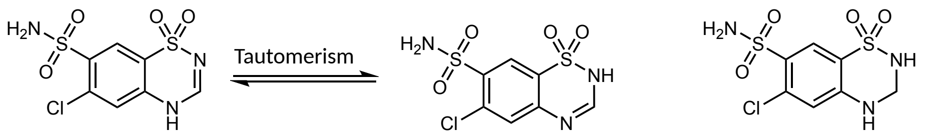

Chlorothiazide Hydrochlorothiazide

SI-B4.12 Amlodipine

For amlodipine, the two olefins were assumed to be responsible for the reaction, leading to a predicted $k_{\mathrm{app,O_3}}$ value of $2.91 \times 10^5\,\mathrm{M^{-1}s^{-1}}$. The experimentally determined rate constant is however more than two orders of magnitude larger and amounts to $(9.9 \pm 2.2){\times}10^7\,\mathrm{M^{-1}s^{-1}}$. Although with two amines other reactive functional groups are present, their reactivity is expected to be even lower than the predicted second-order rate constant for the olefin.

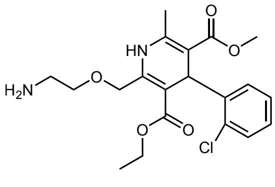

Amlodipine

SI-B4.13 1,7-Dimethyluric acid

Due to the complex heterocyclic ring structure of 1,7-dimethyluric acid, the second-order rate constant was predicted from the parent compound caffeine and equals to $3.0 \times 10^4\,\mathrm{M^{-1}s^{-1}}$. The measured $k_{\mathrm{app,O_3}}$ value is however three orders of magnitude higher and amounts to $(6.6 \pm 1.4){\times}10^7\,\mathrm{M^{-1}s^{-1}}$. In both compounds, the double bond connecting the two rings is assumed to be responsible for the reaction. With pK_a values of 7.50 and 10.46 for the two carbamides,[52] 24 % of 1,7-dimethyluric acid is present as anionic species. This leads to an electron-donating effect towards the olefin, explaining the increased reactivity compared to caffeine. Alternatively, the negatively charged nitrogen may directly react with ozone, as its high electron density renders it highly ozone-reactive.

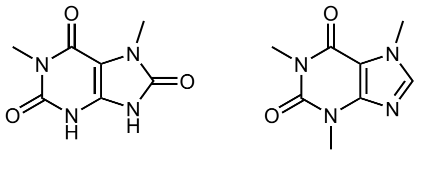

1,7-Dimethyluric acid   Caffeine

SI-B5 Parent vs metabolites

SI-B5.1 Hydroxylated vs non-hydroxylated

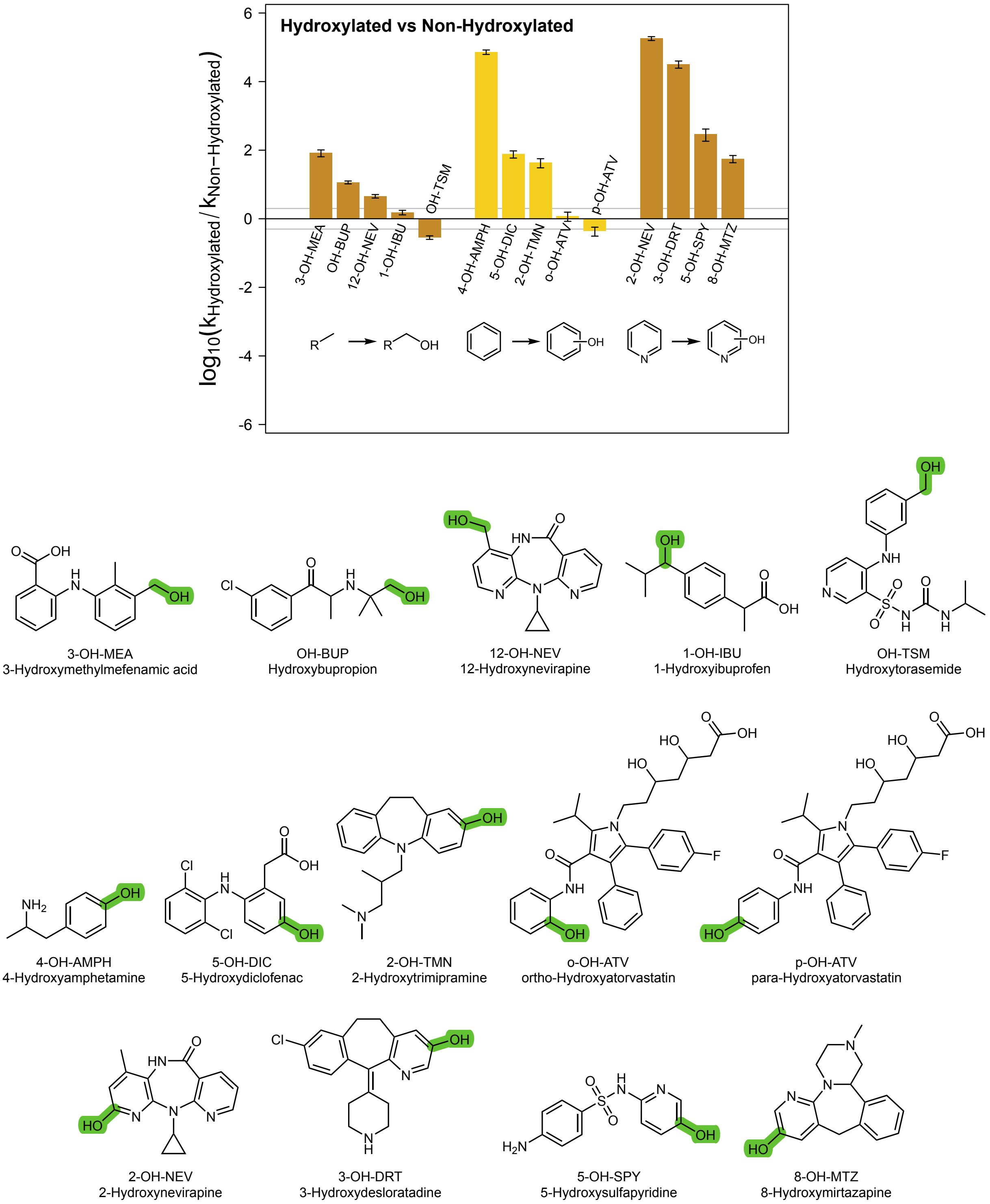

Figure SI-B85: Ratios of measured second-order rate constants for the reactions with ozone of hydroxylated metabolite-parent pairs. The gray lines indicate a reactivity difference of a factor two. The structures of the hydroxylated metabolites are shown below, with the hydroxyl moiety added compared to the parent highlighted in green.

SI-B5.2 *N*-Oxidized vs non-*N*-oxidized

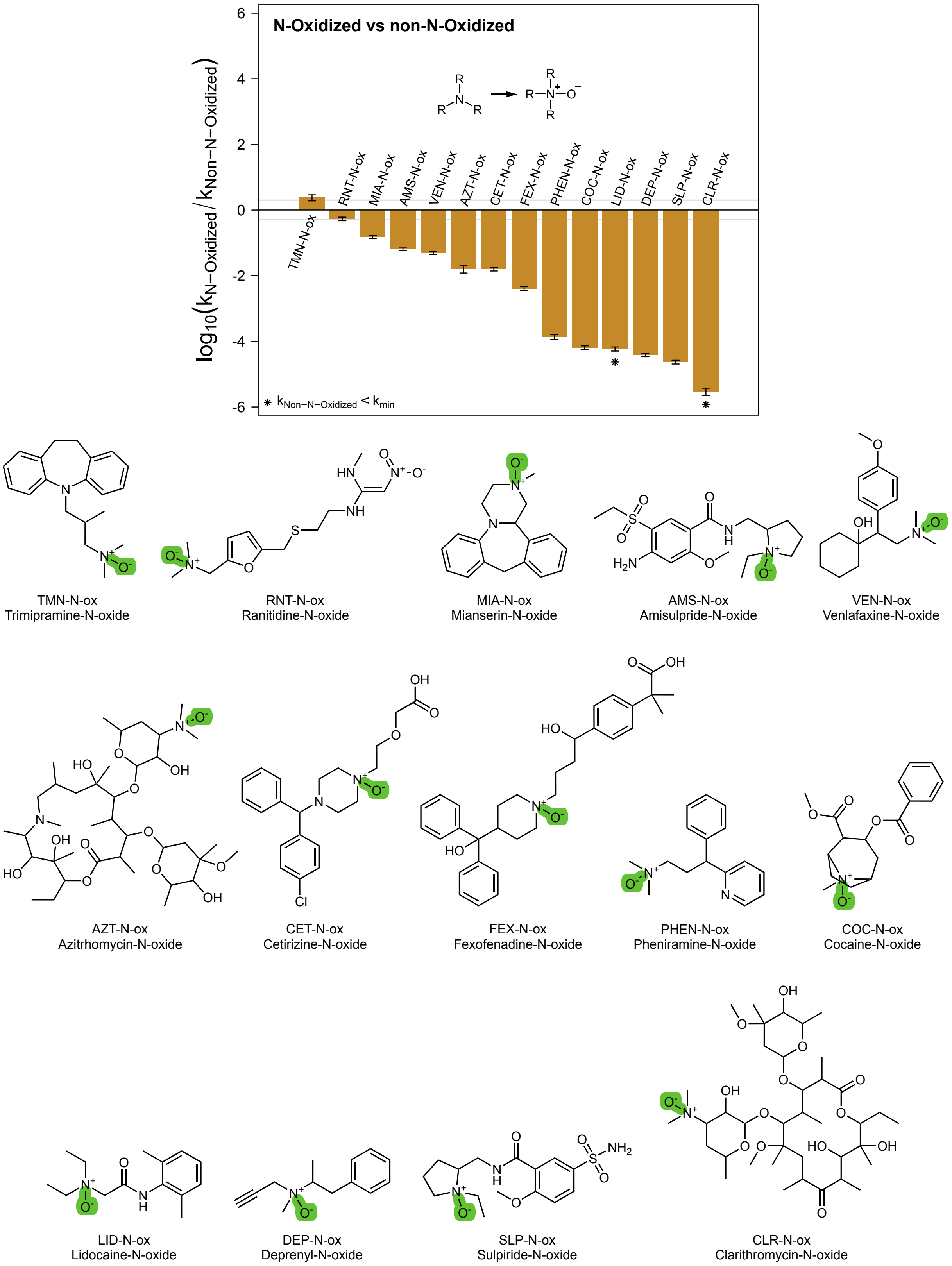

Figure SI-B86: Ratios of measured second-order rate constants for the reactions with ozone of *N*-oxide metabolite-parent pairs. The gray lines indicate a reactivity difference of a factor two. The structures of the *N*-oxide metabolites are shown below, with the the *N*-oxide moiety added compared to the parent highlighted in green.

SI-B5.3 Carboxylated vs non-carboxylated

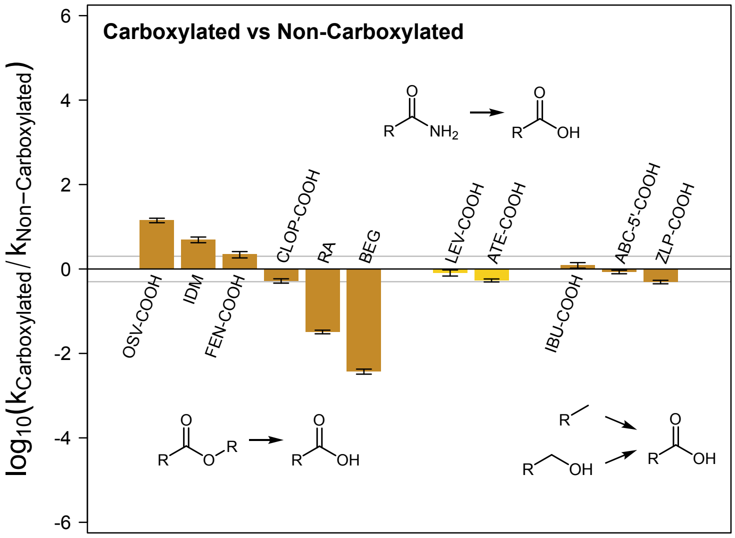

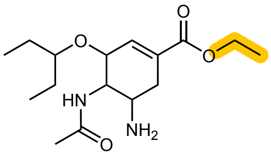

OSV-COOH
Oseltamivir acid

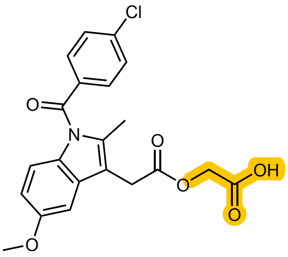

IDM
Indomethacin

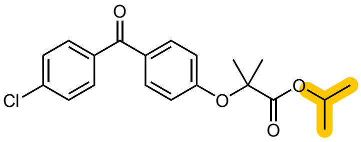

FEN-COOH
Fenofibric acid

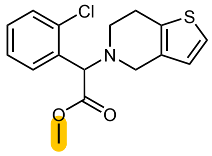

CLOP-COOH
Clopidogrel carboxylic acid

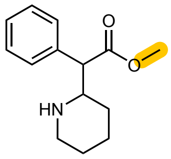

RA
Ritalinic acid

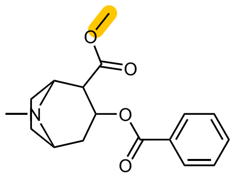

BEG
Benzoylecgonine

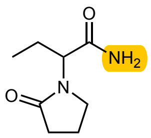

LEV-COOH
Levetiracetam acid

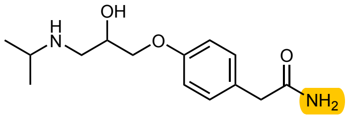

ATE-COOH
Atenolol acid

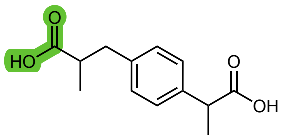

IBU-COOH
Carboxyibuprofen

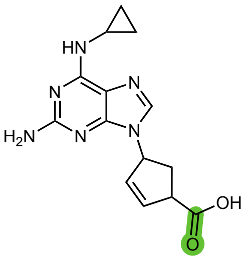

ABC-5'-COOH
Abacavir-5'-carboxylate

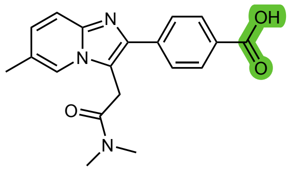

ZLP-COOH
Zolpidem carboxylic acid

Figure SI-B87: Ratios of measured second-order rate constants for the reactions with ozone of carboxylated metabolite-parent pairs. The gray lines indicate a reactivity difference of a factor two. The structures are shown below, with the moieties removed compared to the parent highlighted in yellow and the moieties added compared to the parent in green.

SI-B5.4 Conjugated vs non-conjugated

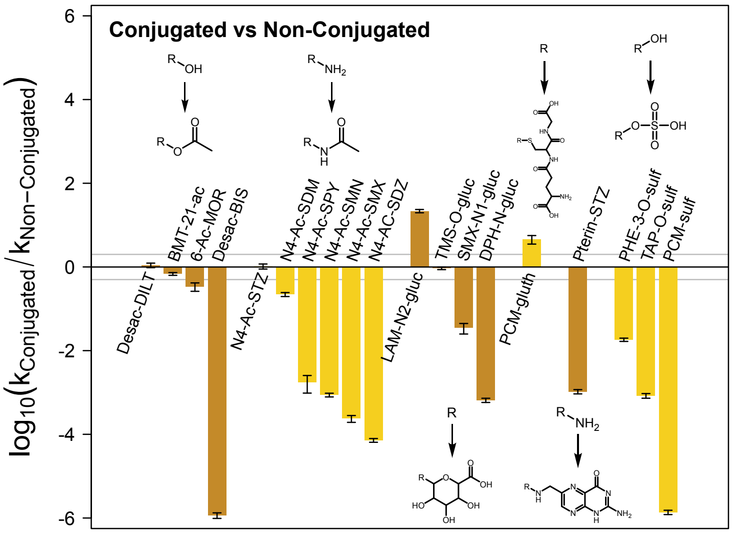

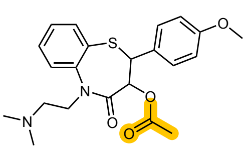

Desac-DILT
Desacetyldiltiazem

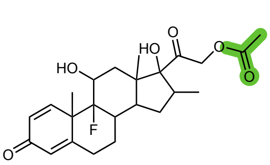

BMT-21-ac
Betamethasone-21-acetate

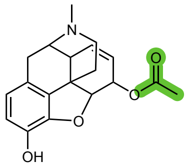

6-Ac-MOR
6-Acetylmorphine

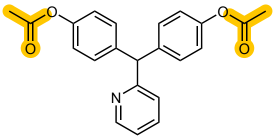

Desac-BIS
Desacetylbisacodyl

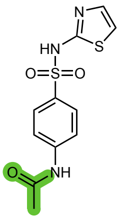

N4-Ac-STZ
N4-Acetylsulfathiazole

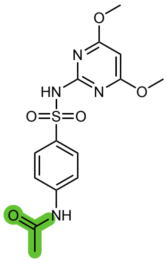

N4-Ac-SDM
N4-Acetylsulfadimethoxine

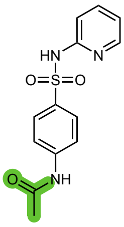

N4-Ac-SPY
N4-Acetylsulfapyridine

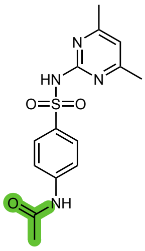

N4-Ac-SMN
N4-Acetylsulfamethazine

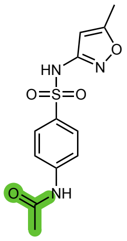

N4-Ac-SMX
N4-Acetylsulfamethoxazole

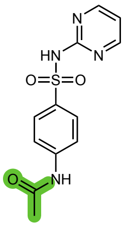

N4-Ac-SDZ
N4-Acetylsulfadiazine

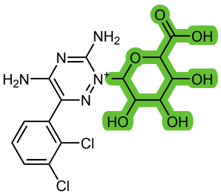

LAM-N2-gluc
Lamotrigine-N2-glucuronide

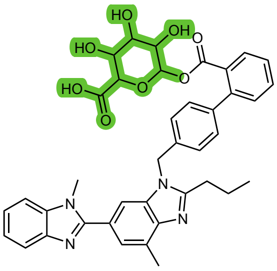

TSM-O-gluc
Telmisartan-O-acyl-glucuronide

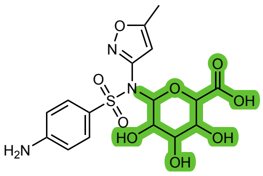

SMX-N1-gluc
Sulfamethoxazole-N1-glucuronide

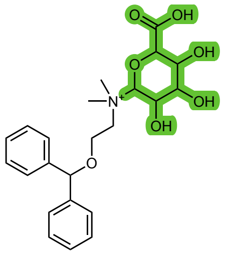

DPH-N-gluc
Diphenhydramine-N-glucuronide

Figure SI-B88: Ratios of measured second-order rate constants for the reactions with ozone of conjugated metabolite-parent pairs. The gray lines indicate a reactivity difference of a factor two. The comparison also includes the parents bisacodyl and diltiazem, as well as the metabolites desacetylbiscaodyl and desacetyldiltiazem. As for the other compound pairs, the second-order rate constant of the acetylated/conjugated compound is divided by the one of the non-acetylated/non-conjugated compound. Compared to the other pairs however, the roles of parent and metabolite are reversed. The structures are shown below, with the moieties removed compared to the parent highlighted in yellow and the moieties added compared to the parent in green. Figure continued on next page.

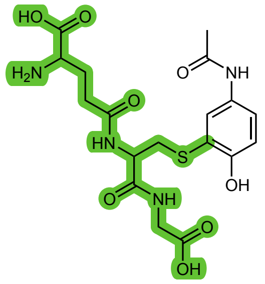

PCM-gluth
Paracetamol-glutathione

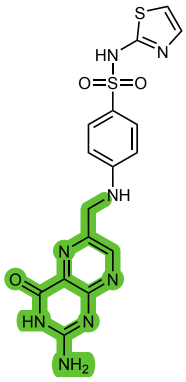

Pterin-STZ
Pterinsulfathiazole

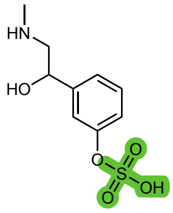

PHE-3-O-sulf
Phenylephrine-3-O-sulfate

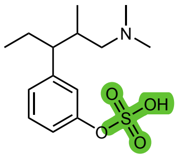

TAP-O-sulf
Tapentadol-O-sulfate

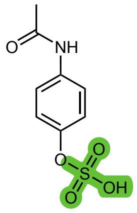

PCM-O-sulf
Paracetamol-O-sulfate

Figure SI-B88 continued.

SI-B5.5 Dealkylated vs non-dealkylated

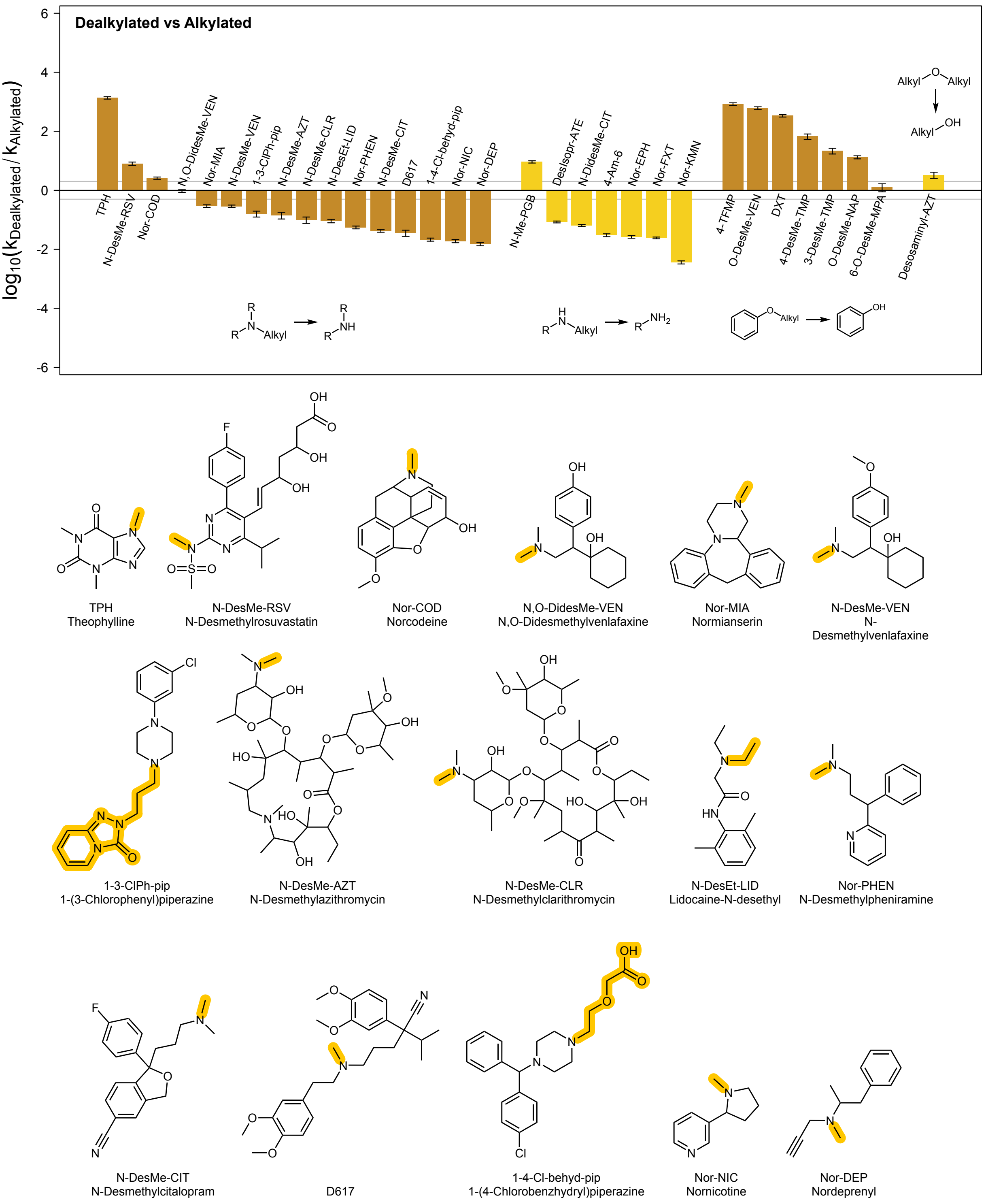

Figure SI-B89: Ratios of measured second-order rate constants for the reactions with ozone of dealkylated metabolite-parent pairs. The gray lines indicate a reactivity difference of a factor two. The comparison also includes the parent pregabalin and the conjugated metabolite *N*-methylpregabalin. As for the other compound pairs, the second-order rate constant of the dealkylated compound is divided by the one of the alkylated compound. Compared to the other pairs however, the roles of parent and metabolite are reversed. The structures are shown below, with the moieties removed compared to the parent highlighted in yellow and the moieties added compared to the parent in green. Figure continued on next page.

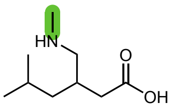

N-Me-PGB
N-Methylpregabalin

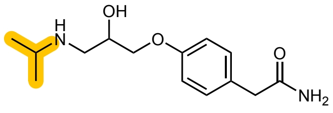

DesIsopr-ATE
Desisopropylatenolol

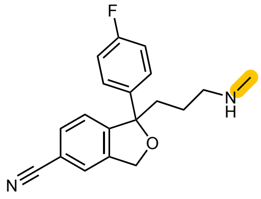

N-DidesMe-CIT
N-Didesmethylcitalopram

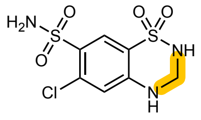

4-Am-6
4-Amino-6-chlorobenzene-1,3-disulfonamide

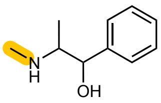

Nor-EPH
Norephedrine

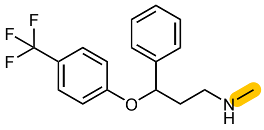

Nor-FXT
Norfluoxetine

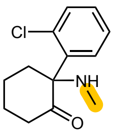

Nor-KMN
Norketamine

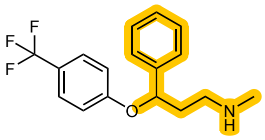

4-TFMP
4-Trifluoromethylphenol

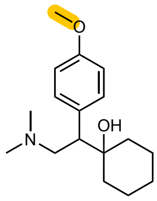

O-DesMe-VEN
O-Desmethylvenlafaxine

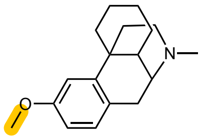

DXT
Dextrorphan

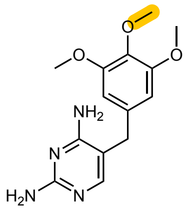

4-DesMe-TMP
4-Desmethyltrimethoprim

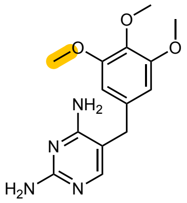

3-DesMe-TMP
3-Desmethyltrimethoprim

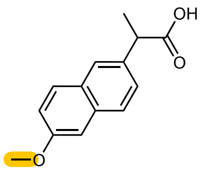

O-DesMe-NAP
O-Desmethylnaproxen

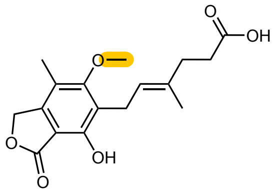

6-O-DesMe-MPA
6-O-Desmethylmycophenolic acid

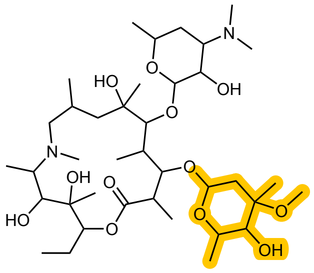

Desosaminyl-AZT
Desosaminylazithromycin

Figure SI-B89 continued.

SI-B6 $k^{\bullet}_{\mathrm{OH}}$ Prediction

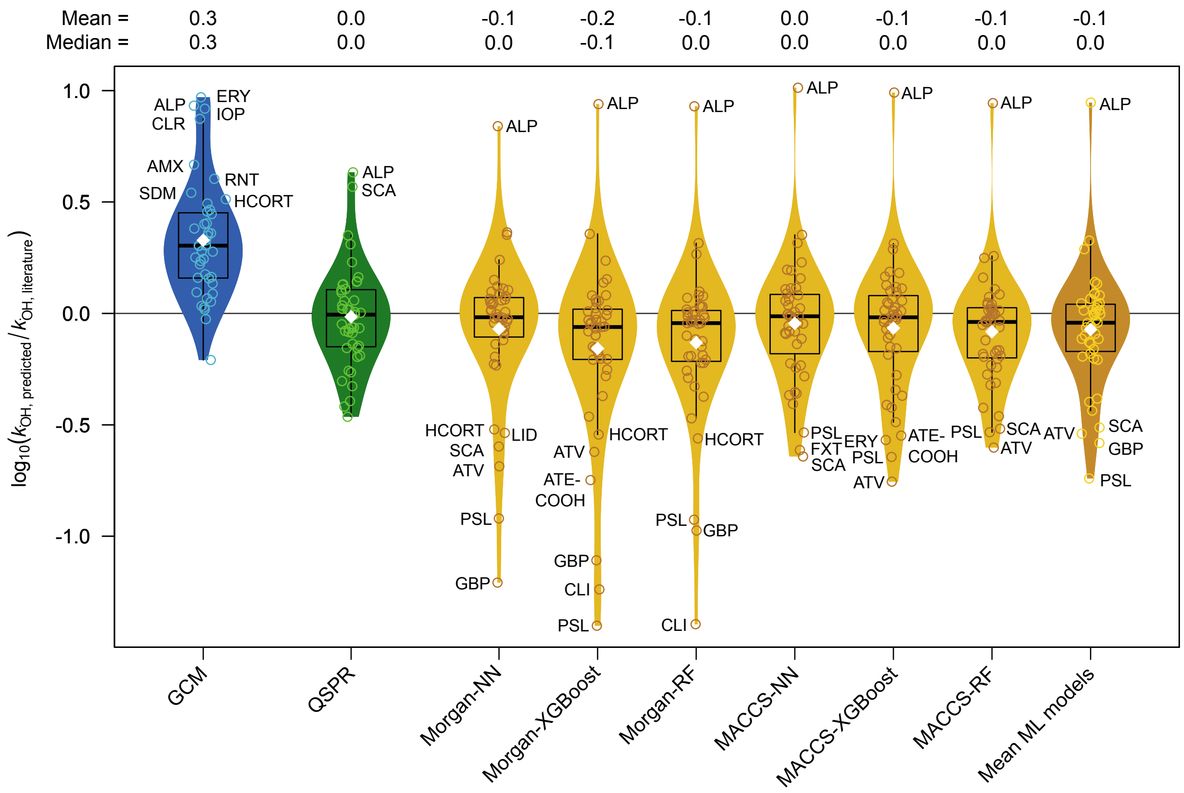

Figure SI-B90: Prediction of $k^{\bullet}_{\mathrm{OH}}$. Violin plots of the logarithmic ratios of literature and predicted $k^{\bullet}_{\mathrm{OH}}$ values of 44 compounds. The white diamonds indicate the mean values. Compounds with more than a factor two deviation from literature are labeled: allopurinol (ALP), amoxicillin (AMX), atenolol acid (ATE-COOH), atrovastatin (ATV), clarithromycin (CLR), clindamycin (CLI), erythromycin (ERY), fluoxetine (FXT), gabapentin (GBP), hydrocortisone (HCORT), iopromide (IOP), lidocaine (LID), prednisolone (PSL), succinic acid (SCA), ranitidine (RNT) and sulfadimethoxine (SDM).

SI-B7 Relative Contribution of $^{\bullet}$OH Scavengers

The relative contribution to $^{\bullet}$OH scavenging was calculated by Equations 0.11 and 0.12. Table SI-B9 provides the second-order rate constants for the reaction with DOM, (bi)carbonate (alkalinity), nitrite and bromide and the resulting relative contributions of the four matrix components to $^{\bullet}$OH scavenging.

$$k_{\text{scaveng, tot}} = k_{\text{DOM}}[\text{DOM}] + k_{\text{Alkalinity}}[\text{Alkalinity}] + k_{\text{Nitrite}}[\text{Nitrite}] + k_{\text{Bromide}}[\text{Bromide}] \tag{0.11}$$

$$\text{Relative Contribution} = \frac{k_i[X_i]}{k_{\text{scaveng, tot}}} \tag{0.12}$$

Table SI-B9: Second-order rate constants of $^{\bullet}$OH with DOM, (bi(carbonate) alkalinity, nitrite and bromide and the relative contributions of $^{\bullet}$OH scavengers in the three wastewater matrices

**Scavenger**	$k_{^{\bullet}\mathbf{OH}}$ [$\text{M}^{-1}\text{s}^{-1}$]	**Altenrhein** [%]	**Neugut** [%]	**Werdhoelzli** [%]
DOM	3.5×10^{4} [63]	80.5	74.4	72.5
Alkalinity	9.06×10^{6} [10]	13.3	23.2	10.0
Nitrite	1×10^{10} [10]	5.9	2.1	17.3
Bromide	1×10^{9}	0.3	0.3	0.2

SI-B8 Measured vs Predicted Abatement

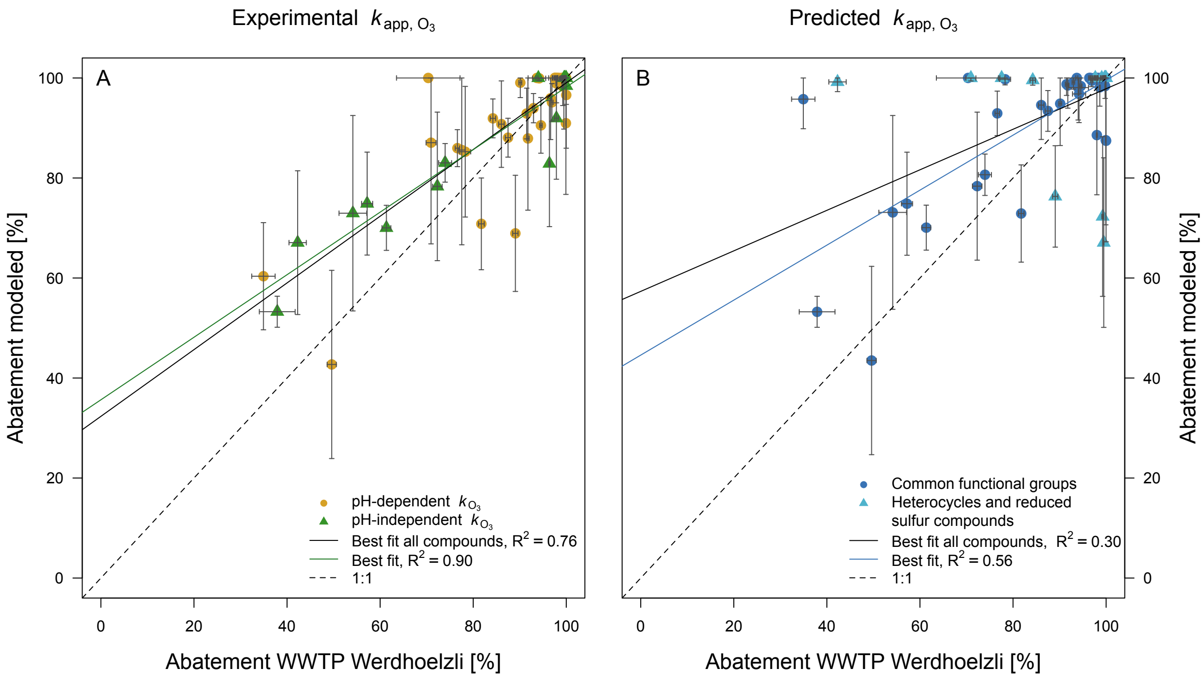

Figure SI-B91: Measured relative abatements of studied compounds in the ozonation of WWTP Werdhoelzli versus modeled relative abatements using experimental k_{O_3} (A) or predicted k_{O_3} values (B). The specific ozone dose was 0.6 g_{O_3}/g_{DOC} and the ozone exposure was set to 2.8×10^{-4} Ms. The error bars of the model were determined with Monte Carlo sampling, while the error bars from the WWTP removal correspond to standard deviations from the triplicate analysis and the five consecutive sampling days. Compounds with common functional groups refer to anilines, amines, benzenes, olefins and phenol(ate)s.

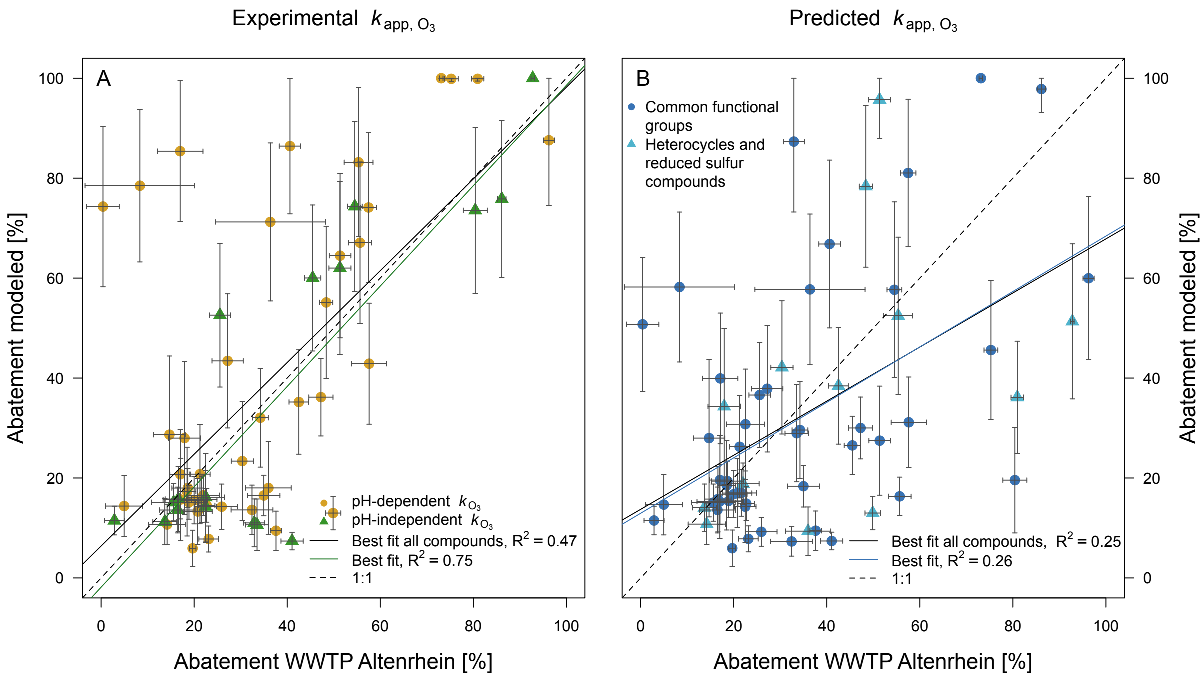

Figure SI-B92: Measured relative abatements of studied compounds in the ozonation of WWTP Altenrhein versus modeled relative abatements using experimental k_{O_3} (A) or predicted k_{O_3} values (B). The specific ozone dose was $0.1\,g_{O_3}/g_{DOC}$ and the ozone exposure was set to 1.3×10^6 Ms. The error bars of the model were determined with Monte Carlo sampling, while the error bars from the WWTP removal correspond to standard deviations from the triplicate analysis and the five consecutive sampling days. Compounds with common functional groups refer to anilines, amines, benzenes, olefins and phenol(ate)s.

SI-B9 Sensitivity analysis

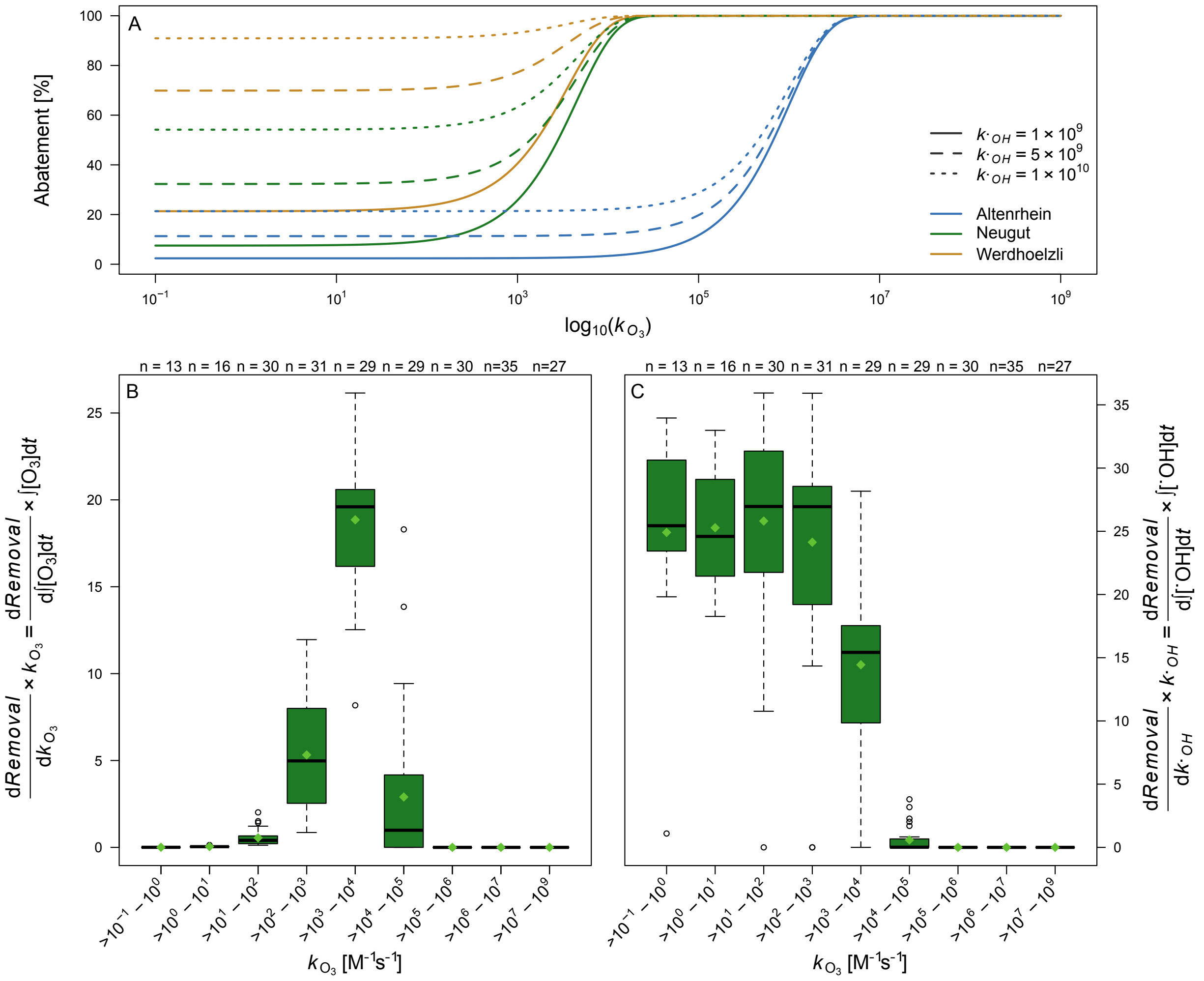

Figure SI-B93: Influence of k_{O_3}, $k_{^\bullet OH}$, O_3 exposure and $^\bullet$OH exposure on abatement of micropollutants during wastewater ozonation. (A) Abatement during ozonation as a function of k_{O_3} for the three WWTPs Altenrhein, Neugut and Werdhoelzli, assuming their $^\bullet$OH and O_3 exposures as constant based on the laboratory experiments and literature values, respectively. Three different $k_{^\bullet OH}$ values in the predicted range were considered. (B) Derivative-based local sensitivity analysis with respect to k_{O_3} and O_3 exposure and (C) with respect to $k_{^\bullet OH}$ and $^\bullet$OH exposure for WWTP Neugut. Light green diamonds indicate mean values.

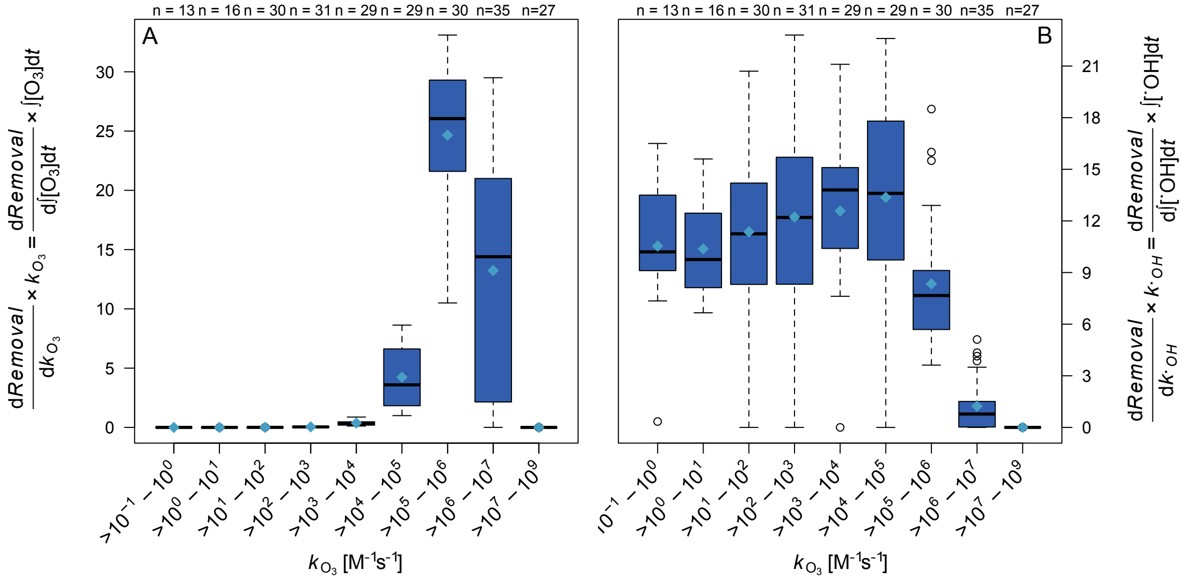

Figure SI-B94: Influence of k_{O_3}, $k_{^\bullet OH}$, O_3 exposure and $^\bullet$OH exposure on abatement of micropollutants during wastewater ozonation. Derivative-based local sensitivity analysis with respect to k_{O_3} and O_3 exposure (B) and with respect to $k_{^\bullet OH}$ and $^\bullet$OH exposure (C) for WWTP Altenrhein. Light blue diamonds indicate mean values.

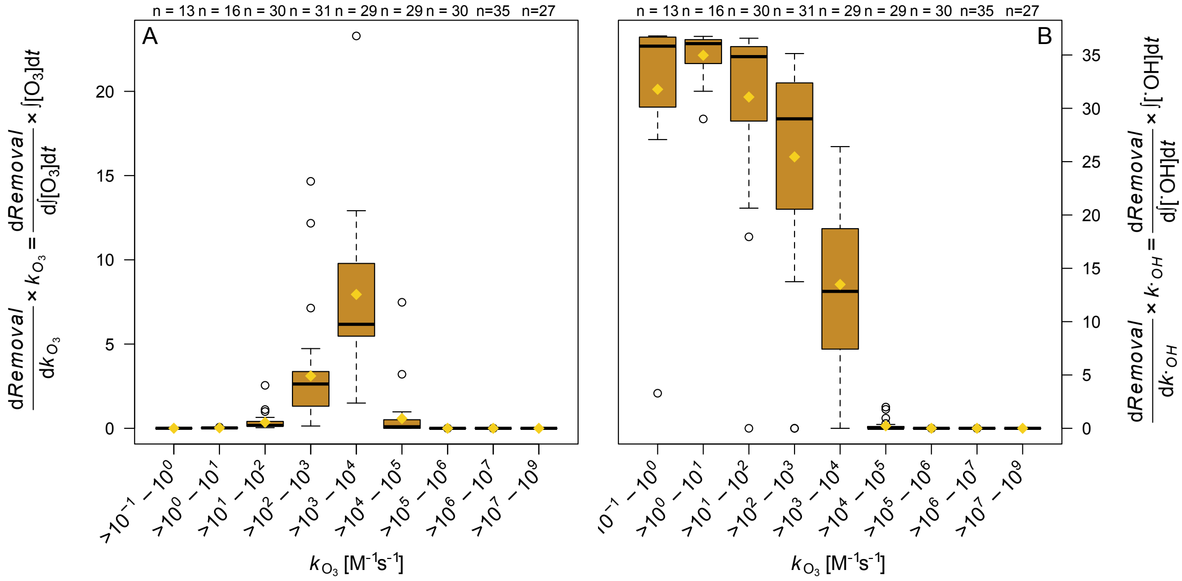

Figure SI-B95: Influence of k_{O_3}, $k_{^\bullet OH}$, O_3 exposure and $^\bullet$OH exposure on abatement of micropollutants during wastewater ozonation. Derivative-based local sensitivity analysis with respect to k_{O_3} and O_3 exposure (B) and with respect to $k_{^\bullet OH}$ and $^\bullet$OH exposure (C) for WWTP Werdhoelzli. Yellow diamonds indicate mean values.

Bibliography

[1] Rougé, V.; von Gunten, U.; Janssen, E. M. Reactivity of cyanobacteria metabolites with ozone: Multicompound competition kinetics. *Environmental Science and Technology* **2024**, *58*, 11802–11811.

[2] David Yao, C. C.; Haag, W. R. Rate constants for direct reactions of ozone with several drinking water contaminants. *Water Research* **1991**, *25*, 761–773.

[3] Huber, M. M.; Canonica, S.; Park, G. Y.; von Gunten, U. Oxidation of pharmaceuticals during ozonation and advanced oxidation processes. *Environmental Science and Technology* **2003**, *37*, 1016–1024.

[4] Wolf, C.; von Gunten, U.; Kohn, T. Kinetics of inactivation of waterborne enteric viruses by ozone. *Environmental Science and Technology* **2018**, *52*, 2170–2177.

[5] Lee, W.; Marcotullio, S.; Yeom, H.; Son, H.; Kim, T. H.; Lee, Y. Reaction kinetics and degradation efficiency of halogenated methylparabens during ozonation and UV/H2O2 treatment of drinking water and wastewater effluent. *Journal of Hazardous Materials* **2022**, *427*, 127878.

[6] Dodd, M. C.; Buffle, M. O.; von Gunten, U. Oxidation of antibacterial molecules by aqueous ozone: Moiety-specific reaction kinetics and application to ozone-based wastewater treatment. *Environmental Science and Technology* **2006**, *40*, 1969–1977.

[7] Zimmermann, S. G.; Schmukat, A.; Schulz, M.; Benner, J.; von Gunten, U.; Ternes, T. A. Kinetic and mechanistic investigations of the oxidation of tramadol by ferrate and ozone. *Environmental Science and Technology* **2012**, *46*, 876–884.

[8] Suarez, S.; Dodd, M. C.; Omil, F.; von Gunten, U. Kinetics of triclosan oxidation by aqueous ozone and consequent loss of antibacterial activity: Relevance to municipal wastewater ozonation. *Water Research* **2007**, *41*, 2481–2490.

[9] Lee, Y.; Gerrity, D.; Lee, M.; Bogeat, A. E.; Salhi, E.; Gamage, S.; Trenholm, R. A.; Wert, E. C.; Snyder, S. A.; von Gunten, U. Prediction of micropollutant elimination during ozonation of municipal wastewater effluents: Use of kinetic and water specific information. *Environmental Science and Technology* **2013**, *47*, 5872–5881.

[10] Buxton, G.; Greenstock, C.; Helman, W. P.; Ross, A.; Helman, W. Critical review of aqueous solution reaction rate constants for hydrogen atoms and hydroxyl radicals (OH/O-) in aqueous solution. *Journal of Physical and Chemical Reference Data* **1988**, *17*, 663.

[11] Jin, X.; Peldszus, S.; Huck, P. M. Predicting the reaction rate constants of micropollutants with hydroxyl radicals in water using QSPR modeling. *Chemosphere* **2015**, *138*, 1–9.

[12] Cao, D. S.; Xu, Q. S.; Hu, Q. N.; Liang, Y. Z. ChemoPy: Freely available python package for computational biology and chemoinformatics. *Bioinformatics* **2013**, *29*, 1092–1094.

[13] Dong, J.; Cao, D. S.; Miao, H. Y.; Liu, S.; Deng, B. C.; Yun, Y. H.; Wang, N. N.; Lu, A. P.; Zeng, W. B.; Chen, A. F. ChemDes: An integrated web-based platform for molecular descriptor and fingerprint computation. *Journal of Cheminformatics* **2015**, *7*, 1–10.

[14] Mauri, A. In *Ecotoxicological QSARs*, 1st ed.; Roy, K., Ed.; Humana Press, 2020; pp 801–820.

[15] Sadowski, J.; Gasteiger, J.; Erlangen, D.; Klebe, G.; Ludwigshafen, D. Comparison of automatic three-dimensional model builders using 639 X-ray structures. *Journal of Chemical Information and Computer Sciences* **1994**, *34*, 1000–1008.

[16] Schwab, C. H. Conformations and 3D pharmacophore searching. *Drug Discovery Today: Technologies* **2010**, *7*, 245–253.

[17] 3D structure generator CORINA Classic. `www.mn-am.com`.

[18] Morgan, H. L. The generation of a unique machine description for chemical structures – A technique developed at chemical abstracts service. *Journal of Chemical Documentation* **1965**, *5*, 107–113.

[19] Durant, J. L.; Leland, B. A.; Henry, D. R.; Nourse, J. G. Reoptimization of MDL keys for use in drug discovery. *Journal of Chemical Information and Computer Sciences* **2002**, *42*, 1273–1280.

[20] Hansen, L. K.; Salamon, P. Neural network ensembles. *IEEE Transactions on Pattern Analysis and Machine Intelligence* **1990**, *12*, 993–1001.

[21] Ho, T. K. Random decision forests. *Proceedings of 3rd International Conference on Document Analysis and Recognition* **1995**, *1*, 278282.

[22] Chen, T.; Guestrin, C. XGBoost: A scalable tree boosting system. *Proceedings of the ACM SIGKDD International Conference on Knowledge Discovery and Data Mining* **2016**, 785–794.

[23] Sanches-Neto, F. O.; Dias-Silva, J. R.; Keng Queiroz Junior, L. H.; Carvalho-Silva, V. H. pySiRC: Machine learning combined with molecular fingerprints to predict the reaction rate constant of the radical-based oxidation processes of aqueous organic contaminants. *Environmental Science and Technology* **2021**, *55*, 12437–12448.

[24] Sanches-Neto, F. O.; Dias-Silva, J. R.; de Oliveira, V. M.; Aquilanti, V.; Carvalho-Silva, V. H. Evaluating and elucidating the reactivity of OH radicals with atmospheric organic pollutants: Reaction kinetics and mechanisms by machine learning. *Atmospheric Environment* **2022**, *275*.

[25] Meyer, C.; Stravs, M. A.; Hollender, J. How wastewater reflects human metabolism – Suspect screening of pharmaceutical metabolites in wastewater influent. *Environmental Science and Technology* **2024**, *58*, 9828–9839.

[26] Meyer, C.; McArdell, C. S.; Fenner, K.; Joss, A.; Hollender, J. Comparing the abatement of pharmaceuticals and their human metabolites in wastewater treatment plants – Insights from biological and advanced treatment stages. *Water Research, in review* **2025**,

[27] Zucker, I.; Mamane, H.; Riani, A.; Gozlan, I.; Avisar, D. Formation and degradation of N-oxide venlafaxine during ozonation and biological post-treatment. *Science of the Total Environment* **2018**, *619-620*, 578–586.

[28] Dodd, M. C.; Kohler, H. P. E.; von Gunten, U. Oxidation of antibacterial compounds by ozone and hydroxyl radical: Elimination of biological activity during aqueous ozonation processes. *Environmental Science and Technology* **2009**, *43*, 2498–2504.

[29] Javier Benitez, F.; Acero, J. L.; Real, F. J.; Roldán, G. Ozonation of pharmaceutical compounds: Rate constants and elimination in various water matrices. *Chemosphere* **2009**, *77*, 53–59.

[30] Hamdi El Najjar, N.; Touffet, A.; Deborde, M.; Journel, R.; Karpel Vel Leitner, N. Kinetics of paracetamol oxidation by ozone and hydroxyl radicals, formation of transformation products and toxicity. *Separation and Purification Technology* **2014**, *136*, 137–143.

[31] Benner, J.; Salhi, E.; Ternes, T.; von Gunten, U. Ozonation of reverse osmosis concentrate: Kinetics and efficiency of beta blocker oxidation. *Water Research* **2008**, *42*, 3003–3012.

[32] Borowska, E.; Bourgin, M.; Hollender, J.; Kienle, C.; McArdell, C. S.; von Gunten, U. Oxidation of cetirizine, fexofenadine and hydrochlorothiazide during ozonation: Kinetics and formation of transformation products. *Water Research* **2016**, *94*, 350–362.

[33] Sein, M. M.; Zedda, M.; Tuerk, J.; Schmidt, T. C.; Golloch, A.; von Sonntag, C. Oxidation of diclofenac with ozone in aqueous solution. *Environmental Science and Technology* **2008**, *42*, 6656–6662.

[34] Real, F. J.; Javier Benitez, F.; Acero, J. L.; Sagasti, J. J.; Casas, F. Kinetics of the chemical oxidation of the pharmaceuticals primidone, ketoprofen, and diatrizoate in ultrapure and natural waters. *Industrial and Engineering Chemistry Research* **2009**, *48*, 3380–3388.

[35] Jeon, D.; Kim, J.; Shin, J.; Hidayat, Z. R.; Na, S.; Lee, Y. Transformation of ranitidine during water chlorination and ozonation: Moiety-specific reaction kinetics and elimination efficiency of NDMA formation potential. *Journal of Hazardous Materials* **2016**, *318*, 802–809.

[36] Bourgin, M.; Beck, B.; Boehler, M.; Borowska, E.; Fleiner, J.; Salhi, E.; Teichler, R.; von Gunten, U.; Siegrist, H.; McArdell, C. S. Evaluation of a full-scale wastewater treatment plant upgraded with ozonation and biological post-treatments: Abatement of micropollutants, formation of transformation products and oxidation by-products. *Water Research* **2018**, *129*, 486–498.

[37] Lange, F.; Cornelissen, S.; Kubac, D.; Sein, M. M.; von Sonntag, J.; Hannich, C. B.; Golloch, A.; Heipieper, H. J.; Möder, M.; von Sonntag, C. Degradation of macrolide antibiotics by ozone: A mechanistic case study with clarithromycin. *Chemosphere* **2006**, *65*, 17–23.

[38] Huber, M. M.; Göbel, A.; Joss, A.; Hermann, N.; Löffler, D.; McArdell, C. S.; Ried, A.; Siegrist, H.; Ternes, T. A.; von Gunten, U. Oxidation of pharmaceuticals during ozonation of municipal wastewater effluents: A pilot study. *Environmental Science and Technology* **2005**, *39*, 4290–4299.

[39] Hoigné, J.; Bader, H. Rate constants of reactions of ozone with organic and inorganic compounds in water – II. dissociating organic compounds. *Water Research* **1983**, *17*, 185–194.

[40] Mestankova, H.; Schirmer, K.; Escher, B. I.; von Gunten, U.; Canonica, S. Removal of the antiviral agent oseltamivir and its biological activity by oxidative processes. *Environmental Pollution* **2012**, *161*, 30–35.

[41] Favier, M.; Dewil, R.; Van Eyck, K.; Van Schepdael, A.; Cabooter, D. High-resolution MS and MSn investigation of ozone oxidation products from phenazone-type pharmaceuticals and metabolites. *Chemosphere* **2015**, *136*, 32–41.

[42] Keen, O. S.; Ferrer, I.; Michael Thurman, E.; Linden, K. G. Degradation pathways of lamotrigine under advanced treatment by direct UV photolysis, hydroxyl radicals, and ozone. *Chemosphere* **2014**, *117*, 316–323.

[43] Tentscher, P. R.; Escher, B. I.; Schlichting, R.; König, M.; Bramaz, N.; Schirmer, K.; von Gunten, U. Toxic effects of substituted p-benzoquinones and hydroquinones in in vitro bioassays are altered by reactions with the cell assay medium. *Water Research* **2021**, *202*.

[44] Mvula, E.; Von Sonntag, C. Ozonolysis of phenols in aqueous solution. *Organic and Biomolecular Chemistry* **2003**, *1*, 1749–1756.

[45] Wang, Y.; Rodriguez, F. E. M.; Rentsch, D.; Qiang, Z.; von Gunten, U. Ozone reactions with olefins and alkynes: kinetics, activation energies, and mechanisms. *Environmental Science & Technology* **2025**, *59*, 4733–4744.

[46] Acero, J. L.; Von Gunten, U. Influence of carbonate on the ozone/hydrogen peroxide based advanced oxidation process for drinking water treatment. *Ozone: Science and Engineering* **2000**, *22*, 305–328.

[47] Perez-Benito, J. F. Reaction pathways in the decomposition of hydrogen peroxide catalyzed by copper(II). *Journal of Inorganic Biochemistry* **2004**, *98*, 430–438.

[48] Rodríguez, E. M.; von Gunten, U. Generation of hydroxyl radical during chlorination of hydroxyphenols and natural organic matter extracts. *Water Research* **2020**, *177*.

[49] Barron, E.; Deborde, M.; Rabouan, S.; Mazellier, P.; Legube, B. Kinetic and mechanistic investigations of progesterone reaction with ozone. *Water Research* **2006**, *40*, 2181–2189.

[50] O'Neil, M., Ed. *The Merck Index – An encyclopedia of chemicals, drugs, and biologicals*, 13th ed.; Whitehouse Station, NJ: Merck and Co., Inc.„ 2001; p 1163.

[51] von Sonntag, C.; von Gunten, U. *Chemistry of ozone in water and wastewater treatment: From basic principles to applications*; IWA Publishing, 2012.

[52] ChemAxon JChem for Office. `https://chemaxon.com/products/jchem-for-office`.

[53] Kolthoff, I. Die Dissoziationskonstante, das Löslichkeitsprodukt und die Titrierbarkeit von Alkaloiden. *Biochemische Zeitschrift* **1925**, *162*, 289.

[54] Morgenthaler, M.; Schweizer, E.; Hoffmann-Röder, A.; Benini, F.; Martin, R. E.; Jaeschke, G.; Wagner, B.; Fischer, H.; Bendels, S.; Zimmerli, D.; Schneider, J.; Diederich, F.; Kansy, M.; Müller, K. Predicting and tuning physicochemical properties in lead optimization: Amine basicities. *ChemMedChem* **2007**, *2*, 1100–1115.

[55] Settimo, L.; Bellman, K.; Knegtel, R. M. Comparison of the accuracy of experimental and predicted pKa values of basic and acidic compounds. *Pharmaceutical Research* **2014**, *31*, 1082–1095.

[56] Osol, A., Chas, G. D., Gennaro, A. R., Gibson, M. R., Granberg, C. B., Harvey, S. C., King, R. E., Martin, A. N., Swinyard, E. A., Zink, G. L., Eds. *Remington's pharmaceutical sciences*, 16th ed.; Mack Publishing Co.: Eason, Pennsylvania, 1980; p 994.

[57] Lee, Y.; von Gunten, U. Quantitative structure-activity relationships (QSARs) for the transformation of organic micropollutants during oxidative water treatment. *Water Research* **2012**, *46*, 6177–6195.

[58] Rath, S. A.; Rougé, V.; Tolu, J.; Rentsch, D.; Halder, M. L.; von Gunten, U. Reactions of N,O- and N,S-azoles and -azolines with ozone. *Environmental Science and Technology, in revision* **2025**,

[59] Hoigné, J.; Bader, H. Rate constants of reactions of ozone with organic and inorganic compounds in water – I. Non-dissociating organic compounds. *Water Research* **1983**, *17*, 173–183.

[60] Pearce, P. J.; Simkins, R. J. J. Acid strengths of some substituted picric acids. *Canadian Journal of Chemistry* **1968**, *46*, 241–248.

[61] Rath, S. A.; Halder, M. L.; von Gunten, U. Oxidation kinetics of organic sulfur compounds in aqueous ozonation. *Water Research, in preparation*

[62] Tekle-Röttering, A. Ozonation of amines : Kinetics, stoichiometry, product formation and mechanistic considerations. Ph.D. thesis, Universität Duisburg-Essen, 2015.

[63] Katsoyiannis, I. A.; Canonica, S.; von Gunten, U. Efficiency and energy requirements for the transformation of organic micropollutants by ozone, O3/H2O2 and UV/H2O2. *Water Research* **2011**, *45*, 3811–3822.
